# Clinical, genetic, and immunologic features of APS-1 patients from the Middle East, and a review of the literature

**DOI:** 10.70962/jhi.20250254

**Published:** 2026-08-03

**Authors:** Elsa Lamah, Nouf Mohammed Althubaiti, Youmna El-Orfali, Hagop Mardirossian, Habib Alkalamouni, Abdulrahman N. Aljaber, Musaab Alhezam, Nader G. Zalaquett, Rana Mansour, Rima Hanna-Wakim, Fayhan Alroqi, Michel J. Massaad

**Affiliations:** 1 Faculty of Medicine, American University of Beirut, Beirut, Lebanon; 2 Division of Allergy and Immunology, Department of Pediatrics, King Abdulaziz Medical City, Riyadh, Saudi Arabia; 3 King Abdullah International Medical Research Center, Ministry of National Guard Health Affairs, King Saud Bin Abdulaziz University for Health Sciences, Riyadh, Saudi Arabia; 4 Department of Experimental Pathology, Immunology, and Microbiology, Faculty of Medicine, American University of Beirut, Beirut, Lebanon; 5 Division of Pediatric Infectious Diseases, Department of Pediatrics and Adolescent Medicine, American University of Beirut Medical Center, Beirut, Lebanon; 6 Center for Infectious Diseases Research, American University of Beirut, Beirut, Lebanon; 7 Research Center of Excellence in Immunity and Infections, American University of Beirut, Beirut, Lebanon

## Abstract

Autoimmune polyglandular syndrome type-1 (APS-1) results from mutations in autoimmune regulator (AIRE), a transcription factor that drives thymic expression of tissue-restricted antigens, thus enabling deletion of self-reactive thymocytes to maintain tolerance. APS-1 typically manifests with hypoparathyroidism, adrenal insufficiency, and candidiasis, but presentation varies. In this study, we characterized the clinical, genetic, and immunologic features of 18 new APS-1 patients and reviewed all reported cases to identify additional manifestations and mutational hotspots useful for targeted sequencing. We report three previously unpublished mutations, reduced T cell function, and diminished Treg numbers in our patients. Furthermore, we identified alopecia, thyroid disease, and diabetes mellitus as additional diagnostic clues, and revealed five recurrent variants that account for over 80% of known mutations. Our findings highlight additional clinical and laboratory features and emphasize key mutational hotspots that can accelerate the diagnosis to improve the timely and cost-effective identification of APS-1, especially in settings with limited awareness or resources.

## Teaser

The authors identify three novel mutations in AIRE and detect T cell defects in patients with APS-1. Furthermore, they review the literature and identify additional clinical features, and define mutational hotspots suitable for targeted sequencing. These parameters could facilitate the timely identification of APS-1, which impacts the early initiation of treatment.

## Introduction

Inborn errors of immunity are genetic disorders that affect the development and/or function of immune cells, leading to variable degrees of immunodeficiency and/or immunodysregulation (1). Autoimmune polyglandular syndrome type 1 (APS-1), also known as autoimmune polyendocrinopathy-candidiasis-ectodermal-dystrophy, is an immunodysregulation disorder that typically manifests during childhood with autoimmunity involving the endocrine and non-endocrine systems (1, 2). APS-1 is a rare disorder with an estimated global incidence of one case per 90,000–200,000 individuals and a total of 500–700 cases reported worldwide. However, this incidence is higher in relatively isolated populations such as Iranian Jews (1:9,000), Sardinians (1:14,000), Finns (1:25,000), and Slovenians (1: 43,000) (3).

**Figure 1.**
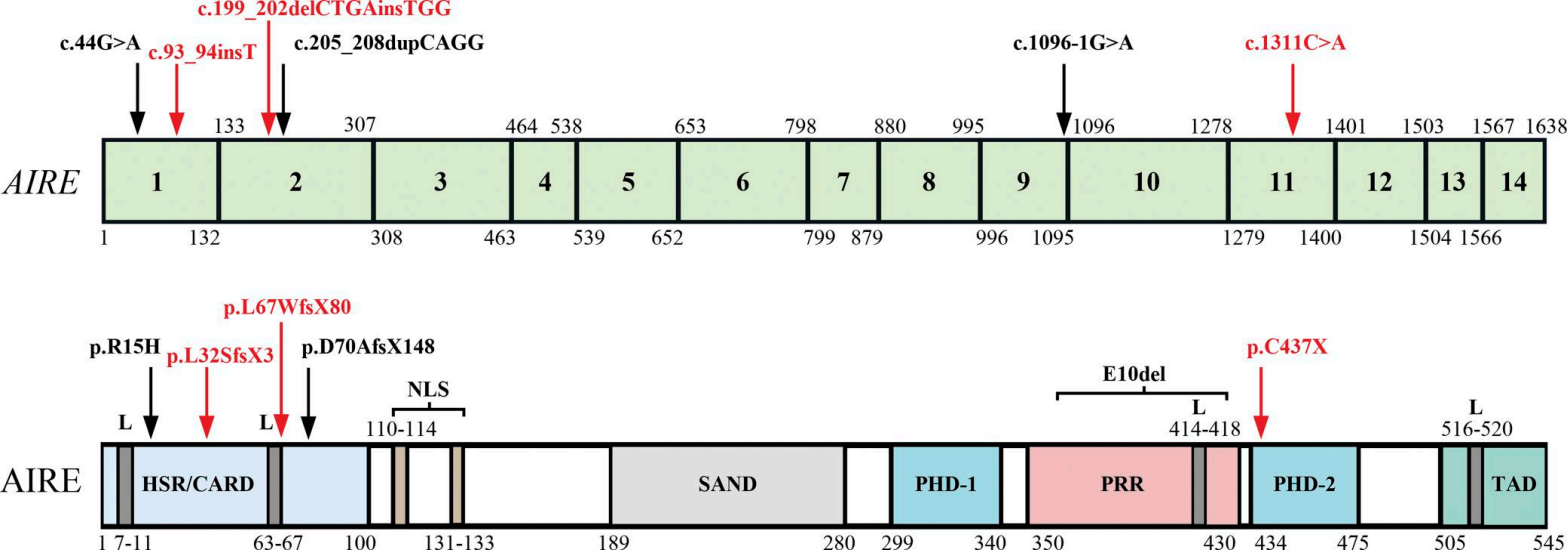
**Schematic representation of *AIRE* cDNA and protein.** Boxes represent exons or protein domains. Red arrows and text represent novel mutations, and black arrows and text represent previously reported mutations identified in our patients. HSR, homogeneously staining region; CARD, caspase activation and recruitment domain; NLS, nuclear localization signal; L, linker region; SAND, SP100, AIRE-1, NucP41/75, DEAF-1 domain; PHD, plant homeodomain; PRR, proline-rich region; TAD, transactivation domain.

APS-1 is caused by homozygous and dominant-negative heterozygous mutations in the autoimmune regulator (*AIRE*) gene (4, 5). *AIRE* is located on chromosome 21q22.3 and consists of 14 exons that encode a 545–amino acid (aa) transcriptional regulator with a molecular weight of 57.7 kilodalton (4). AIRE protein is composed of several functional domains (6) (Fig. 1) that include a caspase recruitment domain/homogeneously staining region (CARD/HSR) that promotes oligomerization (7, 8), a SP100, AIRE, Nuc p41/75, DEAF (SAND) domain important for transactivation (9) and subcellular localization (10), four LXXLL nuclear shuttling signals (7, 11), and two plant homeodomains (PHDs) zinc fingers separated by a proline-rich region (PRR) that mediates histone binding (PHD1) and gene transcription (PHD2) (12, 13, 14, 15).

AIRE is primarily expressed in medullary thymic epithelial cells (mTECs) (16); however, low levels have been detected in the secondary lymphoid organs and peripheral blood mononuclear cells (PBMCs) (17). In mTECs, AIRE exists in a multimeric transcription complex that induces the promiscuous expression or repression of a number of genes in the thymus, including those encoding tissue-restricted antigens (TRA) exclusively expressed in the periphery (6, 16, 18, 19, 20). TRA are presented as self-antigens on major histocompatibility complex (MHC) molecules to developing thymocytes either directly by mTECs or indirectly by dendritic cells, leading to the clonal deletion of self-reactive thymocytes (21) and the development of TRA-specific regulatory T cells (Tregs) (22), thereby preserving self-tolerance (23, 24). Patients with APS-1 carry pathogenic variants in *AIRE* that impair protein expression or function. These defects disrupt thymic expression of TRA and hinder the negative selection of autoreactive T cells, ultimately promoting the development of autoimmunity.

The classical clinical triad of APS-1 includes hypoparathyroidism, adrenal insufficiency, and chronic mucocutaneous candidiasis (25, 26). APS-1 is typically diagnosed when a patient presents with at least two of these three hallmark features. However, in cases where a sibling is suspected of having APS-1, the presence of only one of these features may suffice for diagnosis (27). Not all individuals with APS-1 meet these three classical diagnostic criteria. Some patients present with a broader spectrum of manifestations, such as gonadal insufficiency, autoimmune thyroiditis, autoimmune hepatitis, type 1 diabetes mellitus, vitiligo, gastrointestinal disturbances, keratitis, retinitis, enamel hypoplasia, dermatologic lesions, and an increased susceptibility to infections and neoplasms (25, 26, 28).

Because of its phenotypic variability, APS-1 can be separated into “classical” and “nonclassical” forms. Classical APS-1 typically manifests in childhood and is characterized by the presence of at least two of the three main clinical features. It is marked by the presence of autoantibodies to interferon α and ω and often follows an autosomal recessive inheritance pattern. In contrast, nonclassical APS-1 presents in either childhood or adulthood and is marked by a milder, less penetrant autoimmune phenotype. This form is usually associated with autosomal-dominant heterozygous mutations in the SAND and PHD1 zinc finger domains of AIRE (29, 30). Due to the poor genotype–phenotype correlation in APS-1 patients, accurate classification into classical and nonclassical forms cannot be reliably made based on clinical phenotype alone; rather, it depends on a combination of clinical manifestations and the identification of the underlying genetic etiology.

The heterogeneity in clinical manifestations suggests that the traditional diagnostic criteria may miss cases and delay treatment (2), potentially resulting in irreversible autoimmune damage or life-threatening events such as adrenal crisis. Furthermore, the lack of a laboratory assay for APS-1 renders genetic testing the only definitive method of diagnosis. However, access to next generation sequencing (NGS) remains limited in many regions. In this study, we report 18 newly identified patients with APS-1 from Saudi Arabia, Lebanon, and Syria, diagnosed based on clinical features supported by NGS. We also conducted a comprehensive literature review of all previously published APS-1 cases to highlight additional clinical features beyond the classical triad and determined the most frequently reported pathogenic *AIRE* variants to define mutational hotspots suitable for targeted Sanger sequencing. Additionally, we performed in-depth immunophenotyping and T cell functional analysis in 11 of our patients to explore laboratory parameters that could support the clinical recognition and diagnosis of APS-1.

## Results

### Demographic, clinical, and genetic information of the APS-1 patients identified in this study

Fifteen patients from Saudi Arabia (Pts. 1–15) descending from seven distinct families presented to King Abdulaziz Medical City, and three patients, Pts. 16–17 from a Lebanese family and Pt. 18 from a Syrian family, presented to the American University of Beirut Medical Center (Table 1). Their clinical picture included hypoparathyroidism (16/18), Addison’s disease (11/18), mucocutaneous and nail candidiasis (11/18), alopecia (6/18), diabetes mellitus (6/18), vitiligo (3/18), keratoconjunctivitis (3/18), hypothyroidism (2/18), ovarian failure (2/9 females), as well as other isolated symptoms (Table 1). All patients hailed from consanguineous families, were equally distributed between males and females, and their symptoms manifested at a relatively young age; therefore, their disease was suspected to be of genetic origin. Because the triad of hypoparathyroidism, Addison’s disease, and candidiasis were common among the patients, they were suspected to suffer from APS-1 due to *AIRE* deficiency (1, 2) and underwent genetic analysis to ascertain their diagnosis. All patients were found to harbor homozygous pathogenic variants in *AIRE*, which confirmed their clinical diagnosis (Fig. 1). A total of four different mutations were detected in the patients from Saudi Arabia, two of which are novel. These included c.93_94insT (p.L32SfsX3) in the HSR/CARD domain in Pt. 2 and c.199_202delCTGAinsTGG (p.L67WfsX80) in the L domain in Pts. 3–9, which are expected to result in frame shift and premature termination of protein 3 and 80 aa downstream of the mutation, respectively (Table 1 and Fig. 1). On the other hand, Pt. 1 carried c.44G>A (p.R15H), and Pts. 10–15 c.205_208dupCAGG (p.D70AfsX148), which have previously been described in APS-1 patients and associated with their disease (31, 32, 33, 34, 35, 36, 37). The Lebanese patients (Pts. 16–17) harbored c.1096-1G>A (E10del) that abolishes a splice acceptor site, resulting in deletion of exon 10, which encodes part of the PRR/L domains and has previously been reported in the compound heterozygous (38, 39) and homozygous forms (39). The Syrian patient (Pt. 18) harbored a novel pathogenic variant in *AIRE*, c.1311C>A (p.C437X) that truncates the protein within the PHD2 domain. None of the novel mutations were found in the healthy human databases gnomAD and 1000 Genomes Project.

**Table 1.** Demographic information, clinical manifestations, and *AIRE* mutations in newly identified APS-1 patients described in this study

Patient	Age of onset	Sex	Parents’ consanguinity	Clinical manifestations (Age)	Mutation (Domain)	Reference
Pt. 1	N/A	Female	Consanguineous (Second degree cousins)	- Recurrent oral candidiasis	c.44G>A/c.44G>A p.R15H/p.R15H (HSR/CARD)	Present report
				- Hypoparathyroidism		
				- Addison’s disease		
				- Alopecia areata		
				- Psoriasis		
				- Papillary thyroid cancer		
Pt. 2	3 years	Male	Consanguineous (First degree cousins)	- Mucocutaneous candidiasis (3Y)	c.93_94insT/c.93_94insT p.L32SfsX3/p.L32SfsX3 (HSR/CARD)	Present report
				- Hypoparathyroidism (5Y)		
				- Alopecia universalis (3Y)		
				- Hypothyroidism (5Y)		
				- Vitiligo (4Y)		
Pt. 3	10 years	Male	Consanguineous (First degree cousins)	- Recurrent oral candidiasis	c.199_202delCTGAinsTGG/c.199_202delCTGAinsTGG p.L67WfsX80/p.L67WfsX80 (L)	Present report
				- Hypoparathyroidism		
				- Addison’s disease		
				- Recurrent keratoconjunctivitis		
Pt. 4	6 years	Male	Consanguineous (First degree cousins)	- Hypoparathyroidism (6Y)	c.199_202delCTGAinsTGG/c.199_202delCTGAinsTGG p.L67WfsX80/p.L67WfsX80 (L)	Present report
				- Addison’s disease (7Y)		
				- Diabetes mellitus type 1 (8Y)		
				- Hypothyroidism (7Y)		
Pt. 5	4 years	Female	Consanguineous (First degree cousins)	- Candida onychomycosis	c.199_202delCTGAinsTGG/c.199_202delCTGAinsTGG p.L67WfsX80/p.L67WfsX80 (L)	Present report
				- Hypoparathyroidism		
				- Addison’s disease		
				- Diabetes mellitus type 1		
				- Primary ovarian failure		
				- Recurrent keratoconjunctivitis (7Y)		
Pt. 6	4 years	Male	Consanguineous (First degree cousins)	- Hypoparathyroidism	c.199_202delCTGAinsTGG/c.199_202delCTGAinsTGG p.L67WfsX80/p.L67WfsX80 (L)	Present report
				- Addison’s disease (7Y)		
				- Diabetes mellitus type 1 (8Y)		
				- Vitiligo		
Pt. 7	7 years	Male	Consanguineous (First degree cousins)	- Recurrent oral candidiasis	c.199_202delCTGAinsTGG/c.199_202delCTGAinsTGG p.L67WfsX80/p.L67WfsX80 (L)	Present report
				- Hypoparathyroidism		
				- Addison’s disease		
				- Alopecia areata		
				- Diabetes mellitus type 1		
				- Pernicious anemia		
Pt. 8	5 years	Male	Consanguineous (First degree cousins)	- Hypoparathyroidism (5Y)	c.199_202delCTGAinsTGG/c.199_202delCTGAinsTGG p.L67WfsX80/p.L67WfsX80 (L)	Present report
				- Addison’s disease (5Y)		
				- Alopecia universalis (10Y)		
				- Diabetes mellitus type 1 (7Y)		
				- Recurrent keratoconjunctivitis (7Y)		
Pt. 9	5 years	Male	Consanguineous (First degree cousins)	- Diabetes mellitus type 1 (5Y)	c.199_202delCTGAinsTGG/c.199_202delCTGAinsTGG p.L67WfsX80/p.L67WfsX80 (L)	Present report
Pt. 10	7 years	Male	Consanguineous (First degree cousins)	- Recurrent oral candidiasis (9Y)	c.205_208dupCAGG/c.205_208dupCAGG p.D70AfsX148/p.D70AfsX148 (HSR/CARD)	Present report
				- Hypoparathyroidism (7Y)		
				- Addison’s disease (8Y)		
Pt. 11	4 years	Female	Consanguineous (First degree cousins)	- Hypoparathyroidism (4Y)	c.205_208dupCAGG/c.205_208dupCAGG p.D70AfsX148/p.D70AfsX148 (HSR/CARD)	Present report
				- Alopecia areata (6Y)		
Pt. 12	2 years	Female	Consanguineous (First degree cousins)	- Recurrent oral candidiasis (2Y)	c.205_208dupCAGG/c.205_208dupCAGG p.D70AfsX148/p.D70AfsX148 (HSR/CARD)	Present report
				- Hypoparathyroidism (2Y)		
				- Addison’s disease (4Y)		
				- Perianal abscess (4Y)		
Pt. 13	3 years	Female	Consanguineous (Second degree cousins)	- Recurrent oral candidiasis (3Y)	c.205_208dupCAGG/c.205_208dupCAGG p.D70AfsX148/p.D70AfsX148 (HSR/CARD)	Present report
				- Hypoparathyroidism (4Y)		
				- Vitiligo (3Y)		
Pt. 14	N/A	Female	Consanguineous (Second degree cousins)	- Recurrent oral candidiasis	c.205_208dupCAGG/c.205_208dupCAGG p.D70AfsX148/p.D70AfsX148 (HSR/CARD)	Present report
				- Hypoparathyroidism		
				- Addison’s disease		
				- Primary ovarian failure		
Pt. 15	4 years	Female	Consanguineous (Second degree cousins)	- Recurrent oral candidiasis	c.205_208dupCAGG/c.205_208dupCAGG p.D70AfsX148/p.D70AfsX148 (HSR/CARD)	Present report
				- Hypoparathyroidism		
				- Addison’s disease		
				- Alopecia areata		
Pt. 16	1 year	Female	Consanguineous (Second degree cousins)	- Hypoparathyroidism (4Y)	c.1096-1G>A/c.1096-1G>A E10del/E10del (PRR, L)	Present report
				- Asthma		
				- Chronic cough		
				- Nephrocalcinosis		
				- Recurrent infections (1Y)		
Pt. 17	7 days	Male	Consanguineous (Second degree cousins)	- Hyperactive airways	c.1096-1G>A/c.1096-1G>A E10del/E10del (PRR, L)	Present report
				- Hyperinsulinemia hyperglycemia (7D)		
				- Diaper rash		
				- Recurrent infections (7W)		
				- Right trigger thumb		
Pt. 18	N/A	Female	Consanguineous (First degree cousins)	- Chronic mucocutaneous candidiasis	c.1311C>A/c.1311C>A p.C437X/p.C437X (PHD2)	Present report
				- Hypoparathyroidism (6Y)		
				- Atrophic glossitis		
				- Fingernail candidiasis		
				- Hepatitis (4Y)		
				- Nail dystrophy		
				- Perleche		
				- Tooth enamel hypoplasia		

Patient 1, family 1; patient 2, family 2; patients 3–9, family 3; patients 10 and 12, family 4; patient 11, family 5; patient13, family 6; patients 14–15, family 7; patients 16–17, family 8; patients 17, family 9.

Pt., patient; N/A, not available; dup, duplication; del, deletion; Ins, insertion; L, linker region.

The clinical management of the APS-1 patients involved a multidisciplinary approach targeting the diverse endocrine and non-endocrine manifestations of the disease. Hormone replacement therapy constituted the mainstay of treatment, including calcium and 1-α-hydroxyvitamin D3 supplementation for hypoparathyroidism, glucocorticoid and mineralocorticoid replacement for adrenal insufficiency, insulin therapy for type 1 diabetes mellitus, and thyroxine T4 hormone replacement for hypothyroidism. Chronic mucocutaneous candidiasis was treated with prolonged or intermittent antifungal therapy, most commonly azoles, although resistance and recurrence were observed in some patients. Given the risk of life-threatening adrenal crisis, patients and their families were educated on stress-dose steroid protocols and emergency management. Two of the pediatric patients (Pts. 2 and 8) developed alopecia universalis and were treated with Janus kinase (JAK; JAK1/JAK2) inhibitor at a dose of 10 mg twice daily. Both patients exhibited a marked clinical response with significant improvement of their alopecia and excellent tolerance with no reported clinical or laboratory adverse events upon follow-up for >12 mo while on therapy. This finding is consistent with previous studies that reported improvement of multiorgan autoimmunity in patients with APS-1 treated with ruxolitinib (40, 41). None of our patients were on immunosuppressive treatment when the immune workup was performed.

### Demographic and clinical information of all APS-1 patients found in the literature

Around 22% of our patients (4/18) did not fulfill the clinical criteria for a diagnosis of APS-1 (Pts. 9, 11, 16, and 17) and would have been missed if it were not for unbiased genetic sequencing. Therefore, we undertook a thorough review of the literature and quantified the clinical manifestations of all reported patients to identify additional clinical criteria that develop in nonclassical cases. A total of 116 manuscripts reporting the information of 883 patients were identified (Table S1). Only manuscripts that comprised patient information and genetic data that confirmed the diagnosis were included in the analysis.

**Figure 2.**
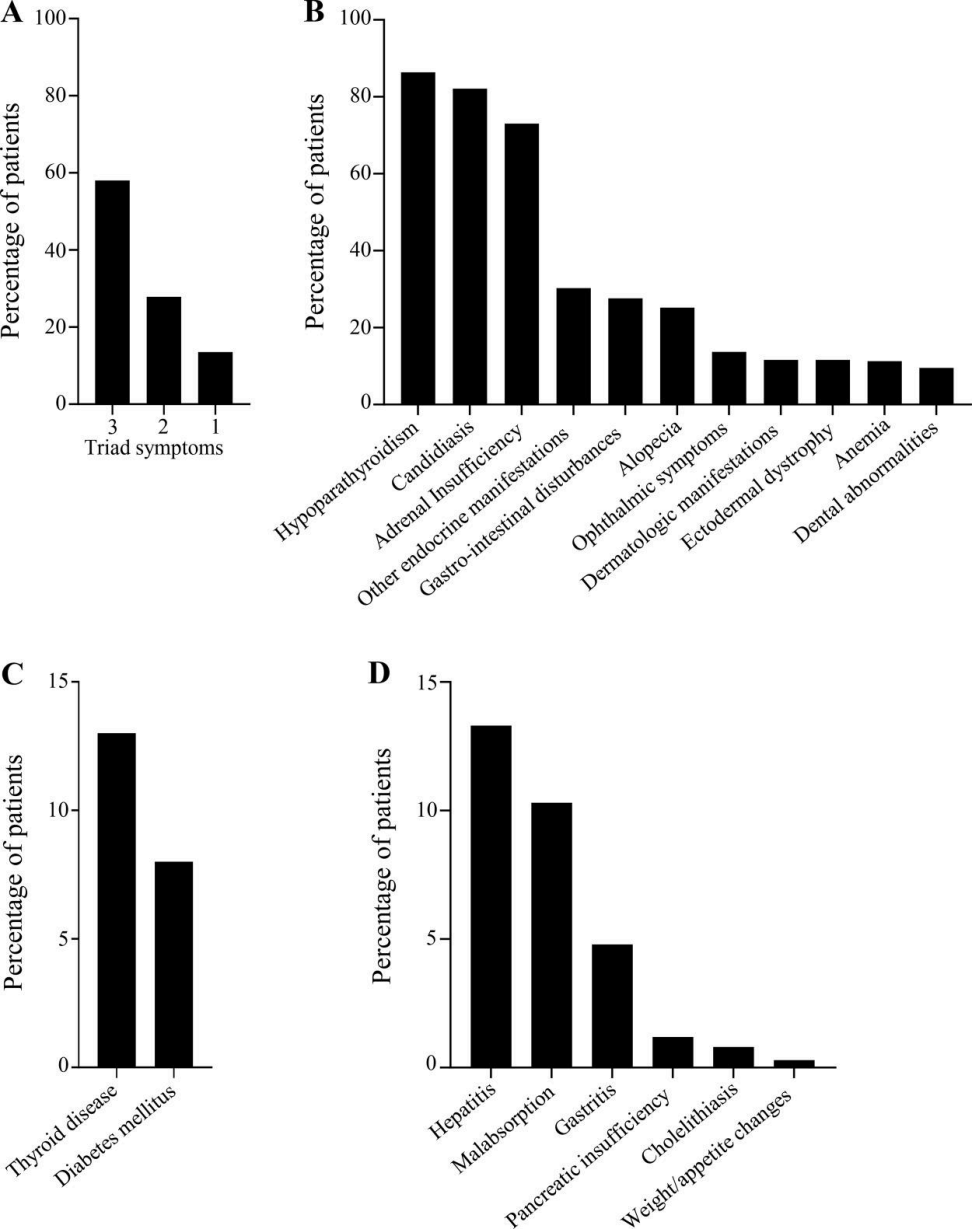
**Clinical manifestations of all published patients with APS-1 due to *AIRE* deficiency. (A)** Percentage of APS-1 patients presenting with one, two, or all three classical manifestations. **(B)** Most common clinical manifestations and their prevalence in APS-1 patients. **(C)** Most common “other endocrine manifestations” listed in B. **(D)** Stratification of the most common “gastrointestinal disturbances” listed in B.

The percentage of patients with consanguineous parents (first, second, and third degree) was 19.2% of 402 patients in whom consanguinity was reported (77/402). The female to male ratio was 1.17:1.00, and the median age of disease onset was 4.0 years with an interquartile range (IQR) of 2.0–7.0 years. A total of 677 patients had detailed clinical information included. Of those, 10 (1.5%) patients did not exhibit any of the classical symptoms of APS-1. Of the remaining 667 patients, 91 (13.6%) manifested only one of the three classical symptoms, 187 (28.0%) manifested two, and 389 (58.4%) manifested the three classical symptoms (Fig. 2 A). Hypoparathyroidism was the most frequent manifestation with a prevalence of 86.4% (576/667), candidiasis was found in 82.1% (547/667), and adrenal insufficiency was found in 73.0% (487/667) of the patients (Fig. 2 B). The fourth most frequent manifestation included endocrine problems other than hypoparathyroidism and adrenal insufficiency, found in 30.3% (205/677) of the patients, followed by gastrointestinal disturbances in 27.6% (187/677) and alopecia in 25.2% (170/677) of the patients (Fig. 2 B). Other symptoms included ophthalmic (13.7%) and dermatologic disorders (11.6%), ectodermal dystrophy (11.6%), anemia (11.3%), and dental abnormalities (9.5%).

Of the “other” endocrine problems identified, thyroid disease and diabetes mellitus were the most common and found in 13% (88/677) and 8% (54/677) of all patients, respectively (Fig. 2 C). Furthermore, the most common gastrointestinal disturbances were further stratified into hepatitis that was found in 13.3% (90/677) of the patients, malabsorption in 10.3% (70/677), gastritis in 4.8% (32/677), pancreatic insufficiency in 1.2%, cholelithiasis in 0.8%, and weight/appetite change in 0.3% of the patients (Fig. 2 D). The percentages of all clinical symptoms, including additional rare manifestations, are detailed in Table S2.

### Genetic information of all APS-1 patients found in the literature

Genetic information was available on 861 of the 883 (97.5%) patients reported in the literature, of which 505 (58.6%) patients harbored homozygous, and 371 (43.1%) harbored compound heterozygous or heterozygous variants. The genetic diagnosis was made but unreported for 21 patients; therefore, they were not included in the analysis (Table S1).

The most common homozygous pathogenic variants included the c.769C>T (p.R257X) found in 44.8% of patients with homozygous mutations, followed by the c.967_979del13bp (p.L323fs) in 22.9%, c.232T>C (p.W78R) in 5.2%, and c.254A>G (p.Y85C) and c.415C>T (p.R139X) each found in 4% of the patients (Table 2). All homozygous mutations with their prevalence are further detailed in Table S3.

**Table 2.** List of the most common homozygous *AIRE* mutations in APS-1 patients reported in the literature

Mutation	Protein (Affected domain)	Prevalence
c.769C>T	p.R257X (SAND)	44.8%
c.967_979del13bp	p.L323fs (PHD1)	22.9%
c.232T>C	p.W78R (HSR/CARD)	5.2%
c.254A>G	p.Y85C (HSR/CARD)	4.0%
c.415C>T	p.R139X (Downstream of NLS)	4.0%
c.205_208dupCAGG	p.D70AfsX148 (HSR/CARD)	2.0%
c.1616C>T	p.P539L (TAD)	1.4%
c.199_202delCTGinsTGG	p.L67WfsX80 (L)	1.2%
c.1193delC	p.P398fs (PRR)	1.2%
c.44G>A	p.R15H (HSR/CARD)	1.0%
c.47C>T	p.T16M (HSR/CARD)	1.0%
c.607C>T	p.R203X (SAND)	1.0%

dup, duplication; del, deletion; Ins, insertion; bp, base pair; HSR, homogeneously staining region; CARD, caspase activation and recruitment domain; L, linker region; SAND, Sp100, AIRE-1, NucP41/75, DEAF-1 domain; PHD, plant homeodomain; PRR, proline-rich region; TAD, transactivation domain; NLS, nuclear localization signal.

Among the heterozygous pathogenic variants, the c.769C>T (p.R257X) and c.967_979del13bp (p.L323fs) were also the most common with a prevalence of 52.5 and 28.6%, respectively, among all patients with heterozygous mutations (Table 3). This is followed by c.232T>C (p.W78R) in 9.1%, c.47C>T (p.T16M) in 6.2%, and c.62C>T (p.A21V) found in 4.7% of the patients. The list of heterozygous mutations found in 1–3.5% of the patients is detailed in Table 3, and all heterozygous mutations with their prevalence are listed in Table S4. Furthermore, all compound heterozygous mutations identified in APS-1 patients and associated with their disease, heterozygous mutations that have been biologically or experimentally demonstrated to act in an autosomal dominant or recessive manner, as well as heterozygous variants of uncertain significance are listed in Table S5.

**Table 3.** List of the most common heterozygous *AIRE* mutations in APS-1 patients reported in the literature

Mutation	Protein (Affected domain)	Prevalence
c.769C>T	p.R257X (SAND)	52.5%
c.967_979del13bp	p.L323fs (PHD1)	28.6%
c.232T>C	p.W78R (HSR/CARD)	9.1%
c.47C>T	p.T16M (HSR/CARD)	6.2%
c.62C>T	p.A21V (HSR/CARD)	4.7%
c.932G>A	p.C311Y (PHD1)	3.5%
c.607C>T	p.R203X (SAND)	2.9%
c.1072C>T	p.Q358X (PRR)	2.9%
c.977C>T	p.P326L (PHD1)	2.4%
c.1616C>T	p.P539L (TAD)	1.8%
c.1249dupC	p.L417fs (L)	1.8%
c.1163_1164insA	p.M388fs (PRR)	1.8%
c.1242_1243insA	p.H415fs (L)	1.8%
c.415C>T	p.R139X (Downstream of NLS)	1.5%
c.274C>T	p.R92W (HSR/CARD)	1.5%
c.260T>C	p.L87P (HSR/CARD)	1.5%
c.755C>T	p.P252L (SAND)	1.5%
c.21_43dup23	p.R15fs (HSR/CARD)	1.2%
c.1249delC	p.L417fs (L)	1.2%
c.1344delC	p.C449fs (PHD2)	1.2%
c.1638A>T	p.X546C+59aa	1.2%

dup, duplication; del, deletion; Ins, insertion; bp, base pair; WT, wilde type; HSR, homogeneously staining region; CARD, caspase activation and recruitment domain; L, linker region; SAND, Sp100, AIRE-1, NucP41/75, DEAF-1 domain; PHD, plant homeodomain; PRR, proline-rich region; TAD, transactivation domain; NLS, nuclear localization signal.

### Immunophenotype and T cell function of APS-1 patients identified in this study

**Figure 3.**
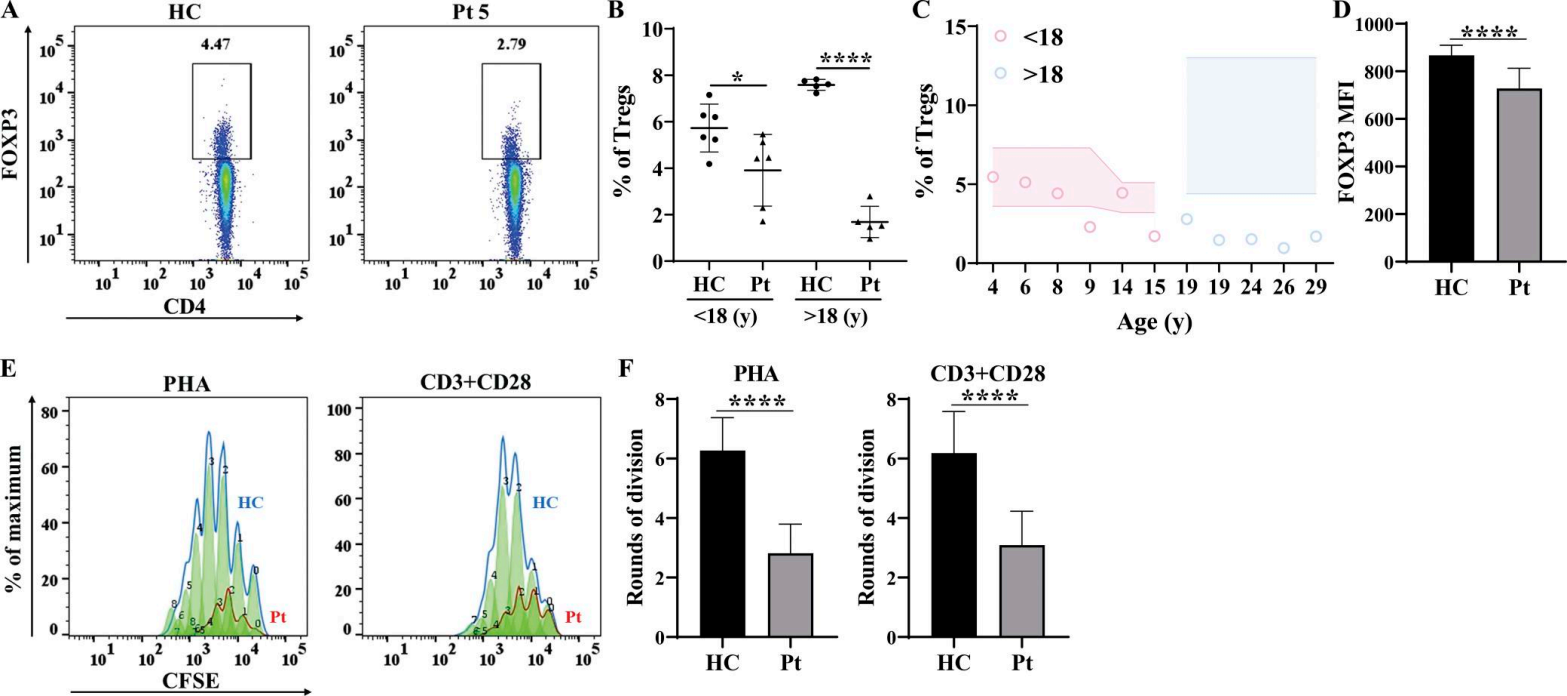
**Diminished Treg percentages and reduced rounds of T cell proliferation in the patients. (A)** Representative Treg (CD4^+^FOXP3^+^) staining in Pt. 5 and a healthy control. **(B and C)** Quantification of the percentages of Tregs in the patients as compared to age-matched controls (B) and the reference range for age represented by the shaded area (C). **(D)** FOXP3 expression levels determined by the mean fluorescent intensity of FOXP3 staining in the CD4^+^FOXP3^+^ double-positive population. **(E and F)** Representative T cell proliferation (E) and quantification of the rounds of cell division (F) upon stimulation with PHA and anti-CD3 + anti-CD28 antibodies. Pt., patient; HC, healthy control; MFI, mean fluorescent intensity. *P < 0.05; ****P < 0.0001.

APS-1 is a disease of T cell dysregulation, yet the detailed nature of the patients’ peripheral lymphocytes, especially T cells, is not broadly studied. Therefore, we undertook an immune workup in 11 of our patients to assess the presence of immune defects associated with the disease. Six patients were below the age of 18 years, and five between 19 and 29 years of age (Table 4). The patients’ white blood cell, lymphocyte, CD3^+^ T cell, and CD4^+^ helper T cell numbers were within the range for age or slightly higher for some patients. On the other hand, the numbers of CD8^+^ cytotoxic T cells were generally normal or higher than their range for age for most of the patients, with the exception of Pts. 10 and 16 who had low CD8^+^ T cells. The number of naive CD4^+^ and CD8^+^ T cells were within the normal range for age or higher for all patients. Central memory CD4^+^ and CD8^+^ T cells were within the range and on the lower end of the range for age, respectively, whereas effector memory T cells were decreased in both populations. The recent thymic emigrant CD4^+^ T cells were within the range for age or slightly higher in the patients. On the other hand, both the absolute numbers and percentages of Tregs were significantly reduced in pediatric/adolescent (<18 years) and adult (>18 years) patients compared to age-matched controls (Fig. 3, A and B; and Table 4). This reduction in Treg percentages was more pronounced in the adult population and was evident when patients values were compared to the broader age-specific reference ranges for each group (Fig. 3 C). In addition, FOXP3 expression levels were significantly decreased in patients Tregs compared to controls (Fig. 3 D). The levels of B cell subpopulations and natural killer (NK) cells were within the normal range for age or inconsistently fluctuating around it. Furthermore, the capacity of the patients’ T cells to proliferate was reduced when compared to controls, as evidenced by significantly fewer rounds of cell division following stimulation with phytohemagglutinin (PHA) (2.91 ± 0.31 in the patients vs. 6.23 ± 0.816 in the controls) and anti-CD3 + anti-CD28 (3.09 ± 0.34 in the patients vs. 6.14 ± 0.64 in the controls) (Fig. 3, E and F; and Table 4). The different rounds of T cell division were calculated using the Proliferation Modeling tool in FlowJo software, and the differences were quantified using GraphPad PRISM.

**Table 4.** Laboratory investigation of 11 APS-1 patients reported in this manuscript

​	P17 4 years M	P12 6 years F	P2 8 years M	P10 9 years M	P16 14 years F	P8 15 years F	P5 19 years F	P6 19 years M	P9 24 years M	P7 26 years M	P3 29 years M
White blood cells/μl	8,700 (5,200–11,000)	5,440 (4,400–9,500)	4,000 (4,400–9,500)	6,030 (4,400–9,500)	6,400 (4,400–8,100)	7,110 (4,400–8,100)	**12,000** (2,600–11,800)	4,720 (2,600–11,800)	7,320 (2,600–11,800)	**13,100** (2,600–11,800)	**13,300** (2,600–11,800)
Lymphocytes (cells/μl)	4,933 (2,300–5,400)	**3,860** (1,900–3,700)	2,390 (1,900–3,700)	2,740 (1,900–3,700)	1,414 (1,400–3,300)	1,570 (1,400–3,300)	**3,450** (1,154–2,223)	1,322 (1,154–2,223)	**2,360** (1,154–2,223)	2,160 (1,154–2,223)	**2,950** (1,154–2,223)
Lymphocytes %	**56.7** (32.3–50.4)	**70.8** (29.5–43.3)	**59.8** (29.5–43.3)	**45.4** (29.5–43.3)	**54.3** (29.8–39.6)	**22.1** (29.8–39.6)	28.8 (25.5–39.4)	28.0 (25.5–39.4)	32.3 (25.5–39.4)	**16.4** (29.8–45.6)	**22.2** (29.8–45.6)
CD3^+^ T cells/μl	3,631 (1,400–3,700)	**2,941** (1,200–2,600)	1,687 (1,200–2,600)	1,877 (1,200–2,600)	1,122 (1,000–2,200)	1,350 (1,000–2,200)	**2,332** (735–1,581)	1,122 (735–1,581)	**1,610** (735–1,581)	1,460 (735–1,581)	**1,714** (735–1,581)
CD3^+^ T cells % of lymphocytes	73.6 (56–75)	76.2 (60–76)	70.6 (60–76)	68.5 (60–76)	79.3 (56–84)	**86.0** (56–84)	67.6 (55–79)	**85.0** (55–79)	68.2 (55–79)	67.6 (55–79)	58.1 (55–79)
CD3^+^CD4^+^ cells/μl	1,935 (700–2,200)	**2,255** (650–1,500)	1,134 (650–1,500)	1,490 (650–1,500)	759 (530–1,300)	921 (530–1,300)	**1,586** (362–909)	858 (362–909)	858 (362–909)	901 (362–909)	427 (362–909)
CD3^+^CD4^+^ cells (% of CD3^+^)	**53.3** (28–47)	**76.7** (31–47)	**67.2** (31–47)	**79.4** (31–47)	**67.7** (31–52)	**68.2** (31–52)	**68.0** (35–65)	**76.5** (35–65)	53.3 (35–65)	61.7 (35–65)	**24.9** (35–65)
Naive CD4^+^ T cells/μl	**1,695** (500–1,600)	**1,871** (200–1,200)	838 (200–1,000)	**1,238** (100–700)	636 (300–700)	**718** (300–700)	**1,175** (300–700)	**716** (300–700)	642 (300–700)	553 (300–700)	**38** (300–700)
Naive CD4^+^ T cells (% of CD4^+^)	**87.6** (46–72)	**83.0** (35–69)	**73.9** (32–68)	**83.1** (31–57)	**83.8** (31–57)	**78** (31–57)	**74.1** (31–57)	**83.4** (31–57)	**74.9** (31–57)	**61.4** (31–57)	**8.9** (31–57)
Central memory CD4^+^ T cells/μl	191 (100–400)	126 (100–300)	142 1(00–300)	**80** (100–300)	106 (100–300)	136 (100–300)	254 (100–300)	**97** (100–300)	111 (100–300)	196 (100–300)	211 (100–300)
Central memory CD4^+^ T cells (% of CD4^+^)	**9.87** (11–20)	**5.6** (9–25)	12.5 (9–24)	**5.4** (11–25)	13.9 (10–27)	14.8 (10–27)	16 (10–27)	11.3 (10–27)	13.0 (10–27)	21.8 (10–27)	**49.4** (10–27)
Effector memory CD4^+^ T cells/μl	**32** (100–400)	145 (100–300)	128 (100–400)	110 (100–300)	**11** (100–600)	**48** (100–600)	124 (100–600)	**34** (100–600)	**82** (100–600)	138 (100–600)	148 (100–600)
Effector memory CD4^+^ T cells (% of CD4^+^)	**1.63** (9–20)	**6.4** (10–30)	11.3 (9–32)	**7.4** (12–30)	**1.44** (12–44)	**5.2** (12–44)	**7.8** (12–44)	**4.0** (12–44)	**9.5** (12–44)	15.3 (12–44)	34.7 (12–44)
Effector memory T_EMRA_ CD4^+^ T cells/μl	16 (0–300)	111 (0–300)	26 (0–200)	61 (0–200)	6 (0–100)	32 (0–100)	32 (0–100)	10 (0–100)	22 (0–100)	13 (0–100)	29 (0–100)
Effector memory T_EMRA_ CD4^+^ (% of CD4^+^)^+^	**0.86** (3–14)	4.9 (4–22)	**2.3** (4–15)	4.1 (4–24)	**0.84** (4–12)	**2.1** (4–12)	**2.1** (4–12)	**1.2** (4–12)	**2.6** (4–12)	**1.4** (4–12)	**6.9** (4–12)
CD3^+^CD8^+^ cells/μl	**1,401** (490–1,300)	406 (370–1,100)	410 (370–1,100)	**170** (370–1,100)	**277** (330–920)	377 (330–920)	457 (225–669)	236 (225–669)	391 (225–669)	504 (225–669)	**1,107** (225–669)
CD3^+^CD8^+^ cells % of CD3^+^	**38.6** (16–30)	**13.8** (18–35)	24.3 (18–35)	**9.04** (18–35)	24.7 (18–35)	27.9 (18–35)	**19.6** (25–54)	**21.0** (25–54)	**24.3** (25–54)	34.5 (25–54)	**64.6** (25–54)
Naive CD8^+^ T cells/μl	**1,251** (200–600)	298 (100–600)	289 (100–600)	105 (100–400)	227 (100–400)	185 (100–400)	296 (100–400)	186 (100–400)	209 (100–400)	140 (100–400)	102 (100–400)
Naive CD8^+^ T cells (% of CD8^+^)	**89.3** (29–72)	**73.4** (23–68)	**70.4** (30–61)	**61.7** (22–58)	**81.8** (18–61)	49.2 (18–61)	**64.7** (18–61)	**79.0** (18–61)	53.5 (18–61)	27.9 (18–61)	**9.17** (18–61)
Central memory CD8^+^ T cells/μl	15 (0–200)	5 (0–100)	42 (0–100)	0 (0–100)	3 (0–100)	32 (0–100)	30 (0–100)	11 (0–100)	12 (0–100)	35 (0–100)	**166** (0–100)
Central memory CD8^+^ T cells (% of CD8^+^)	**1.06** (2–13)	**1.25** (4–11)	10.2 (4–11)	**0.26** (2–15)	**0.89** (3–12)	8.37 (3–12)	6.55 (3–12)	4.73 (3–12)	**2.96** (3–12)	6.92 (3–12)	**15.0** (3–12)
Effector memory CD8^+^ T cells/μl	**22** (100–400)	**31** (100–500)	**7** (100–500)	**25** (100–400)	**4** (100–600)	**44** (100–600)	**81** (100–600)	**32** (100–600)	105 (100–600)	119 (100–600)	537 (100–600)
Effector memory CD8^+^ T cells (% of CD8^+^)	**1.61** (14–49)	**7.75** (14–59)	**1.68** (14–59)	**14.9** (24–58)	**1.61** (25–58)	**11.8** (25–58)	**17.7** (25–58)	**13.6** (25–58)	26.9 (25–58)	**23.7** (25–58)	48.5 (25–58)
Effector memory T_EMRA_ CD8^+^ T cells/μl	112 (0–300)	71 (0–300)	72 (0–300)	39 (0–200)	43 (0–200)	115 (0–200)	50 (0–200)	6 (0–200)	65 (0–200)	**210** (0–200)	**303** (0–200)
Effector memory T_EMRA_ CD8^+^ (% of CD8^+^)^+^	8.01 (5–25)	17.6 (6–30)	17.7 (6–30)	23.2 (7–26)	15.7 (5–20)	**30.6** (5–20)	11.0 (5–20)	**2.69** (5–20)	16.6 (5–20)	**41.5** (5–20)	**27.4** (5–20)
Recent thymic emigrant T cells/μl	1,144 (600–1,600)	**1,643** (300–1,300)	768 (300–1,300)	**997** (300–800)	486 (300–700)	507 (300–700)	**902** (42–399)	**444** (42–399)	364 (42–399)	**419** (42–399)	174 (42–399)
Recent thymic emigrant (% of CD4^+^)	59.1 (55–70)	**72.9** (52–71)	67.7 (52–71)	66.9 (43–67)	**64** (37–62)	55.1 (37–62)	**56.9** (6.4–51)	**51.7** (6.4–51)	42.4 (6.4–51)	46.5 (6.4–51)	40.7 (6.4–51)
Tregss/μl	**106** (2–80)	**115** (2–80)	50 (2–80)	34 (2–80)	34 (1–30)	16 (1–30)	44 (13–66)	13 (13–66)	13 (13–66)	**9** (13–66)	**7** (13–66)
Tregs (% of CD4^+^)	5.46 (3.6–7.3)	5.12 (3.6–7.3)	4.42 (3.6–7.3)	**2.29** (3.6–7.3)	4.45 (3.2–5.1)	**1.71** (3.2–5.1)	**2.79** (4.4–13.0)	**1.47** (4.4–13.0)	**1.52** (4.4–13.0)	**0.96** (4.4–13.0)	**1.69** (4.4–13.0)
CD19^+^ B cells/μl	518 (402–784)	**676** (228–516)	**197** (228–516)	381 (228–516)	**101** (226–370)	**138** (226–370)	**100** (133–255)	**29** (133–255)	**328** (133–255)	**115** (169–271)	197 (169–271)
CD19^+^ B cells % of lymphocytes	10.5 (13.4–21.1)	17.5 (9.8–17.7)	**8.25** (9.8–17.7)	13.9 (9.8–17.7)	7.17 (10.2–15.4)	**8.79** (10.2–15.4)	**2.89** (6.6–10.8)	**2.20** (6.6–10.8)	**13.9** (6.6–10.8)	**5.34** (7.2–11.2)	**6.69** (7.2–11.2)
Naive B cells/μl	349 (334–611)	**507** (133–389)	175 (133–389)	290 (133–389)	**64** (171–293)	**101** (171–293)	**62** (92–199)	**16** (92–199)	**285** (92–199)	**43** (112–169)	**85** (112–169)
Naive B cells (% of CD19^+^)	**67.3** (76.3–84.9)	75.1 (69.4–80.4)	**88.9** (69.4–80.4)	76.3 (69.4–80.4)	**63.1** (75.2–86.7)	**73.3** (75.2–86.7)	**62.3** (65.6–79.6)	**54.3** (65.6–79.6)	**87.0** (65.6–79.6)	**37.5** (58.0–72.1)	**43.2** (58.0–72.1)
Memory unswitched B cells/μl	**74** (25–60)	**3** (22–43)	**9** (22–43)	**14** (22–43)	**9** (12–32)	**11** (12–32)	23 (12–34)	**9** (12–34)	16 (12–34)	42 (22–54)	23 (22–54)
Memory unswitched B cells (% of CD19^+^)	**14.3** (4.1–9.0)	**5.38** (7.5–12.4)	**4.62** (7.5–12.4)	**3.59** (7.5–12.4)	9.26 (4.6–10.2)	8.32 (4.6–10.2)	**22.9** (7.4–13.9)	**31.1** (7.4–13.9)	**4.95** (7.4–13.9)	**36.1** (13.4–21.4)	**11.6** (13.4–21.4)
Memory switched B cells/μl	**82** (16–44)	**103** (16–31)	**10** (16–31)	**37** (16–31)	20 (10–29)	23 (10–29)	11 (10–31)	**4** (10–310)	22 (10–31)	26 (18–40)	**64** (18–40)
Memory switched B cells (% of CD19^+^)	15.9 (3.3–7.4)	15.2 (5.2–12.1)	5.19 (5.2–12.1)	9.74 (5.2–12.1)	19.9 (3.3–9.6)	16.7 (3.3–9.6)	11.3 (7.2–12.7)	12.7 (7.2–12.7)	6.59 (7.2–12.7)	22.9 (9.2–18.9)	32.6 (9.2–18.9)
NK cells/μl	155 (130–720)	**29** (100–480)	137 (100–480)	**59** (100–480)	**13** (70–480)	70 (70–480)	552 (90–600)	97 (90–600)	345 (90–600)	422 (90–600)	460 (90–600)
NK cells (% of CD3^−^)	11.9 (4–17)	**3.16** (4–17)	**19.5** (4–17)	6.86 (4–17)	4.56 (3–22)	**31.7** (3–22)	**49.4** (7–31)	**49.2** (7–31)	**46** (7–31)	**60.3** (7–31)	**37.2** (7–31)
**T cell proliferation**	​	​	​	​	​	​	​	​	​	​	​
Rounds of cell division with α-CD3/CD28 stimulation Controls = 6.14 ± 0.64, (*n* = 22) Patients = 3.09 ± 0.34, (*n* = 11) ****P < 0.0001	**4**	**3**	**3**	**3**	**4**	**3**	**3**	**3**	**4**	**4**	**0**
Rounds of cell division with PHA stimulation Controls = 6.23 ± 0.81, (*n* = 22) Patients = 2.91 ± 0.31, (*n* = 11) ****P < 0.0001	**3**	**4**	**3**	**3**	**3**	**4**	**3**	**3**	**3**	**3**	**0**

Naïve T cells, CD45RA^+^CCR7^+^; central memory T cells, CD45RA^−^CCR7^+^; effector memory T cells, CD45RA^−^CCR7^−^; exhausted effector memory T_EMRA_ T cells, CD45RA^+^CCR7^−^; recent thymic emigrant T cells, CD4^+^CD45RA^+^CD31^+^; Tregs, CD4^+^FOXP3^+^; naïve B cells, CD19^+^CD27^−^IgD^+^; memory unswitched B cells, CD19^+^CD27^+^IgD^+^; memory switched B cells CD19^+^CD27^+^IgD^−^; NK cells, CD3^−^CD16^+^CD56^+^.

Ranges for age put under each value are from (42, 43, 44, 45, 46, 47) and our own clinical laboratory. Bolded are the values that fall outside the range for age. Shown are the average ± SD for T cell proliferation with the P values.

## Discussion

In this manuscript, we present the clinical and genetic information of 18 new patients with APS-1 from Saudi Arabia, Lebanon, and Syria. In addition, we extracted the clinical and genetic data of confirmed patients with APS-1 reported in the literature and identified alopecia, thyroid disease, diabetes mellitus, hepatitis, or malabsorption as additional common manifestations of APS-1. We also identified a mutational hotspot that accounts for >80% of all mutations found in the patients. Furthermore, we identified reduced Treg frequencies and impaired T cell proliferation as two abnormal laboratory parameters in our patients.

The diagnosis of APS-1 is typically made when a patient presents with at least two of the three hallmark features (27). In our cohort of 18 patients, 2 (11%) did not manifest any of the hallmark features (Pts. 9 and 17), and 2 patients had only one manifestation (Pts. 11 and 16) and would have been missed if it were not for unbiased genetic sequencing. Therefore, we considered identifying other common but less frequent manifestations. To achieve this goal, the limited number of patients newly diagnosed in this work was not enough; therefore, we undertook a thorough review of the literature to determine the clinical manifestations of all patients reported (Table S1). This revealed that alopecia was present in >25% of all the patients, which is in accordance with other studies (48), and suggests that it represents an additional manifestation of the disease to consider, especially that it is easily detectable. Other symptoms included a group of endocrine disturbances, of which thyroid disease and diabetes mellitus are the most common. Diabetes mellitus in Pt. 9 and alopecia with hypoparathyroidism in Pt. 11 were the only presenting features in these individuals. These cases would likely have been overlooked if not for a suggestive family history that prompted genetic testing, emphasizing the utility of these extended phenotypic criteria to recognize APS-1. Autoimmune hepatitis and malabsorption are frequent among the gastrointestinal disturbances and could also be supportive of the disease. The frequency of these additional manifestations might be underestimated as some studies only looked for the classical symptoms (49). While they might not be highly specific on their own, we believe that alopecia, thyroid disease, diabetes mellitus, hepatitis, or malabsorption could be strongly indicative of APS-1, especially in the presence of one of the traditional classical features. Our results are in accordance with a previous systematic review of the literature that demonstrated that hypoparathyroidism, adrenal insufficiency, and mucocutaneous candidiasis were the most frequent and earliest to manifest in the majority of APS-1 patients and recognized that ectodermal dystrophy (alopecia found in 28.6%) and non-triad endocrine manifestations (thyroid dysfunction in 14.1% and diabetes mellitus in 10.5%) were also common (48).

Genetic analysis of our cohort identified three previously unpublished homozygous deleterious pathogenic variants in *AIRE*. Two of these variants, c.93_94insT and c.199_202delCTGAinsTGG, were identified in Pt. 2 and Pts. 3–9 from Saudi Arabia, respectively, while the third variant, c.1311C>A, was detected in Pt. 18 from Syria. Although these variants had been independently submitted to ClinVar by clinical testing laboratories, they are reported here for the first time in association with APS-1. Having screened the literature of patients with APS-1, we also identified the most common mutational spectrum of *AIRE* in APS-1. This analysis revealed that the five most frequent homozygous pathogenic variants, c.769C>T, c.967_979del13bp, c.232T>C, c.254A>G, and c.415C>T, account for over 80% of all homozygous mutations reported.

The most commonly reported heterozygous variants include c.769C>T, c.967_979del13bp, c.232T>C, c.47C>T, and c.62C>T. Compound heterozygous mutations represent the most common form of heterozygosity among APS-1 patients reported in the literature (Tables S1 and S5), consistent with the autosomal recessive inheritance of the disease. However, a subset of patients harbors heterozygous autosomal dominant mutations in the SAND or PHD-1 domains of AIRE. These variants impair AIRE oligomerization, DNA binding, and transcriptional activity and are typically associated with later onset, milder clinical phenotypes, and incomplete penetrance (5, 50, 51, 52, 53, 54, 55, 56, 57, 58, 59). Another subset of patients with mild manifestations carry heterozygous AIRE variants that were biologically or experimentally demonstrated to be recessive or that have not been functionally characterized and are, hence, of uncertain significance (Tables S1 and S5). This raises the possibility that the identified variant may exert a dominant-negative effect or that the second allele harbored a noncoding pathogenic variant, such as a mutation in the promoter, enhancer, or intronic region, that disrupts RNA expression or splicing. Definitive assessment of such regulatory or cryptic variants is challenging, in part because *AIRE* mRNA is difficult to reliably amplify from PBMC, as observed in our own experiments, despite some contradictory reports in the literature (60).

When screening for *AIRE* variants in patients presenting with one component of the classic triad, or with combinations of at least two rarer APS-1 manifestations, NGS remains the fastest approach to achieve a diagnosis. However, our findings underscore the utility of a targeted *AIRE* Sanger sequencing strategy focused on mutational hotspots. In particular, emphasis should be placed on the c.769C>T (p.R257X) and c.967_979del13bp (p.L323fs) variants, which are the most prevalent worldwide, especially in Europe and North America, as well as on the c.232T>C (p.W78R) variant in the Apulia region of Italy, the c.415C>T (p.R139X) variant in Sardinia, and the c.254A>G (p.Y85C) variant in Persian Jewish populations (12, 48, 61, 62). In patients from the Middle East, priority could be given to the c.93_94insT (p.L32SfsX3), c.199_202delCTGAinsTGG (p.L67WfsX80), c.205_208dupCAGG (p.D70AfsX148), c.1096-1G>A (E10del), and c.1311C>A (p.C437X) variants identified in this study. Such a targeted approach may represent a cost-effective and rapid diagnostic alternative to whole-exome sequencing (WES), particularly in resource-limited settings.

During the normal process of T cell development, T cells with TCRs that fail to recognize antigen:MHC complexes die by neglect (63), while those with TCRs that recognize antigens strongly are negatively selected, and those that recognize antigens moderately develop into Tregs (64). We investigated the numbers and nature of peripheral lymphocytes in AIRE-deficient patient with special focus on the T cell compartment. We detected normal numbers of recent thymic emigrants and peripheral T cells in the patients, as well as normal naive/memory T cell distribution. However, the quality of peripheral T cell function, assayed by their ability to proliferate following stimulation through their TCR, was decreased in the patients. The proliferation of T cells isolated from APS-1 patients has previously been reported to be normal (65). The discrepancy between our work and the previous work could be due to the use of tritiated-thymidine incorporation previously as compared to the dye dilution method in our case, which captures the different rounds of cell division over the entire stimulation period rather than incorporation of tritiated-thymidine in the last 6–16 h of the assay. Although diminished, the ability of the T cells to proliferate is not completely abolished in our patients, suggesting that AIRE might play a minor role in peripheral T cell function or homeostasis. Alternatively, AIRE might play a role in the optimal developmental program of thymocytes, as it has been detected at low levels in double-positive T cells (66). These results also suggest that the functional T cell defect could contribute to the increased risk of infections in the patients.

The percentages of Tregs were decreased in our patients, with lower levels of FOXP3 expression. This decrease was particularly pronounced in the adult population and might be consistent with the progressive nature of autoimmunity. Our results are in concordance with previous studies that showed that the percentages of Tregs, especially the resting population, and their levels of FOXP3 were diminished in APS-1 patients (65, 67, 68, 69). In addition, freshly isolated Tregs isolated from AIRE-deficient patients were unable to inhibit T effector cell proliferation (65), however, stimulation of AIRE-deficient Tregs *ex vivo* resulted in normal expansion, activation, and restoration of their suppressive activity (70). Therefore, in the absence of AIRE, death by neglect of high affinity TRA-specific autoreactive T effector cells in the thymus, combined with partially functional Tregs, might contribute to the relatively mild autoimmunity observed in APS-1 patients. Although reduced T cell function and diminished Treg numbers are not specific to AIRE deficiency only, they may provide supportive evidence for an APS-1 diagnosis when considered alongside the broader clinical phenotype.

In conclusion, we report 18 newly diagnosed APS-1 patients from the Middle East, including three previously unpublished pathogenic variants in *AIRE*. Additionally, through a comprehensive literature review encompassing, to our knowledge, all genetically confirmed APS-1 cases reported to date, we identified additional clinical manifestations, the most frequent of which is alopecia, that may aid in diagnosing patients who fall outside the classical diagnostic criteria. We also highlight mutational hotspots in *AIRE* that could streamline genetic testing, and laboratory-based immunological markers that further support the diagnosis.

## Materials and methods

### Patients

18 patients with APS-1 are included in this study, 15 of whom are from Saudi Arabia (Pts. 1–15), 2 from Lebanon (Pts. 16 and 17), and 1 from Syria (Pt. 18). Their medical history and clinical symptoms were collected by searching the electronic medical record systems. Peripheral blood was obtained by venipuncture. All studies were performed after securing informed consent approved by the Institutional Review Board of the American University of Beirut Medical Center (Protocol number BIO-2018-0358), King Abdullah International Medical Research Center (Protocol number 0113/24), and according to the Declaration of Helsinki.

### Search and data extraction

All reported genetic variants in *AIRE* were identified in ClinVar. Variants classified as pathogenic or likely pathogenic were investigated through a comprehensive literature review conducted up to June 2025, using PubMed, Medline, Web of Science, and Scopus. Only published patients with a documented pathogenic genetic diagnosis identified through the literature review were included in Table S1. Variants deemed pathogenic in ClinVar based on clinical genetic reports, without documented cases in humans, were excluded. The search was restricted to articles published in English or French. Search terms included all reported nomenclature variants in cDNA, genomic DNA (gDNA), and protein, applied across keywords, titles, and abstracts. Reference lists of all relevant articles were manually screened to identify additional studies, some of which included genetic variants not reported in ClinVar, and were therefore added to Table S1. When patient cases appeared in multiple publications, duplicates were excluded to ensure data accuracy. Extracted data included clinical manifestations, genotype, sex, age of onset of symptoms, and the degree of parental consanguinity.

### Genetic analysis

Clinical WES was performed commercially for Pts. 1–15. A targeted gene panel (TGP) approach was used for Pts. 16 and 18. In Brief, gDNA was extracted from blood using the Gentra Puregene Blood Kit (Qiagen). Custom-made primers were designed and ordered using the Ion AmpliSeq Designer (Thermo Fisher Scientific) and used to amplify a TGP of 300 genes, resulting in 6,203 amplicons of exonic and intronic sequences. The products were subjected to NGS on an Ion Torrent GeneStudio S5 System (Thermo Fisher Scientific), and the results were analyzed on the Ion Reporter software (Thermo Fisher Scientific) using customized filters. Since Pt. 17 is the sibling of Pt. 16, the variant detected using the TGP in Pt. 16 was identified in Pt. 17 by polymerase chain reaction (PCR) with TopTaq DNA Polymerase (Qiagen) using forward primer 5′-GAT​AAC​GGC​CCC​GGA​AGA​TG-3′ and reverse primer 5′-CTC​AGG​ACC​CAC​ACA​CAG​TAG-3′. The amplified PCR products were purified using the QIAquick PCR purification kit (Qiagen) and subjected to Sanger sequencing. Sequences were compared to the reference sequence NM_000383.3 of *AIRE* published at the National Centre for Biotechnology Information and analyzed using SnapGene software (GSL Biotech LLC). All *AIRE* variants were deposited in ClinVar (accession numbers SCV007615326, SCV007615327, SCV007615328, SCV007615329, SCV007615330, and SCV007615331).

### T, B, and NK cells immunophenotyping

PBMCs were isolated from heparinized blood by centrifugation on a Ficoll-Paque PLUS gradient (GE Healthcare). For immunophenotyping, 0.25 million cells were incubated for 30 min at 4°C in the dark with fluorochrome-labeled antibodies that detect the different T cell populations using the surface markers CD3-PE/CY7 (UCHT1; Cat# 300420), CD4-PE (Clone OKT4; Cat# 317410), CD8-AF700 (Clone RPA-T8; Cat# 344724), CD45RA-PB (Clone HI100; Cat# 304123), CCR7-FITC (Clone G043H7; Cat# 353216), CD31-PE/Dazzle (WM59; Cat# 303130), B cell population using the surface markers CD19-PerCP/CY5.5 (Clone HIB19, Cat# 302230), CD27-PE/Dazzle (Clone M-T271; Cat# 356422), and IgD-AF700 (Clone IA6-2; Cat# 348230), and NK cells using the surface markers CD3-FITC (UCHT1; Cat# 300406), CD16-PB (Clone 3G8; Cat# 302032), and CD56-AF700 (Clone 5.1H11; Cat# 362522), all from BioLegend. Lymphocytes were identified using forward scatter area (FSC-A) and side scatter area. Doublets were excluded using FSC-A vs. FSC height and FSC-A vs. FSC width. Viable cells were identified by excluding Zombie Red L/D-positive cells. T cells were identified by gating on CD3^+^ cells and were further differentiated into T helper cells (CD3^+^CD4^+^), T cytotoxic cells (CD3^+^CD8^+^), naive (CD3^+^CD4^+^CD45RA^+^CCR7^+^ or CD3^+^CD8^+^CD45RA^+^CCR7^+^), central memory (CD3^+^CD4^+^CD45RA^−^CCR7^+^ or CD3^+^CD8^+^CD45RA^−^CCR7^+^), effector memory (CD3^+^CD4^+^CD45RA^−^CCR7^−^ or CD3^+^CD8^+^CD45RA^−^CCR7^−^), terminally differentiated (CD3^+^CD4^+^CD45RA^+^CCR7^−^ or CD3^+^CD8^+^CD45RA^+^CCR7^−^), and recent thymic emigrants T cells (CD3^+^CD4^+^CD31^+^). B cells were identified by gating on CD19^+^ cells and were differentiated into naive (CD19^+^CD27^−^IgD^+^), memory unswitched (CD19^+^CD27^+^IgD^+^), and memory switched (CD19^+^CD27^+^IgD^−^) B cells. NK cells were detected by gating on CD3^−^CD16^+^CD56^+^ cells. Cells were analyzed by flow cytometry on a BD FACS Aria cell sorter (BD Biosciences) and analyzed with FlowJo software (Tree Star Inc.).

### Treg staining

1 million PBMCs were incubated for 30 min at 4°C in the dark with FITC-labeled anti-human CD4 (Clone OKT4; Cat# 317408; BioLegend). Cells were then washed, fixed, and permeabilized using FOXP3 staining kit (Cat# 88-8999-40; Thermo Fisher Scientific) according to the manufacturer’s instructions. Intracellular FOXP3 was detected using AF700-labeled anti-human FOXP3 (Clone PCH101; Cat# 56-4776-4; Thermo Fisher Scientific). Tregs were analyzed by flow cytometry and expressed as by the percentage of CD4^+^FOXP3^+^ cells out of the total CD4^+^ T cell population.

### T cell proliferation

PBMCs were labeled with 2.5 µM carboxyfluorescein succinimidyl ester (CFSE; Cat# 423801; BioLegend) and were either left unstimulated, or T cells were stimulated with 2 µg/ml PHA (Cat# L1668; Sigma-Aldrich) or 100 ng/ml anti-CD3 (Clone OKT3; Cat # 317326; BioLegend) + 1 µg/ml anti-CD28 (Clone CD28.2; Cat# 302934; BioLegend) antibodies. 5 days later, samples were stained with PE-labeled anti-CD4 (Clone OKT4; Cat# 317410; BioLegend) and AF700-labeled anti-CD8 (Clone RPA-T8; Cat# 344724; BioLegend) antibodies, and their proliferation was analyzed by flow cytometry. Proliferated, CFSE low, CD4^+^, and CD8^+^ T cells were gated, and the number of cell divisions were determined using the proliferation modeling tool with FlowJo software (Tree Star Inc.) and quantified using GraphPad PRISM V8.0.

### Statistical analysis

Descriptive statistics were used to summarize the study population. The age of onset as a continuous variable was described using the median and IQR. The frequencies of patients’ clinical manifestations and mutations were reported as percentages. Descriptive e analyses were performed using SPSS Statistics software V28.0.0.0 (IBM Corp.). The bar graphs were generated using GraphPad PRISM V8.0 (GraphPad Software). The student’s *t* test was used to compare the mean and standard deviation of the differences in Treg percentages, FOXP3 MFI, and T cell proliferation between control and patients using GraphPad PRISM V8.0.

### Online supplemental material

The supplemental material includes the demographic information, clinical manifestations, and *AIRE* mutations of all APS-1 patients described in the literature (Table S1), their complete list of clinical manifestations and their prevalence (Table S2), as well as the lists of all homozygous (Table S3) and heterozygous (Tables S4 and S5) variants identified in the patients.

## Supplementary Material

Table S1 shows demographic information, clinical manifestations, and AIRE mutations of all APS-1 patients reported in the literature.

Table S2 shows list of all clinical manifestations and their prevalence in APS-1 patients reported in the literature.

Table S3 shows list of all homozygous AIRE mutations in APS-1 patients reported in the literature.

Table S4 shows list of all heterozygous AIRE mutations in APS-1 patients reported in the literature.

Table S5 shows list of compound heterozygous, autosomal dominant, and autosomal recessive AIRE mutations in APS-1 patients and patients with a milder phenotype reported in the literature.

## Data Availability

All data are displayed in the tables and figures of the paper or the supplemental material. Additional original raw data are available from the authors upon request.
