# Supplementary material for: Clinical, genetic, and immunologic features of APS-1 patients from the Middle East, and a review of the literature
Source: J Hum Immun. 2026 Aug 3;2(5):e20250254. doi: 10.70962/jhi.20250254 (PMC13431174; doi:10.70962/jhi.20250254)
Supplement: Table S1 — shows demographic information, clinical manifestations, and AIRE mutations of all APS-1 patients reported in the literature. [file jhi_20250254_tables1.docx]

**Supplemental Table 1.** Demographic information, clinical manifestations, and *AIRE* mutations of all APS-1 patients reported in the literature.

| Patient | Age of onset | Sex | Parents’ Consanguinity | Clinical Manifestations (Age) | Mutation (Domain) | Reference |  |
| --- | --- | --- | --- | --- | --- | --- | --- |
|  | 6 years | Female | Consanguineous (2^nd^ degree cousins) | - Recurrent oral candidiasis  - Hypoparathyroidism  - Addison's disease  - Alopecia areata  - Psoriasis  - Papillary thyroid cancer | c.44G>A/c.44G>A p.R15H/p.R15H (HSR/CARD) | Present report |  |
|  | 3 years | Male | Consanguineous (1^st^ degree cousins) | - Mucocutaneous candidiasis (3Y) - Hypoparathyroidism (5Y) - Alopecia (3Y) - Hypothyroidism (5Y) - Vitiligo (4Y) | c.93_94insT/c.93_94insT p.L32SfsX3/p.L32SfsX3 (HSR/CARD) | Present report |  |
|  | 10 years | Male | Consanguineous (1^st^ degree cousins) | - Recurrent oral candidiasis  - Hypoparathyroidism  - Addison's disease  - Recurrent keratoconjunctivitis | c.199_202delCTGAinsTGG/c.199_202delCTGAinsTGG  p.L67WfsX80/p.L67WfsX80 (L) | Present report |  |
|  | 6 years | Male | Consanguineous (1^st^ degree cousins) | - Hypoparathyroidism (6Y)  - Addison's disease (7Y)  - Diabetes mellitus type 1 (8Y)  - Hypothyroidism (7Y) | c.199_202delCTGAinsTGG/c.199_202delCTGAinsTGG  p.L67WfsX80/p.L67WfsX80 (L) | Present report |  |
|  | 4 years | Female | Consanguineous (1^st^ degree cousins) | - Candida onychomycosis  - Hypoparathyroidism  - Addison's disease  - Diabetes mellitus type 1  - Primary ovarian failure  - Recurrent keratoconjunctivitis (7Y) | c.199_202delCTGAinsTGG/c.199_202delCTGAinsTGG  p.L67WfsX80/p.L67WfsX80 (L) | Present report |  |
|  | 4 years | Male | Consanguineous (1^st^ degree cousins) | - Hypoparathyroidism  - Addison's disease (7Y)  - Diabetes mellitus type 1 (8Y)  - Vitiligo | c.199_202delCTGAinsTGG/c.199_202delCTGAinsTGG  p.L67WfsX80/p.L67WfsX80 (L) | Present report |  |
|  | 7 years | Male | Consanguineous (1^st^ degree cousins) | - Recurrent oral candidiasis  - Hypoparathyroidism  - Addison's disease  - Alopecia areata  - Diabetes mellitus type 1  - Pernicious anemia | c.199_202delCTGAinsTGG/c.199_202delCTGAinsTGG  p.L67WfsX80/p.L67WfsX80 (L) | Present report |  |
|  | 5 years | Male | Consanguineous (1^st^ degree cousins) | - Hypoparathyroidism (5Y)  - Addison's disease (5Y)  - Alopecia (10Y)  - Diabetes mellitus type 1 (7Y)  - Recurrent keratoconjunctivitis (7Y) | c.199_202delCTGAinsTGG/c.199_202delCTGAinsTGG  p.L67WfsX80/p.L67WfsX80 (L) | Present report |  |
|  | 5 years | Male | Consanguineous (1^st^ degree cousins) | - Diabetes mellitus type 1 (5Y) | c.199_202delCTGAinsTGG/c.199_202delCTGAinsTGG  p.L67WfsX80/p.L67WfsX80 (L) | Present report |  |
|  | 7 years | Male | Consanguineous (1^st^ degree cousins) | - Recurrent oral candidiasis (9Y)  - Hypoparathyroidism (7Y)  - Addison's disease (8Y) | c.205_208dupCAGG/c.205_208dupCAGG  p.D70AfsX148/p.D70AfsX148 (HSR/CARD) | Present report |  |
|  | 4 years | Female | Consanguineous (1^st^ degree cousins) | - Hypoparathyroidism (4Y)  - Alopecia areata (6Y) | c.205_208dupCAGG/c.205_208dupCAGG  p.D70AfsX148/p.D70AfsX148 (HSR/CARD) | Present report |  |
|  | 2 years | Female | Consanguineous (1^st^ degree cousins) | - Recurrent oral candidiasis (2Y)  - Hypoparathyroidism (2Y)  - Addison's disease (4Y)  - Perianal abscess (4Y) | c.205_208dupCAGG/c.205_208dupCAGG  p.D70AfsX148/p.D70AfsX148 (HSR/CARD) | Present report |  |
|  | 3 years | Female | Consanguineous (2^nd^ degree cousins) | - Recurrent oral candidiasis (3Y)  - Hypoparathyroidism (4Y) - Vitiligo (3Y) | c.205_208dupCAGG/c.205_208dupCAGG  p.D70AfsX148/p.D70AfsX148 (HSR/CARD) | Present report |  |
|  | N/A | Female | Consanguineous (2^nd^ degree cousins) | - Recurrent oral candidiasis  - Hypoparathyroidism  - Addison's disease  - Primary ovarian failure | c.205_208dupCAGG/c.205_208dupCAGG  p.D70AfsX148/p.D70AfsX148 (HSR/CARD) | Present report |  |
|  | 4 years | Female | Consanguineous (2^nd^ degree cousins) | - Recurrent oral candidiasis  - Hypoparathyroidism  - Addison's disease  - Alopecia areata | c.205_208dupCAGG/c.205_208dupCAGG  p.D70AfsX148/p.D70AfsX148 (HSR/CARD) | Present report |  |
|  | 1 year | Female | Consanguineous (2^nd^ degree cousins) | - Hypoparathyroidism (4Y)  - Congenital adrenal hypoplasia  - Asthma  - Chronic cough  - Dry skin  - Nephrocalcinosis - Recurrent infections (1Y) | c.1096-1G>A/c.1096-1G>A E10del/E10del (PRR, L) | Present report |  |
|  | 7 days | Male | Consanguineous (2^nd^ degree cousins) | - Hyperactive airways  - Hyperinsulinemia hyperglycemia (7D)  - Diaper rash  - Recurrent infections (7W) - Right trigger thumb | c.1096-1G>A/c.1096-1G>A E10del/E10del (PRR, L) | Present report |  |
|  | 4 years | Female | Consanguineous (1^st^ degree cousins) | - Chronic mucocutaneous candidiasis  - Hypoparathyroidism (6Y)  - Atrophic glossitis  - Fingernail candidiasis  - Autoimmune hepatitis (4Y)  - Nail dystrophy  - Perleche  - Seizures  - Tooth enamel hypoplasia | c.1311C>A/c.1311C>A p.C437X/p.C437X (PHD2) | Present report |  |
|  | 2 years | Male | N/A | - Mucocutaneous candidiasis (3Y)  - Addison’s disease (2Y)  - Allergy  - Chronic/tension headaches - Ectodermal dystrophy (3Y) | g.(?_44285987)_(44297747_?)del/c.967_979del13bp complete deletion of AIRE/p.L323fs (PHD1) | ([1](#_ENREF_1)) |  |
|  | 5 years | Female | N/A | - Mucocutaneous candidiasis (7Y)  - Hypoparathyroidism (5Y)  - Addison’s disease (6Y)  - Ectodermal dystrophy (8Y)  - Hypogonadism (9Y)  - Hypothalamic–pituitary dysfunction | g.(?_44285987)_(44297747_?)del/c.967_979del13bp Complete deletion of AIRE/p.L323fs (PHD1) | ([1](#_ENREF_1)) |  |
|  | 0.1 year | Female | Consanguineous | - Mucocutaneous candidiasis (neonatal period)  - Hypoparathyroidism (4Y) - Addison’s disease (6Y) - Alopecia universalis (9Y) - Keratopathy (6Y) | c.1A>G/c.1A>G p.M1V/p.M1V (HSR/CARD) | ([2](#_ENREF_2)) |  |
|  | 4 years | Female | N/A | - Mucocutaneous candidiasis (4Y) - Hypoparathyroidism (4Y) - Alopecia  - Anemia  - Diarrhea - Enamel hypoplasia  - Keratoconjunctivitis  - Pernicious anemia - Sicca syndrome  - Vitiligo | c.1A>G/c.1A>G p.M1V/p.M1V (HSR/CARD) | ([3](#_ENREF_3)) |  |
|  | 1 year | Male | N/A | - Mucocutaneous candidiasis (19Y) - Hypoparathyroidism (1Y) - Primary adrenal insufficiency (17Y) - Enamel hypoplasia  - Facial dysmorphism - Isolated hypogonadotropic hypogonadism | c.1A>G/c.274C>T p.M1V (HSR/CARD)/p.R92W (HSR/CARD) | ([3](#_ENREF_3)) |  |
|  | 4 years | Male | N/A | - Mucocutaneous candidiasis (5Y) - Hypoparathyroidism (4Y) | c.1A>G/c.274C>T p.M1V (HSR/CARD)/p.R92W (HSR/CARD) | ([3](#_ENREF_3)) |  |
|  | N/A | Male | N/A | N/A | c.1A>G/c.274C>T p.M1V (HSR/CARD)/p.R92W (HSR/CARD) | ([3](#_ENREF_3)) |  |
|  | 6 years | Female | N/A | - Fungal infections (6Y) - Hypoparathyroidism (6Y)  - Addison’s disease (9Y) - Aggressive hepatitis (6Y) | c.1A>T/c.769C>T p.M1L (HSR/CARD)/p.R257X (SAND) | ([4](#_ENREF_4)) |  |
|  | 3 years | Female | N/A | - Fungal infections (3Y)  - Hypoparathyroidism (3Y)  - Addison’s disease (9Y) - Chronic hepatitis  - Malabsorption | c.1A>T/c.769C>T p.M1L (HSR/CARD)/p.R257X (SAND) | ([4](#_ENREF_4)) |  |
|  | 6 years | Female | N/A | - Chronic mucocutaneous candidiasis (6Y)  - Hypoparathyroidism (6Y) - Adrenal insufficiency (6Y) | c.2T>C/c.769C>T p.M1T (HSR/CARD)/p.R257X (SAND) | ([5](#_ENREF_5)) |  |
|  | 0.1 year | N/A | N/A | - Chronic mucocutaneous candidiasis (0.1Y)  - Hypoparathyroidism (9Y) - Adrenal insufficiency (9Y)  - Alopecia (21.5Y)  - Hypergonadotropic hypogonadism (19Y)  - Hypothyroidism (9Y) | c.2T>C/c.769C>T p.M1T (HSR/CARD)/p.R257X (SAND) | ([6](#_ENREF_6)) |  |
|  | 2 years | Male | N/A | - Mucocutaneous candidiasis (2Y) | c.21_43dup23bp/c.21_43dup23 p.R15fs/p.R15fs (HSR/CARD) | ([1](#_ENREF_1)) |  |
|  | 1.8 years | Male | N/A | - Mucocutaneous candidiasis (1.8Y) | c.21_43dup23bp/c.21_43dup23 p.R15fs/p.R15fs (HSR/CARD) | ([7](#_ENREF_7)) |  |
|  | 5.8 years | Male | N/A | - Mucocutaneous candidiasis (5.8Y)  - Hypoparathyroidism (7.9Y) | c.21_43dup23bp/c.21_43dup23 p.R15fs/p.R15fs (HSR/CARD) | ([7](#_ENREF_7)) |  |
|  | 7.8 years | Male | Non-consanguineous | - Mucocutaneous candidiasis (23.3Y)  - Addison’s disease (7.8Y)  - Ectodermal dystrophy (7.8Y)  - Hypogonadism (21.3Y)  - Hypothyroidism (26.3Y) | c.21_43dup23bp/c.769C>T p.R15fs (HSR/CARD)/p.R257X (SAND) | ([7](#_ENREF_7), [8](#_ENREF_8)) |  |
|  | N/A | Female | N/A | - Mucocutaneous candidiasis  - Hypoparathyroidism  - Adrenal failure  - Alopecia  - Autoimmune thyroiditis | c.21_43dup23bp/c.769C>T p.R15fs (HSR/CARD)/p.R257X (SAND) | ([9](#_ENREF_9)) |  |
|  | N/A | N/A | N/A | - Mucocutaneous candidiasis  - Hypoparathyroidism  - Addison’s disease  - Gastritis  - Hypothyroidism | c.21_43dup23bp/c.769C>T p.R15fs (HSR/CARD)/p.R257X (SAND) | ([10](#_ENREF_10)) |  |
|  | N/A | N/A | N/A | - Mucocutaneous candidiasis  - Hypoparathyroidism  - Addison’s disease  - Hypergonadotropic hypogonadism | c.21_43dup23bp/c.769C>T p.R15fs (HSR/CARD)/p.R257X (SAND) | ([10](#_ENREF_10)) |  |
|  | N/A | Male | N/A | - Hypoparathyroidism  - Adrenal insufficiency  - Alopecia | c.22C>T/c.402delC p.R8C (L)/p.S135QfsX12 (downstream of NLS) | ([11](#_ENREF_11)) |  |
|  | 9 years | Female | N/A | - Mucocutaneous candidiasis  - Hypoparathyroidism (9Y)  - Primary gonadal insufficiency (17Y)  - Alopecia  - Dental enamel hypoplasia  - Nail pitting | c.22C>T/c.290T>C p.R8C (L)/p.L97P (HSR/CARD) | ([11](#_ENREF_11)) |  |
|  | 4 years | Female | Consanguineous (1^st^ degree cousins) | - Alopecia areata then universalis (6Y)  - Precocious puberty (4Y) | c.44G>A/c.44G>A p.R15H/p.R15H (HSR/CARD) | ([12](#_ENREF_12)) |  |
|  | N/A | N/A | N/A | - Candidiasis  - Hypoparathyroidism  - Adrenal insufficiency  - Hypothyroidism | c.44G>A/c.44G>A p.R15H/p.R15H (HSR/CARD) | ([13](#_ENREF_13)) |  |
|  | 5 years | Female | Consanguineous (1^st^ degree cousins) | - Recurrent chronic  mucocutaneus candidiasis (5Y) - Hypoparathyroidism (9Y)  - Primary adrenal deficiency (9Y)  - Autoimmune hepatitis (17Y) - Autoimmune thyroiditis (15Y)  - Asplenia  - Enlarged thymus  - Hepatopulmonary syndrome (17Y)  - Hyperpigmentation  - Ovarian insufficiency (15Y)  - Pan-gastritis (17Y)  - Short stature | c.44G>A/c.44G>A p.R15H/p.R15H (HSR/CARD) | ([14](#_ENREF_14), [15](#_ENREF_15)) |  |
|  | 9 years | Male | Consanguineous (1^st^ degree cousins) | - Chronic mucocutaneous candidiasis (21Y)  - Hypoparathyroidism (9Y) | c.44G>A/c.44G>A p.R15H/p.R15H (HSR/CARD) | ([14](#_ENREF_14), [15](#_ENREF_15)) |  |
|  | 1 year | Female | Consanguineous (3^rd^ degree cousins) | - Mucocutaneous candidiasis  - Hypoparathyroidism  - Alopecia  - Ectodermal dystrophy | c.47C>T/c.47C>T p.T16M/p.T16M (HSR/CARD) | ([16](#_ENREF_16)) |  |
|  | 6 years | Female | Non-consanguineous | - Chronic mucocutaneous candidiasis  - Chronic hypoparathyroidism  - Addison’s disease  - Alopecia  - Enamel dysplasia | c.47C>T/c.47C>T p.T16M/p.T16M (HSR/CARD) | ([17](#_ENREF_17)) |  |
|  | N/A | N/A | N/A | N/A | c.47C>T/c.47C>T p.T16M/p.T16M (HSR/CARD) | ([18](#_ENREF_18)) |  |
|  | N/A | N/A | N/A | N/A | c.47C>T/c.47C>T p.T16M/p.T16M (HSR/CARD) | ([18](#_ENREF_18)) |  |
|  | N/A | N/A | N/A | N/A | c.47C>T/c.47C>T p.T16M/p.T16M (HSR/CARD) | ([18](#_ENREF_18)) |  |
|  | N/A | N/A | N/A | N/A | c.47C>T/c.232T>C p.T16M (HSR/CARD)/p.W78R (HSR/CARD) | ([18](#_ENREF_18)) |  |
|  | N/A | N/A | N/A | N/A | c.47C>T/c.232T>C p.T16M (HSR/CARD)/p.W78R (HSR/CARD) | ([18](#_ENREF_18)) |  |
|  | N/A | N/A | N/A | N/A | c.47C>T/Unknown p.T16M (HSR/CARD)/p.S196S (SAND) | ([18](#_ENREF_18)) |  |
|  | N/A | N/A | N/A | N/A | c.47C>T/c.47C>T+c.755C>T p.T16M (HSR/CARD)/p.T16M (HSR/CARD)+P252L (SAND) | ([18](#_ENREF_18)) |  |
|  | N/A | N/A | N/A | N/A | c.47C>T/c.47C>T+c.755C>T p.T16M (HSR/CARD)/p.T16M (HSR/CARD)+P252L (SAND) | ([18](#_ENREF_18)) |  |
|  | N/A | N/A | N/A | N/A | c.47C>T/c.319_321delAGCinsTG  p.T16M (HSR/CARD)/p.S107fs (upstream of NLS) | ([18](#_ENREF_18)) |  |
|  | N/A | N/A | N/A | N/A | c.47C>T/c.892G>A p.T16M (HSR/CARD)/p.E298K (upstream of PHD1) The E298K mutation was wrongly reported as G298L in the paper | ([18](#_ENREF_18)) |  |
|  | N/A | N/A | N/A | N/A | c.47C>T/c.62C>T p.T16M (HSR/CARD)/p.A21V (HSR/CARD) | ([18](#_ENREF_18)) |  |
|  | N/A | N/A | N/A | N/A | c.47C>T/c.967_979del13bp p.T16M (HSR/CARD)/p.L323fs (PHD1) | ([18](#_ENREF_18)) |  |
|  | N/A | N/A | N/A | - Candidiasis  - Autoimmune thyroiditis  - Nail dystrophy | c.47C>T/c.232T>C p.T16M (HSR/CARD)/p.W78R (HSR/CARD) | ([19](#_ENREF_19)) |  |
|  | N/A | N/A | N/A | - Candidiasis  - Hypoparathyroidism  - Addison's disease | c.47C>T/c.62C>T p.T16M (HSR/CARD)/p.A21V (HSR/CARD) | ([19](#_ENREF_19)) |  |
|  | 0.7 year | Female | Non-consanguineous | - Chronic candidiasis  - Chronic hypoparathyroidism (3.2Y)  - Alopecia aerate (3Y)  - Nail dystrophy | c.47C>T/c.319_321delAGCinsTG  p.T16M (HSR/CARD)/p.S107fs (upstream of NLS) | ([20](#_ENREF_20)) |  |
|  | 2 years | Female | Non-consanguineous | - Chronic mucocutaneous candidiasis  - Chronic hypoparathyroidism  - Addison’s disease  - Alopecia  - Autoimmune thyroiditis  - Ectodermal dystrophy  - Enamel dysplasia  - GH deficiency  - Hypergonadotropic hypogonadism  - Stipsis/diarrhea | c.47C>T/c.232T>C p.T16M (HSR/CARD)/p.W78R (HSR/CARD) | ([17](#_ENREF_17)) |  |
|  | 0.7 year | Female | Non-consanguineous | - Chronic mucocutaneous candidiasis  - Alopecia  - Autoimmune thyroiditis  - Stipsis/diarrhea  - Vasculitis | c.47C>T/c.232T>C p.T16M (HSR/CARD)/p.W78R (HSR/CARD) | ([17](#_ENREF_17)) |  |
|  | N/A | Female | Non-consanguineous | - Chronic mucocutaneous candidiasis  - Hypoparathyroidism  - Addison’s disease  - Alopecia areata  - Dystrophic teeth with yellowish discoloration  - Onychomycosis | c.47C>T/c.169C>T p.T16M (HSR/CARD)/p.Q57X (HSR/CARD) | ([21](#_ENREF_21)) |  |
|  | 11 years | Male | N/A | - Hypoparathyroidism (11Y)  - Adrenal Insufficiency (13Y)  - Diabetes Mellitus (16Y)  - Vitiligo (17Y) | c.47C>T/c.821delG p.T16M (HSR/CARD)/p.G274fs (SAND) | ([5](#_ENREF_5)) |  |
|  | 10 years | Male | N/A | - Chronic mucocutaneous candidiasis (10Y)  - Adrenal insufficiency (10Y)  - Alopecia (10Y) | c.47C>T/c.769C>T p.T16M (HSR/CARD)/p.R257X (SAND) | ([5](#_ENREF_5)) |  |
|  | 0.2 year | Female | N/A | - Chronic mucocutaneous candidiasis (0.2Y)  - Hypoparathyroidism (9Y)  - Adrenal insufficiency (9Y)  - Alopecia (20Y)  - Hypothyroidism (9Y)  - Malabsorption (26Y)  - Primary ovarian insufficiency (19Y) | c.47C>T/c.769C>T p.T16M (HSR/CARD)/p.R257X (SAND) | ([5](#_ENREF_5)) |  |
|  | 6 years | Male | N/A | - Chronic mucocutaneous candidiasis (6.1Y)  - Pernicious anemia (8.5Y)  - Adrenal insufficiency (10.3Y)  - Alopecia (20Y)  - Chronic elevation of liver enzymes (10Y)  - Dry eyes (20Y) | c.47C>T/c.769C>T p.T16M (HSR/CARD)/p.R257X (SAND) | ([5](#_ENREF_5), [6](#_ENREF_6)) |  |
|  | 0.6 year | Female | Non-consanguineous | - Mucocutaneous candidiasis (2Y)  - Chronic idiopathic hypoparathyroidism (9Y)  - Addison disease (11Y)  - Abdominal pain with stipsis and diarrhea (5Y)  - Alopecia (5Y)  - Autoimmune thyroiditis (5Y)  - Cutaneous vasculitis (7M)  - Ectodermal dystrophy (9Y)  - Ovarian failure (12Y)  - Vitiligo (9Y) | c.47C>T/c.232T>A  p.T16M (HSR/CARD)/p.W78R (HSR/CARD) | ([22](#_ENREF_22)) |  |
|  | 3.7 years | Male | Non-consanguineous | - Mucocutaneous candidiasis (3.7Y)  - Hypoparathyroidism (3.7Y)  - Alopecia (3.7Y)  - Ectodermal dystrophy (3.7Y)  - Malabsorption (6.3Y) | c.47C>T/c.769C>T p.T16M (HSR/CARD)/p.R257X (SAND) | ([8](#_ENREF_8)) |  |
|  | 5.5 years | Female | Non-consanguineous | - Mucocutaneous candidiasis (11.4Y)  - Hypoparathyroidism (5.5)  - Ectodermal dystrophy (5.5Y) | c.47C>T/c.769C>T p.T16M (HSR/CARD)/p.R257X (SAND) | ([8](#_ENREF_8)) |  |
|  | N/A | N/A | N/A | - Mucocutaneous candidiasis  - Hypoparathyroidism  - Addison's Disease  - Hypothyroidism | c.47C>T/c.769C>T p.T16M (HSR/CARD)/p.R257X (SAND) | ([10](#_ENREF_10)) |  |
|  | N/A | N/A | N/A | N/A | c.62C>T/c.62C>T p.A21V/p.A21V (HSR/CARD) | ([18](#_ENREF_18)) |  |
|  | N/A | N/A | N/A | N/A | c.62C>T/c.62C>T p.A21V/p.A21V (HSR/CARD) | ([23](#_ENREF_23)) |  |
|  | N/A | N/A | N/A | N/A | c.62C>T/c.769C>T  p.A21V (HSR/CARD)/p.R257X (SAND) | ([18](#_ENREF_18)) |  |
|  | N/A | N/A | N/A | N/A | c.62C>T/c.769C>T  p.A21V (HSR/CARD)/p.R257X (SAND) | ([18](#_ENREF_18)) |  |
|  | N/A | N/A | N/A | N/A | c.62C>T/c.967_979del13bp  p.A21V (HSR/CARD)/p.L323fs (PHD1) | ([18](#_ENREF_18)) |  |
|  | N/A | N/A | N/A | N/A | c.62C>T/Unknown  p.A21V (HSR/CARD)/Unknown | ([23](#_ENREF_23)) |  |
|  | N/A | N/A | N/A | N/A | c.62C>T/Unknown  p.A21V (HSR/CARD)/Unknown | ([23](#_ENREF_23)) |  |
|  | 4 years | Male | Non-consanguineous | - Mucocutaneous candidiasis (5Y) - Hypoparathyroidism (8Y)  - Adrenal insufﬁciency (8Y) - Alopecia (6Y) - Chronic malabsorption (11Y)  - Gingivitis (6Y)  - Mild photophobia (20Y), loss of lashes, shortened tear break-up time, conjunctival redness, and inferior superﬁcial punctate staining - Pernicious anemia (11Y)  - Pulmonary involvement (4Y) | c.62C>T/c.1096-1G>A  p.A21V (HSR/CARD)/E10del (PRR, L) | ([24](#_ENREF_24)) |  |
|  | 2 years | Female | Non-consanguineous | - Chronic mucocutaneous candidiasis - Chronic hypoparathyroidism  - Addison’s disease  - Ectodermal dystrophy | c.62C>T/c.967_979del13bp  p.A21V (HSR/CARD)/p.L323fs (PHD1) | ([17](#_ENREF_17)) |  |
|  | N/A | Female | N/A | - Mucocutaneous candidiasis  - Adrenal insufficiency  - Congenital adrenal hyperplasia  - Hepatitis  - Malabsorption  - Pernicious anemia  - Primary gonadal insufficiency | c.62C>T/c.1163_1164insA p.A21V (HSR/CARD)/p.M388fs (PRR) | ([25](#_ENREF_25)) |  |
|  | N/A | N/A | N/A | N/A | c.83T>C/Unknown p.L28P (HSR/CARD)/Unknown | ([26](#_ENREF_26)) |  |
|  | N/A | Male | Non-consanguineous | - Chronic mucocutaneous candidiasis - Adrenal failure  - Pernicious anemia - Red-cell aplasia - Squamous-cell carcinoma of oral mucosa  - Type 1 diabetes | c.83T>C/c.1249delC p.L28P (HSR/CARD)/p.L417fs (L)  It was mistakenly written K417fs in the reference | ([27](#_ENREF_27)) |  |
|  | N/A | Male | N/A | - Candidiasis - Adrenal failure - Malabsorption - Type 1 diabetes | c.83T>C/c.1249delC p.L28P (HSR/CARD)/p.L417fs (L) It was mistakenly written K417fs in the reference | ([28](#_ENREF_28)) |  |
|  | 1.5 years | Male | Consanguineous (3^rd^ degree cousins) | - Chronic mucocutaneous candidiasis (5Y)  - Chronic hypoparathyroidism (5Y)  - Addison’s disease (5Y)  - Abdominal bloating (5Y)  - Alopecia (5Y)  - Atrophic gastritis (5Y)  - Chronic hepatitis (1.5Y)  - Chronic thyroiditis with hypothyroidism (5Y)  - Ectodermal dystrophy (5Y)  - Posterior reversible encephalopathy syndrome (5Y)  - Stipsis/diarrhea  - Urticaria-like erythema fever (5Y)  - Vitiligo (5Y) | c.132+1_132+3delGTGinsCT/c.132+1_132+3delGTGinsCT  Abolishes E1-E2 splicing (NLS) | ([17](#_ENREF_17), [29](#_ENREF_29)) |  |
|  | 4 years | Female | Consanguineous (3^rd^ degree cousins) | - Chronic mucocutaneous candidiasis  - Chronic hypoparathyroidism  - Ectodermal dystrophy | c.132+1_132+3delGTGinsCT/c.132+1_132+3delGTGinsCT  Abolishes E1-E2 splicing (NLS) | ([17](#_ENREF_17)) |  |
|  | 14 years | Male | N/A | - Mucocutaneous candidiasis (14Y)  - Hypoparathyroidism (14Y) | c.132+1_132+3delGTGinsCT/c.769C>T Abolishes E1-E2 splicing (NLS)/p.R257X (SAND) | ([4](#_ENREF_4)) |  |
|  | 1 year | Female | Non-consanguineous | - Hypoparathyroidism (3Y)  - Chronic abdominal pain, bloating, and diarrhea  - Chronic polyarthritis (4Y)  - Hepatitis (4Y)  - Interstitial pneumonitis (4Y) - Mild eczema  - Pneumonia (1Y)  - Poor dentition  - Recurrent otitis media (2Y) | c.132+1_132+3delGTGinsCT/c.769C>T Abolishes E1-E2 splicing (NLS)/p.R257X (SAND) | ([30](#_ENREF_30)) |  |
|  | 4 years | Female | N/A | - Hypoparathyroidism (4Y)  - Alopecia (5Y)  - Conjunctivitis and keratitis (13Y)  - Connective tissue disorder (15Y)  - Hypogonadism (13Y)  - Hypopituitarism (11Y)  - Hypothyroidism (5Y)  - Vitiligo (9Y) | c.132+1_132+3delGTGinsCT/Unknown  Abolishes E1-E2 splicing (NLS)/Unknown | ([31](#_ENREF_31)) |  |
|  | N/A | N/A | N/A | - Mucocutaneous candidiasis  - Hypoparathyroidism - Addison’s disease - Alopecia  - Malabsorption | IVS1_IVS4/IVS1_IVS4 E2-E4del/E2-E4del (L, HSR/CARD, NLS) | ([10](#_ENREF_10), [15](#_ENREF_15)) |  |
|  | 5 years | Female | Consanguineous (1^st^ degree cousins) | - Mucocutaneous candidiasis  - Adrenal insufﬁciency  - Alopecia  - Hepatitis  - Severe keratopathy: loss of lashes, severe corneal stromal opacities, and deep neovascularization. | c.173C>A/c.173C>A p.A58D/p.A58D (HSR/CARD) | ([24](#_ENREF_24)) |  |
|  | 9 years | Male | N/A | - Mucocutaneous candidiasis | c.195G>A/c.195G>A p.W65X/p.W65X (L) | ([32](#_ENREF_32)) |  |
|  | 10 years | Female | N/A | - Chronic persistent candidiasis  - Hypoparathyroidism | c.195G>A/c.769C>T  p.W65X (L)/p.R25X (SAND) | ([32](#_ENREF_32)) |  |
|  | 2 years | Male | Consanguineous (2^nd^ degree cousins) | - Hypoparathyroidism (2Y)  - Primary adrenal insufficiency (19Y)  - Binocular cataract (14Y)  - Chronic intestinal dysfunction (14Y)  - Enamel dystrophy (14Y) | c.206A>C/c.206A>C p.Q69P/p.Q69P (HSR/CARD) | ([33](#_ENREF_33), [34](#_ENREF_34)) |  |
|  | 5 years | Male | Consanguineous (2^nd^ degree cousins) | - Recurrent oral candidiasis (10Y)  - Addison disease (13Y)  - Autoimmune depletion of enteroendocrine cells (13Y)  - Autoimmune diabetes (13Y)  - Chronic recurrent intermittent diarrhea (5Y)  - Eosinophilic ileitis and focal active colitis (13Y)  - Functional hyposplenism (13Y)  - Necrotizing pneumonia and abscess (13Y)  - Oligoarticular juvenile idiopathic arthritis (10Y)  - Renal tubulopathy (13Y) | c.205_208dup/c.205_208dup p.D70fs/p.D70fs (HSR/CARD) | ([35](#_ENREF_35)) |  |
|  | 2 years | Female | Consanguineous | - Hypoparathyroidism (2Y) | c.205_208dup/c.205_208dup p.D70fs/p.D70fs (HSR/CARD) | ([2](#_ENREF_2)) |  |
|  | 1 year | Male | Consanguineous | - Mucocutaneous candidiasis (1Y)  - Hypoparathyroidism (1Y)  - Addison disease (8Y)  - Nephrocalcinosis (10Y) | c.205_208dup/c.205_208dup p.D70fs/p.D70fs (HSR/CARD) | ([2](#_ENREF_2)) |  |
|  | N/A | N/A | N/A | N/A | c.205_208dup/c.205_208dup p.D70fs/p.D70fs (HSR/CARD) | ([26](#_ENREF_26)) |  |
|  | 6 years | Male | Non-consanguineous | - Chronic candidiasis (6Y)  - Chronic hypoparathyroidism (19Y)  - Addison’s disease (19Y)  - Alopecia  - Cancer of the esophagus  - Malignant melanoma | c.232T>C/c.232T>C p.W78R/p.W78R (HSR/CARD) | ([36](#_ENREF_36)) |  |
|  | 1 year | Male | Non-consanguineous | - Candidiasis  - Hypoparathyroidism  - Addison’s disease  - Alopecia  - Atrophic gastritis  - Cataract  - Enamel hypoplasia  - Nephrocalcinosis  - Tympanic calcifications | c.232T>C/c.232T>C p.W78R/p.W78R (HSR/CARD) | ([37](#_ENREF_37)) |  |
|  | 1 year | Female | Non-consanguineous | - Candidiasis  - Hypoparathyroidism  - Addison’s disease  - Alopecia  - Autoimmune hepatitis  - Enamel hypoplasia  - Hypogonadism  - Hypothyroidism  - Keratitis  - Malabsorption | c.232T>C/c.232T>C p.W78R/p.W78R (HSR/CARD) | ([37](#_ENREF_37)) |  |
|  | 6 years | Female | Non-consanguineous | - Candidiasis  - Hypoparathyroidism  - Alopecia  - Atrophic gastritis  - Cataract  - Enamel hypoplasia  - Hypogonadism  - Nephrocalcinosis | c.232T>C/c.232T>C p.W78R/p.W78R (HSR/CARD) | ([37](#_ENREF_37)) |  |
|  | 1 year | Male | Non-consanguineous | - Candidiasis  - Hypoparathyroidism  - Addison’s disease  - Alopecia  - Cataract  - Enamel hypoplasia | c.232T>C/c.232T>C p.W78R/p.W78R (HSR/CARD) | ([37](#_ENREF_37)) |  |
|  | <1 year | Female | Non-consanguineous | - Candidiasis  - Hypoparathyroidism  - Addison’s disease  - Cataract  - Enamel hypoplasia  - Tympanic calcifications | c.232T>C/c.232T>C p.W78R/p.W78R (HSR/CARD) | ([37](#_ENREF_37)) |  |
|  | N/A | N/A | N/A | N/A | c.232T>C/c.232T>C p.W78R/p.W78R (HSR/CARD) | ([18](#_ENREF_18)) |  |
|  | N/A | N/A | N/A | N/A | c.232T>C/c.232T>C p.W78R/p.W78R (HSR/CARD) | ([18](#_ENREF_18)) |  |
|  | N/A | N/A | N/A | N/A | c.232T>C/c.232T>C p.W78R/p.W78R (HSR/CARD) | ([18](#_ENREF_18)) |  |
|  | N/A | N/A | N/A | N/A | c.232T>C/c.232T>C p.W78R/p.W78R (HSR/CARD) | ([18](#_ENREF_18)) |  |
|  | N/A | N/A | N/A | N/A | c.232T>C/c.232T>C p.W78R/p.W78R (HSR/CARD) | ([18](#_ENREF_18)) |  |
|  | N/A | N/A | N/A | N/A | c.232T>C/c.232T>C p.W78R/p.W78R (HSR/CARD) | ([18](#_ENREF_18)) |  |
|  | N/A | N/A | N/A | N/A | c.232T>C/c.232T>C p.W78R/p.W78R (HSR/CARD) | ([18](#_ENREF_18)) |  |
|  | N/A | N/A | N/A | N/A | c.232T>C/c.232T>C p.W78R/p.W78R (HSR/CARD) | ([18](#_ENREF_18)) |  |
|  | N/A | N/A | N/A | N/A | c.232T>C/c.232T>C p.W78R/p.W78R (HSR/CARD) | ([18](#_ENREF_18)) |  |
|  | N/A | N/A | N/A | N/A | c.232T>C/c.232T>C p.W78R/p.W78R (HSR/CARD) | ([18](#_ENREF_18)) |  |
|  | 5 years | Female | Non-consanguineous | - Chronic mucocutaneous candidiasis (5Y)  - Chronic hypoparathyroidism (5Y)  - Addison’s disease (5Y)  - Alopecia areata (5Y)  - Chronic thyroiditis (29Y)  - Enamel hypoplasia or ectodermal dystrophy (5Y)  - GH deficiency (11Y)  - Hypergonadotropic hypogonadism (12.5Y)  - Nephrocalcinosis (20Y) | c.232T>C/c.232T>C p.W78R/p.W78R (HSR/CARD) | ([38](#_ENREF_38)) |  |
|  | 8 years | Male | Non-consanguineous | - Chronic hypoparathyroidism (8Y)  - Addison’s disease (8Y)  - Chronic thyroiditis (10Y)  - GH deficiency (12Y)  - GI dysfunction (16Y) | c.232T>C/c.232T>C p.W78R/p.W78R (HSR/CARD) | ([38](#_ENREF_38)) |  |
|  | 1 year | Female | Non-consanguineous | - Chronic mucocutaneous candidiasis (1Y)  - Chronic hypoparathyroidism (8Y)  - Addison’s disease (3Y)  - Autoimmune hepatitis (1Y)  - Chronic thyroiditis (3Y)  - GH deficiency (4Y) | c.232T>C/c.232T>C p.W78R/p.W78R (HSR/CARD) | ([38](#_ENREF_38)) |  |
|  | 4 years | Female | N/A | - Chronic hypoparathyroidism (4Y)  - Adrenal insufﬁciency (6Y) | c.232T>C/c.232T>C p.W78R/p.W78R (HSR/CARD) | ([39](#_ENREF_39)) |  |
|  | N/A | N/A | N/A | - Hypoparathyroidism  - Addison's disease  - Autoimmune thyroiditis | c.232T>C/c.232T>C p.W78R/p.W78R (HSR/CARD) | ([19](#_ENREF_19)) |  |
|  | N/A | N/A | N/A | - Candidiasis  - Addison's disease  - Autoimmune hepatitis  - Autoimmune thyroiditis - Nail dystrophy | c.232T>C/c.232T>C p.W78R/p.W78R (HSR/CARD) | ([19](#_ENREF_19)) |  |
|  | N/A | N/A | N/A | - Diarrhea | c.232T>C/c.232T>C p.W78R/p.W78R (HSR/CARD) | ([40](#_ENREF_40)) |  |
|  | N/A | N/A | N/A | N/A | c.232T>C/c.232T>C p.W78R/p.W78R (HSR/CARD) | ([40](#_ENREF_40)) |  |
|  | 5 years | Female | Consanguineous | - Hypoparathyroidism  - Addison's disease | c.232T>C/c.232T>C p.W78R/p.W78R (HSR/CARD) | ([15](#_ENREF_15)) |  |
|  | 4.6 years | Female | Consanguineous | - Hypoparathyroidism | c.232T>C/c.232T>C p.W78R/p.W78R (HSR/CARD) | ([15](#_ENREF_15)) |  |
|  | 1.1 years | Male | Consanguineous (1^st^ degree cousins) | - Mucosal candidiasis (1.1Y) - Hypoparathyroidism (4.5Y) - Addison disease (4.5Y) - Autoimmune thyroiditis - Insulin dependent diabetes mellitus | c.232T>C/c.1496delC p.W78R (HSR/CARD)/p.P499LfsX22 (downstream of PHD2) | ([41](#_ENREF_41)) |  |
|  | 1.2 years | Female | Consanguineous (1^st^ degree cousins) | - Mucosal candidiasis (1.2Y)  - Hypoparathyroidism  - Addison disease | c.232T>C/c.1496delC p.W78R (HSR/CARD)/p.P499LfsX22 (downstream of PHD2) | ([41](#_ENREF_41)) |  |
|  | 1 year | Male | Non-consanguineous | - Chronic candidiasis (1Y)  - Chronic hypoparathyroidism (5Y)  - Addison’s disease (5Y)  - Hashimoto’s thyroiditis  - Hypogonadism  - Juvenile rheumatoid arthritis | c.232T>C/c.1072C>T p.W78R (HSR/CARD)/p.Q358X (PRR) | ([36](#_ENREF_36)) |  |
|  | 8 years | Male | Non-consanguineous | - Chronic candidiasis (8Y) - Chronic hypoparathyroidism - Addison’s disease (8Y) | c.232T>C/c.1072C>T p.W78R (HSR/CARD)/p.Q358X (PRR) | ([36](#_ENREF_36)) |  |
|  | 0.1 year | Male | Non-consanguineous | - Chronic candidiasis (0.1Y)  - Chronic hypoparathyroidism (1Y)  - Addison’s disease (13Y)  - Alopecia  - Autoimmune hepatitis  - Hashimoto’s thyroiditis  - Vitiligo | c.232T>C/c.1072C>T p.W78R (HSR/CARD)/p.Q358X (PRR) | ([36](#_ENREF_36)) |  |
|  | N/A | N/A | N/A | - Hypoparathyroidism  - Addison’s disease  - Alopecia | c.232T>C/c.769C>T p.W78R (HSR/CARD)/p.R257X (SAND) | ([10](#_ENREF_10)) |  |
|  | 0.5 year | Male | Non-consanguineous | - Candidiasis  - Hypoparathyroidism  - Addison’s disease  - Alopecia  - Autoimmune hepatitis  - Cataract  - Enamel hypoplasia  - Hypothyroidism  - Tympanic calcifications  - Vitiligo | c.232T>C/c.1072C>T p.W78R (HSR/CARD)/p.Q358X (PRR) | ([37](#_ENREF_37)) |  |
|  | 0.5 year | Male | Non-consanguineous | - Candidiasis  - Hypoparathyroidism  - Addison’s disease  - Cataract  - Enamel hypoplasia  - Hypogonadism  - Hypothyroidism | c.232T>C/c.1072C>T p.W78R (HSR/CARD)/p.Q358X (PRR) | ([37](#_ENREF_37)) |  |
|  | 7 years | Male | Non-consanguineous | - Candidiasis  - Hypoparathyroidism  - Addison’s disease  - Enamel hypoplasia | c.232T>C/c.1072C>T p.W78R (HSR/CARD)/p.Q358X (PRR) | ([37](#_ENREF_37)) |  |
|  | 1 year | Female | Non-consanguineous | - Candidiasis  - Hypoparathyroidism  - Addison’s disease  - Hypothyroidism  - Alopecia  - Atrophic gastritis  - Autoimmune hepatitis  - Enamel hypoplasia  - Keratitis  - Malabsorption  - Pernicious anemia | c.232T>C/c.755C>T p.W78R (HSR/CARD)/p.P252L (SAND) | ([37](#_ENREF_37)) |  |
|  | N/A | N/A | N/A | N/A | c.232T>C/c.361delG p.W78R (HSR/CARD)/p.A121fs (NLS) | ([18](#_ENREF_18)) |  |
|  | N/A | N/A | N/A | N/A | c.232T>C/c.62C>T  p.W78R (HSR/CARD)/p.A21V (HSR/CARD) | ([18](#_ENREF_18)) |  |
|  | N/A | N/A | N/A | N/A | c.232T>C/c.1072C>T p.W78R (HSR/CARD)/p.Q358X (PRR) | ([18](#_ENREF_18)) |  |
|  | N/A | N/A | N/A | N/A | c.232T>C/c.1072C>T p.W78R (HSR/CARD)/p.Q358X (PRR) | ([18](#_ENREF_18)) |  |
|  | N/A | N/A | N/A | N/A | c.232T>C/c.1072C>T p.W78R (HSR/CARD)/p.Q358X (PRR) | ([18](#_ENREF_18)) |  |
|  | N/A | N/A | N/A | N/A | c.232T>C/c.755C>T p.W78R (HSR/CARD)/p.P252L (SAND) | ([18](#_ENREF_18)) |  |
|  | N/A | N/A | N/A | N/A | c.232T>C/c.64_69delGTGGAC p.W78R (HSR/CARD)/p.V22_D23del (HSR/CARD) | ([18](#_ENREF_18)) |  |
|  | N/A | N/A | N/A | N/A | c.232T>C/c.769C>T p.W78R (HSR/CARD)/p.R257X (SAND) | ([18](#_ENREF_18)) |  |
|  | N/A | N/A | N/A | N/A | c.232T>C/c.62C>T  p.W78R (HSR/CARD)/p.A21V (HSR/CARD) | ([18](#_ENREF_18)) |  |
|  | 4 years | N/A | N/A | - Chronic mucocutaneous candidiasis (4Y)  - Hypoparathyroidism (5.5Y)  - Primary adrenal insufficiency (9Y)  - Alopecia (18.1Y) | c.232T>C/c.769C>T p.W78R (HSR/CARD)/p.R257X (SAND) | ([6](#_ENREF_6)) |  |
|  | 4 years | N/A | N/A | - Chronic mucocutaneous candidiasis (4Y)  - Hypoparathyroidism (11.3Y)  - Primary adrenal insufficiency (11.3Y)  - Hypergonadotropic hypogonadism (18.5Y) | c.232T>C/c.769C>T p.W78R (HSR/CARD)/p.R257X (SAND) | ([6](#_ENREF_6)) |  |
|  | 4.5 years | N/A | N/A | - Chronic mucocutaneous candidiasis (14Y)  - Hypoparathyroidism (4.5Y)  - Primary adrenal insufficiency (23.2Y) | c.232T>C/c.769C>T p.W78R (HSR/CARD)/p.R257X (SAND) | ([6](#_ENREF_6)) |  |
|  | N/A | N/A | N/A | - Candidiasis  - Hypoparathyroidism  - Autoimmune gastritis  - Autoimmune hepatitis  - Encephalitis  - Hypogonadism  - Pernicious anemia | c.232T>C/IVS8 p.W78R (HSR/CARD)/E8del (PHD1) | ([19](#_ENREF_19)) |  |
|  | N/A | N/A | N/A | - Constipation | c.232T>C/c.755C>T p.W78R (HSR/CARD)/p.P252L (SAND) | ([40](#_ENREF_40)) |  |
|  | 2 years | Female | N/A | - Hypoparathyroidism (8Y)  - Pauciarticular juvenile rheumatoid arthritis (2Y) | c.232T>A/c.64_69delGTGGAC p.W78R (HSR/CARD)/p.V22_D23del (HSR/CARD) | ([42](#_ENREF_42)) |  |
|  | 1 year | Female | Consanguineous (1^st^ degree cousins) | - Mucocutaneous candidiasis (1Y)  - Hypoparathyroidism (5Y)  - Addison’s Disease (17Y)  - Alopecia  - Premature ovarian failure | c.239T>G/c.239T>G p.V80G/p.V80G (HSR/CARD) | ([43](#_ENREF_43)) |  |
|  | 2 years | Female | Non-consanguineous | - Mucocutaneous candidiasis (2Y)  - Hypoparathyroidism (4Y)  - Addison’s Disease (7Y) | c.239T>G/c.239T>G p.V80G/p.V80G (HSR/CARD) | ([43](#_ENREF_43)) |  |
|  | 9 years | Male | Non-consanguineous | - Chronic mucocutaneous candidiasis (9Y)  - Adrenal insufficiency (15Y)  - Autoimmune thyroiditis (15Y)  - Alopecia (15Y)  - Diabetes insipidus (20Y) | c.239T>G/CNV of 21q22.3 (chr21:45,670,150–45,706,528)*1 p.V80G (HSR/CARD)/duplication including E1-E2 | ([44](#_ENREF_44)) |  |
|  | N/A | N/A | Non-consanguineous | Presence of at least two of the following symptoms:  - Chronic mucocutaneous candidiasis  - Hypoparathyroidism  - Primary adrenocortical failure | c.254A>G/c.254A>G p.Y85C/p.Y85C (HSR/CARD) | ([45](#_ENREF_45)) |  |
|  | N/A | N/A | Non-consanguineous | Presence of at least two of the following symptoms:  - Chronic mucocutaneous candidiasis  - Hypoparathyroidism  - Primary adrenocortical failure | c.254A>G/c.254A>G p.Y85C/p.Y85C (HSR/CARD) | ([45](#_ENREF_45)) |  |
|  | N/A | N/A | Non-consanguineous | Presence of at least two of the following symptoms:  - Chronic mucocutaneous candidiasis  - Hypoparathyroidism  - Primary adrenocortical failure | c.254A>G/c.254A>G p.Y85C/p.Y85C (HSR/CARD) | ([45](#_ENREF_45)) |  |
|  | N/A | N/A | Non-consanguineous | Presence of at least two of the following symptoms:  - Chronic mucocutaneous candidiasis  - Hypoparathyroidism  - Primary adrenocortical failure | c.254A>G/c.254A>G p.Y85C/p.Y85C (HSR/CARD) | ([45](#_ENREF_45)) |  |
|  | N/A | N/A | Non-consanguineous | Presence of at least two of the following symptoms:  - Chronic mucocutaneous candidiasis  - Hypoparathyroidism  - Primary adrenocortical failure | c.254A>G/c.254A>G p.Y85C/p.Y85C (HSR/CARD) | ([45](#_ENREF_45)) |  |
|  | N/A | N/A | Non-consanguineous | Presence of at least two of the following symptoms:  - Chronic mucocutaneous candidiasis  - Hypoparathyroidism  - Primary adrenocortical failure | c.254A>G/c.254A>G p.Y85C/p.Y85C (HSR/CARD) | ([45](#_ENREF_45)) |  |
|  | N/A | N/A | Non-consanguineous | Presence of at least two of the following symptoms:  - Chronic mucocutaneous candidiasis  - Hypoparathyroidism  - Primary adrenocortical failure | c.254A>G/c.254A>G p.Y85C/p.Y85C (HSR/CARD) | ([45](#_ENREF_45)) |  |
|  | N/A | N/A | Non-consanguineous | Presence of at least two of the following symptoms:  - Chronic mucocutaneous candidiasis  - Hypoparathyroidism  - Primary adrenocortical failure | c.254A>G/c.254A>G p.Y85C/p.Y85C (HSR/CARD) | ([45](#_ENREF_45)) |  |
|  | N/A | N/A | Non-consanguineous | Presence of at least two of the following symptoms:  - Chronic mucocutaneous candidiasis  - Hypoparathyroidism  - Primary adrenocortical failure | c.254A>G/c.254A>G p.Y85C/p.Y85C (HSR/CARD) | ([45](#_ENREF_45)) |  |
|  | N/A | N/A | Non-consanguineous | Presence of at least two of the following symptoms:  - Chronic mucocutaneous candidiasis  - Hypoparathyroidism  - Primary adrenocortical failure | c.254A>G/c.254A>G p.Y85C/p.Y85C (HSR/CARD) | ([45](#_ENREF_45)) |  |
|  | N/A | N/A | Non-consanguineous | Presence of at least two of the following symptoms:  - Chronic mucocutaneous candidiasis  - Hypoparathyroidism  - Primary adrenocortical failure | c.254A>G/c.254A>G p.Y85C/p.Y85C (HSR/CARD) | ([45](#_ENREF_45)) |  |
|  | N/A | N/A | Non-consanguineous | Presence of at least two of the following symptoms:  - Chronic mucocutaneous candidiasis  - Hypoparathyroidism  - Primary adrenocortical failure | c.254A>G/c.254A>G p.Y85C/p.Y85C (HSR/CARD) | ([45](#_ENREF_45)) |  |
|  | N/A | N/A | Non-consanguineous | Presence of at least two of the following symptoms:  - Chronic mucocutaneous candidiasis  - Hypoparathyroidism  - Primary adrenocortical failure | c.254A>G/c.254A>G p.Y85C/p.Y85C (HSR/CARD) | ([45](#_ENREF_45)) |  |
|  | N/A | N/A | N/A | N/A | c.254A>G/c.254A>G p.Y85C/p.Y85C (HSR/CARD) | ([23](#_ENREF_23)) |  |
|  | N/A | N/A | N/A | N/A | c.254A>G/c.254A>G p.Y85C/p.Y85C (HSR/CARD) | ([23](#_ENREF_23)) |  |
|  | N/A | N/A | N/A | N/A | c.254A>G/c.254A>G p.Y85C/p.Y85C (HSR/CARD) | ([23](#_ENREF_23)) |  |
|  | N/A | N/A | N/A | N/A | c.254A>G/c.254A>G p.Y85C/p.Y85C (HSR/CARD) | ([23](#_ENREF_23)) |  |
|  | N/A | N/A | N/A | N/A | c.254A>G/c.254A>G p.Y85C/p.Y85C (HSR/CARD) | ([23](#_ENREF_23)) |  |
|  | 2 years | Male | Non-consanguineous | - Hypoparathyroidism (2Y) | c.254A>G/c.254A>G p.Y85C/p.Y85C (HSR/CARD) | ([46](#_ENREF_46)) |  |
|  | 2 years | Female | Non-consanguineous | - Hypoparathyroidism (2Y)  - Central diabetes insipidus (3Y) | c.254A>G/c.254A>G p.Y85C/p.Y85C (HSR/CARD) | ([46](#_ENREF_46)) |  |
|  | N/A | Male | N/A | - Mucocutaneous candidiasis - Hypoparathyroidism  - Alopecia  - Diabetes type 1 | c.254A>G/Unknown p.Y85C (HSR/CARD)/Unknown | ([25](#_ENREF_25)) |  |
|  | 1 year | Female | N/A | - Oral candidiasis (1Y)  - Hypoparathyroidism (1.6Y)  - Abdominal pain with chronic diarrhea and fat-soluble vitamin deficiencies (12Y)  - Alopecia (1.6Y)  - Autoimmune insulitis (1.6Y)  - Enamel hypoplasia (1.6Y)  - Hypogonadism (12Y)  - Onychodystrophy (1Y)  - Transient transaminitis and hepatomegaly (1Y)  - Vitiligo (1.6Y) | c.260T>C/c.967_979del13bp p.L87P (HSR/CARD)/p.L323fs (PHD1) | ([47](#_ENREF_47)) |  |
|  | 6.4 years | Male | N/A | - Mild oral candidiasis (6.4Y)  - Hypoparathyroidism (6.4Y)  - Adrenal insufficiency (13.9Y)  - Alopecia (6.4Y)  - Dermatitis rosaceiforme and severe acne (8.3Y)  - GADA-positive autoimmune insulitis without glucose impairment (8.3Y)  - Tetanic crise (8Y) | c.260T>C/c.967_979del13bp p.L87P (HSR/CARD)/p.L323fs (PHD1) | ([47](#_ENREF_47)) |  |
|  | 8 years | Female | Consanguineous (1^st^ degree cousins) | - Oral chronic mucocutaneous candidiasis - Hypoparathyroidism - Alopecia - Atrophic gastritis  - Ectodermal dysplasia - Ectodermal dystrophy of nails - Pernicious anemia - Retinal pigmentary changes (pigment epithelial atrophy, decreased visual acuity) | c.267_275del9/c.267_275del9 p.Y90-R92del/p.Y90-R92del (HSR/CARD) | ([15](#_ENREF_15), [48](#_ENREF_48)) |  |
|  | N/A | Male | Non-consanguineous | - Chronic mucocutaneous candidiasis  - Hypoparathyroidism | c.271A>G/c.769C>T (Mistakenly referred to as c.768C>T in the reference) p.Y90C (HSR/CARD)/p.R257X (SAND) | ([27](#_ENREF_27)) |  |
|  | N/A | Female | Non-consanguineous | - Chronic mucocutaneous candidiasis  - Hypoparathyroidism  - Fulminant hepatic failure | c.271A>G/c.769C>T (Mistakenly referred to as c.768C>T in the reference) p.Y90C (HSR/CARD)/p.R257X (SAND) | ([27](#_ENREF_27)) |  |
|  | 13 years | Male | N/A | - Mucocutaneous candidiasis  - Hypoparathyroidism  - Adrenal Insufficiency  - Anemia  - Carpopedal spasms  - Tubulo-interstitial nephritis (TIN)  - Vit B12 deficiency | c.274C>T/c.274C>T p.R92W/p.R92W (HSR/CARD) | ([49](#_ENREF_49)) |  |
|  | 4 years | Female | Non-consanguineous | - Chronic mucocutaneous candidiasis  - Hypoparathyroidism (4Y)  - Hypogonadism  - Nail dystrophy | c.274C>T/c.274C>T p.R92W/p.R92W (HSR/CARD) | ([50](#_ENREF_50)) |  |
|  | 3 years | Female | Non-consanguineous | - Chronic mucocutaneous candidiasis (3Y)  - Adrenal insufficiency  - Alopecia  - Anemia  - Autoimmune hepatitis - Nail dystrophy | c.274C>T/c.967_979del13bp p.R92W (HSR/CARD)/p.L323fs (PHD1) | ([50](#_ENREF_50)) |  |
|  | 1 year | Female | Consanguineous (1^st^ degree cousins) | - Mucosal candidiasis  - Hypoparathyroidism  - Adrenal insufficiency  - Nail dystrophy | c.308-1G>C/c.308-1G>C E3del/E3del (NLS) | ([41](#_ENREF_41)) |  |
|  | 1.5-5years | Male | N/A | - Hypoparathyroidism - Addison’s disease - Chronic active hepatitis (1Y)  - Chronic malabsorption  - Nephrocalcinosis  - Nephrolithiasis - Seizure - Tetany | c.328delC/c.328delC p.R110fs/pR110fs (NLS) | ([51](#_ENREF_51)) |  |
|  | 7 years | Female | Non-consanguineous | - Hypoparathyroidism  - Chronic atrophic gastritis  - Primary ovarian insufficiency  - Pure red cell aplasia | c.371C>T/c.623G>T p.P124L (NLS)/p.G208V (SAND) | ([33](#_ENREF_33)) |  |
|  | 2 years | Female | Consanguineous | - Chronic mucocutaneous candidiasis (14.7Y)  - Hypoparathyroidism (8Y)  - Primary adrenal insufficiency (14.7Y)  - Chronic allergic obstructive bronchopneumopathy (10.5Y)  - Dental enamel dysplasia (14.7Y)  - Hashimoto's thyroiditis (14.7Y)  - Onychomycosis (2Y)  - Secondary amenorrhea (14.6Y)  - Vitiligo (14Y) | c.396G>C/c.396G>C p.R132S/p.R132S (NLS) | ([52](#_ENREF_52)) |  |
|  | 5 years | Female | Non-consanguineous | - Candidiasis (11Y) - Hypoparathyroidism (5Y) - Addison’s disease (6Y) | c.415C>T/c.415C>T p.R139X/p.R139X (downstream of NLS) | ([43](#_ENREF_43)) |  |
|  | N/A | N/A | N/A | - Mucocutaneous candidiasis  - Hypoparathyroidism  - Addison’s disease  - Keratoconjunctivitis | c.415C>T/c.415C>T p.R139X/p.R139X (downstream of NLS) | ([10](#_ENREF_10)) |  |
|  | 12 years | N/A | N/A | - Hypoparathyroidism (12Y)  - Alopecia areata (14.5Y) | c.415C>T/c.415C>T p.R139X/p.R139X (downstream of NLS) | ([6](#_ENREF_6)) |  |
|  | 0.1 year | Female | Non-consanguineous | - Chronic candidiasis (0.1Y)  - Chronic hypoparathyroidism (6Y)  - Addison’s disease (8Y)  - Type 1 diabetes  - Epilepsy | c.415C>T/c.415C>T p.R139X/p.R139X (downstream of NLS) | ([36](#_ENREF_36)) |  |
|  | 6 years | Male | Consanguineous (1^st^ degree cousins) | - Mucocutaneous candidiasis (12Y)  - Hypoparathyroidism (14Y)  - Addison’s disease (6Y)  - Chronic diarrhea (10Y)  - Diabetes mellitus (10Y)  - Dry eyes (11Y)  - Hypothyroidism (10Y) | c.415C>T/c.415C>T p.R139X/p.R139X (downstream of NLS) | ([53](#_ENREF_53)) |  |
|  | N/A | Male | Consanguineous (1^st^ degree cousins) | - Addison’s disease  - Hypoparathyroidism | c.415C>T/c.415C>T p.R139X/p.R139X (downstream of NLS) | ([53](#_ENREF_53)) |  |
|  | 1 year | Male | Non-consanguineous | - Chronic mucocutaneous candidiasis (1Y)  - Hypoparathyroidism (3Y)  - Addison's disease (5Y)  - Keratitis  - Nail dystrophy | c.415C>T/c.415C>T p.R139X/p.R139X (downstream of NLS) | ([41](#_ENREF_41)) |  |
|  | 1-11 year(s) | Female | Non-consanguineous | Presence of at least two of the following symptoms:  - Chronic mucocutaneous candidiasis  - Hypoparathyroidism  - Adrenal failure  A single manifestation was sufficient for diagnosis in a sibling of an already established case. | c.415C>T/c.415C>T p.R139X/p.R139X (downstream of NLS) | ([54](#_ENREF_54)) |  |
|  | 1-11 year(s) | Female | Non-consanguineous | Presence of at least two of the following symptoms:  - Chronic mucocutaneous candidiasis  - Hypoparathyroidism  - Adrenal failure  A single manifestation was sufficient for diagnosis in a sibling of an already established case. | c.415C>T/c.415C>T p.R139X/p.R139X (downstream of NLS) | ([54](#_ENREF_54)) |  |
|  | 1-11 year(s) | Male | Non-consanguineous | Presence of at least two of the following symptoms:  - Chronic mucocutaneous candidiasis  - Hypoparathyroidism  - Adrenal failure  A single manifestation was sufficient for diagnosis in a sibling of an already established case. | c.415C>T/c.415C>T p.R139X/p.R139X (downstream of NLS) | ([54](#_ENREF_54)) |  |
|  | 1-11 year(s) | Male | Consanguineous (1^st^ degree cousins) | Presence of at least two of the following symptoms:  - Chronic mucocutaneous candidiasis  - Hypoparathyroidism  - Adrenal failure  A single manifestation was sufficient for diagnosis in a sibling of an already established case. | c.415C>T/c.415C>T p.R139X/p.R139X (downstream of NLS) | ([54](#_ENREF_54)) |  |
|  | 1-11 year(s) | Female | Consanguineous (1^st^ degree cousins) | Presence of at least two of the following symptoms:  - Chronic mucocutaneous candidiasis  - Hypoparathyroidism  - Adrenal failure  A single manifestation was sufficient for diagnosis in a sibling of an already established case. | c.415C>T/c.415C>T p.R139X/p.R139X (downstream of NLS) | ([54](#_ENREF_54)) |  |
|  | 1-11 year(s) | Female | Non-consanguineous | Presence of at least two of the following symptoms:  - Chronic mucocutaneous candidiasis  - Hypoparathyroidism  - Adrenal failure  A single manifestation was sufficient for diagnosis in a sibling of an already established case. | c.415C>T/c.415C>T p.R139X/p.R139X (downstream of NLS) | ([54](#_ENREF_54)) |  |
|  | 1-11 year(s) | Female | Non-consanguineous | Presence of at least two of the following symptoms:  - Chronic mucocutaneous candidiasis  - Hypoparathyroidism  - Adrenal failure  A single manifestation was sufficient for diagnosis in a sibling of an already established case. | c.415C>T/c.415C>T p.R139X/p.R139X (downstream of NLS) | ([54](#_ENREF_54)) |  |
|  | 1-11 year(s) | Male | Non-consanguineous | Presence of at least two of the following symptoms:  - Chronic mucocutaneous candidiasis  - Hypoparathyroidism  - Adrenal failure  A single manifestation was sufficient for diagnosis in a sibling of an already established case. | c.415C>T/c.415C>T p.R139X/p.R139X (downstream of NLS) | ([54](#_ENREF_54)) |  |
|  | 1-11 year(s) | Female | Non-consanguineous | Presence of at least two of the following symptoms:  - Chronic mucocutaneous candidiasis  - Hypoparathyroidism  - Adrenal failure  A single manifestation was sufficient for diagnosis in a sibling of an already established case. | c.415C>T/c.415C>T p.R139X/p.R139X (downstream of NLS) | ([54](#_ENREF_54)) |  |
|  | 1-11 year(s) | Female | Non-consanguineous | Presence of at least two of the following symptoms:  - Chronic mucocutaneous candidiasis  - Hypoparathyroidism  - Adrenal failure  A single manifestation was sufficient for diagnosis in a sibling of an already established case. | c.415C>T/c.415C>T p.R139X/p.R139X (downstream of NLS) | ([54](#_ENREF_54)) |  |
|  | 1-11 year(s) | Female | Non-consanguineous | Presence of at least two of the following symptoms:  - Chronic mucocutaneous candidiasis  - Hypoparathyroidism  - Adrenal failure  A single manifestation was sufficient for diagnosis in a sibling of an already established case. | c.415C>T/c.415C>T p.R139X/p.R139X (downstream of NLS) | ([54](#_ENREF_54)) |  |
|  | 1.1 years | Male | N/A | - Chronic mucocutaneous candidiasis (1.1Y) - Addison’s disease (4Y) - Autoimmune hepatitis (2.5Y) | c.415C>T/c.415C>T p.R139X/p.R139X (downstream of NLS) | ([55](#_ENREF_55)) |  |
|  | 2.5 years | Female | N/A | - Chronic mucocutaneous candidiasis (2.5Y) - Hypoparathyroidism (4.4Y) - Autoimmune hepatitis (2.5Y) | c.415C>T/c.415C>T p.R139X/p.R139X (downstream of NLS) | ([55](#_ENREF_55)) |  |
|  | 0.5 year | Female | Consanguineous | - Chronic mucocutaneous candidiasis - Hypoparathyroidism - Addison's disease | c.415C>T/c.62C>T p.R139X (downstream of NLS)/p.A21V (HSR/CARD) | ([41](#_ENREF_41)) |  |
|  | 1-11 year(s) | Male | Non-consanguineous | Presence of at least two of the following symptoms:  - Chronic mucocutaneous candidiasis  - Hypoparathyroidism  - Adrenal failure  A single manifestation was sufficient for diagnosis in a sibling of an already established case. | c.415C>T/Unknown p.R139X (downstream of NLS)/Unknown | ([54](#_ENREF_54)) |  |
|  | 1-11 year(s) | Female | Non-consanguineous | Presence of at least two of the following symptoms:  - Chronic mucocutaneous candidiasis  - Hypoparathyroidism  - Adrenal failure  A single manifestation was sufficient for diagnosis in a sibling of an already established case. | c.415C>T/c.967_979del13bp  p.R139X (downstream of NLS)/L323fs (PHD1) | ([54](#_ENREF_54)) |  |
|  | 2 years | Male | Non-consanguineous | - Mucocutaneous candidiasis (2Y) - Hypoparathyroidism (21Y)  - Adrenal insufficiency (7Y)  - Diabetes (36Y)  - Gonadal deﬁciency (15Y)  - Keratopathy: epithelial ulcerations, stromal opacities, deep neovascularization. (4Y) - Pernicious anemia (25Y) | c.415C>T/c.892G>T p.R139X (downstream of NLS)/p.E298K (upstream of PHD1) | ([24](#_ENREF_24)) |  |
|  | 18 years | Male | Consanguineous (3^rd^ degree cousins) | - Hypoparathyroidism (18Y)  - Chronic/tension headaches (18Y)  - Epilepsy (18Y)  - Keratopathy (19Y)  - Pernicious anemia (18Y)  - Type 1 diabetes mellitus (24Y) | c.463G>A/c.463G>A p.G155S/p.G155S (downstream of NLS) | ([56](#_ENREF_56)) |  |
|  | 1 year | Female | Consanguineous (3^rd^ degree cousins) | - Mucocutaneous candidiasis (1Y)  - Hypoparathyroidism (15Y)  - Epilepsy (15Y)  - Japanese encephalitis (7Y) | c.463G>A/c.463G>A p.G155S/p.G155S (downstream of NLS) | ([56](#_ENREF_56)) |  |
|  | 1 year | Female | Non-consanguineous | - Mucocutaneous candidiasis (2Y) - Hypocalcemia (4Y) - Addison disease (4Y)  - Anemia (4Y)  - Autoimmune hepatitis (2Y)  - Latent hypothyroidism (4Y) - Malabsorption (4Y)  - Monoarthritis (2Y)  - Nail mycosis (4Y)  - Pernicious anemia (8Y) - Recurrent episodes of fever and rash (1Y)  - Vitiligo (4Y) | c.463G>A/c.463G>A p.G155S/p.G155S (downstream of NLS) | ([57](#_ENREF_57)) |  |
|  | N/A | Female | N/A | - Hypoparathyroidism - Addison disease - Alopecia - Ovarian failure | c.463+2T>C/WT E3del (NLS)/WT | ([58](#_ENREF_58)) |  |
|  | 5 years | Female | N/A | - Chronic mucocutaneous candidiasis  - Hypoparathyroidism (5Y) | c.463+2T>C/c.463+2T>C E3del/E3del (NLS) | ([59](#_ENREF_59)) |  |
|  | 1 year | Male | N/A | - Chronic mucocutaneous candidiasis  - Hypoparathyroidism (1Y) | c.463+2T>C/c.463+2T>C E3del/E3del (NLS) | ([59](#_ENREF_59)) |  |
|  | 4 years | Male | N/A | - Chronic mucocutaneous candidiasis  - Hypoparathyroidism (4Y) - Adrenal insufficiency - Growth hormone deficiency | c.463+2T>C/c.967_979del13bp E3del (NLS)/p.L323fs (PHD1) | ([59](#_ENREF_59)) |  |
|  | 10 years | Female | Consanguineous (3^rd^ degree cousins) | - Candidiasis in ﬁnger and toenails (10Y)  - Chronic hypoparathyroidism (18Y)  - Addison’s disease (20Y)  - Autoimmune thyroiditis (22Y) | c.483_484insC/c.483_484insC  p.163fs/p.163fs (downstream of NLS) | ([60](#_ENREF_60)) |  |
|  | N/A | N/A | N/A | N/A | c.517C>T/IVS9-1G>A p.Q173X (downstream of NLS)/E9del (PHD1, PRR) | ([26](#_ENREF_26)) |  |
|  | N/A | N/A | Non-consanguineous | N/A | c.522_523ins13/c.967_979del13bp p.L175fs (downstream of NLS)/p.L323fs (PHD1) | ([61](#_ENREF_61)) |  |
|  | 5 years | Female | Consanguineous | - Chronic mucocutaneous candidiasis  - Hypoparathyroidism  - Alopecia  - Bronchiectasis  - Dental enamel hypoplasia  - Hypogonadism  - Intestinal dysfunction  - Keratopathy  - Nail dystrophy  - Nephropathy  - Pernicious anemia | c.560C>G/c.560C>G p.S187X/p.S187X (upstream of SAND) | ([50](#_ENREF_50)) |  |
|  | 4 years | Female | Non-consanguineous | - Chronic mucocutaneous candidiasis  - Hypoparathyroidism  - Alopecia  - Anemia  - Hypogonadism | c.560C>G/c.1072C>T  p.S187X (upstream of SAND)/p.Q358X (PRR) | ([50](#_ENREF_50)) |  |
|  | N/A | Male | N/A | - Candidiasis  - Autoimmune hepatitis - Chronic diarrhea - Recurrent pneumonia | c.607C>T/c.607C>T p.R203X/p.R203X (SAND) | ([62](#_ENREF_62)) |  |
|  | 1 year | Female | N/A | - Chronic mucocutaneous candidiasis (1Y)  - Chronic hypoparathyroidism (8Y)  - Addison’s disease (8Y)  - Atrophic gastritis (15Y)  - Enamel hypoplasia or ectodermal dystrophy (10Y)  - GI dysfunction (21Y)  - Hypergonadotropic hypogonadism (21Y)  - Nephrocalcinosis (17Y)  - Pernicious anemia (15Y) | c.607C>T/c.607C>T p.R203X/p.R203X (SAND) | ([38](#_ENREF_38)) |  |
|  | N/A | Female | Non-consanguineous | - Chronic hypoparathyroidism (10Y)  - Addison’s disease (22Y)  - Epilepsy  - Nephrocalcinosis  - Pernicious anemia  - Premature ovarian failure  - Vitiligo | c.607C>T/c.607C>T p.R203X/p.R203X (SAND) | ([20](#_ENREF_20), [36](#_ENREF_36)) |  |
|  | 8 years | Female | Consanguineous (1^st^ degree cousins) | - Chronic mucocutaneous candidiasis (24Y)  - Chronic hypoparathyroidism (8Y)  - Alopecia areata (11Y)  - Gastrointestinal dysfunction (25Y)  - Graves’ disease (25Y)  - Hypergonadotropic hypogonadism (14Y) | c.607C>T/c.607C>T p.R203X/p.R203X (SAND) | ([20](#_ENREF_20), [63](#_ENREF_63)) |  |
|  | 6 years | Female | Non-consanguineous | - Chronic mucocutaneous candidiasis (12Y)  - Chronic hypoparathyroidism (10Y)  - Addison’s disease (22Y)  - Autoimmune gastritis (12Y) - Enamel dysplasia (12Y)  - Gastrointestinal dysfunction (27Y)  - Hypergonadotropic hypogonadism (14Y)  - Pernicious anemia (12Y)  - Vitiligo (6Y) | c.607C>T/c.607C>T p.R203X/p.R203X (SAND) | ([63](#_ENREF_63)) |  |
|  | 1 year | Female | Non-consanguineous | - Chronic mucocutaneous candidiasis (1Y)  - Chronic hypoparathyroidism (5Y)  - Autoimmune hepatitis (30Y)  - Cervical arthrosis and spondyloarthropathy associated with the presence of small marginal osteophytes (33Y)  - Edentulous (18Y)  - Grand mal type seizures (5Y)  - Osteochondrosis dissecans (22Y) | c.607C>T/c.415C>T  p.R203X (SAND)/p.R139X (downstrem of NLS) | ([63](#_ENREF_63)) |  |
|  | 4 years | Male | Non-consanguineous | - Chronic mucocutaneous candidiasis (15Y)  - Chronic hypoparathyroidism (4Y)  - Addison’s disease (25Y)  - Autoimmune hepatitis (27Y) | c.607C>T/c.769C>T  p.R203X (SAND)/p.R257X (SAND) | ([20](#_ENREF_20), [63](#_ENREF_63)) |  |
|  | 1 year | Male | Non-consanguineous | - Chronic candidiasis (1Y)  - Hypoparathyroidism (4Y)  - Addison disease (17Y)  - Malabsorption (5Y)  - Nail dystrophy (15Y)  - Pernicious anemia (17Y)  - Severe obstructive lung disease with bronchiectasis (5Y) | c.607C>T/c.769C>T  p.R203X (SAND)/p.R257X (SAND) | ([20](#_ENREF_20), [64](#_ENREF_64)) |  |
|  | 0.7 year | Male | Non-consanguineous | - Chronic candidiasis (0.7Y)  - Hypoparathyroidism (11Y)  - Addison disease (14Y)  - Autoimmune gastritis (14Y)  - Nail dystrophy (12Y)  - Vitiligo (14Y) | c.607C>T/c.769C>T  p.R203X (SAND)/p.R257X (SAND) | ([64](#_ENREF_64)) |  |
|  | N/A | Male | N/A | N/A | c.607C>T/c.769C>T  p.R203X (SAND)/p.R257X (SAND) | ([65](#_ENREF_65)) |  |
|  | N/A | Male | N/A | N/A | c.607C>T/c.769C>T  p.R203X (SAND)/p.R257X (SAND) | ([65](#_ENREF_65)) |  |
|  | 4 years | Female | Non-consanguineous | - Chronic hypoparathyroidism (4Y) | c.607C>T/IVS9+5G>T  p.R203X (SAND)/E9del (PHD1, PRR) | ([39](#_ENREF_39)) |  |
|  | N/A | N/A | N/A | N/A | c.607C>T/Unknown  p.R203X (SAND)/Unknown | ([23](#_ENREF_23)) |  |
|  | 1 year | Male | Non-consanguineous | - Chronic candidiasis (1Y)  - Chronic hypoparathyroidism (4Y)  - Addison’s disease (17Y)  - Ectodermal dystrophy  - Hashimoto’s thyroiditis  - Malabsorption  - Obstructive lung disease  - Pernicious anemia | c.607C>T/c.769C>T  p.R203X (SAND)/p.R257X (SAND) | ([36](#_ENREF_36)) |  |
|  | 1 year | Male | Non-consanguineous | - Chronic candidiasis (1Y)  - Chronic hypoparathyroidism (11Y)  - Addison’s disease (12Y)  - Autoimmune gastritis  - Ectodermal dystrophy  - Vitiligo | c.607C>T/c.769C>T  p.R203X (SAND)/p.R257X (SAND) | ([36](#_ENREF_36)) |  |
|  | 12 years | Female | N/A | - Chronic mucocutaneous candidiasis  - Hypoparathyroidism  - Anemia | c.622G>T/Unknown p.G208W (SAND)/Unknown | ([33](#_ENREF_33), [66](#_ENREF_66)) |  |
|  | 5 years | Female | Non-consanguineous | - Mucocutaneous candidiasis  - Hypoparathyroidism (5Y)  - Enamel dysplasia  - High levels of antiparietal cell anti-bodies (APCA)  - Hypothyroid autoimmune thyroiditis (24Y) | c.682G >T/WT  p.G228W (SAND)/WT | ([67](#_ENREF_67)) |  |
|  | childhood | Male | Non-consanguineous | - Mucocutaneous candidiasis (childhood)  - High levels of antiglutamic acid de-carboxylase antibodies (anti-GAD)  - High levels of antiparietal cell anti-bodies (APCA)  - Hypothyroid autoimmune thyroiditis | c.682G>T/WT  p.G228W (SAND)/WT | ([67](#_ENREF_67)) |  |
|  | N/A | Female | Non-consanguineous | - Hypoparathyroidism  - High levels of antiglutamic acid de-carboxylase antibodies (anti-GAD)  - Hypothyroid autoimmune thyroiditis | c.682G>T/WT  p.G228W (SAND)/WT | ([67](#_ENREF_67)) |  |
|  | N/A | Male | Non-consanguineous | - Hypothyroid autoimmune thyroiditis | c.682G>T/WT  p.G228W (SAND)/WT | ([67](#_ENREF_67)) |  |
|  | N/A | Female | Non-consanguineous | - Hypothyroid autoimmune thyroiditis | c.682G>T/WT  p.G228W (SAND)/WT | ([67](#_ENREF_67)) |  |
|  | childhood | Female | Non-consanguineous | - Severe hypocalcemia (childhood) | c.682G>T/WT  p.G228W (SAND)/WT | ([67](#_ENREF_67)) |  |
|  | N/A | Female | Non-consanguineous | - High levels of antiglutamic acid de-carboxylase antibodies (anti-GAD)  - High levels of antiparietal cell anti-bodies (APCA)  - Hypothyroid autoimmune thyroiditis | c.682G>T/WT  p.G228W (SAND)/WT | ([67](#_ENREF_67)) |  |
|  | N/A | Male | N/A | - Mucocutaneous candidiasis  - Hypoparathyroidism  - Adrenal insufficiency  - Alopecia  - Vitiligo | c.769C>T/c.769C>T p.R257X/p.R257X (SAND) | ([68](#_ENREF_68)) |  |
|  | N/A | Male | N/A | - Mucocutaneous candidiasis  - Hypoparathyroidism  - Adrenal insufficiency  - Keratopathy | c.769C>T/c.769C>T p.R257X/p.R257X (SAND) | ([68](#_ENREF_68)) |  |
|  | 0.2 year | N/A | N/A | - Chronic mucocutaneous candidiasis (0.2Y)  - Hypoparathyroidism (8.2Y)  - Adrenal insufficiency (8.6Y)  - Alopecia (8Y) | c.769C>T/c.769C>T p.R257X/p.R257X (SAND) | ([6](#_ENREF_6)) |  |
|  | 5 years | N/A | N/A | - Chronic mucocutaneous candidiasis (5Y)  - Hypoparathyroidism (6.5Y)  - Adrenal insufficiency (7Y)  - Autoimmune hepatitis (6.5Y)  - Diabetes mellitus (13Y)  - Hypergonadotropic hypogonadism (19.5Y)  - Hypothyroidism (11.1Y)  - Malabsorption (6.5Y)  - Pernicious anemia (16.2Y) | c.769C>T/c.769C>T p.R257X/p.R257X (SAND) | ([6](#_ENREF_6)) |  |
|  | 0.1 year | N/A | N/A | - Hypoparathyroidism (5Y)  - Adrenal insufficiency (11.3Y)  - Alopecia (5Y)  - Malabsorption (5.5Y)  - Pernicious anemia (16.4Y)  - Retinitis pigmentosa (0.1Y) | c.769C>T/c.769C>T p.R257X/p.R257X (SAND) | ([6](#_ENREF_6)) |  |
|  | 9 years | N/A | N/A | - Chronic mucocutaneous candidiasis (9Y)  - Hypoparathyroidism (13.5Y)  - Adrenal insufficiency (12.3Y)  - Hypergonadotropic hypogonadism (15.6)  - Hypothyroidism (13.5Y) | c.769C>T/c.769C>T p.R257X/p.R257X (SAND) | ([6](#_ENREF_6)) |  |
|  | 11 years | N/A | N/A | - Chronic mucocutaneous candidiasis (15Y)  - Hypoparathyroidism (11.2Y) | c.769C>T/c.769C>T p.R257X/p.R257X (SAND) | ([6](#_ENREF_6)) |  |
|  | 4 years | N/A | N/A | - Hypoparathyroidism (8Y)  - Adrenal insufficiency (4Y) | c.769C>T/c.769C>T p.R257X/p.R257X (SAND) | ([6](#_ENREF_6)) |  |
|  | 1 year | N/A | N/A | - Chronic mucocutaneous candidiasis (1Y)  - Hypoparathyroidism (4.6Y)  - Adrenal insufficiency (6.3Y)  - Alopecia (5.2Y)  - Malabsorption (3.5Y) | c.769C>T/c.769C>T p.R257X/p.R257X (SAND) | ([6](#_ENREF_6)) |  |
|  | 2 years | N/A | N/A | - Chronic mucocutaneous candidiasis (2Y)  - Hypoparathyroidism (5.4Y)  - Adrenal insufficiency (8Y)  - Alopecia (7.5Y)  - Malabsorption (10Y)  - Metaphyseal dysplasia (10.5Y)  - Pure red cell aplasia (21.5Y) | c.769C>T/c.769C>T p.R257X/p.R257X (SAND) | ([6](#_ENREF_6)) |  |
|  | 4 years | N/A | N/A | - Chronic mucocutaneous candidiasis (4Y)  - Hypoparathyroidism (5.2Y)  - Adrenal insufficiency (5.2Y)  - Alopecia (10.2Y)  - Autoimmune hepatitis (5.5Y)  - Asplenism (16.3Y)  - Hair depigmentation (10.2Y)  - Hypergonadotropic hypogonadism (15Y)  - Malabsorption (4Y)  - Vitiligo (13.5Y) | c.769C>T/c.769C>T p.R257X/p.R257X (SAND) | ([6](#_ENREF_6)) |  |
|  | 5 years | N/A | N/A | - Chronic mucocutaneous candidiasis (5Y)  - Hypoparathyroidism (6.5Y)  - Adrenal insufficiency (8.1Y)  - Alopecia (5Y) | c.769C>T/c.769C>T p.R257X/p.R257X (SAND) | ([6](#_ENREF_6)) |  |
|  | 1 year | N/A | N/A | - Chronic mucocutaneous candidiasis (1Y)  - Hypoparathyroidism (8.3Y)  - Malabsorption (5Y) | c.769C>T/c.769C>T p.R257X/p.R257X (SAND) | ([6](#_ENREF_6)) |  |
|  | 1.6 years | N/A | N/A | - Chronic mucocutaneous candidiasis (1.6Y)  - Hypoparathyroidism (7Y)  - Adrenal insufficiency (7Y)  - Alopecia (10Y)  - Dry eye (10.5Y)  - Hypothyroidism (10.2Y)  - Keratitis (10.5Y) | c.769C>T/c.769C>T p.R257X/p.R257X (SAND) | ([6](#_ENREF_6)) |  |
|  | 3.3 years | N/A | N/A | - Chronic mucocutaneous candidiasis (3.3Y)  - Hypoparathyroidism (3.3Y)  - Adrenal insufficiency (3.3Y) | c.769C>T/c.769C>T p.R257X/p.R257X (SAND) | ([6](#_ENREF_6)) |  |
|  | 1.8 years | N/A | N/A | - Chronic mucocutaneous candidiasis (1.8Y)  - Hypoparathyroidism (10Y)  - Alopecia (14Y)  - Diabetes mellitus (9.5Y)  - Pernicious anemia (10Y) | c.769C>T/c.769C>T p.R257X/p.R257X (SAND) | ([6](#_ENREF_6)) |  |
|  | 10.2 years | N/A | N/A | - Chronic mucocutaneous candidiasis (10.2Y)  - Adrenal insufficiency (13.4Y) | c.769C>T/c.769C>T p.R257X/p.R257X (SAND) | ([6](#_ENREF_6)) |  |
|  | 4 years | N/A | N/A | - Hypoparathyroidism (11.4Y)  - Malabsorption (4Y)  - Vitiligo (19.2Y) | c.769C>T/c.769C>T p.R257X/p.R257X (SAND) | ([6](#_ENREF_6)) |  |
|  | 1 year | N/A | N/A | - Chronic mucocutaneous candidiasis (1Y)  - Autoimmune hepatitis (2Y)  - Diabetes mellitus (2Y) | c.769C>T/c.769C>T p.R257X/p.R257X (SAND) | ([6](#_ENREF_6)) |  |
|  | 3 years | N/A | N/A | - Chronic mucocutaneous candidiasis (3Y)  - Hypoparathyroidism (10Y)  - Alopecia (4Y)  - Diabetes mellitus (11.1Y)  - Malabsorption (6Y)  - Metaphyseal dysplasia (7Y)  - Pernicious anemia (10.2Y)  - Renal failure (14.3Y) | c.769C>T/c.769C>T p.R257X/p.R257X (SAND) | ([6](#_ENREF_6)) |  |
|  | 4 years | N/A | N/A | - Chronic mucocutaneous candidiasis (4Y)  - Alopecia (11Y) | c.769C>T/c.769C>T p.R257X/p.R257X (SAND) | ([6](#_ENREF_6)) |  |
|  | 0.3 year | N/A | N/A | - Chronic mucocutaneous candidiasis (0.3Y)  - Adrenal insufficiency (6.5Y)  - Autoimmune hepatitis (4Y) | c.769C>T/c.769C>T p.R257X/p.R257X (SAND) | ([6](#_ENREF_6)) |  |
|  | 0.3 year | N/A | N/A | - Chronic mucocutaneous candidiasis (0.3Y)  - Hypoparathyroidism (7.5Y) | c.769C>T/c.769C>T p.R257X/p.R257X (SAND) | ([6](#_ENREF_6)) |  |
|  | 0.1 year | N/A | N/A | - Hypoparathyroidism (7Y)  - Adrenal insufficiency (16.5Y)  - Retinitis pigmentosa (0.1Y) | c.769C>T/c.769C>T p.R257X/p.R257X (SAND) | ([6](#_ENREF_6)) |  |
|  | 0.1 year | Female | N/A | - Hypoparathyroidism (1Y)  - Adrenal insufficiency (15Y)  - Diabetes mellitus (14.8Y)  - Ovarian failure (15Y)  - Ptosis (0.1Y)  - Retinitis pigmentosa (1Y)  - Vitiligo (14Y) | c.769C>T/c.769C>T p.R257X/p.R257X (SAND) | ([6](#_ENREF_6)) |  |
|  | 2 years | N/A | N/A | - Chronic mucocutaneous candidiasis (5Y)  - Hypoparathyroidism (7Y)  - Adrenal insufficiency (3Y)  - Autoimmune hepatitis (2Y) | c.769C>T/c.769C>T p.R257X/p.R257X (SAND) | ([6](#_ENREF_6)) |  |
|  | N/A | Male | N/A | - Candidiasis | c.769C>T/c.769C>T p.R257X/p.R257X (SAND) | ([69](#_ENREF_69)) |  |
|  | N/A | Female | N/A | N/A | c.769C>T/c.769C>T p.R257X/p.R257X (SAND) | ([69](#_ENREF_69)) |  |
|  | N/A | N/A | N/A | N/A | c.769C>T/c.769C>T p.R257X/p.R257X (SAND) | ([69](#_ENREF_69)) |  |
|  | N/A | N/A | N/A | N/A | c.769C>T/c.769C>T p.R257X/p.R257X (SAND) | ([69](#_ENREF_69)) |  |
|  | N/A | N/A | N/A | N/A | c.769C>T/c.769C>T p.R257X/p.R257X (SAND) | ([69](#_ENREF_69)) |  |
|  | N/A | N/A | N/A | N/A | c.769C>T/c.769C>T p.R257X/p.R257X (SAND) | ([69](#_ENREF_69)) |  |
|  | 2 years | Male | Non-consanguineous | - Candidiasis (2Y)  - Hypoparathyroidism (20Y)  - Addison’s disease (15Y)  - Chronic otitis media  - Chronic sinusitis  - Enamel hypoplasia  - Gallstones  - Hyposplenia - Nasal polyps - Pitted nails  - Primary hypothyroidism  - Sicca syndrome | c.769C>T/c.769C>T p.R257X/p.R257X (SAND) | ([43](#_ENREF_43)) |  |
|  | 4 years | Male | Non-consanguineous | - Chronic candidiasis (4Y)  - Chronic hypoparathyroidism (5Y)  - Addison’s disease (12Y)  - Alopecia  - Autoimmune gastritis  - Autoimmune hepatitis - Keratopathy  - Malabsorption - Type 1 Diabetes  - Vitiligo | c.769C>T/c.769C>T p.R257X/p.R257X (SAND) | ([36](#_ENREF_36)) |  |
|  | 4 years | Male | Non-consanguineous | - Chronic candidiasis (4Y)  - Chronic hypoparathyroidism (6Y)  - Addison’s disease (12Y)  - Alopecia  - Keratopathy  - Malabsorption - Vitiligo | c.769C>T/c.769C>T p.R257X/p.R257X (SAND) | ([36](#_ENREF_36)) |  |
|  | 3 years | Male | Non-consanguineous | - Chronic candidiasis (4Y)  - Chronic hypoparathyroidism (3Y)  - Addison’s disease (10Y)  - Alopecia  - Keratopathy - Malabsorption - Vitiligo | c.769C>T/c.769C>T p.R257X/p.R257X (SAND) | ([36](#_ENREF_36)) |  |
|  | 7 years | Female | Non-consanguineous | - Chronic candidiasis (7Y)  - Chronic hypoparathyroidism (14Y)  - Addison’s disease (19Y)  - Autoimmune gastritis - Cancer of oral mucosa  - Pernicious anemia  - Premature ovarian failure - Sjogren’s syndrome - Vasculitis | c.769C>T/c.769C>T p.R257X/p.R257X (SAND) | ([36](#_ENREF_36)) |  |
|  | 2 years | Female | Non-consanguineous | - Chronic candidiasis (2Y)  - Chronic hypoparathyroidism (6Y)  - Addison’s disease (9Y)  - Alopecia - Autoimmune hepatitis - Graves’ disease - Keratopathy - Pernicious anemia  - Premature ovarian failure  - Sjogren’s syndrome | c.769C>T/c.769C>T p.R257X/p.R257X (SAND) | ([36](#_ENREF_36)) |  |
|  | 6 years | Male | Non-consanguineous | - Chronic candidiasis (6Y)  - Chronic hypoparathyroidism (14Y)  - Addison’s disease (9Y)  - Alopecia  - Autoimmune hepatitis  - Keratopathy | c.769C>T/c.769C>T p.R257X/p.R257X (SAND) | ([36](#_ENREF_36)) |  |
|  | 5 years | Male | Non-consanguineous | - Chronic candidiasis (1Y)  - Chronic hypoparathyroidism (5Y)  - Addison’s disease (8Y)  - Hashimoto’s thyroiditis | c.769C>T/c.769C>T p.R257X/p.R257X (SAND) | ([36](#_ENREF_36)) |  |
|  | 3 years | Female | Consanguineous | - Oral chronic mucocutaneous candidiasis (4Y)  - Hypoparathyroidism (3Y) - Adrenal insufficiency (23Y)  - Asthma (10Y)  - Bilateral endothelial condensations on the cornea  - Bilateral forearm panniculitis (lupus-like) (31Y) - Chronic hepatitis B (13Y)  - Type 1 diabetes (18Y) | c.769C>T/c.769C>T  p.R257X/p.R257X (SAND) | ([70](#_ENREF_70)) |  |
|  | N/A | Female | Consanguineous | - Adrenal failure (20Y) - Type 1 diabetes (17Y) | c.769C>T/c.769C>T  p.R257X/p.R257X (SAND) | ([70](#_ENREF_70)) |  |
|  | N/A | Female | Consanguineous | - Adrenal failure  - Hypoparathyroidism | Unknown  Sibling of Pts. 274 and 275  Probably harbors the same mutation | ([70](#_ENREF_70)) |  |
|  | 2 years | Female | Consanguineous | - Chronic oral candidiasis (2Y)  - Hypoparathyroidism (4Y)  - Primary adrenocortical insufficiency (8Y)  - Cyst on ovary (22Y)  - Primary pulmonary hypertension (30Y) | c.769C>T/c.769C>T p.R257X/p.R257X (SAND) | ([71](#_ENREF_71)) |  |
|  | 1.3 years | Male | Non-consanguineous | - Oral candidiasis (4Y) - Adrenal insufficiency (4.5Y)  - Arthritis (2Y) - Autoimmune hepatitis (4Y) - Diarrhea (1.3Y)  - Hepatic cirrhosis Child-Pugh class C (4Y)  - Lipodystrophy (1.3Y) | c.769C>T/c.769C>T p.R257X/p.R257X (SAND) | ([72](#_ENREF_72)) |  |
|  | 3.5 years | Male | N/A | - Alopecia totalis (4Y)  - Dental enamel hypoplasia (3.5Y)  - Nail dystrophy (3.5Y) - Nail pitting (3.5Y) | c.769C>T/c.769C>T p.R257X/p.R257X (SAND) | ([73](#_ENREF_73)) |  |
|  | 1.5 years | Female | N/A | - Oral and nail candidiasis (1.5Y)  - Hypoparathyroidism - Addison’s disease  - Autoimmune hepatitis (4Y)  - Iron deficiency anemia (5Y) | c.769C>T/c.769C>T p.R257X/p.R257X (SAND) | ([73](#_ENREF_73)) |  |
|  | 0.1 year | Male | N/A | - Hypoparathyroidism (0.1Y) | c.769C>T/c.769C>T p.R257X/p.R257X (SAND) | ([73](#_ENREF_73)) |  |
|  | N/A | Female | N/A | - Severe chronic mucocutaneous candidiasis - Parathyroid insufficiency  - Adrenal failure | Unknown  Sibling of Pt. 281  Probably harbors the same mutation | ([73](#_ENREF_73)) |  |
|  | 1.5 years | Female | N/A | - Oral and vulvovaginal candidiasis (Until 3Y)  - Hypoparathyroidism  - Mixed-type hearing impairment (5Y)  - Recurrent otitis media (18M-5Y) | c.769C>T/c.769C>T p.R257X/p.R257X (SAND) | ([73](#_ENREF_73)) |  |
|  | N/A | Female | N/A | - Mucocutaneous candidiasis  - Hypoparathyroidism  - Addison’s disease  - Chronic active hepatitis | c.769C>T/c.769C>T p.R257X/p.R257X (SAND) | ([74](#_ENREF_74)) |  |
|  | N/A | Female | N/A | - Mucocutaneous candidiasis - Hypoparathyroidism  - Addison’s disease  - Alopecia - Cholelithiasis  - Keratoconjunctivitis | c.769C>T/c.769C>T p.R257X/p.R257X (SAND) | ([74](#_ENREF_74)) |  |
|  | N/A | Male | N/A | - Candidiasis  - Adrenal insufficiency  - Chronic diarrhea | c.769C>T/c.769C>T p.R257X/p.R257X (SAND) | ([62](#_ENREF_62)) |  |
|  | 1.5-5 years | Female | N/A | - Hypoparathyroidism - Addison’s disease  - Chronic active hepatitis (8Y)  - Chronic malabsorption  - Hypogonadism | c.769C>T/c.769C>T p.R257X/p.R257X (SAND) | ([51](#_ENREF_51)) |  |
|  | 1.5-5 years | Male | N/A | - Hypoparathyroidism  - Chronic malabsorption  - Vitiligo | c.769C>T/c.769C>T p.R257X/p.R257X (SAND) | ([51](#_ENREF_51)) |  |
|  | 2 years | Female | Non-consanguineous | - Chronic hypoparathyroidism (2Y)  - Hypergonadotropic hypogonadism | c.769C>T/c.769C>T p.R257X/p.R257X (SAND) | ([39](#_ENREF_39)) |  |
|  | N/A | N/A | N/A | N/A | c.769C>T/c.769C>T p.R257X/p.R257X (SAND) | ([23](#_ENREF_23)) |  |
|  | N/A | N/A | N/A | N/A | c.769C>T/c.769C>T p.R257X/p.R257X (SAND) | ([23](#_ENREF_23)) |  |
|  | N/A | N/A | N/A | N/A | c.769C>T/c.769C>T p.R257X/p.R257X (SAND) | ([23](#_ENREF_23)) |  |
|  | N/A | N/A | N/A | N/A | c.769C>T/c.769C>T p.R257X/p.R257X (SAND) | ([23](#_ENREF_23)) |  |
|  | N/A | N/A | N/A | N/A | c.769C>T/c.769C>T p.R257X/p.R257X (SAND) | ([23](#_ENREF_23)) |  |
|  | N/A | N/A | N/A | N/A | c.769C>T/c.769C>T p.R257X/p.R257X (SAND) | ([23](#_ENREF_23)) |  |
|  | N/A | N/A | N/A | N/A | c.769C>T/c.769C>T p.R257X/p.R257X (SAND) | ([23](#_ENREF_23)) |  |
|  | N/A | N/A | N/A | N/A | c.769C>T/c.769C>T p.R257X/p.R257X (SAND) | ([23](#_ENREF_23)) |  |
|  | N/A | N/A | N/A | N/A | c.769C>T/c.769C>T p.R257X/p.R257X (SAND) | ([23](#_ENREF_23)) |  |
|  | N/A | N/A | N/A | N/A | c.769C>T/c.769C>T p.R257X/p.R257X (SAND) | ([23](#_ENREF_23)) |  |
|  | N/A | N/A | N/A | N/A | c.769C>T/c.769C>T p.R257X/p.R257X (SAND) | ([23](#_ENREF_23)) |  |
|  | N/A | N/A | N/A | N/A | c.769C>T/c.769C>T p.R257X/p.R257X (SAND) | ([23](#_ENREF_23)) |  |
|  | N/A | N/A | N/A | N/A | c.769C>T/c.769C>T p.R257X/p.R257X (SAND) | ([23](#_ENREF_23)) |  |
|  | N/A | N/A | N/A | N/A | c.769C>T/c.769C>T p.R257X/p.R257X (SAND) | ([23](#_ENREF_23)) |  |
|  | N/A | N/A | N/A | N/A | c.769C>T/c.769C>T p.R257X/p.R257X (SAND) | ([23](#_ENREF_23)) |  |
|  | N/A | N/A | N/A | N/A | c.769C>T/c.769C>T p.R257X/p.R257X (SAND) | ([23](#_ENREF_23)) |  |
|  | N/A | N/A | N/A | N/A | c.769C>T/c.769C>T p.R257X/p.R257X (SAND) | ([23](#_ENREF_23)) |  |
|  | N/A | N/A | N/A | N/A | c.769C>T/c.769C>T p.R257X/p.R257X (SAND) | ([23](#_ENREF_23)) |  |
|  | N/A | N/A | N/A | N/A | c.769C>T/c.769C>T p.R257X/p.R257X (SAND) | ([23](#_ENREF_23)) |  |
|  | N/A | N/A | N/A | N/A | c.769C>T/c.769C>T p.R257X/p.R257X (SAND) | ([23](#_ENREF_23)) |  |
|  | N/A | N/A | N/A | N/A | c.769C>T/c.769C>T p.R257X/p.R257X (SAND) | ([23](#_ENREF_23)) |  |
|  | N/A | N/A | N/A | N/A | c.769C>T/c.769C>T p.R257X/p.R257X (SAND) | ([23](#_ENREF_23)) |  |
|  | N/A | N/A | N/A | N/A | c.769C>T/c.769C>T p.R257X/p.R257X (SAND) | ([23](#_ENREF_23)) |  |
|  | N/A | N/A | N/A | N/A | c.769C>T/c.769C>T p.R257X/p.R257X (SAND) | ([23](#_ENREF_23)) |  |
|  | N/A | N/A | N/A | N/A | c.769C>T/c.769C>T p.R257X/p.R257X (SAND) | ([23](#_ENREF_23)) |  |
|  | N/A | N/A | N/A | N/A | c.769C>T/c.769C>T p.R257X/p.R257X (SAND) | ([23](#_ENREF_23)) |  |
|  | N/A | N/A | N/A | N/A | c.769C>T/c.769C>T p.R257X/p.R257X (SAND) | ([23](#_ENREF_23)) |  |
|  | N/A | N/A | N/A | N/A | c.769C>T/c.769C>T p.R257X/p.R257X (SAND) | ([23](#_ENREF_23)) |  |
|  | N/A | N/A | N/A | N/A | c.769C>T/c.769C>T p.R257X/p.R257X (SAND) | ([23](#_ENREF_23)) |  |
|  | N/A | N/A | N/A | N/A | c.769C>T/c.769C>T p.R257X/p.R257X (SAND) | ([23](#_ENREF_23)) |  |
|  | N/A | N/A | N/A | N/A | c.769C>T/c.769C>T p.R257X/p.R257X (SAND) | ([23](#_ENREF_23)) |  |
|  | N/A | N/A | N/A | N/A | c.769C>T/c.769C>T p.R257X/p.R257X (SAND) | ([23](#_ENREF_23)) |  |
|  | N/A | N/A | N/A | N/A | c.769C>T/c.769C>T p.R257X/p.R257X (SAND) | ([23](#_ENREF_23)) |  |
|  | N/A | N/A | N/A | N/A | c.769C>T/c.769C>T p.R257X/p.R257X (SAND) | ([23](#_ENREF_23)) |  |
|  | N/A | N/A | N/A | N/A | c.769C>T/c.769C>T p.R257X/p.R257X (SAND) | ([23](#_ENREF_23)) |  |
|  | N/A | N/A | N/A | N/A | c.769C>T/c.769C>T p.R257X/p.R257X (SAND) | ([23](#_ENREF_23)) |  |
|  | N/A | N/A | N/A | N/A | c.769C>T/c.769C>T p.R257X/p.R257X (SAND) | ([23](#_ENREF_23)) |  |
|  | N/A | N/A | N/A | N/A | c.769C>T/c.769C>T p.R257X/p.R257X (SAND) | ([23](#_ENREF_23)) |  |
|  | N/A | N/A | N/A | N/A | c.769C>T/c.769C>T p.R257X/p.R257X (SAND) | ([23](#_ENREF_23)) |  |
|  | N/A | N/A | N/A | N/A | c.769C>T/c.769C>T p.R257X/p.R257X (SAND) | ([23](#_ENREF_23)) |  |
|  | N/A | N/A | N/A | N/A | c.769C>T/c.769C>T p.R257X/p.R257X (SAND) | ([23](#_ENREF_23)) |  |
|  | N/A | N/A | N/A | N/A | c.769C>T/c.769C>T p.R257X/p.R257X (SAND) | ([23](#_ENREF_23)) |  |
|  | N/A | N/A | N/A | N/A | c.769C>T/c.769C>T p.R257X/p.R257X (SAND) | ([23](#_ENREF_23)) |  |
|  | N/A | N/A | N/A | N/A | c.769C>T/c.769C>T p.R257X/p.R257X (SAND) | ([23](#_ENREF_23)) |  |
|  | N/A | N/A | N/A | N/A | c.769C>T/c.769C>T p.R257X/p.R257X (SAND) | ([23](#_ENREF_23)) |  |
|  | N/A | N/A | N/A | N/A | c.769C>T/c.769C>T p.R257X/p.R257X (SAND) | ([23](#_ENREF_23)) |  |
|  | N/A | N/A | N/A | N/A | c.769C>T/c.769C>T p.R257X/p.R257X (SAND) | ([23](#_ENREF_23)) |  |
|  | N/A | N/A | N/A | N/A | c.769C>T/c.769C>T p.R257X/p.R257X (SAND) | ([23](#_ENREF_23)) |  |
|  | N/A | N/A | N/A | N/A | c.769C>T/c.769C>T p.R257X/p.R257X (SAND) | ([23](#_ENREF_23)) |  |
|  | N/A | N/A | N/A | N/A | c.769C>T/c.769C>T p.R257X/p.R257X (SAND) | ([23](#_ENREF_23)) |  |
|  | N/A | N/A | N/A | N/A | c.769C>T/c.769C>T p.R257X/p.R257X (SAND) | ([23](#_ENREF_23)) |  |
|  | N/A | N/A | N/A | N/A | c.769C>T/c.769C>T p.R257X/p.R257X (SAND) | ([23](#_ENREF_23)) |  |
|  | 8 years | Female | Non-consanguineous | - Chronic mucocutaneous candidiasis (8Y)  - Hypoparathyroidism (24Y)  - Enamel hypoplasia (17Y) - Nail dystrophy (17Y)  - Vitiligo (14Y) | c.769C>T/c.769C>T p.R257X/p.R257X (SAND) | ([15](#_ENREF_15), [75](#_ENREF_75)) |  |
|  | N/A | N/A | Non-consanguineous | Presence of at least two of the following symptoms:  - Chronic mucocutaneous candidiasis  - Hypoparathyroidism  - Primary adrenocortical failure | c.769C>T/c.769C>T p.R257X/p.R257X (SAND) | ([45](#_ENREF_45)) |  |
|  | N/A | N/A | Non-consanguineous | Presence of at least two of the following symptoms:  - Chronic mucocutaneous candidiasis  - Hypoparathyroidism  - Primary adrenocortical failure | c.769C>T/c.769C>T p.R257X/p.R257X (SAND) | ([45](#_ENREF_45)) |  |
|  | N/A | N/A | Non-consanguineous | Presence of at least two of the following symptoms:  - Chronic mucocutaneous candidiasis  - Hypoparathyroidism  - Primary adrenocortical failure | c.769C>T/c.769C>T p.R257X/p.R257X (SAND) | ([45](#_ENREF_45)) |  |
|  | N/A | N/A | Non-consanguineous | Presence of at least two of the following symptoms:  - Chronic mucocutaneous candidiasis  - Hypoparathyroidism  - Primary adrenocortical failure | c.769C>T/c.769C>T p.R257X/p.R257X (SAND) | ([45](#_ENREF_45)) |  |
|  | N/A | N/A | Non-consanguineous | Presence of at least two of the following symptoms:  - Chronic mucocutaneous candidiasis  - Hypoparathyroidism  - Primary adrenocortical failure | c.769C>T/c.769C>T p.R257X/p.R257X (SAND) | ([45](#_ENREF_45)) |  |
|  | N/A | N/A | Non-consanguineous | Presence of at least two of the following symptoms:  - Chronic mucocutaneous candidiasis  - Hypoparathyroidism  - Primary adrenocortical failure | c.769C>T/c.769C>T p.R257X/p.R257X (SAND) | ([45](#_ENREF_45)) |  |
|  | N/A | N/A | Non-consanguineous | Presence of at least two of the following symptoms:  - Chronic mucocutaneous candidiasis  - Hypoparathyroidism  - Primary adrenocortical failure | c.769C>T/c.769C>T p.R257X/p.R257X (SAND) | ([45](#_ENREF_45)) |  |
|  | N/A | N/A | Non-consanguineous | Presence of at least two of the following symptoms:  - Chronic mucocutaneous candidiasis  - Hypoparathyroidism  - Primary adrenocortical failure | c.769C>T/c.769C>T p.R257X/p.R257X (SAND) | ([45](#_ENREF_45)) |  |
|  | N/A | N/A | Non-consanguineous | Presence of at least two of the following symptoms:  - Chronic mucocutaneous candidiasis  - Hypoparathyroidism  - Primary adrenocortical failure | c.769C>T/c.769C>T p.R257X/p.R257X (SAND) | ([45](#_ENREF_45)) |  |
|  | N/A | N/A | Non-consanguineous | Presence of at least two of the following symptoms:  - Chronic mucocutaneous candidiasis  - Hypoparathyroidism  - Primary adrenocortical failure | c.769C>T/c.769C>T p.R257X/p.R257X (SAND) | ([45](#_ENREF_45)) |  |
|  | N/A | N/A | Non-consanguineous | Presence of at least two of the following symptoms:  - Chronic mucocutaneous candidiasis  - Hypoparathyroidism  - Primary adrenocortical failure | c.769C>T/c.769C>T p.R257X/p.R257X (SAND) | ([45](#_ENREF_45)) |  |
|  | N/A | N/A | Non-consanguineous | Presence of at least two of the following symptoms:  - Chronic mucocutaneous candidiasis  - Hypoparathyroidism  - Primary adrenocortical failure | c.769C>T/c.769C>T p.R257X/p.R257X (SAND) | ([45](#_ENREF_45)) |  |
|  | N/A | N/A | Non-consanguineous | Presence of at least two of the following symptoms:  - Chronic mucocutaneous candidiasis  - Hypoparathyroidism  - Primary adrenocortical failure | c.769C>T/c.769C>T p.R257X/p.R257X (SAND) | ([45](#_ENREF_45)) |  |
|  | N/A | N/A | Non-consanguineous | Presence of at least two of the following symptoms:  - Chronic mucocutaneous candidiasis  - Hypoparathyroidism  - Primary adrenocortical failure | c.769C>T/c.769C>T p.R257X/p.R257X (SAND) | ([45](#_ENREF_45)) |  |
|  | N/A | N/A | Non-consanguineous | Presence of at least two of the following symptoms:  - Chronic mucocutaneous candidiasis  - Hypoparathyroidism  - Primary adrenocortical failure | c.769C>T/c.769C>T p.R257X/p.R257X (SAND) | ([45](#_ENREF_45)) |  |
|  | N/A | N/A | Non-consanguineous | Presence of at least two of the following symptoms:  - Chronic mucocutaneous candidiasis  - Hypoparathyroidism  - Primary adrenocortical failure | c.769C>T/c.769C>T p.R257X/p.R257X (SAND) | ([45](#_ENREF_45)) |  |
|  | N/A | N/A | Non-consanguineous | Presence of at least two of the following symptoms:  - Chronic mucocutaneous candidiasis  - Hypoparathyroidism  - Primary adrenocortical failure | c.769C>T/c.769C>T p.R257X/p.R257X (SAND) | ([45](#_ENREF_45)) |  |
|  | N/A | N/A | Non-consanguineous | Presence of at least two of the following symptoms:  - Chronic mucocutaneous candidiasis  - Hypoparathyroidism  - Primary adrenocortical failure | c.769C>T/c.769C>T p.R257X/p.R257X (SAND) | ([45](#_ENREF_45)) |  |
|  | N/A | N/A | Non-consanguineous | Presence of at least two of the following symptoms:  - Chronic mucocutaneous candidiasis  - Hypoparathyroidism  - Primary adrenocortical failure | c.769C>T/c.769C>T p.R257X/p.R257X (SAND) | ([45](#_ENREF_45)) |  |
|  | N/A | N/A | Non-consanguineous | Presence of at least two of the following symptoms:  - Chronic mucocutaneous candidiasis  - Hypoparathyroidism  - Primary adrenocortical failure | c.769C>T/c.769C>T p.R257X/p.R257X (SAND) | ([45](#_ENREF_45)) |  |
|  | N/A | N/A | Non-consanguineous | Presence of at least two of the following symptoms:  - Chronic mucocutaneous candidiasis  - Hypoparathyroidism  - Primary adrenocortical failure | c.769C>T/c.769C>T p.R257X/p.R257X (SAND) | ([45](#_ENREF_45)) |  |
|  | N/A | N/A | Non-consanguineous | Presence of at least two of the following symptoms:  - Chronic mucocutaneous candidiasis  - Hypoparathyroidism  - Primary adrenocortical failure | c.769C>T/c.769C>T p.R257X/p.R257X (SAND) | ([45](#_ENREF_45)) |  |
|  | N/A | N/A | Non-consanguineous | Presence of at least two of the following symptoms:  - Chronic mucocutaneous candidiasis  - Hypoparathyroidism  - Primary adrenocortical failure | c.769C>T/c.769C>T p.R257X/p.R257X (SAND) | ([45](#_ENREF_45)) |  |
|  | N/A | N/A | Non-consanguineous | Presence of at least two of the following symptoms:  - Chronic mucocutaneous candidiasis  - Hypoparathyroidism  - Primary adrenocortical failure | c.769C>T/c.769C>T p.R257X/p.R257X (SAND) | ([45](#_ENREF_45)) |  |
|  | N/A | N/A | Non-consanguineous | Presence of at least two of the following symptoms:  - Chronic mucocutaneous candidiasis  - Hypoparathyroidism  - Primary adrenocortical failure | c.769C>T/c.769C>T p.R257X/p.R257X (SAND) | ([45](#_ENREF_45)) |  |
|  | N/A | N/A | Non-consanguineous | Presence of at least two of the following symptoms:  - Chronic mucocutaneous candidiasis  - Hypoparathyroidism  - Primary adrenocortical failure | c.769C>T/c.769C>T p.R257X/p.R257X (SAND) | ([45](#_ENREF_45)) |  |
|  | N/A | N/A | Non-consanguineous | Presence of at least two of the following symptoms:  - Chronic mucocutaneous candidiasis  - Hypoparathyroidism  - Primary adrenocortical failure | c.769C>T/c.769C>T p.R257X/p.R257X (SAND) | ([45](#_ENREF_45)) |  |
|  | N/A | N/A | Non-consanguineous | Presence of at least two of the following symptoms:  - Chronic mucocutaneous candidiasis  - Hypoparathyroidism  - Primary adrenocortical failure | c.769C>T/c.769C>T p.R257X/p.R257X (SAND) | ([45](#_ENREF_45)) |  |
|  | N/A | N/A | Non-consanguineous | Presence of at least two of the following symptoms:  - Chronic mucocutaneous candidiasis  - Hypoparathyroidism  - Primary adrenocortical failure | c.769C>T/c.769C>T p.R257X/p.R257X (SAND) | ([45](#_ENREF_45)) |  |
|  | N/A | N/A | Non-consanguineous | Presence of at least two of the following symptoms:  - Chronic mucocutaneous candidiasis  - Hypoparathyroidism  - Primary adrenocortical failure | c.769C>T/c.769C>T p.R257X/p.R257X (SAND) | ([45](#_ENREF_45)) |  |
|  | N/A | N/A | Non-consanguineous | Presence of at least two of the following symptoms:  - Chronic mucocutaneous candidiasis  - Hypoparathyroidism  - Primary adrenocortical failure | c.769C>T/c.769C>T p.R257X/p.R257X (SAND) | ([45](#_ENREF_45)) |  |
|  | N/A | N/A | Non-consanguineous | Presence of at least two of the following symptoms:  - Chronic mucocutaneous candidiasis  - Hypoparathyroidism  - Primary adrenocortical failure | c.769C>T/c.769C>T p.R257X/p.R257X (SAND) | ([45](#_ENREF_45)) |  |
|  | N/A | N/A | Non-consanguineous | Presence of at least two of the following symptoms:  - Chronic mucocutaneous candidiasis  - Hypoparathyroidism  - Primary adrenocortical failure | c.769C>T/c.769C>T p.R257X/p.R257X (SAND) | ([45](#_ENREF_45)) |  |
|  | N/A | N/A | Non-consanguineous | Presence of at least two of the following symptoms:  - Chronic mucocutaneous candidiasis  - Hypoparathyroidism  - Primary adrenocortical failure | c.769C>T/c.769C>T p.R257X/p.R257X (SAND) | ([45](#_ENREF_45)) |  |
|  | N/A | N/A | Non-consanguineous | Presence of at least two of the following symptoms:  - Chronic mucocutaneous candidiasis  - Hypoparathyroidism  - Primary adrenocortical failure | c.769C>T/c.769C>T p.R257X/p.R257X (SAND) | ([45](#_ENREF_45)) |  |
|  | N/A | N/A | Non-consanguineous | Presence of at least two of the following symptoms:  - Chronic mucocutaneous candidiasis  - Hypoparathyroidism  - Primary adrenocortical failure | c.769C>T/c.769C>T p.R257X/p.R257X (SAND) | ([45](#_ENREF_45)) |  |
|  | N/A | N/A | Non-consanguineous | Presence of at least two of the following symptoms:  - Chronic mucocutaneous candidiasis  - Hypoparathyroidism  - Primary adrenocortical failure | c.769C>T/c.769C>T p.R257X/p.R257X (SAND) | ([45](#_ENREF_45)) |  |
|  | N/A | N/A | Non-consanguineous | Presence of at least two of the following symptoms:  - Chronic mucocutaneous candidiasis  - Hypoparathyroidism  - Primary adrenocortical failure | c.769C>T/c.769C>T p.R257X/p.R257X (SAND) | ([45](#_ENREF_45)) |  |
|  | N/A | N/A | Non-consanguineous | Presence of at least two of the following symptoms:  - Chronic mucocutaneous candidiasis  - Hypoparathyroidism  - Primary adrenocortical failure | c.769C>T/c.769C>T p.R257X/p.R257X (SAND) | ([45](#_ENREF_45)) |  |
|  | N/A | N/A | Non-consanguineous | Presence of at least two of the following symptoms:  - Chronic mucocutaneous candidiasis  - Hypoparathyroidism  - Primary adrenocortical failure | c.769C>T/c.769C>T p.R257X/p.R257X (SAND) | ([45](#_ENREF_45)) |  |
|  | N/A | N/A | Non-consanguineous | Presence of at least two of the following symptoms:  - Chronic mucocutaneous candidiasis  - Hypoparathyroidism  - Primary adrenocortical failure | c.769C>T/c.769C>T p.R257X/p.R257X (SAND) | ([45](#_ENREF_45)) |  |
|  | N/A | N/A | Non-consanguineous | Presence of at least two of the following symptoms:  - Chronic mucocutaneous candidiasis  - Hypoparathyroidism  - Primary adrenocortical failure | c.769C>T/c.769C>T p.R257X/p.R257X (SAND) | ([45](#_ENREF_45)) |  |
|  | N/A | N/A | Non-consanguineous | Presence of at least two of the following symptoms:  - Chronic mucocutaneous candidiasis  - Hypoparathyroidism  - Primary adrenocortical failure | c.769C>T/c.769C>T p.R257X/p.R257X (SAND) | ([45](#_ENREF_45)) |  |
|  | N/A | N/A | Non-consanguineous | Presence of at least two of the following symptoms:  - Chronic mucocutaneous candidiasis  - Hypoparathyroidism  - Primary adrenocortical failure | c.769C>T/c.769C>T p.R257X/p.R257X (SAND) | ([45](#_ENREF_45)) |  |
|  | N/A | N/A | Non-consanguineous | Presence of at least two of the following symptoms:  - Chronic mucocutaneous candidiasis  - Hypoparathyroidism  - Primary adrenocortical failure | c.769C>T/c.769C>T p.R257X/p.R257X (SAND) | ([45](#_ENREF_45)) |  |
|  | N/A | N/A | Non-consanguineous | Presence of at least two of the following symptoms:  - Chronic mucocutaneous candidiasis  - Hypoparathyroidism  - Primary adrenocortical failure | c.769C>T/c.769C>T p.R257X/p.R257X (SAND) | ([45](#_ENREF_45)) |  |
|  | N/A | N/A | Non-consanguineous | Presence of at least two of the following symptoms:  - Chronic mucocutaneous candidiasis  - Hypoparathyroidism  - Primary adrenocortical failure | c.769C>T/c.769C>T p.R257X/p.R257X (SAND) | ([45](#_ENREF_45)) |  |
|  | N/A | N/A | Non-consanguineous | Presence of at least two of the following symptoms:  - Chronic mucocutaneous candidiasis  - Hypoparathyroidism  - Primary adrenocortical failure | c.769C>T/c.769C>T p.R257X/p.R257X (SAND) | ([45](#_ENREF_45)) |  |
|  | N/A | N/A | Non-consanguineous | Presence of at least two of the following symptoms:  - Chronic mucocutaneous candidiasis  - Hypoparathyroidism  - Primary adrenocortical failure | c.769C>T/c.769C>T p.R257X/p.R257X (SAND) | ([45](#_ENREF_45)) |  |
|  | N/A | N/A | N/A | - Hypoparathyroidism  - Addison's disease  - Autoimmune thyroiditis  - Basal ganglia calcification  - Nephrocalcinosis  - Premature ovarian failure  - Renal insufficiency  - Type 1 diabetes | c.769C>T/c.769C>T p.R257X/p.R257X (SAND) | ([76](#_ENREF_76)) |  |
|  | N/A | Female | N/A | - Hypoparathyroidism  - Addison's disease  - Fatal hypoglycemia  - Premature ovarian failure  - Type 1 diabetes | c.769C>T/c.769C>T p.R257X/p.R257X (SAND) | ([76](#_ENREF_76)) |  |
|  | N/A | Female | N/A | - Hypoparathyroidism  - Autoimmune thyroiditis  - Basal ganglia calcification  - Nephrocalcinosis  - Premature ovarian failure  - Renal insufficiency | c.769C>T/c.769C>T p.R257X/p.R257X (SAND) | ([76](#_ENREF_76)) |  |
|  | N/A | N/A | N/A | - Hypoparathyroidism  - Addison's disease  - Nephrocalcinosis  - Type 1 diabetes | c.769C>T/c.769C>T p.R257X/p.R257X (SAND) | ([76](#_ENREF_76)) |  |
|  | N/A | Female | N/A | - Hypoparathyroidism  - Addison's disease  - Autoimmune thyroiditis  - Premature ovarian failure | c.769C>T/c.769C>T p.R257X/p.R257X (SAND) | ([76](#_ENREF_76)) |  |
|  | N/A | N/A | N/A | - Addison's disease | c.769C>T/c.769C>T p.R257X/p.R257X (SAND) | ([76](#_ENREF_76)) |  |
|  | N/A | N/A | N/A | - Hypoparathyroidism | c.769C>T/c.769C>T p.R257X/p.R257X (SAND) | ([76](#_ENREF_76)) |  |
|  | 2 years | Female | Non-consanguineous | - Oral candidiasis (3Y)  - Nail candidiasis (2Y)  - Hypoparathyroidism (8Y)  - Primary adrenal failure (6Y)  - Bilateral autoimmune keratitis (8Y)  - Enamel hypoplasia (8Y)  - Growth retardation | c.769C>T/c.769C>T p.R257X/p.R257X (SAND) | ([77](#_ENREF_77)) |  |
|  | 3 years | Female | Non-consanguineous | - Partial adrenal failure (6Y)  - Anemia (3Y)  - Ictal epileptic discharge (6Y) | c.769C>T/c.769C>T p.R257X/p.R257X (SAND) | ([77](#_ENREF_77)) |  |
|  | 4 years | Male | Consanguineous | - Oral candidiasis (14Y)  - Adrenal insufficiency (4Y)  - Abdominal pain (29Y)  - Alopecia totalis  - Asplenia (29Y)  - Cholelithiasis (29Y)  - Corneal opacity (6Y)  - Dysphagia (29Y)  - Weakness (29Y) | c.769C>T/c.769C>T p.R257X/p.R257X (SAND) | ([78](#_ENREF_78)) |  |
|  | 0.5 year | Male | Consanguineous | - Recurrent thrush (0.5Y)  - Yellowish discoloration and thickening of nails (2Y)  - Retinitis pigmentosa (2Y) | c.769C>T/c.769C>T p.R257X/p.R257X (SAND) | ([79](#_ENREF_79)) |  |
|  | 0.5 year | Female | Consanguineous | - Recurrent thrush (0.5Y)  - Yellowish discoloration and thickening of nails (2Y) | c.769C>T/c.769C>T p.R257X/p.R257X (SAND) | ([79](#_ENREF_79)) |  |
|  | 3 years | Male | Non-consanguineous | - Chronic mucocutaneous candidiasis (8Y)  - Alopecia (10Y)  - Arthralgia (10Y)  - Autoimmune enteropathy (9Y)  - Ectodermal dystrophy  - Autoimmune hemolytic anemia (10Y)  - Preclinical Sjogren syndrome (9.6Y)  - Undifferentiated connective tissue disease  - Vitiligo (10Y) | c.769C>T/c.769C>T p.R257X/p.R257X (SAND) | ([15](#_ENREF_15)) |  |
|  | 8 years | Female | Non-consanguineous | - Chronic mucocutaneous candidiasis  - Hypoparathyroidism  - Dental enamel hypoplasia  - Nail dystrophy  - Vitiligo | c.769C>T/c.769C>T p.R257X/p.R257X (SAND) | ([15](#_ENREF_15)) |  |
|  | N/A | N/A | Non-consanguineous | - Chronic mucocutaneous candidiasis  - Hypoparathyroidism  - Alopecia  - Autoimmune thyroiditis  - Pernicious anemia  - Type 1 diabetes mellitus | c.769C>T/c.769C>T p.R257X/p.R257X (SAND) | ([15](#_ENREF_15), [19](#_ENREF_19)) |  |
|  | 6 years | Female | Non-consanguineous | - Candidiasis  - Hypoparathyroidism  - Adrenal insufficiency (6Y)  - Alopecia (12Y and 17Y)  - Enamel dysplasia  - Hypoalbunemia  - Malabsorption  - Ungual dystrophy | c.769C>T/c.769C>T p.R257X/p.R257X (SAND) | ([80](#_ENREF_80)) |  |
|  | 5 years | Male | Non-consanguineous | - Mucocutaneous candidiasis (12Y)  - Hypoparathyroidism (5Y)  - Chronic gastritis  - Duodenogastral bile reflux  - Hypertonia  - Nail mycosis (12Y)  - Recurrent vomiting (11Y)  - Tachycardia  - Type 1 diabetes (11Y)  - Vegetative dystonia | c.769C>T/c.769C>T  p.R257X/p.R257X (SAND) | ([57](#_ENREF_57)) |  |
|  | 10 years | Male | Non-consanguineous | - Mucocutaneous candidiasis (12Y)  - Hypoparathyroidism (14Y)  - Addison's disease (10Y) | c.769C>T/c.769C>T  p.R257X/p.R257X (SAND) | ([8](#_ENREF_8)) |  |
|  | 6 years | Male | Non-consanguineous | - Mucocutaneous candidiasis (17.5Y)  - Hypoparathyroidism (6.3Y)  - Alopecia (7Y) | c.769C>T/c.769C>T  p.R257X/p.R257X (SAND) | ([8](#_ENREF_8)) |  |
|  | 7 years | Female | Non-consanguineous | - Mucocutaneous candidiasis (7.9Y)  - Hypoparathyroidism (7.8Y)  - Addison's disease (7.9Y)  - Malabsorption (13.5Y) | c.769C>T/c.769C>T  p.R257X/p.R257X (SAND) | ([8](#_ENREF_8)) |  |
|  | 6 years | Male | Non-consanguineous | - Mucocutaneous candidiasis (6.5Y)  - Hypoparathyroidism (6.5Y)  - Addison's disease (6.4Y) - Ectodermal dystrophy (6.5Y)  - Hypothyroidism (11.1Y)  - Keratoconjunctivitis (10.3Y) | c.769C>T/c.769C>T  p.R257X/p.R257X (SAND) | ([8](#_ENREF_8)) |  |
|  | 10.9 years | Male | Non-consanguineous | - Mucocutaneous candidiasis (11.1Y)  - Hypoparathyroidism (11.1Y)  - Addison's disease (10.9Y)  - Keratoconjunctivitis (13.1Y) | c.769C>T/c.769C>T  p.R257X/p.R257X (SAND) | ([8](#_ENREF_8)) |  |
|  | 3.6 years | Male | Non-consanguineous | - Mucocutaneous candidiasis (3.6Y)  - Addison's disease (8.5Y)  - Alopecia (3.6Y)  - Chronic hepatitis (3.6Y) | c.769C>T/c.769C>T  p.R257X/p.R257X (SAND) | ([8](#_ENREF_8)) |  |
|  | 0.3 year | Male | N/A | None | c.769C>T/c.769C>T  p.R257X/p.R257X (SAND) | ([9](#_ENREF_9)) |  |
|  | 0.7 year | Female | N/A | - Mucocutaneous candidiasis  - Adrenal failure  - Autoimmune hepatitis  - Celiac disease | c.769C>T/c.769C>T  p.R257X/p.R257X (SAND) | ([9](#_ENREF_9)) |  |
|  | 15 years | Female | N/A | - Hypoparathyroidism  - Adrenal failure  - Delayed puberty  - Short stature | c.769C>T/c.769C>T  p.R257X/p.R257X (SAND) | ([9](#_ENREF_9)) |  |
|  | 3.5 years | Male | Non-consanguineous | - Hypoparathyroidism (4Y)  - Addison’s disease (3.5Y) | c.769C>T/c.769C>T  p.R257X/p.R257X (SAND) | ([81](#_ENREF_81)) |  |
|  | 4 years | Female | Non-consanguineous | - Mucocutaneous candidiasis  - Hypoparathyroidism (4Y)  - Addison's disease (11Y)  - Diffuse alopecia with madarosis  - Ectodermal dystrophy  - Hashimoto's thyroiditis (13.5Y)  - Onychomycosis  - Premature ovarian failure (16Y) | c.769C>T/c.769C>T  p.R257X/p.R257X (SAND) | ([81](#_ENREF_81)) |  |
|  | 7 years | Female | N/A | - Mucocutaneous candidiasis (16Y)  - Hypoparathyroidism (7Y)  - Addison's disease (14Y) - Exocrine pancreas insufficiency (36Y)  - Hashimoto's thyroiditis  - Premature ovarian failure (30Y)  - Pure red cell aplasia  - Renal dysfunction | c.769C>T/c.769C>T  p.R257X/p.R257X (SAND) | ([81](#_ENREF_81), [82](#_ENREF_82)) |  |
|  | N/A | Female | N/A | - Chronic mucocutaneous candidiasis  - Hypoparathyroidism  - Addison's disease  - Lichen ruber planus  - Pernicious anemia | c.769C>T/c.769C>T  p.R257X/p.R257X (SAND) | ([81](#_ENREF_81), [82](#_ENREF_82)) |  |
|  | N/A | N/A | N/A | - Mucocutaneous candidiasis  - Addison's disease  - Alopecia  - Autoimmune hepatitis - Ectodermal dystrophy  - Vitiligo | c.769C>T/c.769C>T  p.R257X/p.R257X (SAND) | ([10](#_ENREF_10)) |  |
|  | N/A | N/A | N/A | - Mucocutaneous candidiasis  - Hypoparathyroidism  - Addison’s disease  - Chronic active hepatitis | c.769C>T/c.769C>T  p.R257X/p.R257X (SAND) | ([10](#_ENREF_10)) |  |
|  | N/A | N/A | N/A | - Mucocutaneous candidiasis  - Hypoparathyroidism  - Addison’s disease  - Alopecia  - Keratoconjunctivitis | c.769C>T/c.769C>T  p.R257X/p.R257X (SAND) | ([10](#_ENREF_10)) |  |
|  | N/A | N/A | N/A | - Mucocutaneous candidiasis  - Hypoparathyroidism  - Alopecia | c.769C>T/c.769C>T  p.R257X/p.R257X (SAND) | ([10](#_ENREF_10)) |  |
|  | N/A | N/A | N/A | - Hypoparathyroidism  - Addison’s disease | c.769C>T/c.769C>T  p.R257X/p.R257X (SAND) | ([10](#_ENREF_10)) |  |
|  | N/A | N/A | N/A | - Hypoparathyroidism  - Addison’s disease | c.769C>T/c.769C>T p.R257X/p.R257X (SAND) | ([10](#_ENREF_10)) |  |
|  | N/A | N/A | N/A | - Mucocutaneous candidiasis  - Hypoparathyroidism  - Addison’s disease  - Ectodermal dystrophy | c.769C>T/c.769C>T  p.R257X/p.R257X (SAND) | ([10](#_ENREF_10)) |  |
|  | N/A | N/A | N/A | - Mucocutaneous candidiasis  - Hypoparathyroidism  - Addison’s disease | c.769C>T/c.769C>T  p.R257X/p.R257X (SAND) | ([10](#_ENREF_10)) |  |
|  | N/A | N/A | N/A | - Mucocutaneous candidiasis  - Hypoparathyroidism  - Ectodermal dystrophy | c.769C>T/c.769C>T  p.R257X/p.R257X (SAND) | ([10](#_ENREF_10)) |  |
|  | N/A | N/A | N/A | - Mucocutaneous candidiasis  - Hypoparathyroidism  - Addison’s disease  - Alopecia - Chronic active hepatitis  - Ectodermal dystrophy  - Keratoconjunctivitis  - Malabsorption  - Type 1 diabetes  - Vitiligo | c.769C>T/c.769C>T  p.R257X/p.R257X (SAND) | ([10](#_ENREF_10)) |  |
|  | N/A | N/A | N/A | - Mucocutaneous candidiasis  - Hypoparathyroidism  - Addison’s disease  - Alopecia  - Ectodermal dystrophy  - Keratoconjunctivitis  - Malabsorption  - Vitiligo | c.769C>T/c.769C>T  p.R257X/p.R257X (SAND) | ([10](#_ENREF_10)) |  |
|  | N/A | N/A | N/A | - Mucocutaneous candidiasis  - Hypoparathyroidism  - Addison’s disease - Chronic active hepatitis  - Hypergonadotrophic hypogonadism | c.769C>T/c.769C>T  p.R257X/p.R257X (SAND) | ([10](#_ENREF_10)) |  |
|  | N/A | N/A | N/A | - Mucocutaneous candidiasis  - Hypoparathyroidism  - Addison’s disease  - Alopecia | c.769C>T/c.769C>T  p.R257X/p.R257X (SAND) | ([10](#_ENREF_10)) |  |
|  | N/A | N/A | N/A | - Mucocutaneous candidiasis  - Hypoparathyroidism  - Addison’s disease  - Alopecia | c.769C>T/c.769C>T  p.R257X/p.R257X (SAND) | ([10](#_ENREF_10)) |  |
|  | N/A | N/A | N/A | - Mucocutaneous candidiasis  - Hypoparathyroidism  - Addison’s disease - Gastritis  - Malabsorption | c.769C>T/c.769C>T  p.R257X/p.R257X (SAND) | ([10](#_ENREF_10)) |  |
|  | N/A | N/A | N/A | - Mucocutaneous candidiasis  - Hypoparathyroidism  - Addison’s disease - Hypothyroidism  - Malabsorption | c.769C>T/c.769C>T  p.R257X/p.R257X (SAND) | ([10](#_ENREF_10)) |  |
|  | N/A | N/A | N/A | - Mucocutaneous candidiasis  - Hypoparathyroidism  - Addison’s disease - Ectodermal dystrophy  - Gastritis  - Hypothyroidism | c.769C>T/c.769C>T  p.R257X/p.R257X (SAND) | ([10](#_ENREF_10)) |  |
|  | N/A | Female | N/A | - Mucocutaneous candidiasis  - Hypoparathyroidism  - Addison’s disease | c.769C>T/c.769C>T  p.R257X/p.R257X (SAND) | ([58](#_ENREF_58)) |  |
|  | N/A | Male | N/A | - Mucocutaneous candidiasis  - Hypoparathyroidism  - Addison’s disease | c.769C>T/c.769C>T  p.R257X/p.R257X (SAND) | ([58](#_ENREF_58)) |  |
|  | 1 year | Female | N/A | - Mucocutaneous candidiasis (1Y)  - Hypoparathyroidism (5Y)  - Addison’s disease (4Y)  - Vitiligo (1Y) | c.769C>T/c.769C>T  p.R257X/p.R257X (SAND) | ([1](#_ENREF_1)) |  |
|  | 5 years | Female | N/A | - Mucocutaneous candidiasis (5Y)  - Hypoparathyroidism (15Y)  - Addison’s disease (16Y)  - Chronic hepatitis (15Y)  - Pernicious anemia (16Y) | c.769C>T/c.769C>T  p.R257X/p.R257X (SAND) | ([1](#_ENREF_1)) |  |
|  | 6 years | Male | N/A | - Mucocutaneous candidiasis (6Y)  - Hypoparathyroidism (9Y)  - Addison's disease (11Y)  - Malabsorption (9Y) | c.769C>T/c.769C>T  p.R257X/p.R257X (SAND) | ([1](#_ENREF_1)) |  |
|  | 9 years | Female | N/A | - Mucocutaneous candidiasis (10Y)  - Hypoparathyroidism (9Y)  - Addison's disease (10Y)  - Alopecia (10Y)  - Hypogonadism (18Y) | c.769C>T/c.769C>T  p.R257X/p.R257X (SAND) | ([1](#_ENREF_1)) |  |
|  | 10 years | Male | N/A | - Mucocutaneous candidiasis (11Y)  - Hypoparathyroidism (13Y)  - Addison's disease (14Y)  - Alopecia (13Y)  - Malabsorption (11Y)  - Pernicious anemia (10Y) | c.769C>T/c.769C>T  p.R257X/p.R257X (SAND) | ([1](#_ENREF_1), [81](#_ENREF_81), [83](#_ENREF_83)) |  |
|  | N/A | N/A | N/A | - Chronic mucocutaneous candidiasis  - Hypoparathyroidism  - Addison's disease  - Alopecia  - Dental enamel hypoplasia  - Dystrophy of the nails  - Keratopathy  - Malabsorption  - Pernicious anemia | c.769C>T/c.769C>T  p.R257X/p.R257X (SAND) | ([84](#_ENREF_84)) |  |
|  | 9 years | Male | Non-consanguineous | - Mucocutaneous candidiasis - Hypoparathyroidism (9Y) - Adrenal insufficiency (16Y) - Alopecia - Vitiligo | c.769C>T/c.769C>T  p.R257X/p.R257X (SAND) | ([11](#_ENREF_11), [85](#_ENREF_85)) |  |
|  | 1 year | Female | Non-consanguineous | - Mucocutaneous candidiasis (5Y) - Hypoparathyroidism (1Y) - Alopecia - Dental enamel hypoplasia - Nail pitting  - Primary gonadal insufficiency | c.769C>T/c.769C>T  p.R257X/p.R257X (SAND) | ([11](#_ENREF_11)) |  |
|  | N/A | Male | N/A | N/A | c.769C>T/c.769C>T  p.R257X/p.R257X (SAND) | ([55](#_ENREF_55)) |  |
|  | 3.5 years | Male | N/A | - Alopecia (4Y) - Dental enamel hypoplasia (3.5Y) | c.769C>T/c.769C>T  p.R257X/p.R257X (SAND) | ([55](#_ENREF_55)) |  |
|  | 1 year | Female | N/A | - Chronic mucocutaneous candidiasis (4.5Y) - Hypoparathyroidism (4.1Y) - Addison’s disease (25.7Y) - Diabetes mellitus (38Y) - Malabsorption, diarrhea or obstipation (4Y) - Ovarian failure (26Y) - Pernicious anemia (15.5Y) - Urticaria (1Y) - Vasculitis (1Y) - Vitiligo (11Y) | c.769C>T/c.769C>T  p.R257X/p.R257X (SAND) | ([55](#_ENREF_55)) |  |
|  | 2.6 years | Female | N/A | - Chronic mucocutaneous candidiasis (2.6Y) - Hypoparathyroidism (2.8Y) - Autoimmune thyroiditis (26Y) | c.769C>T/c.769C>T  p.R257X/p.R257X (SAND) | ([55](#_ENREF_55)) |  |
|  | 1.5 years | Female | N/A | - Chronic mucocutaneous candidiasis (1.5Y) - Hypoparathyroidism (1.5Y) - Autoimmune hepatitis (4Y) - Urticaria (5Y) | c.769C>T/c.769C>T  p.R257X/p.R257X (SAND) | ([55](#_ENREF_55)) |  |
|  | 2 years | Male | N/A | - Chronic mucocutaneous candidiasis (2Y) - Addison’s disease (4.5Y) - Alopecia (4Y) - Malabsorption, diarrhea or obstipation (2Y) | c.769C>T/c.769C>T  p.R257X/p.R257X (SAND) | ([55](#_ENREF_55)) |  |
|  | 1.5 years | Male | N/A | - Chronic mucocutaneous candidiasis (1.5Y) - Hypoparathyroidism (5.5Y) - Addison’s disease (4Y) - Dental enamel hypoplasia (10Y) - Epilepsy (8.5Y) | c.769C>T/c.769C>T  p.R257X/p.R257X (SAND) | ([55](#_ENREF_55)) |  |
|  | 11 years | Male | N/A | - Fungal infections (11Y) - Hypoparathyroidism (11Y) - Addison’s disease (11Y) - Hepatitis (12Y) | c.769C>T/c.769C>T  p.R257X/p.R257X (SAND) | ([4](#_ENREF_4)) |  |
|  | 4 years | Male | N/A | - Hypoparathyroidism (4Y) - Addison’s disease (9Y) - Alopecia - Keratitis | c.769C>T/c.769C>T  p.R257X/p.R257X (SAND) | ([4](#_ENREF_4)) |  |
|  | 5 years | Female | N/A | - Fungal infections (5Y) - Hypoparathyroidism (5Y) | c.769C>T/c.769C>T  p.R257X/p.R257X (SAND) | ([4](#_ENREF_4)) |  |
|  | 1 year | Female | N/A | - Fungal infections (1Y) - Hypoparathyroidism (7Y) | c.769C>T/c.769C>T  p.R257X/p.R257X (SAND) | ([4](#_ENREF_4)) |  |
|  | 4 years | Female | N/A | - Fungal infections (4Y) - Hypoparathyroidism (6.5Y) - Addison’s disease (10Y) | c.769C>T/c.769C>T  p.R257X/p.R257X (SAND) | ([4](#_ENREF_4)) |  |
|  | 2 years | Female | N/A | - Fungal infections (2Y) - Hypoparathyroidism (6Y) - Conjunctivitis  - Keratitis  - Premature ovarian failure | c.769C>T/c.769C>T  p.R257X/p.R257X (SAND) | ([4](#_ENREF_4)) |  |
|  | 6 years | Female | N/A | - Fungal infections (6Y) - Hypoparathyroidism (13Y) - Alopecia - Keratitis | c.769C>T/c.769C>T  p.R257X/p.R257X (SAND) | ([4](#_ENREF_4)) |  |
|  | 10 years | Male | N/A | - Fungal infections (10Y) - Hypoparathyroidism (10Y) - Keratitis (11Y) | Unknown  Sibling of Pt. 553  Probably harbors the same mutation | ([4](#_ENREF_4)) |  |
|  | 3 years | Female | N/A | - Fungal infections (7.5Y) - Hypoparathyroidism (3Y) - Addison’s disease (12Y) - Aggressive hepatitis - Alopecia - Malabsorption - Pancreatitis - Premature ovarian failure | c.769C>T/c.967_979del13bp  p.R257X (SAND)/p.L323fs (PHD1) | ([4](#_ENREF_4)) |  |
|  | 1 year | Female | N/A | - Fungal infections (1.5Y) - Hypoparathyroidism (1Y) - Addison’s disease (3.5Y) - Conjunctivitis  - Hepatitis - Premature ovarian failure (16Y) - Premature puberty | c.769C>T/c.932G>A p.R257X (SAND)/p.C311Y (PHD1) | ([4](#_ENREF_4)) |  |
|  | 2 years | Male | N/A | - Fungal infections (2Y) - Hypoparathyroidism (9.5Y) - Addison’s disease (8Y) - Hepatitis - Cataract - Pernicious anemia | c.769C>T/Unknown  p.R257X (SAND)/Unknown | ([4](#_ENREF_4)) |  |
|  | 12 years | Female | N/A | - Fungal infections (12Y) - Hypoparathyroidism (12Y) - Deafness | c.769C>T/Unknown  p.R257X (SAND)/Unknown | ([4](#_ENREF_4)) |  |
|  | 4.2 years | Female | N/A | - Chronic mucocutaneous candidiasis (9Y)  - Hypoparathyroidism (4.8Y) - Addison’s disease (5.2Y) - Malabsorption, diarrhea or obstipation (4.2Y) | c.769C>T/c.967_979del13bp  p.R257X (SAND)/p.L323fs (PHD1) | ([55](#_ENREF_55)) |  |
|  | 3 years | Male | N/A | - Chronic mucocutaneous candidiasis (3Y)  - Hypoparathyroidism (3Y)  - Addison’s disease (12Y) - Alopecia (5Y) - Autoimmune thyroiditis (14Y) - Keratoconjunctivitis (3Y) | c.769C>T/c.967_979del13bp  p.R257X (SAND)/p.L323fs (PHD1) | ([55](#_ENREF_55)) |  |
|  | 2 years | Male | N/A | - Chronic mucocutaneous candidiasis (2Y) - Hypoparathyroidism (6Y) - Blepharitis (11Y) - Malabsorption, diarrhea or obstipation (15Y) | c.769C>T/c.967_979del13bp  p.R257X (SAND)/p.L323fs (PHD1) | ([55](#_ENREF_55)) |  |
|  | N/A | N/A | N/A | - Chronic mucocutaneous candidiasis  - Hypoparathyroidism  - Insulin dependent diabetes mellitus | c.769C>T/Unknown  p.R257X (SAND)/Unknown | ([84](#_ENREF_84)) |  |
|  | N/A | N/A | N/A | - Chronic mucocutaneous candidiasis  - Hypoparathyroidism  - Addison's disease  - Alopecia  - Mosaic of Down's syndrome (46, XY/47, XY + 21) | c.769C>T/Unknown  p.R257X (SAND)/Unknown | ([84](#_ENREF_84)) |  |
|  | 15 years | Male | N/A | - Mucocutaneous candidiasis | c.769C>T/Unknown  p.R257X (SAND)/Unknown | ([86](#_ENREF_86)) |  |
|  | 7 years | Male | N/A | - Hypoparathyroidism - Mild mental retardation  - Minor facial dysmorphism - Prolonged Q-T interval  - Recurrent laryngitis | c.769C>T/Unknown  p.R257X (SAND)/Unknown | ([86](#_ENREF_86)) |  |
|  | N/A | N/A | N/A | N/A | c.769C>T/c.1264_1265insA  p.R257X (SAND)/p.P422fs (PRR) | ([87](#_ENREF_87)) |  |
|  | N/A | N/A | N/A | N/A | c.769C>T/c.1264_1265insA  p.R257X (SAND)/p.P422fs (PRR) | ([87](#_ENREF_87)) |  |
|  | N/A | N/A | N/A | N/A | c.769C>T/c.1264_1265insA  p.R257X (SAND)/p.P422fs (PRR) | ([87](#_ENREF_87)) |  |
|  | N/A | Male | N/A | - Hypoparathyroidism (9.5Y)  - Adrenal hypofunction (11Y)  - Corneal degeneration (14Y)  - Cataract (14Y) - Allergic atopic dermatitis  - Atopic allergic asthma - Intermittent angular cheilitis (21Y)  - Recurrent non-purulent conjunctivitis - Recurrent sinusitis  - Mitral prolapse with mild regurgitation | c.769C>T/c.1344delC  p.R257X (SAND)/p.C449fs (PHD2) | ([73](#_ENREF_73)) |  |
|  | 11 years | Male | N/A | - Isolated nail candidiasis (17Y)  - Hypoparathyroidism (11Y) - Hypoadrenia (21Y) | c.769C>T/c.1344delC  p.R257X (SAND)/p.C449fs (PHD2) | ([73](#_ENREF_73)) |  |
|  | N/A | Female | N/A | - Hypoparathyroidism  - Addison’s disease  - Alopecia | c.769C>T/c.232T>C p.R257X (SAND)/p.W78R (HSR/CARD) | ([74](#_ENREF_74)) |  |
|  | N/A | Female | N/A | - Candidiasis  - Hypoparathyroidism  - Alopecia areata  - Bronchial asthma  - Celiac disease | c.769C>T/c.32T>C p.R257X (SAND)/p.L11P (L) | ([62](#_ENREF_62)) |  |
|  | 11 years | Female | N/A | - Vulvovaginal candidiasis (26Y) - Hypoparathyroidism (13Y) - Primary adrenal insufficiency  - Alopecia areata (14Y) - Primary hypothyroidism (22Y)  - Vitamin B12 deficiency (14Y) | c.769C>T/c.821delG p.R257X (SAND)/p.G274fs (SAND) | ([88](#_ENREF_88)) |  |
|  | 9 years | Male | Non-consanguineous | - Mucocutaneous candidiasis (24.5Y)  - Hypoparathyroidism (9.4Y)  - Alopecia (9Y)  - Type 1 diabetes (21Y)  - Vitiligo (11.6Y) | c.769C>T/c.540delG p.R257X (SAND)/p.G180fs (upstream of SAND) | ([8](#_ENREF_8)) |  |
|  | 3.8 years | Female | Non-consanguineous | - Mucocutaneous candidiasis (4.6Y)  - Hypoparathyroidism (3.8Y)  - Addison's disease (4Y) | c.769C>T/c.653-7_-5delCTC p.R257X (SAND)/p.G218fs (SAND) | ([8](#_ENREF_8)) |  |
|  | 1.4 years | Male | Non-consanguineous | - Oral thrush (candidiasis)  - Cough, congestion  - Diaper rash  - Hypoglycemia, low serum ketones  - Jaundice  - Native liver showed greater than 95% parenchymal extinction with bile ductular reaction and associated inflammatory infiltrates  - Ravenous feeder with intermittent episodes of feeding  - Solitary right kidney | c.769C>T/c.1095+1G>A p.R257X (SAND)/E9del (PHD1) | ([89](#_ENREF_89)) |  |
|  | N/A | N/A | N/A | - Mucocutaneous candidiasis  - Hypoparathyroidism | c.769C>T/c.967_979del13bp  p.R257X (SAND)/p.L323fs (PHD1) | ([10](#_ENREF_10)) |  |
|  | 1 year | Male | N/A | - Hypoparathyroidism | c.769C>T/c.967_979del13bp  p.R257X (SAND)/p.L323fs (PHD1) | ([9](#_ENREF_9)) |  |
|  | 6.5 years | Male | N/A | - Mucocutaneous candidiasis  - Hypoparathyroidism  - Adrenal failure  - Autoimmune thyroiditis | c.769C>T/c.1344delC p.R257X (SAND)/p.C449fs (PHD2) | ([9](#_ENREF_9)) |  |
|  | 11 years | Male | N/A | - Hypoparathyroidism | c.769C>T/c.1344delC p.R257X (SAND)/p.C449fs (PHD2) | ([9](#_ENREF_9)) |  |
|  | 3 years | Female | Non-consanguineous | - Chronic candidiasis (3Y) - Chronic hypoparathyroidism (6Y) - Addison’s disease (21Y) - Autoimmune gastritis - Hashimoto’s thyroiditis - Pernicious anemia  - Premature ovarian failure | c.769C>T/c.967_979del13bp  p.R257X (SAND)/p.L323fs (PHD1) | ([36](#_ENREF_36)) |  |
|  | 1 year | Female | Non-consanguineous | - Chronic candidiasis (1Y) - Chronic hypoparathyroidism (18Y) - Addison’s disease (14Y) - Alopecia - Autoimmune gastritis - Cancer of oral mucosa - Pernicious anemia - Premature ovarian failure - Vitiligo | c.769C>T/c.967_979del13bp  p.R257X (SAND)/p.L323fs (PHD1) | ([36](#_ENREF_36)) |  |
|  | 10 years | Female | Non-consanguineous | - Chronic candidiasis (10Y) - Chronic hypoparathyroidism (13Y) - Addison’s disease (32Y) - Alopecia - Autoimmune gastritis - Gastric adenocarcinoma - Turner’s syndrome | c.769C>T/c.967_979del13bp  p.R257X (SAND)/p.L323fs (PHD1) | ([36](#_ENREF_36)) |  |
|  | 3 years |  | Non-consanguineous | - Chronic candidiasis (3Y) - Chronic hypoparathyroidism (3Y) - Addison’s disease (21Y) - Asplenia - Autoimmune gastritis - Celiac disease - Extrapyramidal syndrome  - Pernicious anemia - Premature ovarian failure | c.769C>T/c.967_979del13bp  p.R257X (SAND)/p.L323fs (PHD1) | ([36](#_ENREF_36)) |  |
|  | 5.5 years | N/A | N/A | - Hypoparathyroidism (6Y)  - Adrenal insufficiency (5.5Y)  - Autoimmune hepatitis (7.1Y) | c.769C>T/c.892G>A  p.R257X (SAND)/p.E298K (upstream of PHD1) | ([6](#_ENREF_6)) |  |
|  | 1.5 years | N/A | N/A | - Chronic mucocutaneous candidiasis (1.5Y) - Hypoparathyroidism (3.5Y) - Hyperthyroidism (21Y) | c.769C>T/c.1053_1060del8 p.R257X (SAND)/p.R351fs (PRR) | ([6](#_ENREF_6)) |  |
|  | 7.5 years | Female | N/A | - Mucocutaneous candidiasis (11Y)  - Hypoparathyroidism (7.5Y)  - Addison's disease (8Y)  - Autoimmune bronchiolitis (3.5Y)  - Chronic otitis media with effusion (16.5Y)  - Hypogonadism (12Y)  - Pernicious anemia (17Y)  - Systemic juvenile rheumatoid arthritis | c.769C>T/c.892G>A  p.R257X (SAND)/p.E298K (upstream of PHD1) | ([1](#_ENREF_1)) |  |
|  | 2 years | Male | N/A | - Addison's disease (11Y)  - Alopecia (2.5Y) - Ectodermal dystrophy (2Y)  - Malabsorption (2Y)  - Vitiligo (2Y) | c.769C>T/c.462A>T  p.R257X (SAND)/p.P154P (Silent mutation affecting E3 splicing resulting in E3del) (downstream of NLS) | ([1](#_ENREF_1)) |  |
|  | N/A | Female | N/A | - Mucocutaneous candidiasis  - Hypoparathyroidism  - Adrenal insufficiency - Alopecia  - Autoimmune thyroid disease - Dental enamel hypoplasia  - Primary gonadal insufficiency - Type 1 diabetes | c.769C>T/c.1336T>G p.R257X (SAND)/p.C446G (PHD2) | ([68](#_ENREF_68)) |  |
|  | N/A | Female | N/A | - Adrenal insufficiency | c.769C>T/c.1242_1243insA p.R257X (SAND)/p.H415fs (L) | ([68](#_ENREF_68)) |  |
|  | N/A | Female | N/A | - Mucocutaneous candidiasis  - Adrenal insufficiency  - Nail pitting  - Primary gonadal insufficiency  - Vitiligo | c.769C>T/c.1242_1243insA p.R257X (SAND)/p.H415fs (L) | ([68](#_ENREF_68)) |  |
|  | N/A | Female | N/A | - Mucocutaneous candidiasis - Hypoparathyroidism - Adrenal insufficiency | c.769C>T/c.967_979del13bp p.R257X (SAND)/p.L323fs (PHD1) | ([68](#_ENREF_68)) |  |
|  | 7 years | N/A | N/A | - Hypoparathyroidism (7Y) - Adrenal insufficiency (14.5Y) | c.769C>T/c.967_979del13bp p.R257X (SAND)/p.L323fs (PHD1) | ([6](#_ENREF_6)) |  |
|  | 4 years | Female | Non-consanguineous | - Chronic mucocutaneous candidiasis  - Hypoparathyroidism - Primary adrenal insufficiency | c.769C>T/c.55G>A p.R257X (SAND)/p.A19T (HSR/CARD) | ([33](#_ENREF_33)) |  |
|  | N/A | N/A | N/A | N/A | c.769C>T/c.247A>G p.R257X (SAND)/p.K83E (HSR/CARD) | ([69](#_ENREF_69)) |  |
|  | 2 years | Female | Non-consanguineous | - Hypoparathyroidism (4Y)  - Autoimmune hepatitis (5Y)  - Constipation with painful defecation (3Y)  - Diarrhea and constipation (2Y)  - Exocrine pancreas insufficiency (7Y)  - Megacolon and ileostomy (5Y) | c.769C>T/c.1567-2A>G p.R257X (SAND)/IVS-2A>G | ([57](#_ENREF_57)) |  |
|  | 1 year | Male | Non-consanguineous | - Mucocutaneous candidiasis (3Y)  - Autoimmune hepatitis (2Y)  - Chronic recurrent urticaria (5Y)  - Gastritis  - Iron deficiency anemia (2Y)  - Malabsorption (8Y)  - Ocular sicca syndrome (6Y)  - Recurrent episodes of fever (1Y)  - Stomatitis (1Y)  - Vitamin B12 anemia (7Y) | c.769C>T/c.1616C>T  p.R257X (SAND)/p.P539L (TAD) | ([57](#_ENREF_57)) |  |
|  | 0.8 year | Male | N/A | - Oral thrush (0.8Y)  - Candidal onychomycosis (5Y)  - Hypoparathyroidism (11Y)  - Adrenal insufficiency (3Y) - Alopecia (7Y)  - Aural polyp (12Y)  - Autoimmune hepatitis (15Y)  - Enteropathy (6Y)  - Gastroparesis (12Y)  - Hypothyroidism (12Y)  - Megacolon (12Y)  - Nasal polyps recurrent (12Y)  - Pancreatic insufficiency (2Y) - Raynaud's disease (7Y)  - Sjogren's syndrome (10Y)  - Uveitis (4Y)  - Vitiligo (8Y) | AIRE: c.769C>T/WT  p.R257X (SAND)/WT  BTNL2: frameshift mutation at the enhancer coding sequence region 6:32370969 (TG>T), rs139418003  p.His151ThrfsTer96/WT  This reference identified a heterozygous mutation in *AIRE* and a heretozygous deletion in the regulatory gene *BTNL2*, and proposes that the mutation in *BTNL2* may have contributed to the development of the severe autoimmune manifestations in this patient with a single pathogenic AIRE mutation. | ([90](#_ENREF_90)) |  |
|  | 3 years | Female | Non-consanguineous | - Candidiasis (21Y)  - Hypoparathyroidism (3Y)  - Gastritis (18Y)  - Hypogonadism (22Y) | c.769C>T/c.977C>T p.R257X (SAND)/p.P326L (PHD1) | ([91](#_ENREF_91)) |  |
|  | 4 years | Female | Non-consanguineous | - Candidiasis  - Hypoparathyroidism (4Y)  - Addison's disease (12Y)  - Gastritis (33Y)  - Hypogonadism (14Y)  - Vitiligo | c.769C>T/c.977C>T p.R257X (SAND)/p.P326L (PHD1) | ([91](#_ENREF_91)) |  |
|  | 29 years | Female | Non-consanguineous | - Hypoparathyroidism (29Y) | c.769C>T/c.977C>T p.R257X (SAND)/p.P326L (PHD1) | ([91](#_ENREF_91)) |  |
|  | 9 years | Male | Non-consanguineous | - Candidiasis (11Y)  - Hypoparathyroidism (11Y)  - Addison’s disease (9Y)  - Malabsorption (17Y)  - Vitiligo (24Y) | c.769C>T/Unknown  p.R257X (SAND)/Unknown | ([91](#_ENREF_91)) |  |
|  | N/A | N/A | N/A | N/A | c.769C>T/Unknown p.R257X (SAND)/Unknown | ([69](#_ENREF_69)) |  |
|  | N/A | N/A | N/A | - Hypoparathyroidism  - Addison’s disease  - Hypothyroidism | c.769C>T/Unknown p.R257X (SAND)/Unknown | ([10](#_ENREF_10)) |  |
|  | N/A | Female | N/A | - Mucocutaneous candidiasis  - Hypoparathyroidism  - Addison’s disease | c.769C>T/WT  p.R257X (SAND)/WT | ([58](#_ENREF_58)) |  |
|  | N/A | N/A | N/A | N/A | c.769C>T/Unknown  p.R257X (SAND)/Unknown | ([23](#_ENREF_23)) |  |
|  | N/A | N/A | N/A | N/A | c.769C>T/Unknown  p.R257X (SAND)/Unknown | ([23](#_ENREF_23)) |  |
|  | N/A | N/A | N/A | N/A | c.769C>T/Unknown  p.R257X (SAND)/Unknown | ([23](#_ENREF_23)) |  |
|  | N/A | N/A | N/A | N/A | c.769C>T/Unknown  p.R257X (SAND)/Unknown | ([23](#_ENREF_23)) |  |
|  | N/A | N/A | N/A | N/A | c.769C>T/Unknown  p.R257X (SAND)/Unknown | ([23](#_ENREF_23)) |  |
|  | N/A | N/A | N/A | N/A | c.769C>T/Unknown  p.R257X (SAND)/Unknown | ([23](#_ENREF_23)) |  |
|  | N/A | N/A | N/A | N/A | c.769C>T/Unknown  p.R257X (SAND)/Unknown | ([23](#_ENREF_23)) |  |
|  | N/A | N/A | N/A | N/A | c.769C>T/Unknown  p.R257X (SAND)/Unknown | ([23](#_ENREF_23)) |  |
|  | N/A | N/A | N/A | N/A | c.769C>T/Unknown  p.R257X (SAND)/Unknown | ([23](#_ENREF_23)) |  |
|  | N/A | N/A | N/A | N/A | c.769C>T/Unknown  p.R257X (SAND)/Unknown | ([23](#_ENREF_23)) |  |
|  | N/A | N/A | N/A | N/A | c.769C>T/Unknown  p.R257X (SAND)/Unknown | ([23](#_ENREF_23)) |  |
|  | N/A | N/A | N/A | N/A | c.769C>T/Unknown  p.R257X (SAND)/Unknown | ([23](#_ENREF_23)) |  |
|  | N/A | N/A | Non-consanguineous | Presence of at least two of the following symptoms:  - Chronic mucocutaneous candidiasis  - Hypoparathyroidism  - Primary adrenocortical failure | c.769C>T/Unknown  p.R257X (SAND)/Unknown | ([45](#_ENREF_45)) |  |
|  | N/A | N/A | Non-consanguineous | Presence of at least two of the following symptoms:  - Chronic mucocutaneous candidiasis  - Hypoparathyroidism  - Primary adrenocortical failure | c.769C>T/Unknown  p.R257X (SAND)/Unknown | ([45](#_ENREF_45)) |  |
|  | N/A | N/A | Non-consanguineous | Presence of at least two of the following symptoms:  - Chronic mucocutaneous candidiasis  - Hypoparathyroidism  - Primary adrenocortical failure | c.769C>T/Unknown  p.R257X (SAND)/Unknown | ([45](#_ENREF_45)) |  |
|  | N/A | N/A | Non-consanguineous | Presence of at least two of the following symptoms:  - Chronic mucocutaneous candidiasis  - Hypoparathyroidism  - Primary adrenocortical failure | c.769C>T/Unknown  p.R257X (SAND)/Unknown | ([45](#_ENREF_45)) |  |
|  | N/A | N/A | Non-consanguineous | Presence of at least two of the following symptoms:  - Chronic mucocutaneous candidiasis  - Hypoparathyroidism  - Primary adrenocortical failure | c.769C>T/Unknown  p.R257X (SAND)/Unknown | ([45](#_ENREF_45)) |  |
|  | N/A | N/A | Non-consanguineous | Presence of at least two of the following symptoms:  - Chronic mucocutaneous candidiasis  - Hypoparathyroidism  - Primary adrenocortical failure | c.769C>T/Unknown  p.R257X (SAND)/Unknown | ([45](#_ENREF_45)) |  |
|  | N/A | N/A | Non-consanguineous | Presence of at least two of the following symptoms:  - Chronic mucocutaneous candidiasis  - Hypoparathyroidism  - Primary adrenocortical failure | c.769C>T/Unknown  p.R257X (SAND)/Unknown | ([45](#_ENREF_45)) |  |
|  | N/A | N/A | Non-consanguineous | Presence of at least two of the following symptoms:  - Chronic mucocutaneous candidiasis  - Hypoparathyroidism  - Primary adrenocortical failure | c.769C>T/Unknown  p.R257X (SAND)/Unknown | ([45](#_ENREF_45)) |  |
|  | N/A | N/A | Non-consanguineous | Presence of at least two of the following symptoms:  - Chronic mucocutaneous candidiasis  - Hypoparathyroidism  - Primary adrenocortical failure | c.769C>T/Unknown  p.R257X (SAND)/Unknown | ([45](#_ENREF_45)) |  |
|  | N/A | N/A | Non-consanguineous | Presence of at least two of the following symptoms:  - Chronic mucocutaneous candidiasis  - Hypoparathyroidism  - Primary adrenocortical failure | c.769C>T/Unknown  p.R257X (SAND)/Unknown | ([45](#_ENREF_45)) |  |
|  | N/A | N/A | Non-consanguineous | Presence of at least two of the following symptoms:  - Chronic mucocutaneous candidiasis  - Hypoparathyroidism  - Primary adrenocortical failure | c.769C>T/Unknown  p.R257X (SAND)/Unknown | ([45](#_ENREF_45)) |  |
|  | N/A | N/A | Non-consanguineous | Presence of at least two of the following symptoms:  - Chronic mucocutaneous candidiasis  - Hypoparathyroidism  - Primary adrenocortical failure | c.769C>T/Unknown  p.R257X (SAND)/Unknown | ([45](#_ENREF_45)) |  |
|  | N/A | N/A | Non-consanguineous | Presence of at least two of the following symptoms:  - Chronic mucocutaneous candidiasis  - Hypoparathyroidism  - Primary adrenocortical failure | c.769C>T/Unknown  p.R257X (SAND)/Unknown | ([45](#_ENREF_45)) |  |
|  | N/A | N/A | Non-consanguineous | Presence of at least two of the following symptoms:  - Chronic mucocutaneous candidiasis  - Hypoparathyroidism  - Primary adrenocortical failure | c.769C>T/Unknown  p.R257X (SAND)/Unknown | ([45](#_ENREF_45)) |  |
|  | N/A | N/A | Non-consanguineous | Presence of at least two of the following symptoms:  - Chronic mucocutaneous candidiasis  - Hypoparathyroidism  - Primary adrenocortical failure | c.769C>T/Unknown  p.R257X (SAND)/Unknown | ([45](#_ENREF_45)) |  |
|  | N/A | N/A | Non-consanguineous | Presence of at least two of the following symptoms:  - Chronic mucocutaneous candidiasis  - Hypoparathyroidism  - Primary adrenocortical failure | c.769C>T/Unknown  p.R257X (SAND)/Unknown | ([45](#_ENREF_45)) |  |
|  | N/A | N/A | Non-consanguineous | Presence of at least two of the following symptoms:  - Chronic mucocutaneous candidiasis  - Hypoparathyroidism  - Primary adrenocortical failure | c.769C>T/Unknown  p.R257X (SAND)/Unknown | ([45](#_ENREF_45)) |  |
|  | N/A | N/A | Non-consanguineous | Presence of at least two of the following symptoms:  - Chronic mucocutaneous candidiasis  - Hypoparathyroidism  - Primary adrenocortical failure | c.769C>T/Unknown  p.R257X (SAND)/Unknown | ([45](#_ENREF_45)) |  |
|  | N/A | N/A | Non-consanguineous | Presence of at least two of the following symptoms:  - Chronic mucocutaneous candidiasis  - Hypoparathyroidism  - Primary adrenocortical failure | c.769C>T/Unknown  p.R257X (SAND)/Unknown | ([45](#_ENREF_45)) |  |
|  | N/A | N/A | Non-consanguineous | Presence of at least two of the following symptoms:  - Chronic mucocutaneous candidiasis  - Hypoparathyroidism  - Primary adrenocortical failure | c.769C>T/Unknown  p.R257X (SAND)/Unknown | ([45](#_ENREF_45)) |  |
|  | N/A | N/A | Non-consanguineous | Presence of at least two of the following symptoms:  - Chronic mucocutaneous candidiasis  - Hypoparathyroidism  - Primary adrenocortical failure | c.769C>T/Unknown  p.R257X (SAND)/Unknown | ([45](#_ENREF_45)) |  |
|  | N/A | N/A | Non-consanguineous | Presence of at least two of the following symptoms:  - Chronic mucocutaneous candidiasis  - Hypoparathyroidism  - Primary adrenocortical failure | c.769C>T/Unknown  p.R257X (SAND)/Unknown | ([45](#_ENREF_45)) |  |
|  | N/A | N/A | Non-consanguineous | Presence of at least two of the following symptoms:  - Chronic mucocutaneous candidiasis  - Hypoparathyroidism  - Primary adrenocortical failure | c.769C>T/Unknown  p.R257X (SAND)/Unknown | ([45](#_ENREF_45)) |  |
|  | N/A | N/A | Non-consanguineous | Presence of at least two of the following symptoms:  - Chronic mucocutaneous candidiasis  - Hypoparathyroidism  - Primary adrenocortical failure | c.769C>T/Unknown  p.R257X (SAND)/Unknown | ([45](#_ENREF_45)) |  |
|  | N/A | N/A | Non-consanguineous | Presence of at least two of the following symptoms:  - Chronic mucocutaneous candidiasis  - Hypoparathyroidism  - Primary adrenocortical failure | c.769C>T/Unknown  p.R257X (SAND)/Unknown | ([45](#_ENREF_45)) |  |
|  | N/A | N/A | Non-consanguineous | Presence of at least two of the following symptoms:  - Chronic mucocutaneous candidiasis  - Hypoparathyroidism  - Primary adrenocortical failure | c.769C>T/Unknown  p.R257X (SAND)/Unknown | ([45](#_ENREF_45)) |  |
|  | N/A | N/A | Non-consanguineous | Presence of at least two of the following symptoms:  - Chronic mucocutaneous candidiasis  - Hypoparathyroidism  - Primary adrenocortical failure | c.769C>T/Unknown  p.R257X (SAND)/Unknown | ([45](#_ENREF_45)) |  |
|  | N/A | N/A | Non-consanguineous | Presence of at least two of the following symptoms:  - Chronic mucocutaneous candidiasis  - Hypoparathyroidism  - Primary adrenocortical failure | c.769C>T/Unknown  p.R257X (SAND)/Unknown | ([45](#_ENREF_45)) |  |
|  | N/A | N/A | Non-consanguineous | Presence of at least two of the following symptoms:  - Chronic mucocutaneous candidiasis  - Hypoparathyroidism  - Primary adrenocortical failure | c.769C>T/Unknown  p.R257X (SAND)/Unknown | ([45](#_ENREF_45)) |  |
|  | N/A | N/A | Non-consanguineous | Presence of at least two of the following symptoms:  - Chronic mucocutaneous candidiasis  - Hypoparathyroidism  - Primary adrenocortical failure | c.769C>T/Unknown  p.R257X (SAND)/Unknown | ([45](#_ENREF_45)) |  |
|  | N/A | N/A | Non-consanguineous | Presence of at least two of the following symptoms:  - Chronic mucocutaneous candidiasis  - Hypoparathyroidism  - Primary adrenocortical failure | c.769C>T/Unknown  p.R257X (SAND)/Unknown | ([45](#_ENREF_45)) |  |
|  | N/A | N/A | Non-consanguineous | Presence of at least two of the following symptoms:  - Chronic mucocutaneous candidiasis  - Hypoparathyroidism  - Primary adrenocortical failure | c.769C>T/Unknown  p.R257X (SAND)/Unknown | ([45](#_ENREF_45)) |  |
|  | N/A | N/A | Non-consanguineous | Presence of at least two of the following symptoms:  - Chronic mucocutaneous candidiasis  - Hypoparathyroidism  - Primary adrenocortical failure | c.769C>T/Unknown  p.R257X (SAND)/Unknown | ([45](#_ENREF_45)) |  |
|  | N/A | N/A | Non-consanguineous | Presence of at least two of the following symptoms:  - Chronic mucocutaneous candidiasis  - Hypoparathyroidism  - Primary adrenocortical failure | c.769C>T/Unknown  p.R257X (SAND)/Unknown | ([45](#_ENREF_45)) |  |
|  | N/A | N/A | Non-consanguineous | Presence of at least two of the following symptoms:  - Chronic mucocutaneous candidiasis  - Hypoparathyroidism  - Primary adrenocortical failure | c.769C>T/Unknown  p.R257X (SAND)/Unknown | ([45](#_ENREF_45)) |  |
|  | N/A | N/A | Non-consanguineous | Presence of at least two of the following symptoms:  - Chronic mucocutaneous candidiasis  - Hypoparathyroidism  - Primary adrenocortical failure | c.769C>T/Unknown  p.R257X (SAND)/Unknown | ([45](#_ENREF_45)) |  |
|  | 1.5 years | N/A | N/A | - Chronic mucocutaneous candidiasis (1.5Y)  - Hypoparathyroidism (4Y)  - Adrenal insufficiency (8.5Y)  - Hypothyroidism (6Y) | c.769C>T/Unknown  p.R257X (SAND)/Unknown | ([6](#_ENREF_6)) |  |
|  | 2 years | N/A | N/A | - Chronic mucocutaneous candidiasis (2Y)  - Hypoparathyroidism (6.3Y)  - Autoimmune hepatitis (4.3Y)  - Vitiligo (6.1Y) | c.769C>T/Unknown  p.R257X (SAND)/Unknown | ([6](#_ENREF_6)) |  |
|  | 5.3 years | N/A | N/A | - Chronic mucocutaneous candidiasis (6Y)  - Hypoparathyroidism (5.3Y)  - Adrenal insufficiency (19.3Y) | c.769C>T/Unknown  p.R257X (SAND)/Unknown | ([6](#_ENREF_6)) |  |
|  | 3.3 years | N/A | N/A | - Chronic mucocutaneous candidiasis (3.3Y)  - Hypoparathyroidism (10.1Y) | c.769C>T/Unknown  p.R257X (SAND)/Unknown | ([6](#_ENREF_6)) |  |
|  | 0.6 year | N/A | N/A | - Chronic mucocutaneous candidiasis (0.6Y)  - Hypoparathyroidism (9.5Y) | c.769C>T/Unknown  p.R257X (SAND)/Unknown | ([6](#_ENREF_6)) |  |
|  | 14 years | N/A | N/A | - Hypoparathyroidism (14Y) | c.769C>T/Unknown  p.R257X (SAND)/Unknown | ([6](#_ENREF_6)) |  |
|  | 10 years | N/A | N/A | - Hypoparathyroidism (15Y)  - Vitiligo (10Y) | c.769C>T/Unknown  p.R257X (SAND)/Unknown | ([6](#_ENREF_6)) |  |
|  | 3 years | N/A | N/A | - Hypoparathyroidism (3Y)  - Growth retardation (10Y)  - Metaphyseal dysplasia (12Y) | c.769C>T/Unknown  p.R257X (SAND)/Unknown | ([6](#_ENREF_6)) |  |
|  | 6 years | N/A | N/A | - Hypoparathyroidism (6Y)  - Adrenal insufficiency (19.5Y) | c.769C>T/Unknown  p.R257X (SAND)/Unknown | ([6](#_ENREF_6)) |  |
|  | N/A | N/A | Non-consanguineous | - Chronic mucocutaneous candidiasis  - Hypoparathyroidism  - Addison's disease  - Alopecia  - Autoimmune hepatitis  - Autoimmune thyroiditis  - Keratitis  - Nail dystrophy  - Pernicious anemia  - Type 1 diabetes mellitus  - Vitiligo | c.769C>T/c.1370-1371insG  p.R257X (SAND)/p.C457fs (PHD2) | ([15](#_ENREF_15), [19](#_ENREF_19)) |  |
|  | 0.6 year | Female | Non-consanguineous | - Mucocutaneous candidiasis (0.6Y)  - Primary hypoparathyroidism (6Y)  - Preclinical Addison’s disease (9.3Y)  - Febrile seizure (0.6Y)  - Syncopes and tonic-clonic seizures (6Y)  - Transient occipital alopecia (9.3Y) | c.769 C>T/c.1214delC p.R257X (SAND)/p.P405fs (PRR) | ([92](#_ENREF_92)) |  |
|  | <1 year | Female | Non-consanguineous | - Mucocutaneous candidiasis (0Y) - Hypoparathyroidism (5Y) - Adrenal insufficiency (5Y)  - Dental enamel hypoplasia | c.769C>T/c.1242_1243insA p.R257X (SAND)/p.H415fs (L) | ([11](#_ENREF_11), [85](#_ENREF_85)) |  |
|  | 9 years | Female | Non-consanguineous | - Adrenal insufficiency (9Y) | c.769C>T/c.1242_1243insA p.R257X (SAND)/p.H415fs (L) | ([11](#_ENREF_11), [85](#_ENREF_85)) |  |
|  | 14 years | Female | Non-consanguineous | - Mucocutaneous candidiasis (14Y) - Adrenal insufficiency (14Y) - Nail pitting - Primary gonadal insufficiency - Vitiligo | c.769C>T/c.1242_1243insA p.R257X (SAND)/p.H415fs (L) | ([11](#_ENREF_11), [85](#_ENREF_85)) |  |
|  | 13 years | Female | Non-consanguineous | - Mucocutaneous candidiasis (13Y) - Hypoparathyroidism (13Y) - Adrenal insufficiency (13Y) - Alopecia - Autoimmune thyroid disease - Dental enamel hypoplasia - Diabetes type 1 - Primary gonadal insufficiency | c.769C>T/c.1336T>G  p.R257X (SAND)/p.C446G (PHD2) | ([11](#_ENREF_11)) |  |
|  | N/A | Female | N/A | - Candidiasis  - Autoimmune hepatitis - Enamel dental hypoplasia | c.961C>G/WT p.S278R (SAND)/WT | ([93](#_ENREF_93)) |  |
|  | 21 years | Female | Non-consanguineous | - Mucocutaneous candidiasis  - Adrenal insufficiency | c.879+1G>A/c.879+1G>A E7del/E7del (SAND) | ([11](#_ENREF_11)) |  |
|  | 43 years | Male | Non-consanguineous | - Hypoparathyroidism (43Y) - Diabetes type 1 - Vitiligo | c.879+1G>A/c.879+1G>A E7del/E7del (SAND) | ([11](#_ENREF_11)) |  |
|  | N/A | Female | Non-consanguineous | - Adrenal insufficiency  - Autoimmune thyroid disease  - Primary ovarian insufficiency | c.901G>A/WT p.V301M (PHD1)/WT | ([94](#_ENREF_94), [95](#_ENREF_95)) |  |
|  | 6.2 years | N/A | N/A | - Chronic mucocutaneous candidiasis (6.2Y) - Hypoparathyroidism (6.5Y) - Adrenal insufficiency (7.3Y) | c.901G>A/Unknown p.V301M (PHD1)/Unknown | ([6](#_ENREF_6)) |  |
|  | 4 years | Female | Non-consanguineous | - Hypoparathyroidism (4Y) | c.905G>A/WT p.C302Y (PHD1)/WT | ([94](#_ENREF_94)) |  |
|  | 4 years | Female | Non-consanguineous | - Hypoparathyroidism (4Y) | c.905G>A/WT p.C302Y (PHD1)/WT | ([94](#_ENREF_94)) |  |
|  | 5.5 years | Female | N/A | - Hypoparathyroidism (5.5Y) | c.905G>A/WT p.C302Y (PHD1)/WT | ([55](#_ENREF_55)) |  |
|  | 4 years | Female | Non-consanguineous | - Candidiasis (4Y) - Hypoparathyroidism (6Y) - Enamel hypoplasia - Facial dysmorphism - Pitted nails | c.906T>A/c.906T>A p.C302X/p.C302X (PHD1) | ([43](#_ENREF_43)) |  |
|  | 8 years | Female | N/A | - Hypoparathyroidism (5.5Y)  - Growth hormone deficiency (10Y)  - Premature ovarian failure (16Y) | c.908G>C/WT  p.R303P (PHD1)/WT | ([4](#_ENREF_4)) |  |
|  | 43 years | Female | Non-consanguineous | - Pernicious anemia  - Neuropathy | c.913G>A/WT p.G305S (PHD1)/WT | ([94](#_ENREF_94)) |  |
|  | N/A | N/A | N/A | N/A | c.931delT/Unknown p.C311fs (PHD1)/Unknown | ([23](#_ENREF_23)) |  |
|  | N/A | N/A | N/A | N/A | c.932G>A/Unknown p.C311Y (PHD1)/Unknown | ([23](#_ENREF_23)) |  |
|  | 23 years | Male | Non-consanguineous | - Chronic mucocutaneous candidiasis (26Y) - Adrenocortical insufficiency (26Y) - Enamel hypoplasia - Partial diabetes insipidus - Pernicious anemia (23Y) | c.932G>A/WT p.C311Y (PHD1)/WT | ([94](#_ENREF_94)) |  |
|  | 5 years | Female | Non-consanguineous | - Hypoparathyroidism (5Y) - Enamel hypoplasia - Pernicious anemia (35Y) - Primary ovarian insufficiency (18Y) | c.932G>A/WT p.C311Y (PHD1)/WT | ([94](#_ENREF_94)) |  |
|  | N/A | Female | Non-consanguineous | - Alopecia areata - Nail dystrophy | c.932G>A/WT p.C311Y (PHD1)/WT | ([94](#_ENREF_94)) |  |
|  | N/A | Male | Non-consanguineous | N/A | c.932G>A/WT p.C311Y (PHD1)/WT | ([94](#_ENREF_94)) |  |
|  | 13 years | Female | Non-consanguineous | - Hypoparathyroidism (13Y) - Primary ovarian insufficiency (15Y) | c.932G>A/WT p.C311Y (PHD1)/WT | ([94](#_ENREF_94)) |  |
|  | 44 years | Female | Non-consanguineous | - Blind  - Pernicious anemia (44Y) - Type 2 diabetes | c.932G>A/WT p.C311Y (PHD1)/WT | ([94](#_ENREF_94)) |  |
|  | 11 years | Male | Non-consanguineous | - Pernicious anemia (11Y) - Vitiligo (12Y) | c.932G>A/WT p.C311Y (PHD1)/WT | ([94](#_ENREF_94)) |  |
|  | 5 years | Female | Non-consanguineous | - Chronic mucocutaneous candidiasis (5Y) - Adrenocortical insufficiency  - Asplenia - Primary ovarian insufficiency | c.932G>A/c.769C>T p.C311Y (PHD1)/p.R257X (SAND) | ([94](#_ENREF_94)) |  |
|  | 5 years | Female | Non-consanguineous | - Chronic mucocutaneous candidiasis (5Y) - Hypoparathyroidism (8Y)  - Adrenocortical insufficiency  - Asplenia - Primary ovarian insufficiency | c.932G>A/c.769C>T p.C311Y (PHD1)/p.R257X (SAND) | ([94](#_ENREF_94)) |  |
|  | 17 years | Female | Non-consanguineous | - Adrenocortical insufficiency (17Y)  - Autoimmune thyroid disease (37Y) - Primary ovarian insufficiency (39Y) | c.932G>A/WT p.C311Y (PHD1)/WT | ([94](#_ENREF_94)) |  |
|  | 4 years | Male | Consanguineous | - Hypoparathyroidism (4Y)  - Malabsorption | c.958delC/c.958delC  p.L320fs/p.L320fs (PHD1) | ([96](#_ENREF_96)) |  |
|  | N/A | Male | Consanguineous | - Hypoparathyroidism | c.958delC/c.958delC  p.L320fs/p.L320fs (PHD1) | ([96](#_ENREF_96)) |  |
|  | 1.5-5years | Female | N/A | - Hypoparathyroidism - Addison’s disease  - Asthma - Chronic active hepatitis (3Y)  - Chronic malabsorption  - Nephrolithiasis  - Vitiligo | c.967_979del13bp/c.967_979del13bp p.L323fs/p.L323fs (PHD1) | ([51](#_ENREF_51)) |  |
|  | 3 years | Female | N/A | - Severe hypocalcemia (3Y)  - Alopecia  - Blepharospasm (3Y)  - Chronic urticaria (12Y)  - Hypothyroidism  - Intermittent chronic diarrhea (3Y)  - Keratoconjunctivitis (3Y)  - Premature ovarian failure  - Tooth enamel hypoplasia  - Vitiligo | c.967_979del13bp/c.967_979del13bp p.L323fs/p.L323fs (PHD1) | ([97](#_ENREF_97)) |  |
|  | N/A | N/A | Non-consanguineous | N/A | c.967_979del13bp/c.967_979del13bp p.L323fs/p.L323fs (PHD1) | ([61](#_ENREF_61)) |  |
|  | N/A | N/A | Non-consanguineous | N/A | c.967_979del13bp/c.967_979del13bp p.L323fs/p.L323fs (PHD1) | ([61](#_ENREF_61)) |  |
|  | N/A | N/A | Non-consanguineous | N/A | c.967_979del13bp/c.967_979del13bp p.L323fs/p.L323fs (PHD1) | ([61](#_ENREF_61)) |  |
|  | N/A | N/A | Non-consanguineous | N/A | c.967_979del13bp/c.967_979del13bp p.L323fs/p.L323fs (PHD1) | ([61](#_ENREF_61)) |  |
|  | N/A | N/A | Non-consanguineous | N/A | c.967_979del13bp/c.967_979del13bp p.L323fs/p.L323fs (PHD1) | ([61](#_ENREF_61)) |  |
|  | N/A | N/A | Non-consanguineous | N/A | c.967_979del13bp/c.967_979del13bp p.L323fs/p.L323fs (PHD1) | ([61](#_ENREF_61)) |  |
|  | N/A | N/A | Non-consanguineous | N/A | c.967_979del13bp/c.967_979del13bp p.L323fs/p.L323fs (PHD1) | ([61](#_ENREF_61)) |  |
|  | N/A | N/A | Non-consanguineous | N/A | c.967_979del13bp/c.967_979del13bp p.L323fs/p.L323fs (PHD1) | ([61](#_ENREF_61)) |  |
|  | N/A | N/A | Non-consanguineous | N/A | c.967_979del13bp/c.967_979del13bp p.L323fs/p.L323fs (PHD1) | ([61](#_ENREF_61)) |  |
|  | N/A | N/A | Non-consanguineous | N/A | c.967_979del13bp/c.967_979del13bp p.L323fs/p.L323fs (PHD1) | ([61](#_ENREF_61)) |  |
|  | 14 years | Female | Consanguineous | - Hypoparathyroidism (14Y) - Premature ovarian insufficiency (28Y) | c.967_979del13bp/c.967_979del13bp p.L323fs/p.L323fs (PHD1) | ([98](#_ENREF_98)) |  |
|  | 14 years | Female | Consanguineous | - Hypoparathyroidism (14Y) | c.967_979del13bp/c.967_979del13bp p.L323fs/p.L323fs (PHD1) | ([98](#_ENREF_98)) |  |
|  | 12 years | Male | N/A | - Addison’s disease (12Y) | c.967_979del13bp/c.967_979del13bp p.L323fs/p.L323fs (PHD1) | ([99](#_ENREF_99)) |  |
|  | 2 years | Female | Consanguineous | - Candidiasis (4Y) - Hypoparathyroidism (3Y) - Malabsorption - Primary hypothyroidism  - Type 1 diabetes (2Y) | c.967_979del13bp/c.967_979del13bp p.L323fs/p.L323fs (PHD1) | ([43](#_ENREF_43)) |  |
|  | 5 years | Female | Non-consanguineous | - Candidiasis (11Y) - Hypoparathyroidism (5Y) - Addison's disease (6Y) | c.967_979del13bp/c.967_979del13bp p.L323fs/p.L323fs (PHD1) | ([43](#_ENREF_43)) |  |
|  | 5 years | Female | Non-consanguineous | - Addison's disease (5Y) | c.967_979del13bp/c.967_979del13bp p.L323fs/p.L323fs (PHD1) | ([43](#_ENREF_43)) |  |
|  | 9 years | Female | Consanguineous (1^st^ degree cousins) | - Candidiasis (9Y) - Hypoparathyroidism (12Y) - Addison's disease (11Y) | c.967_979del13bp/c.967_979del13bp p.L323fs/p.L323fs (PHD1) | ([43](#_ENREF_43)) |  |
|  | 4.8 years | Male | N/A | - Candidiasis (6.2Y)  - Hypocalcemia (4.8Y) - Mineralocorticoid deficiency (11.3Y) - Alopecia (10.2Y)  - Dental (8.2Y)  - Visual problems (5.8Y) | c.967_979del13bp/c.967_979del13bp p.L323fs/p.L323fs (PHD1) | ([100](#_ENREF_100)) |  |
|  | 1.7 years | Female | N/A | - Candidiasis (2.5Y)  - Hypocalcemia (1.7Y)  - Mineralocorticoid deficiency (5.2Y) - Visual problems (6.3Y) - Vitiligo (3.1Y) | c.967_979del13bp/c.967_979del13bp p.L323fs/p.L323fs (PHD1) | ([100](#_ENREF_100)) |  |
|  | 1 year | Male | N/A | - Candidiasis (1.7Y) - Urticaria (1Y) | c.967_979del13bp/c.967_979del13bp p.L323fs/p.L323fs (PHD1) | ([100](#_ENREF_100)) |  |
|  | 2.9 years | Male | N/A | - Candidiasis (11.6Y) - Hypocalcemia (2.9Y)  - Mineralocorticoid deficiency (6.4Y) - Glucocorticoid deficiency (6.4Y) - Nephritis/renal disease (14.2Y) - Vitiligo (13Y) | c.967_979del13bp/c.967_979del13bp p.L323fs/p.L323fs (PHD1) | ([100](#_ENREF_100)) |  |
|  | 2.9 years | Female | N/A | - Candidiasis (2.9Y)  - Hypocalcemia (2.9Y) - Mineralocorticoid deficiency (7.3Y)  - Glucocorticoid deficiency (7.3Y)  - Dental (6Y)  - Nephritis/renal disease (16.2Y) - Pancreatic insufficiency (5.4Y)  - Primary ovarian failure (15.6Y) - Type 1 diabetes (11.8Y) - Visual problems (11.1Y) - Vitiligo (11.1Y) | c.967_979del13bp/c.967_979del13bp p.L323fs/p.L323fs (PHD1) | ([100](#_ENREF_100)) |  |
|  | 5 years | Male | N/A | - Hypocalcemia (5.5Y) - Mineralocorticoid deficiency (8Y) - Asthma (4.8Y)  - GH deficiency (8Y) | c.967_979del13bp/c.967_979del13bp p.L323fs/p.L323fs (PHD1) | ([100](#_ENREF_100)) |  |
|  | 1 year | Male | N/A | - Candidiasis (5.3Y)  - Hypocalcemia (6.3Y) - Mineralocorticoid deficiency (4.5Y) - Glucocorticoid deficiency (4.5Y)  - Hepatitis (1.1Y) - Visual problems (8.8Y) | c.967_979del13bp/c.967_979del13bp p.L323fs/p.L323fs (PHD1) | ([100](#_ENREF_100)) |  |
|  | 2.4 years | Female | N/A | - Candidiasis (3.5Y)  - Hypocalcemia (2.4Y) - Glucocorticoid deficiency (14Y) - Dental (5.7Y) - Nephritis/renal disease (15.9Y)  - Pancreatic insufficiency (8Y)  - Pernicious anemia (10Y) - Type 1 diabetes (7.3Y)  - Vitiligo (7.3Y) | c.967_979del13bp/c.967_979del13bp p.L323fs/p.L323fs (PHD1) | ([100](#_ENREF_100)) |  |
|  | N/A | Male | N/A | - Mucocutaneous candidiasis  - Hypoparathyroidism - Addison disease - Alopecia - Hashimoto’s thyroiditis - Vitiligo | c.967_979del13bp/c.967_979del13bp p.L323fs/p.L323fs (PHD1) | ([58](#_ENREF_58)) |  |
|  | N/A | Male | N/A | - Mucocutaneous Candidiasis  - Hypoparathyroidism - Addison disease - Chronic active hepatitis - Hypogonadism | c.967_979del13bp/c.967_979del13bp p.L323fs/p.L323fs (PHD1) | ([58](#_ENREF_58)) |  |
|  | N/A | Female | N/A | - Mucocutaneous Candidiasis  - Hypoparathyroidism - Addison disease | c.967_979del13bp/c.967_979del13bp p.L323fs/p.L323fs (PHD1) | ([58](#_ENREF_58)) |  |
|  | N/A | Female | N/A | - Mucocutaneous Candidiasis  - Hypoparathyroidism - Addison disease  - Chronic active hepatitis | c.967_979del13bp/c.967_979del13bp p.L323fs/p.L323fs (PHD1) | ([58](#_ENREF_58)) |  |
|  | N/A | Male | N/A | - Hypoparathyroidism  - Addison disease  - Alopecia - Hashimoto’s thyroiditis | c.967_979del13bp/c.967_979del13bp p.L323fs/p.L323fs (PHD1) | ([58](#_ENREF_58)) |  |
|  | N/A | Male | N/A | - Mucocutaneous Candidiasis  - Hypoparathyroidism - Addison disease - Alopecia - Chronic active hepatitis | c.967_979del13bp/c.967_979del13bp p.L323fs/p.L323fs (PHD1) | ([58](#_ENREF_58)) |  |
|  | N/A | Female | N/A | - Mucocutaneous candidiasis  - Hypoparathyroidism - Alopecia - Malabsorption | c.967_979del13bp/c.967_979del13bp p.L323fs/p.L323fs (PHD1) | ([58](#_ENREF_58)) |  |
|  | N/A | Female | N/A | - Mucocutaneous candidiasis  - Hypoparathyroidism - Addison disease | c.967_979del13bp/c.967_979del13bp p.L323fs/p.L323fs (PHD1) | ([58](#_ENREF_58)) |  |
|  | N/A | Female | N/A | - Mucocutaneous candidiasis  - Hypoparathyroidism - Addison disease | c.967_979del13bp/c.967_979del13bp p.L323fs/p.L323fs (PHD1) | ([58](#_ENREF_58)) |  |
|  | 4 years | Male | Non-consanguineous | - Mucocutaneous candidiasis (4Y) - Hypoparathyroidism (4Y) - Adrenal insufficiency (4Y) - Dental enamel hypoplasia - Malabsorption | c.967_979del13bp/c.967_979del13bp p.L323fs/p.L323fs (PHD1) | ([11](#_ENREF_11), [85](#_ENREF_85)) |  |
|  | 4 years | Male | Non-consanguineous | - Mucocutaneous candidiasis (9Y) - Hypoparathyroidism (4Y) - Adrenal insufficiency (11Y) - Dental enamel hypoplasia | c.967_979del13bp/c.967_979del13bp p.L323fs/p.L323fs (PHD1) | ([11](#_ENREF_11), [85](#_ENREF_85)) |  |
|  | 9 years | Male | Non-consanguineous | - Mucocutaneous candidiasis  - Hypoparathyroidism (9Y) - Adrenal insufficiency (12Y) - Dental enamel hypoplasia | c.967_979del13bp/c.967_979del13bp p.L323fs/p.L323fs (PHD1) | ([11](#_ENREF_11), [85](#_ENREF_85)) |  |
|  | 4 years | Female | Non-consanguineous | - Mucocutaneous candidiasis  - Hypoparathyroidism (4Y)  - Autoimmune thyroid disease  - Dental enamel hypoplasia  - Vitiligo | c.967_979del13bp/c.967_979del13bp p.L323fs/p.L323fs (PHD1) | ([11](#_ENREF_11), [85](#_ENREF_85)) |  |
|  | 4 years | Male | Non-consanguineous | - Mucocutaneous candidiasis (26Y)  - Hypoparathyroidism (4Y)  - Adrenal insufficiency (4Y)  - Alopecia  - Dental enamel hypoplasia  - Oral squamous cell carcinoma | c.967_979del13bp/c.967_979del13bp p.L323fs/p.L323fs (PHD1) | ([11](#_ENREF_11), [85](#_ENREF_85)) |  |
|  | 20 years | Male | Non-consanguineous | - Mucocutaneous candidiasis (30Y)  - Hypoparathyroidism (27Y)  - Adrenal insufficiency (20Y) | c.967_979del13bp/c.967_979del13bp p.L323fs/p.L323fs (PHD1) | ([11](#_ENREF_11), [85](#_ENREF_85)) |  |
|  | 10 years | Female | Non-consanguineous | - Mucocutaneous candidiasis (10Y)  - Hypoparathyroidism (10Y) - Adrenal insufficiency (13Y) - Primary gonadal insufficiency | c.967_979del13bp/c.967_979del13bp p.L323fs/p.L323fs (PHD1) | ([11](#_ENREF_11), [85](#_ENREF_85)) |  |
|  | 1 year | Female | Non-consanguineous | - Mucocutaneous candidiasis (1Y)  - Hypoparathyroidism (15Y)  - Adrenal insufficiency (17Y)  - Primary gonadal insufficiency | c.967_979del13bp/c.967_979del13bp p.L323fs/p.L323fs (PHD1) | ([11](#_ENREF_11), [85](#_ENREF_85)) |  |
|  | 5 years | Male | Non-consanguineous | - Mucocutaneous candidiasis (5Y) - Hypoparathyroidism (20Y) - Adrenal insufficiency (17Y)  - Alopecia - Keratopathy - Vitiligo | c.967_979del13bp/c.967_979del13bp p.L323fs/p.L323fs (PHD1) | ([11](#_ENREF_11), [85](#_ENREF_85)) |  |
|  | 12 years | Male | Non-consanguineous | - Adrenal insufficiency (12Y) | c.967_979del13bp/c.967_979del13bp p.L323fs/p.L323fs (PHD1) | ([11](#_ENREF_11)) |  |
|  | N/A | Male | Consanguineous | - Mucocutaneous candidiasis - Hypoparathyroidism | c.967_979del13bp/c.967_979del13bp p.L323fs/p.L323fs (PHD1) | ([11](#_ENREF_11)) |  |
|  | 1 year | Female | Non-consanguineous | - Mucocutaneous candidiasis (1Y) - Hypoparathyroidism (2.4Y) - Addison’s disease (3Y) - Keratitis-uveitis (10Y)  - Malabsorption  - Mild anemia (9.5Y)  - Primary gonadal failure (16.9Y) - Reversible metaphyseal dysplasia (9.5Y) | c.967_979del13bp/c.967_979del13bp p.L323fs/p.L323fs (PHD1) | ([101](#_ENREF_101)) |  |
|  | N/A | Male | Non-consanguineous | - Mucocutaneous candidiasis  - Hypoparathyroidism  - Addison’s disease | c.967_979del13bp/c.967_979del13bp p.L323fs/p.L323fs (PHD1) | ([101](#_ENREF_101)) |  |
|  | N/A | Male | Non-consanguineous | - Mucocutaneous candidiasis  - Hypoparathyroidism  - Addison’s disease  - Very mild metaphyseal sclerosis | c.967_979del13bp/c.967_979del13bp p.L323fs/p.L323fs (PHD1) | ([101](#_ENREF_101)) |  |
|  | N/A | Male | N/A | - Chronic mucocutaneous candidiasis  - Hypoparathyroidism  - Adrenal insufficiency | c.967_979del13bp/c.967_979del13bp p.L323fs/p.L323fs (PHD1) | ([102](#_ENREF_102)) |  |
|  | N/A | Male | N/A | - Chronic mucocutaneous candidiasis | c.967_979del13bp/c.967_979del13bp p.L323fs/p.L323fs (PHD1) | ([102](#_ENREF_102)) |  |
|  | N/A | Male | N/A | - Chronic mucocutaneous candidiasis  - Hypoparathyroidism  - Adrenal insufficiency  - Enamel dysplasia | c.967_979del13bp/c.967_979del13bp p.L323fs/p.L323fs (PHD1) | ([102](#_ENREF_102)) |  |
|  | N/A | Male | N/A | - Chronic mucocutaneous candidiasis  - Hypoparathyroidism  - Adrenal insufficiency  - Hypothyroidism | c.967_979del13bp/c.967_979del13bp p.L323fs/p.L323fs (PHD1) | ([102](#_ENREF_102)) |  |
|  | N/A | Female | N/A | - Chronic mucocutaneous candidiasis  - Hypoparathyroidism  - Autoimmune hepatitis  - Malabsorption  - Pancreatic insufficiency | c.967_979del13bp/c.967_979del13bp p.L323fs/p.L323fs (PHD1) | ([102](#_ENREF_102)) |  |
|  | N/A | Male | N/A | - Chronic mucocutaneous candidiasis  - Autoimmune hepatitis | c.967_979del13bp/c.967_979del13bp p.L323fs/p.L323fs (PHD1) | ([102](#_ENREF_102)) |  |
|  | N/A | N/A | N/A | N/A | c.967_979del13bp/c.967_979del13bp p.L323fs/p.L323fs (PHD1) | ([87](#_ENREF_87)) |  |
|  | N/A | N/A | N/A | N/A | c.967_979del13bp/c.967_979del13bp p.L323fs/p.L323fs (PHD1) | ([87](#_ENREF_87)) |  |
|  | N/A | N/A | N/A | N/A | c.967_979del13bp/c.967_979del13bp p.L323fs/p.L323fs (PHD1) | ([87](#_ENREF_87)) |  |
|  | N/A | N/A | N/A | - Hypoparathyroidism - Addison's disease | c.967_979del13bp/c.967_979del13bp p.L323fs/p.L323fs (PHD1) | ([84](#_ENREF_84)) |  |
|  | N/A | Male | Non-consanguineous | - Chronic mucocutaneous candidiasis  - Hypoparathyroidism  - Adrenal failure | c.967_979del13bp/c.967_979del13bp p.L323fs/p.L323fs (PHD1) | ([27](#_ENREF_27), [28](#_ENREF_28)) |  |
|  | N/A | Female | Non-consanguineous | - Chronic mucocutaneous candidiasis  - Hypoparathyroidism  - Adrenal failure | c.967_979del13bp/c.967_979del13bp p.L323fs/p.L323fs (PHD1) | ([27](#_ENREF_27), [28](#_ENREF_28)) |  |
|  | N/A | Female | Non-consanguineous | - Chronic mucocutaneous candidiasis  - Hypoparathyroidism  - Adrenal failure | c.967_979del13bp/c.967_979del13bp p.L323fs/p.L323fs (PHD1) | ([27](#_ENREF_27), [28](#_ENREF_28)) |  |
|  | N/A | Female | Non-consanguineous | - Chronic mucocutaneous candidiasis  - Hypoparathyroidism | c.967_979del13bp/c.967_979del13bp p.L323fs/p.L323fs (PHD1) | ([27](#_ENREF_27), [28](#_ENREF_28)) |  |
|  | N/A | Male | Non-consanguineous | - Chronic mucocutaneous candidiasis  - Hypoparathyroidism  - Adrenal failure - Alopecia totalis  - Hypothyroidism  - Keratopathy  - Type 1 diabetes | c.967_979del13bp/c.967_979del13bp p.L323fs/p.L323fs (PHD1) | ([27](#_ENREF_27), [28](#_ENREF_28)) |  |
|  | N/A | Female | Non-consanguineous | - Chronic mucocutaneous candidiasis  - Hypoparathyroidism  - Adrenal failure  - Primary gonadal failure | c.967_979del13bp/c.967_979del13bp p.L323fs/p.L323fs (PHD1) | ([27](#_ENREF_27), [28](#_ENREF_28)) |  |
|  | N/A | Male | Non-consanguineous | - Chronic mucocutaneous candidiasis  - Hypoparathyroidism  - Adrenal failure - Pernicious anemia  - Primary gonadal failure  - Type 1 diabetes | c.967_979del13bp/c.967_979del13bp p.L323fs/p.L323fs (PHD1) | ([27](#_ENREF_27), [28](#_ENREF_28)) |  |
|  | N/A | Male | Non-consanguineous | - Chronic mucocutaneous candidiasis  - Hypoparathyroidism  - Adrenal failure - Alopecia totalis  - Intestinal malabsorption  - Type 1 diabetes | c.967_979del13bp/c.967_979del13bp p.L323fs/p.L323fs (PHD1) | ([27](#_ENREF_27), [28](#_ENREF_28)) |  |
|  | N/A | Male | Non-consanguineous | - Chronic mucocutaneous candidiasis  - Adrenal failure | c.967_979del13bp/c.967_979del13bp p.L323fs/p.L323fs (PHD1) | ([27](#_ENREF_27), [28](#_ENREF_28)) |  |
|  | N/A | Male | Non-consanguineous | - Chronic mucocutaneous candidiasis  - Hypoparathyroidism  - Adrenal failure | c.967_979del13bp/c.967_979del13bp p.L323fs/p.L323fs (PHD1) | ([27](#_ENREF_27), [28](#_ENREF_28)) |  |
|  | N/A | Male | Non-consanguineous | - Chronic mucocutaneous candidiasis  - Hypoparathyroidism  - Adrenal failure  - Alopecia totalis - Intestinal malabsorption  - Pernicious anemia  - Primary gonadal failure | c.967_979del13bp/c.967_979del13bp p.L323fs/p.L323fs (PHD1) | ([27](#_ENREF_27), [28](#_ENREF_28)) |  |
|  | N/A | Female | Non-consanguineous | - Chronic mucocutaneous candidiasis  - Hypoparathyroidism  - Adrenal failure  - Alopecia totalis  - Intestinal malabsorption  - Keratopathy  - Pernicious anemia  - Primary gonadal failure  - Type 1 diabetes | c.967_979del13bp/c.967_979del13bp p.L323fs/p.L323fs (PHD1) | ([27](#_ENREF_27), [28](#_ENREF_28)) |  |
|  | 2 years | Female | N/A | - Chronic mucocutaneous candidiasis  - Hypoparathyroidism (2Y)  - Alopecia areata  - Insulin-dependent diabetes mellitus  - Pernicious anemia | c.967_979del13bp/c.967_979del13bp p.L323fs/p.L323fs (PHD1) | ([59](#_ENREF_59)) |  |
|  | 6 years | Male | N/A | - Chronic mucocutaneous candidiasis  - Hypoparathyroidism (6Y)  - Alopecia areata  - Chronic active hepatitis  - Chronic diarrhea | c.967_979del13bp/c.967_979del13bp p.L323fs/p.L323fs (PHD1) | ([59](#_ENREF_59)) |  |
|  | 6 years | Male | N/A | - Chronic mucocutaneous candidiasis  - Hypoparathyroidism (8Y)  - Alopecia areata | c.967_979del13bp/c.967_979del13bp p.L323fs/p.L323fs (PHD1) | ([59](#_ENREF_59)) |  |
|  | 8 years | Female | N/A | - Chronic mucocutaneous candidiasis  - Hypoparathyroidism (7Y)  - Adrenal insufficiency  - Alopecia areata  - Chronic diarrhea  - Ovarian failure | c.967_979del13bp/c.967_979del13bp p.L323fs/p.L323fs (PHD1) | ([59](#_ENREF_59)) |  |
|  | 7 years | Female | N/A | - Chronic mucocutaneous candidiasis  - Hypoparathyroidism  - Adrenal insufficiency  - Ophthalmological features  - Ovarian failure | c.967_979del13bp/c.967_979del13bp p.L323fs/p.L323fs (PHD1) | ([59](#_ENREF_59)) |  |
|  | 1 year | Male | N/A | - Chronic mucocutaneous candidiasis (1Y)  - Adrenal insufficiency (mineralocorticoid only)  - Chronic active hepatitis (1Y) | c.967_979del13bp/c.967_979del13bp p.L323fs/p.L323fs (PHD1) | ([59](#_ENREF_59)) |  |
|  | 3 years | Female | N/A | - Chronic mucocutaneous candidiasis  - Hypoparathyroidism (3Y)  - Chronic diarrhea  - Ophthalmological features | c.967_979del13bp/c.967_979del13bp p.L323fs/p.L323fs (PHD1) | ([59](#_ENREF_59)) |  |
|  | N/A | Male | N/A | - Chronic mucocutaneous candidiasis | c.967_979del13bp/c.967_979del13bp p.L323fs/p.L323fs (PHD1) | ([59](#_ENREF_59)) |  |
|  | 6 years | Male | N/A | - Chronic mucocutaneous candidiasis  - Adrenal insufficiency (6Y) | c.967_979del13bp/c.967_979del13bp p.L323fs/p.L323fs (PHD1) | ([59](#_ENREF_59)) |  |
|  | 3 years | Male | N/A | - Chronic mucocutaneous candidiasis  - Hypoparathyroidism  - Adrenal insufficiency - Chronic active hepatitis (3Y) | c.967_979del13bp/c.967_979del13bp p.L323fs/p.L323fs (PHD1) | ([59](#_ENREF_59)) |  |
|  | N/A | Female | N/A | - Chronic mucocutaneous candidiasis | c.967_979del13bp/c.967_979del13bp p.L323fs/p.L323fs (PHD1) | ([59](#_ENREF_59)) |  |
|  | 3 years | Female | N/A | - Chronic mucocutaneous candidiasis  - Hypoparathyroidism (3Y)  - Adrenal insufficiency  - Alopecia areata | c.967_979del13bp/c.967_979del13bp p.L323fs/p.L323fs (PHD1) | ([59](#_ENREF_59)) |  |
|  | 8 years | Female | N/A | - Chronic mucocutaneous candidiasis  - Hypoparathyroidism (8Y)  - Adrenal insufficiency | c.967_979del13bp/c.967_979del13bp p.L323fs/p.L323fs (PHD1) | ([59](#_ENREF_59)) |  |
|  | 2 years | Female | N/A | - Chronic mucocutaneous candidiasis  - Hypoparathyroidism (2Y)  - Adrenal insufficiency  - Ovarian failure | c.967_979del13bp/c.967_979del13bp p.L323fs/p.L323fs (PHD1) | ([59](#_ENREF_59)) |  |
|  | 6 years | Female | N/A | - Chronic mucocutaneous candidiasis  - Hypoparathyroidism (6Y)  - Adrenal insufficiency  - Hypothyroidism  - Pernicious anemia | c.967_979del13bp/c.967_979del13bp p.L323fs/p.L323fs (PHD1) | ([59](#_ENREF_59)) |  |
|  | 2 years | Female | N/A | - Chronic mucocutaneous candidiasis  - Hypoparathyroidism (2Y)  - Adrenal insufficiency  - Ovarian failure | c.967_979del13bp/c.967_979del13bp p.L323fs/p.L323fs (PHD1) | ([59](#_ENREF_59)) |  |
|  | N/A | N/A | N/A | N/A | c.967_979del13bp/c.967_979del13bp p.L323fs/p.L323fs (PHD1) | ([23](#_ENREF_23)) |  |
|  | N/A | N/A | N/A | N/A | c.967_979del13bp/c.967_979del13bp p.L323fs/p.L323fs (PHD1) | ([23](#_ENREF_23)) |  |
|  | N/A | N/A | N/A | N/A | c.967_979del13bp/c.967_979del13bp p.L323fs/p.L323fs (PHD1) | ([23](#_ENREF_23)) |  |
|  | N/A | N/A | N/A | N/A | c.967_979del13bp/c.967_979del13bp p.L323fs/p.L323fs (PHD1) | ([23](#_ENREF_23)) |  |
|  | N/A | N/A | N/A | N/A | c.967_979del13bp/c.967_979del13bp p.L323fs/p.L323fs (PHD1) | ([23](#_ENREF_23)) |  |
|  | N/A | N/A | N/A | N/A | c.967_979del13bp/c.967_979del13bp p.L323fs/p.L323fs (PHD1) | ([23](#_ENREF_23)) |  |
|  | N/A | N/A | N/A | N/A | c.967_979del13bp/c.967_979del13bp p.L323fs/p.L323fs (PHD1) | ([23](#_ENREF_23)) |  |
|  | N/A | N/A | N/A | N/A | c.967_979del13bp/c.967_979del13bp p.L323fs/p.L323fs (PHD1) | ([23](#_ENREF_23)) |  |
|  | N/A | N/A | N/A | N/A | c.967_979del13bp/c.967_979del13bp p.L323fs/p.L323fs (PHD1) | ([23](#_ENREF_23)) |  |
|  | N/A | N/A | N/A | N/A | c.967_979del13bp/c.967_979del13bp p.L323fs/p.L323fs (PHD1) | ([23](#_ENREF_23)) |  |
|  | N/A | N/A | N/A | N/A | c.967_979del13bp/c.967_979del13bp p.L323fs/p.L323fs (PHD1) | ([23](#_ENREF_23)) |  |
|  | N/A | N/A | N/A | N/A | c.967_979del13bp/c.967_979del13bp p.L323fs/p.L323fs (PHD1) | ([23](#_ENREF_23)) |  |
|  | N/A | N/A | N/A | N/A | c.967_979del13bp/c.967_979del13bp p.L323fs/p.L323fs (PHD1) | ([23](#_ENREF_23)) |  |
|  | N/A | N/A | N/A | N/A | c.967_979del13bp/c.967_979del13bp p.L323fs/p.L323fs (PHD1) | ([23](#_ENREF_23)) |  |
|  | 0.5 year | Male | Non-consanguineous | - Candidiasis (0.5Y) - Addison’s disease (7Y)  - Alopecia (11Y) - Gastritis (12Y)  - Hypophysitis (6Y) - Malabsorption (6Y) | c.967_979del13bp/c.967_979del13bp p.L323fs/p.L323fs (PHD1) | ([91](#_ENREF_91)) |  |
|  | 11 years | Male | Non-consanguineous | - Candidiasis (15Y)  - Addison’s disease (12Y)  - Alopecia (14Y)  - Malabsorption (11Y) | c.967_979del13bp/c.967_979del13bp p.L323fs/p.L323fs (PHD1) | ([91](#_ENREF_91)) |  |
|  | 1 year | Male | Non-consanguineous | - Candidiasis (1Y) - Hypoparathyroidism (8Y) - Addison’s disease (11Y)  - Alopecia (16Y) - Hepatitis (11Y) | c.967_979del13bp/c.967_979del13bp p.L323fs/p.L323fs (PHD1) | ([91](#_ENREF_91)) |  |
|  | 1 year | Male | Non-consanguineous | - Candidiasis (1Y) - Hypoparathyroidism (10Y) - Addison’s disease (10Y) | c.967_979del13bp/c.967_979del13bp p.L323fs/p.L323fs (PHD1) | ([91](#_ENREF_91)) |  |
|  | 12 years | Male | Consanguineous | - Candidiasis (13Y) - Alopecia (15Y) - Keratitis (12Y) | c.967_979del13bp/c.967_979del13bp p.L323fs/p.L323fs (PHD1) | ([91](#_ENREF_91)) |  |
|  | 12 years | Female | Consanguineous | - Candidiasis (12Y) - Hypoparathyroidism (12Y) - Addison’s disease (23Y)  - Alopecia (12Y)  - Gastritis (24Y)  - Hemolytic anemia (24Y) - Hypogonadism (26Y) | c.967_979del13bp/c.967_979del13bp p.L323fs/p.L323fs (PHD1) | ([91](#_ENREF_91)) |  |
|  | 4 years | Male | Non-consanguineous | - Candidiasis (7Y) - Hypoparathyroidism (4Y) - Addison’s disease (8Y)  - Alopecia (5Y) - Autoimmune thyroiditis (8Y) - Keratitis - Large granular lymphocytic leukemia (27Y) | c.967_979del13bp/c.967_979del13bp p.L323fs/p.L323fs (PHD1) | ([91](#_ENREF_91)) |  |
|  | 4 years | Female | Non-consanguineous | - Candidiasis (8Y) - Hypoparathyroidism (4Y) - Addison’s disease (19Y)  - Alopecia (12Y)  - Diabetes mellitus (34Y)  - Gastritis (29Y)  - Hepatitis (35Y) - Hypogonadism (15Y)  - Keratitis (34Y) - Vitiligo (12Y) | c.967_979del13bp/c.967_979del13bp p.L323fs/p.L323fs (PHD1) | ([91](#_ENREF_91)) |  |
|  | 6 years | Female | Non-consanguineous | - Chronic candidiasis (6Y) - Chronic hypoparathyroidism (10y) - Addison’s disease (34Y) - Alopecia - Cancer of the lip - Vitiligo | c.967_979del13bp/c.967_979del13bp p.L323fs/p.L323fs (PHD1) | ([36](#_ENREF_36)) |  |
|  | 1 year | Female | Non-consanguineous | - Chronic candidiasis (1Y) - Chronic hypoparathyroidism (19y) - Addison’s disease (31Y) - Alopecia - Autoimmune hepatitis - Arthrosis - Premature ovarian failure | c.967_979del13bp/c.967_979del13bp p.L323fs/p.L323fs (PHD1) | ([36](#_ENREF_36)) |  |
|  | N/A | N/A | N/A | - Mucocutaneous candidiasis - Hypoparathyroidism  - Addison’s disease  - Alopecia  - Hypothyroidism  - Keratoconjunctivitis | c.967_979del13bp/c.967_979del13bp p.L323fs/p.L323fs (PHD1) | ([10](#_ENREF_10)) |  |
|  | 12 years | Male | Non-consanguineous | - Hypoparathyroidism - Addison’s disease - Psoriasis vulgaris | c.967_979del13bp/c.967_979del13bp p.L323fs/p.L323fs (PHD1) | ([15](#_ENREF_15), [103](#_ENREF_103)) |  |
|  | 6 years | Male | N/A | - Hypoparathyroidism (6Y) - Addison’s disease (6Y) - Dental enamel hypoplasia - Keratoconjunctivitis | c.967_979del13bp/c.967_979del13bp p.L323fs/p.L323fs (PHD1) | ([55](#_ENREF_55)) |  |
|  | 0.2 year | Female | N/A | - Chronic mucocutaneous candidiasis (2.9Y) - Hypoparathyroidism (2.2Y) - Addison’s disease (4.7Y) - Asplenia (2.9Y)  - Autoimmune hepatitis (3.3Y) - Retinal dystrophy (0.2Y) | c.967_979del13bp/c.274C>T p.L323fs (PHD1)/p.R92W (HSR/CARD) | ([55](#_ENREF_55)) |  |
|  | 7 years | Male | Non-consanguineous | - Chronic candidiasis (7Y) - Chronic hypoparathyroidism (29y) - Addison’s disease (33Y) | c.967_979del13bp/c.1033_1034delGT p.L323fs (PHD1)/p.V345fs (downstream of PHD1) | ([36](#_ENREF_36)) |  |
|  | 6 years | Male | N/A | - Mucocutaneous candidiasis  - Hypoparathyroidism (6Y)  - Addison’s disease (8Y) | c.967_979del13bp/c.62C>T  p.L323fs (PHD1)/p.A21V (HSR/CARD) | ([1](#_ENREF_1)) |  |
|  | N/A | Female | N/A | - Candidiasis  - Hypoparathyroidism  - Adrenal failure | c.967_979del13bp/c.1249delC p.L323fs (PHD1)/p.L417fs (L) It was mistakenly written K417fs in the reference | ([28](#_ENREF_28)) |  |
|  | N/A | Female | N/A | - Candidiasis  - Hypoparathyroidism  - Adrenal failure  - Hepatitis  - Hypogonadism  - Malabsorption  - Pernicious anemia | c.967_979del13bp/c.1249delC p.L323fs (PHD1)/p.L417fs (L) It was mistakenly written K417fs in the reference | ([28](#_ENREF_28)) |  |
|  | 2 years | Male | Non-consanguineous | - Candidiasis (3Y)  - Hypoparathyroidism (8Y) - Addison’s disease (2Y) - Alopecia (3Y) | c.967_979del13bp/c.769C>T p.L323fs (PHD1)/p.R257X (SAND) | ([91](#_ENREF_91)) |  |
|  | 19 years | Male | Non-consanguineous | - Candidiasis - Hypoparathyroidism - Addison’s disease (19Y)  - Alopecia  - Keratitis - Malabsorption (27Y) - Vitiligo | c.967_979del13bp/c.769C>T p.L323fs (PHD1)/p.R257X (SAND) | ([91](#_ENREF_91)) |  |
|  | N/A | Male | Non-consanguineous | - Candidiasis - Alopecia - Keratitis | c.967_979del13bp/c.769C>T p.L323fs (PHD1)/p.R257X (SAND) | ([91](#_ENREF_91)) |  |
|  | 10 years | Male | Non-consanguineous | - Candidiasis - Addison’s disease (10Y)  - Keratitis (16Y) | c.967_979del13bp/c.769C>T p.L323fs (PHD1)/p.R257X (SAND) | ([91](#_ENREF_91)) |  |
|  | N/A | N/A | Non-consanguineous | N/A | c.967_979del13bp/c.769C>T p.L323fs (PHD1)/p.R257X (SAND) | ([61](#_ENREF_61)) |  |
|  | N/A | N/A | Non-consanguineous | N/A | c.967_979del13bp/c.769C>T p.L323fs (PHD1)/p.R257X (SAND) | ([61](#_ENREF_61)) |  |
|  | N/A | N/A | Non-consanguineous | N/A | c.967_979del13bp/c.769C>T p.L323fs (PHD1)/p.R257X (SAND) | ([61](#_ENREF_61)) |  |
|  | N/A | N/A | Non-consanguineous | N/A | c.967_979del13bp/c.769C>T p.L323fs (PHD1)/p.R257X (SAND) | ([61](#_ENREF_61)) |  |
|  | N/A | N/A | Non-consanguineous | N/A | c.967_979del13bp/c.769C>T p.L323fs (PHD1)/p.R257X (SAND) | ([61](#_ENREF_61)) |  |
|  | N/A | N/A | Non-consanguineous | N/A | c.967_979del13bp/c.769C>T p.L323fs (PHD1)/p.R257X (SAND) | ([61](#_ENREF_61)) |  |
|  | N/A | N/A | Non-consanguineous | N/A | c.967_979del13bp/c.769C>T p.L323fs (PHD1)/p.R257X (SAND) | ([61](#_ENREF_61)) |  |
|  | N/A | N/A | Non-consanguineous | N/A | c.967_979del13bp/c.769C>T p.L323fs (PHD1)/p.R257X (SAND) | ([61](#_ENREF_61)) |  |
|  | N/A | N/A | Non-consanguineous | N/A | c.967_979del13bp/c.769C>T p.L323fs (PHD1)/p.R257X (SAND) | ([61](#_ENREF_61)) |  |
|  | N/A | N/A | Non-consanguineous | N/A | c.967_979del13bp/c.190_226del37 p.L323fs (PHD1)/p.S64_L75delfs (L + HSR/CARD) | ([61](#_ENREF_61)) |  |
|  | N/A | N/A | Non-consanguineous | N/A | c.967_979del13bp/c.190_226del37 p.L323fs (PHD1)/p.S64_L75delfs (L + HSR/CARD) | ([61](#_ENREF_61)) |  |
|  | N/A | N/A | Non-consanguineous | N/A | c.967_979del13bp/c.190_226del37 p.L323fs (PHD1)/p.S64_L75delfs (L + HSR/CARD) | ([61](#_ENREF_61)) |  |
|  | N/A | N/A | Non-consanguineous | N/A | c.967_979del13bp/c.1249dupC p.L323fs (PHD1)/p.L417fs (L) | ([61](#_ENREF_61)) |  |
|  | N/A | N/A | Non-consanguineous | N/A | c.967_979del13bp/c.1249dupC p.L323fs (HSR/CARD)/p.L417fs (L) | ([61](#_ENREF_61)) |  |
|  | N/A | N/A | Non-consanguineous | N/A | c.967_979del13bp/c.1616C>T p.L323fs (HSR/CARD)/p.P539L (TAD) | ([61](#_ENREF_61)) |  |
|  | N/A | N/A | Non-consanguineous | N/A | c.967_979del13bp/c.1-7_538+20del p.L323fs (PHD1)/E1-E4del (HSR/CARD, L, NLS) | ([61](#_ENREF_61)) |  |
|  | N/A | N/A | Non-consanguineous | N/A | c.967_979del13bp/WT p.L323fs (PHD1)/WT | ([61](#_ENREF_61)) |  |
|  | N/A | Female | Consanguineous | - Hypoparathyroidism (7Y) | c.967_979del13bp/c.202A>C p.L323fs (PHD1)/p.T68P (HSR/CARD) | ([98](#_ENREF_98)) |  |
|  | N/A | Male | N/A | - Chronic mucocutaneous candidiasis (1Y)  - Hypoparathyroidism (3Y)  - Malabsorption (3Y) | c.967_979del13bp/c.769C>T p.L323fs (PHD1)/p.R257X (SAND) | ([5](#_ENREF_5)) |  |
|  | N/A | Female | N/A | - Chronic mucocutaneous candidiasis (9Y)  - Hypoparathyroidism (10Y)  - Adrenal insufficiency (12Y)  - Alopecia (15Y)  - Enamel hypoplasia | c.967_979del13bp/c.769C>T p.L323fs (PHD1)/p.R257X (SAND) | ([5](#_ENREF_5)) |  |
|  | N/A | Male | N/A | - Chronic mucocutaneous candidiasis (3Y)  - Hypoparathyroidism (5Y)  - Adrenal insufficiency (8Y)  - Alopecia (8Y)  - Pernicious anemia (12Y) | c.967_979del13bp/c.1195G>C p.L323fs (PHD1)/p.A399P (PRR) | ([5](#_ENREF_5)) |  |
|  | N/A | Male | N/A | - Hypoparathyroidism (11Y)  - Adrenal insufficiency (11Y) | c.967_979del13bp/c.769C>T p.L323fs (PHD1)/p.R257X (SAND) | ([5](#_ENREF_5)) |  |
|  | N/A | N/A | N/A | - Mucocutaneous candidiasis - Addison’s disease - Alopecia - Ectodermal dystrophy - Gastritis - Hypothyroidism | c.967_979del13bp/Unknown  p.L323fs (PHD1)/Unknown | ([10](#_ENREF_10)) |  |
|  | N/A | Male | Non-consanguineous | - Mucocutaneous candidiasis - Hypoparathyroidism  - Dental enamel hypoplasia  - Nail pitting | c.967_979del13bp/c.1283_1284insA p.L323fs (PHD1)/p.L428fs (PRR) | ([85](#_ENREF_85)) |  |
|  | N/A | Male | Non-consanguineous | - Mucocutaneous candidiasis - Hypoparathyroidism  - Adrenal insufficiency  - Alopecia  - Autoimmune hepatitis | c.967_979del13bp/c.1249dupC p.L323fs (PHD1)/p.L417fs (L) | ([85](#_ENREF_85)) |  |
|  | N/A | Female | N/A | - Mucocutaneous candidiasis  - Hypoparathyroidism - Addison disease  - Alopecia - Hashimoto’s thyroiditis  - Vitiligo | c.967_979del13bp/c.769C>T p.L323fs (PHD1)/p.R257X (SAND) | ([58](#_ENREF_58)) |  |
|  | N/A | Female | N/A | - Mucocutaneous candidiasis  - Hypoparathyroidism - Addison disease | c.967_979del13bp/c.769C>T p.L323fs (PHD1)/p.R257X (SAND) | ([58](#_ENREF_58)) |  |
|  | 3 years | Male | Non-consanguineous | - Mucocutaneous candidiasis (3Y)  - Hypoparathyroidism (3Y)  - Alopecia  - Keratopathy | c.967_979del13bp/c.769C>T p.L323fs (PHD1)/p.R257X (SAND) | ([11](#_ENREF_11), [85](#_ENREF_85)) |  |
|  | 2 years | Male | Non-consanguineous | - Mucocutaneous candidiasis (2Y)  - Hypoparathyroidism (5Y) | c.967_979del13bp/c.769C>T p.L323fs (PHD1)/p.R257X (SAND) | ([11](#_ENREF_11), [85](#_ENREF_85)) |  |
|  | 8 years | Male | Non-consanguineous | - Mucocutaneous candidiasis (14Y)  - Hypoparathyroidism (8Y)  - Dental enamel hypoplasia - Nail pitting | c.967–979del13bp/c.1163_1164insA p.L323fs (PHD1)/p.M388fs (PRR) | ([11](#_ENREF_11), [85](#_ENREF_85)) |  |
|  | <1 year | Male | N/A | - Mucocutaneous candidiasis (0Y)  - Hypoparathyroidism (10Y)  - Adrenal insufficiency (10Y)  - Alopecia  - Autoimmune hepatitis  - Malabsorption | c.967_979del13bp/c.1244_1245insC p.L323fs (PHD1)/p.H415fs (L) | ([11](#_ENREF_11), [85](#_ENREF_85)) |  |
|  | 14 years | Male | N/A | - Mucocutaneous candidiasis - Adrenal insufficiency | c.967_979del13bp/c.769C>T p.L323fs (PHD1)/p.R257X (SAND) | ([11](#_ENREF_11)) |  |
|  | N/A | Male | N/A | - Chronic mucocutaneous candidiasis  - Hypoparathyroidism | c.967_979del13bp/c.977C>T p.L323fs (PHD1)/p.P326L (PHD1) | ([102](#_ENREF_102)) |  |
|  | 9 years | Female | N/A | - Recurrent candidiasis(9Y)  - Severe tetany and hypocalcemia(9Y)  - Adrenal insufﬁciency  - Chronic intermittent diarrhea(9Y)  - Chronic urticaria(13Y)  - Dystrophic nails  - Gonadal failure  - Tooth enamel hypoplasia  - Vitiligo | c.967_979del13bp/c.769C>T p.L323fs (PHD1)/p.R257X (SAND) | ([97](#_ENREF_97)) |  |
|  | N/A | N/A | N/A | N/A | c.967_979del13bp/Unknown  p.L323fs (PHD1)/Unknown | ([87](#_ENREF_87)) |  |
|  | 2 years | Male | N/A | - Hypoparathyroidism (2Y) | c.967_979del13bp/c.995+(3_5)delGAGinsTAT p.L323fs (PHD1)/E8del (PHD1) | ([104](#_ENREF_104)) |  |
|  | 5 years | Male | N/A | - Hypoparathyroidism (5Y) | c.967_979del13bp/c.995+(3_5)delGAGinsTAT p.L323fs (PHD1)/E8del (PHD1) | ([104](#_ENREF_104)) |  |
|  | 7 years | Female | N/A | - Hypoparathyroidism (7Y)  - Premature ovarian failure (33Y) | c.967_979del13bp/c.995+(3_5)delGAGinsTAT p.L323fs (PHD1)/E8del (PHD1) | ([104](#_ENREF_104)) |  |
|  | 3 years | Female | Non-consanguineous | - Chronic mucocutaneous candidiasis  - Hypoparathyroidism  - Adrenal failure  - Ankylosing spondylitis | c.967_979del13bp/c.1264delC p.L323fs (PHD1)/p.P422fs (PRR) | ([27](#_ENREF_27), [105](#_ENREF_105)) |  |
|  | N/A | Female | Non-consanguineous | - Chronic mucocutaneous candidiasis  - Hypoparathyroidism  - Adrenal failure  - Chronic autoimmune hepatitis  - Intestinal malabsorption  - Pernicious anemia  - Primary gonadal failure | c.967_979del13bp/c.1264delC p.L323fs (PHD1)/p.P422fs (PRR) | ([27](#_ENREF_27)) |  |
|  | N/A | Male | Non-consanguineous | - Chronic mucocutaneous candidiasis  - Hypoparathyroidism  - Adrenal failure  - Chronic autoimmune hepatitis  - Primary gonadal failure | c.967_979del13bp/c.44G>T p.L323fs (PHD1)/p.R15L (HSR/CARD) | ([27](#_ENREF_27)) |  |
|  | N/A | N/A | N/A | - Pernicious anemia | c.967_979del13bp/c.946C>T p.L323fs (PHD1)/p.R316W (PHD1) | ([94](#_ENREF_94)) |  |
|  | 4 years | Male | N/A | - Chronic mucocutaneous candidiasis  - Hypoparathyroidism  - Adrenal insufficiency  - Chronic active hepatitis  - Chronic diarrhea - Insulin-dependent diabetes mellitus (4Y) | c.967_979del13bp/c.290T>C p.L323fs (PHD1)/p.L97P (HSR/CARD) | ([59](#_ENREF_59)) |  |
|  | 1 year | Female | N/A | - Chronic mucocutaneous candidiasis  - Hypoparathyroidism  - Adrenal insufficiency (1Y)  - Alopecia areata  - Ovarian failure | c.967_979del13bp/Unknown  p.L323fs (PHD1)/Unknown | ([59](#_ENREF_59)) |  |
|  | 10 years | Male | N/A | - Chronic mucocutaneous candidiasis  - Hypoparathyroidism (10Y)  - Adrenal insufficiency | c.967_979del13bp/Unknown  p.L323fs (PHD1)/Unknown | ([59](#_ENREF_59)) |  |
|  | N/A | N/A | N/A | N/A | c.967_979del13bp/Unknown  p.L323fs (PHD1)/Unknown | ([23](#_ENREF_23)) |  |
|  | N/A | N/A | N/A | N/A | c.967_979del13bp/Unknown  p.L323fs (PHD1)/Unknown | ([23](#_ENREF_23)) |  |
|  | N/A | N/A | N/A | N/A | c.967_979del13bp/Unknown  p.L323fs (PHD1)/Unknown | ([23](#_ENREF_23)) |  |
|  | N/A | N/A | N/A | N/A | c.967_979del13bp/Unknown  p.L323fs (PHD1)/Unknown | ([23](#_ENREF_23)) |  |
|  | N/A | N/A | N/A | N/A | c.967_979del13bp/Unknown  p.L323fs (PHD1)/Unknown | ([23](#_ENREF_23)) |  |
|  | N/A | N/A | N/A | N/A | c.967_979del13bp/Unknown  p.L323fs (PHD1)/Unknown | ([23](#_ENREF_23)) |  |
|  | N/A | N/A | N/A | N/A | c.967_979del13bp/Unknown  p.L323fs (PHD1)/Unknown | ([23](#_ENREF_23)) |  |
|  | N/A | N/A | N/A | N/A | c.967_979del13bp/Unknown  p.L323fs (PHD1)/Unknown | ([23](#_ENREF_23)) |  |
|  | N/A | N/A | N/A | - Candidiasis - Hypoparathyroidism  - Autoimmune hepatitis - Nail dystrophy | c.967_979del13bp/c.260T>C  p.L323fs (PHD1)/p.L87P (HSR/CARD) | ([19](#_ENREF_19)) |  |
|  | N/A | N/A | N/A | - Candidiasis  - Hypoparathyroidism  - Addison's disease  - Nail dystrophy | c.967_979del13bp/c.62C>T  p.L323fs (PHD1)/p.A21V (HSR/CARD) | ([19](#_ENREF_19)) |  |
|  | N/A | N/A | N/A | - Hypoparathyroidism  - Addison's disease | c.967_979del13bp/c.769C>T p.L323fs (PHD1)/p.R257X (SAND) | ([19](#_ENREF_19)) |  |
|  | N/A | N/A | N/A | - Candidiasis  - Hypoparathyroidism  - Autoimmune hepatitis | c.967_979del13bp/c.1347C>A p.L323fs (PHD1)/p.C449X (PHD2) | ([19](#_ENREF_19)) |  |
|  | N/A | N/A | N/A | - Candidiasis  - Hypoparathyroidism  - Addison's disease  - Nail dystrophy | c.967_979del13bp/c.62C>T  p.L323fs (PHD1)/p.A21V (HSR/CARD) | ([19](#_ENREF_19)) |  |
|  | 1 year | Male | Non-consanguineous | - Chronic mucocutaneous candidiasis (1Y)  - Addison’s disease (10Y)  - Chronic hepatitis (10Y)  - Hyposplenia (17Y)  - Pernicious anemia (9Y)  - Reversible metaphyseal dysplasia (5Y)  - Type 1 diabetes mellitus (11Y) | c.967_979del13bp/c.789delC p.L323fs (PHD1)/p.G263fs (SAND) | ([101](#_ENREF_101)) |  |
|  | 10.7 years | Female | N/A | - Candidiasis and/or hypoparathyroidism  - Autoimmune adrenal failure (10.7Y) | c.967_979del13bp/c.278T>G p.L323fs (PHD1)/p.L93R (HSR/CARD) | ([106](#_ENREF_106)) |  |
|  | 13.6 years | Female | N/A | - Candidiasis and/or hypoparathyroidism  - Autoimmune adrenal failure (13.6Y) | c.967_979del13bp/c.278T>G p.L323fs (PHD1)/p.L93R (HSR/CARD) | ([106](#_ENREF_106)) |  |
|  | N/A | Female | N/A | - Chronic mucocutaneous candidiasis | c.967_979del13bp/c.967_979del13bp + c.290T>C p.L323fs (PHD1)/p.L323fs (PHD1) + p.L97P (HSR/CARD) | ([59](#_ENREF_59)) |  |
|  | Infancy | Female | Non-consanguineous | - Candidiasis (infancy)  - Hypoparathyroidism (4.6Y)  - Adrenal failure (13.6Y) - Alopecia (9Y)  - Hepatitis (11.8Y)  - Hypogonadism (13.6Y)  - Keratopathy (6Y)  - Malabsorption (7.5Y)  - Panhypopituitarism (12Y) - Type 1 diabetes mellitus (11Y) | c.967_979del13bp/c.38T>G p.L323fs (PHD1)/p.L13R (HSR/CARD) | ([28](#_ENREF_28), [107](#_ENREF_107)) |  |
|  | 29 years | Female | Non-consanguineous | - Hypoparathyroidism (29Y) | c.977C>T/c.769C>T p.P326L (PHD1)/p.R257X (SAND) | ([108](#_ENREF_108)) |  |
|  | 3 years | Female | Non-consanguineous | - Oral and vaginal candidiasis (21Y)  - Hypoparathyroidism (3Y)  - Enamel hypoplasia (14Y)  - Pernicious anemia (18Y)  - Primary ovarian failure (22Y) | c.977C>T/c.769C>T p.P326L (PHD1)/p.R257X (SAND) | ([108](#_ENREF_108)) |  |
|  | 4 years | Female | Non-consanguineous | - Hypoparathyroidism (4Y)  - Primary adrenocortical insufficiency (12Y)  - Pernicious anemia (33Y)  - Primary ovarian failure (14Y) | c.977C>T/c.769C>T p.P326L (PHD1)/p.R257X (SAND) | ([108](#_ENREF_108)) |  |
|  | 3 years | N/A | N/A | - Adrenal insufficiency (12Y)  - Enamel hypoplasia  - Glucose intolerance (15Y)  - Hearing loss (3Y)  - Pernicious anemia (5Y)  - Ptosis  - Vitiligo (9Y) | c.977C>T/c.967_979del13bp p.P326L (PHD1)/p.L323fs (PHD1) | ([5](#_ENREF_5)) |  |
|  | N/A | Female | Non-consanguineous | - Adrenal insufficiency  - Autoimmune thyroid disease  - Pernicious anemia  - Vitiligo | c.977C>T/WT p.P326L (PHD1)/WT | ([94](#_ENREF_94)) |  |
|  | 10 years | Female | Non-consanguineous | - Vitiligo (10Y) | c.977C>T/WT p.P326L (PHD1)/WT | ([94](#_ENREF_94)) |  |
|  | 7 years | Male | Non-consanguineous | - Vitiligo (7Y) | c.977C>T/WT p.P326L (PHD1)/WT | ([94](#_ENREF_94)) |  |
|  | 2 years | Male | Non-consanguineous | - Hypoparathyroidism (2Y) | c.995+(3_5)delGAGinsTAT/  c.995+(3_5)delGAGinsTAT E8del/E8del (PHD1) | ([104](#_ENREF_104)) |  |
|  | 5 years | Male | Non-consanguineous | - Hypoparathyroidism (5Y) | c.995+(3_5)delGAGinsTAT/ c.995+(3_5)delGAGinsTAT E8del/E8del (PHD1) | ([104](#_ENREF_104)) |  |
|  | 7 years | Female | Non-consanguineous | - Hypoparathyroidism (7Y)  - Premature ovarian failure (33Y) | c.995+(3_5)delGAGinsTAT/ c.995+(3_5)delGAGinsTAT E8del/E8del (PHD1) | ([104](#_ENREF_104)) |  |
|  | 0.3 year | Male | Non-consanguineous | - Mucocutaneous candidiasis (0.3Y) - Hypoparathyroidism (12.9Y) - Ectodermal dystrophy (5.5Y) | c.1064_1068dupCCCGG/ c.1064_1068dupCCCGG  p.Q358fs/p.Q358fs (PRR) | ([8](#_ENREF_8)) |  |
|  | N/A | Female | N/A | - Candidiasis  - Anemia | c.1066C>T/WT  p.R356W (PRR)/WT | ([62](#_ENREF_62)) |  |
|  | 14 years | Male | N/A | - Hyperpigmentation  - Postural hypotension | c.1066C>T/WT  p.R356W (PRR)/WT | ([109](#_ENREF_109)) |  |
|  | 5 years | Female | Consanguineous (3^rd^ degree cousins) | - Mucocutaneous candidiasis - Hypoparathyroidism - Addison’s disease  - Hypothyroidism (5Y) | c.1095+2T>A/c.1095+2T>A E9del/E9del (PHD1, PRR) | ([53](#_ENREF_53)) |  |
|  | 6 years | Female | Consanguineous | - Chronic mucocutaneous candidiasis (14Y) - Hypoparathyroidism (6Y) - Alopecia areata (7Y) - Asthma (7Y)  - Autoimmune hepatitis (19Y)  - Dental enamel hypoplasia - GH deficiency (13.5Y) - Keratopathy (17Y) - Osteoporosis  - Premature ovarian failure (19Y)  - Subclinical hypothyroidism (19Y) - Vertebral fragility fractures (19Y) | c.1095+2T>A/c.1095+2T>A E9del/E9del (PHD1, PRR) | ([110](#_ENREF_110)) |  |
|  | 1 year | Female | Consanguineous (1^st^ degree cousins) | - Chronic mucocutaneous candidiasis (1Y) - Hypoparathyroidism (4Y) - Addison disease - Keratitis - Nail dystrophy | c.1095+2T>A/c.1095+2T>A E9del/E9del (PHD1, PRR) | ([41](#_ENREF_41)) |  |
|  | 2 years | Female | Consanguineous (1^st^ degree cousins) | - Chronic mucocutaneous candidiasis (2Y) - Hypoparathyroidism (4Y) - Addison disease - Keratitis - Vitiligo | c.1095+2T>A/c.1095+2T>A E9del/E9del (PHD1, PRR) | ([41](#_ENREF_41)) |  |
|  | 7 years | Male | Consanguineous (2^nd^ degree cousins) | - Hypoparathyroidism (7Y)  - Adrenal insufficiency  - Anorexia and weight loss of 6 kg  - Beard loss (40Y)  - Bilateral cataracts (25Y)  - Calcium density spot in kidney  - Cholelithiasis (46Y)  - Chronic gastritis (46Y)  - Dyspepsia  - Dysphagia  - Glaucoma (47Y)  - Mucocutaneous hyperpigmentation  - Nausea  - Non-malignant thyroid nodule (45Y)  - Occasional paraesthesia in the extremities, weakness, musculoskeletal pain, fatigue  - Onychomycosis  - Recurrent keratitis  - Two hair loss patches (42Y) | c.1103dupC/c.1103dupC p.L370fs/p.L370fs (PRR) | ([111](#_ENREF_111)) |  |
|  | 0.1 year | Female | Non-consanguineous | - Candidiasis (0.1Y)  - Hypoparathyroidism (8Y)  - Alopecia (10Y) | c.1103_1104insC/c.1503delG p.L370fs (PRR)/p.A505fs (TAD) | ([28](#_ENREF_28)) |  |
|  | N/A | Male | Non-consanguineous | - Candidiasis | c.1103_1104insC/c.1503delG p.L370fs (PRR)/p.A505fs (TAD) | ([28](#_ENREF_28)) |  |
|  | N/A | N/A | N/A | N/A | c.1111ins4bp/c.1111ins4bp p.R371fs/p.R371fs (PRR) | ([87](#_ENREF_87)) |  |
|  | N/A | Female | N/A | - Chronic diarrhea  - Candidiasis  - Very early onset Crohn’s disease | c.1118C>T/WT  p.A373V (PRR)/WT | ([62](#_ENREF_62)) |  |
|  | N/A | N/A | N/A | N/A | c.1163_1164insA/Unknown  p.M388fs (PRR)/Unknown | ([23](#_ENREF_23)) |  |
|  | N/A | N/A | N/A | N/A | c.1163_1164insA/Unknown  p.M388fs (PRR)/Unknown | ([23](#_ENREF_23)) |  |
|  | N/A | N/A | N/A | N/A | c.1163_1164insA/Unknown  p.M388fs (PRR)/Unknown | ([23](#_ENREF_23)) |  |
|  | N/A | N/A | N/A | N/A | c.1163_1164insA/Unknown  p.M388fs (PRR)/Unknown | ([23](#_ENREF_23)) |  |
|  | N/A | N/A | N/A | N/A | c.1189delC/Unknown  p.L397fs (PRR)/Unknown | ([23](#_ENREF_23)) |  |
|  | N/A | N/A | N/A | N/A | c.1193delC/c.1193delC p.P398fs/p.P398fs (PRR) | ([23](#_ENREF_23)) |  |
|  | N/A | N/A | N/A | N/A | c.1193delC/c.1193delC p.P398fs/p.P398fs (PRR) | ([87](#_ENREF_87)) |  |
|  | 3 years | Female | Consanguineous (1^st^ degree cousins) | - Mucocutaneous candidiasis (12Y)  - Hypoparathyroidism (8Y)  - Adrenal insufficiency (8Y)  - Chronic malabsorption (19Y)  - Gingivitis (26Y)  - Grave disease (19Y)  - Hepatitis (3Y)  - Keratopathy (15Y): Superﬁcial diffuse punctate staining, Peripheral ring-like subepithelial opacities, Superﬁcial neovascularization.  - Ovaritis (20Y)  - Pernicious anemia (10Y)  - Splenic atrophy (25Y)  - Vitiligo (26Y) | c.1193delC/c.1193delC p.P398fs/p.P398fs (PRR) | ([24](#_ENREF_24)) |  |
|  | 3 years | Male | Consanguineous (1^st^ degree cousins) | - Mucocutaneous candidiasis (8Y)  - Hypoparathyroidism (8Y)  - Adrenal insufficiency (3Y)  - Chronic malabsorption (10Y)  - Diabetes (10Y)  - Keratopathy (5Y): Epithelial ulcerations, Central subepithelial opacities.  - Pernicious anemia (7Y) | c.1193delC/c.1193delC p.P398fs/p.P398fs (PRR) | ([24](#_ENREF_24)) |  |
|  | 1 year | Female | Consanguineous (1^st^ degree cousins) | - Mucocutaneous candidiasis (1Y)  - Hypoparathyroidism (3Y)  - Adrenal insufficiency (1Y)  - Hypothyroidism (9Y)  - Keratopathy (6Y): Superior superﬁcial punctate staining  - Ovaritis (9Y) | c.1193delC/c.1193delC p.P398fs/p.P398fs (PRR) | ([24](#_ENREF_24)) |  |
|  | 6 years | Male | Consanguineous | - Candidiasis (6Y)  - Hypoparathyroidism (25Y)  - Addison’s disease (25Y)  - Gastritis (15Y)  - Malabsorption (25Y) | c.1193delC/c.1193delC p.P398fs/p.P398fs (PRR) | ([91](#_ENREF_91)) |  |
|  | N/A | N/A | Non-consanguineous | Presence of at least two of the following symptoms:  - Chronic mucocutaneous candidiasis  - Hypoparathyroidism  - Primary adrenocortical failure | c.1193delC/Unknown p.P398fs (PRR)/Unknown | ([45](#_ENREF_45)) |  |
|  | N/A | N/A | N/A | N/A | c.1242_1243insA/Unknown p.H415fs (L)/Unknown | ([23](#_ENREF_23)) |  |
|  | 13 years | Female | Non-consanguineous | - Adrenal insufficiency (14Y) - Hypoparathyroidism (13Y) - Alopecia - Autoimmune thyroid disease - Mucocutaneous candidiasis  - Primary gonadal insufficiency | c.1244_1245insC/c.1244_1245insC p.L417fs/p.L417fs (L) | ([11](#_ENREF_11), [85](#_ENREF_85)) |  |
|  | N/A | N/A | N/A | N/A | c.1244_1245insC/c.1244_1245insC p.L417fs/p.L417fs (L) | ([23](#_ENREF_23)) |  |
|  | N/A | N/A | N/A | N/A | c.1244_1245insC/c.1244_1245insC p.L417fs/p.L417fs (L) | ([23](#_ENREF_23)) |  |
|  | N/A | N/A | Non-consanguineous | Presence of at least two of the following symptoms:  - Chronic mucocutaneous candidiasis  - Hypoparathyroidism  - Primary adrenocortical failure | c.1249dupC/c.1249dupC p.L417fs/p.L417fs (L) | ([45](#_ENREF_45)) |  |
|  | N/A | N/A | N/A | N/A | c.1244_1245insC/Unknown  p.L417fs (L)/Unknown | ([23](#_ENREF_23)) |  |
|  | 13 years | Male | N/A | - Mucocutaneous candidiasis  - Hypoparathyroidism - Adrenal insufficiency - Alopecia - Chronic renal failure | c.1249dupC/c.967–979del13bp p.L417fs (L)/p.L323fs (PHD1) | ([11](#_ENREF_11)) |  |
|  | 14 years | Male | N/A | - Mucocutaneous candidiasis (22Y)  - Hypoparathyroidism (14Y)  - Alopecia (41Y) - Diabetes type 1 (23Y)  - Enamel hypoplasia - Keratopathy (25Y) - Nail pitting (25Y) - Vitiligo (41Y) | c.1249dupC/c.769C>T p.L417fs (L)/p.R257X (SAND) | ([11](#_ENREF_11), [112](#_ENREF_112)) |  |
|  | N/A | Male | N/A | - Mucocutaneous candidiasis  - Enamel hypoplasia | c.1249dupC/c.769C>T p.L417fs (L)/p.R257X (SAND) | ([112](#_ENREF_112)) |  |
|  | At birth | Female | Non-consanguineous | - Candidial diaper dermatitis (first 2 years)  - Oral thrush (between 2Y and 3Y)  - Abnormal movements of upper extremities (concerning for seizure) (5Y)  - 4 months of daily urticaria (between 2Y and 3Y)  - Dystrophic nails (4Y)  - Prolonged QTc interval  - 1 min of irregular eye movements (5Y) | c.1265delC/c.268T>C p.P422LfsX58 (PRR)/p.Y90H (HSR/CARD) | ([113](#_ENREF_113)) |  |
|  | N/A | Female | N/A | - Mucocutaneous candidiasis  - Hypoparathyroidism - Addison’s disease  - Chronic active hepatitis | c.1422insAC/WT p.C475fs (downstream of PHD2)/WT | ([58](#_ENREF_58)) |  |
|  | 14 years | Female | N/A | - Alopecia aerata  - Ectodermal dystrophy  - Goiter  - Nodular image in the right thyroid lobe  - Pitted nail dystrophy | c.1450G>A/WT p.V484M (downstream of PHD2)/WT | ([86](#_ENREF_86)) |  |
|  | 3.5 years | Male | N/A | - Chronic mucocutaneous candidiasis (5.5Y) - Addison’s disease (3.5Y) - Alopecia (4.5Y) | c.1497delT/c.1497delT  p.A500fs/p.A500fs (upstream of TAD) | ([55](#_ENREF_55)) |  |
|  | 1 year | Male | Consanguineous | - Chronic mucocutaneous candidiasis  - Hypoparathyroidism - Addison's disease (4Y) - Alopecia universalis (2Y) - Ectodermal dystrophy  - Hypergonadotropic hypogonadism - Pernicious anemia - Pneumonia - Preclinical autoimmune thyroiditis  - Urolithiasis, Nephrocalcinosis  - Vitiligo | c.1616C>T/c.1616C>T p.P539L/p.P539L (TAD) | ([15](#_ENREF_15), [114](#_ENREF_114)) |  |
|  | 3 years | Female | Consanguineous | - Oral chronic mucocutaneous candidiasis  - Hypoparathyroidism (3Y) - Addison's disease (9Y) - Alopecia areata - Autoimmune thyroiditis - Diabetes mellitus (14Y)  - Ectodermal dystrophy - Hypopituitarism (retarded growth and pubertal development) - Non suppurative otitis (7Y) | c.1616C>T/c.1616C>T p.P539L/p.P539L (TAD) | ([15](#_ENREF_15), [114](#_ENREF_114)) |  |
|  | N/A | Female | N/A | - Chronic mucocutaneous candidiasis - Hypoparathyroidism - Addison’s disease - Premature ovarian failure | c.1616C>T/c.1616C>T p.P539L/p.P539L (TAD) | ([115](#_ENREF_115)) |  |
|  | N/A | Female | N/A | - Hypoparathyroidism - Addison’s disease - Enamel hypoplasia | c.1616C>T/c.1616C>T p.P539L/p.P539L (TAD) | ([116](#_ENREF_116)) |  |
|  | 0.1 year | Female | Non-consanguineous | - Candidiasis - Hypoparathyroidism - Addison’s disease - Enamel hypoplasia - Nephrocalcinosis | c.1616C>T/c.1616C>T p.P539L/p.P539L (TAD) | ([37](#_ENREF_37)) |  |
|  | 3 years | Male | N/A | - Mucocutaneous candidiasis (3Y) - Hypoparathyroidism (4.5Y) - Alopecia  - Buphthalmos  - Enamel hypoplasia  - Nail dystrophy  - Pernicious anemia  - Short stature - Vitiligo | c.1616C>T/c.1616C>T p.P539L/p.P539L (TAD) | ([3](#_ENREF_3)) |  |
|  | N/A | N/A | N/A | N/A | c.1616C>T/c.1616C>T p.P539L/p.P539L (TAD) | ([18](#_ENREF_18)) |  |
|  | N/A | N/A | N/A | N/A | c.1616C>T/c.260T>C p.P539L (TAD)/p.L87P (HSR/CARD) | ([18](#_ENREF_18)) |  |
|  | N/A | N/A | N/A | N/A | c.1616C>T/c.931delT p.P539L (TAD)/p.C311fs (PHD1) | ([18](#_ENREF_18)) |  |
|  | N/A | Male | N/A | - Chronic mucocutaneous candidiasis - Autoimmune adrenal failure | c.1616C>T/c.967_979del13  p.P539L (TAD)/p.L323fs (PHD1) | ([106](#_ENREF_106)) |  |
|  | N/A | N/A | N/A | - Hypoparathyroidism - Addison’s disease - Insulin dependent diabetes mellitus | c.1616C>T/c.260T>C p.P539L (TAD)/p.L87P (HSR/CARD) | ([19](#_ENREF_19)) |  |
|  | N/A | N/A | N/A | N/A | c.1638A>T/Unknown p.X546C+59aa/Unknown | ([23](#_ENREF_23)) |  |
|  | N/A | N/A | N/A | N/A | c.1638A>T/Unknown p.X546C+59aa/Unknown | ([23](#_ENREF_23)) |  |
|  | N/A | N/A | N/A | N/A | c.1638A>T/Unknown p.X546C+59aa/Unknown | ([23](#_ENREF_23)) |  |
|  | N/A | N/A | N/A | N/A | c.1638A>T/Unknown p.X546C+59aa/Unknown | ([23](#_ENREF_23)) |  |
|  | N/A | Female | N/A | - Mucocutaneous candidiasis - Hypoparathyroidism  - Addison’s disease  - Alopecia  - Chronic active hepatitis  - Sjögren syndrome - Vitiligo | Unknown | ([74](#_ENREF_74)) |  |
|  | 36 years | Male | Non-consanguineous | - Chronic candidiasis (36Y) - Chronic hypoparathyroidism (36Y) - Alopecia - Autoimmune hepatitis - Dupuytren’s disease - Keratopathy - Malabsorption - Vitiligo | Unknown | ([36](#_ENREF_36)) |  |
|  | N/A | Female | Non-consanguineous | - Chronic candidiasis  - Chronic hypoparathyroidism (51Y) - Autoimmune gastritis - Cholelithiasis | Unknown | ([36](#_ENREF_36)) |  |
|  | 32 years | Female | Non-consanguineous | - Chronic mucocutaneous candidiasis  (candida esophagitis) - Addison’s disease - Antral gastritis - Ectodermal dystrophy - Primary hypogonadism | Unknown | ([15](#_ENREF_15)) |  |
|  | 9 years | Female | Non-consanguineous | - Oral chronic mucocutaneous candidiasis - Hypoparathyroidism - Addison’s disease - Ectodermal dystrophy - Pericarditis | Unknown | ([15](#_ENREF_15)) |  |
|  | 18 years | Female | Non-consanguineous | - Oral chronic mucocutaneous candidiasis - Hypoparathyroidism - Addison’s disease - Ectodermal dystrophy - Hyperpigmentation | Unknown | ([15](#_ENREF_15)) |  |
|  | 41 years | Male | Consanguineous | - Chronic mucocutaneous candidiasis  - Addison’s disease - Alopecia - Chronic urticaria - Chronic active hepatitis - Hypogonadotropic hypogonadism - Malabsorption - Vitiligo | Unknown | ([15](#_ENREF_15)) |  |
|  | 12 years | Male | N/A | - Oral chronic mucocutaneous candidiasis - Hypoparathyroidism - Amelogenesis imperfecta - Tooth eruption delay | Unknown | ([15](#_ENREF_15)) |  |
|  | 15 years | Female | N/A | - Oral chronic mucocutaneous candidiasis - Hypoparathyroidism - Epilepsy | Unknown | ([15](#_ENREF_15)) |  |
|  | 17 years | Female | N/A | - Oral chronic mucocutaneous candidiasis - Hypoparathyroidism - Addison’s disease - Ectodermal dystrophy in fingernails | Unknown | ([15](#_ENREF_15)) |  |
|  | 3 years | Male | N/A | - Chronic mucocutaneous candidiasis - Hypoparathyroidism - Addison’s disease | Unknown | ([15](#_ENREF_15)) |  |
|  | 7 years | Female | Non-consanguineous | - Mucocutaneous candidiasis (7Y) - Adrenal insufficiency (22Y) - Alopecia - Dental enamel hypoplasia - Malabsorption - Vitiligo | Unknown | ([11](#_ENREF_11), [85](#_ENREF_85)) |  |
|  | 3 years | Female | N/A | - Hypoparathyroidism | Unknown | ([11](#_ENREF_11)) |  |
|  | 5 years | Male | N/A | - Mucocutaneous candidiasis  - Hypoparathyroidism - Adrenal insufficiency - Alopecia - Dental enamel hypoplasia | Unknown | ([11](#_ENREF_11)) |  |
|  | 8 years | Female |  | - Hypoparathyroidism  - Autoimmune thyroid disease - Dental enamel hypoplasia | Unknown | ([11](#_ENREF_11)) |  |
|  | 0.6 year | N/A | N/A | - Chronic mucocutaneous candidiasis (0.6Y) - Hypoparathyroidism (8.1Y) - Adrenal insufficiency (9.4Y) - Hypothyroidism (10Y) | Unknown | ([6](#_ENREF_6)) |  |
|  | 7 years | N/A | N/A | - Chronic mucocutaneous candidiasis (7Y) - Hypoparathyroidism (7Y) - Adrenal insufficiency (7Y) - Hypothyroidism (9.2) - Pernicious anemia (7Y)  - Vitiligo (14.3Y) | Unknown | ([6](#_ENREF_6)) |  |
|  | 3 years | N/A | N/A | - Chronic mucocutaneous candidiasis (3Y) - Hypoparathyroidism (5.5Y) - Adrenal insufficiency (8.5Y) - Alopecia (8Y) - Pernicious anemia (12Y) | Unknown | ([6](#_ENREF_6)) |  |
|  | N/A | Male | N/A | - Hypoparathyroidism - Addison disease - Alopecia | Unknown | ([58](#_ENREF_58)) |  |
|  | N/A | Female | N/A | - Hypoparathyroidism - Addison disease - Alopecia | Unknown | ([58](#_ENREF_58)) |  |
|  | N/A | N/A | Non-consanguineous | Presence of at least two of the following symptoms:  - Chronic mucocutaneous candidiasis  - Hypoparathyroidism  - Primary adrenocortical failure | Unknown | ([45](#_ENREF_45)) |  |
|  | 1.5-5 years | Male | N/A | - Mucocutaneous candidiasis  - Hypoparathyroidism  - Addison’s disease  - Chronic malabsorption - Classic pituitary growth hormone deficiency  - Hypothyroidism | Unknown | ([51](#_ENREF_51)) |  |

*Abbreviations*: WT, wild type; N/A, not available; dup, Duplication; del, Deletion; Ins, Insertion; IVS, intervening sequence; bp, base pair; HSR, Homogeneously staining region; CARD, Caspase activation and recruitment domain; L, Linker region; SAND, Sp100, AIRE-1, NucP41/75, DEAF-1 domain; PHD, Plant homeodomain; PRR, Proline-rich region; TAD, Transactivation domain.

**References**

1. Podkrajsek, K.T., T. Milenkovic, R.J. Odink, H.L. Claasen-van der Grinten, N. Bratanic, T. Hovnik, T. Battelino. 2008. Detection of a complete autoimmune regulator gene deletion and two additional novel mutations in a cohort of patients with atypical phenotypic variants of autoimmune polyglandular syndrome type 1. *Eur. J. Endocrinol.* 159(5):633-9. 10.1530/EJE-08-0328.

2. Faiyaz-Ul-Haque, M., B. Bin-Abbas, A. Al-Abdullatif, H. Abdullah Abalkhail, M. Toulimat, S. Al-Gazlan, A.M. Almutawa, A. Al-Sagheir, I. Peltekova, F. Al-Dayel, S.H. Zaidi. 2009. Novel and recurrent mutations in the AIRE gene of autoimmune polyendocrinopathy syndrome type 1 (APS1) patients. *Clin. Genet.* 76(5):431-40. 10.1111/j.1399-0004.2009.01278.x.

3. Zaidi, G., V. Bhatia, S.K. Sahoo, A.N. Sarangi, N. Bharti, L. Zhang, L. Yu, D. Eriksson, S. Bensing, O. Kampe, N. Bharani, S.K. Yachha, A. Bhansali, A. Sachan, V. Jain, N. Shah, R. Aggarwal, A. Aggarwal, M. Srinivasan, S. Agarwal, E. Bhatia. 2017. Autoimmune polyendocrine syndrome type 1 in an Indian cohort: a longitudinal study. *Endocr Connect*. 6(5):289-96. 10.1530/EC-17-0022.

4. Stolarski, B., E. Pronicka, L. Korniszewski, A. Pollak, G. Kostrzewa, E. Rowinska, P. Wlodarski, A. Skorka, M. Gremida, P. Krajewski, R. Ploski. 2006. Molecular background of polyendocrinopathy-candidiasis-ectodermal dystrophy syndrome in a Polish population: novel AIRE mutations and an estimate of disease prevalence. *Clin. Genet.* 70(4):348-54. 10.1111/j.1399-0004.2006.00690.x.

5. Orlova, E.M., L.S. Sozaeva, M.A. Kareva, B.E. Oftedal, A.S.B. Wolff, L. Breivik, E.Y. Zakharova, O.N. Ivanova, O. Kampe, Dedov, II, P.M. Knappskog, V.A. Peterkova, E.S. Husebye. 2017. Expanding the Phenotypic and Genotypic Landscape of Autoimmune Polyendocrine Syndrome Type 1. *J. Clin. Endocrinol. Metab.* 102(9):3546-56. 10.1210/jc.2017-00139.

6. Orlova, E.M., A.M. Bukina, E.S. Kuznetsova, M.A. Kareva, E.U. Zakharova, V.A. Peterkova, Dedov, II. 2010. Autoimmune polyglandular syndrome type 1 in Russian patients: clinical variants and autoimmune regulator mutations. *Horm. Res. Paediatr.* 73(6):449-57. 10.1159/000313585.

7. Bratanic, N., K. Kisand, M. Avbelj Stefanija, T. Battelino, K. Trebusak Podkrajsek. 2015. Clinical, Genetic and Immunological Characteristics of Paediatric Autoimmune Polyglandular Syndrome Type 1 Patients in Slovenia. *Zdr Varst*. 54(2):112-8. 10.1515/sjph-2015-0017.

8. Podkrajsek, K.T., N. Bratanic, C. Krzisnik, T. Battelino. 2005. Autoimmune regulator-1 messenger ribonucleic acid analysis in a novel intronic mutation and two additional novel AIRE gene mutations in a cohort of autoimmune polyendocrinopathy-candidiasis-ectodermal dystrophy patients. *J. Clin. Endocrinol. Metab.* 90(8):4930-5. 10.1210/jc.2005-0418.

9. Toth, B., A.S. Wolff, Z. Halasz, A. Tar, P. Szuts, I. Ilyes, M. Erdos, G. Szegedi, E.S. Husebye, M. Zeher, L. Marodi. 2010. Novel sequence variation of AIRE and detection of interferon-omega antibodies in early infancy. *Clin. Endocrinol. (Oxf.)*. 72(5):641-7. 10.1111/j.1365-2265.2009.03740.x.

10. Cihakova, D., K. Trebusak, M. Heino, V. Fadeyev, A. Tiulpakov, T. Battelino, A. Tar, Z. Halasz, P. Blumel, S. Tawfik, K. Krohn, J. Lebl, P. Peterson. 2001. Novel AIRE mutations and P450 cytochrome autoantibodies in Central and Eastern European patients with APECED. *Hum. Mutat.* 18(3):225-32. 10.1002/humu.1178.

11. Wolff, A.S., M.M. Erichsen, A. Meager, N.F. Magitta, A.G. Myhre, J. Bollerslev, K.J. Fougner, K. Lima, P.M. Knappskog, E.S. Husebye. 2007. Autoimmune polyendocrine syndrome type 1 in Norway: phenotypic variation, autoantibodies, and novel mutations in the autoimmune regulator gene. *J. Clin. Endocrinol. Metab.* 92(2):595-603. 10.1210/jc.2006-1873.

12. Al Jabri, A., Al Essa, A. and Al Shaib, S. . 2020. Unusual Presentation of Precocious Puberty and Alopecia Universalis in Saudi Patients with Autoimmune Polyglandular Syndrome Type 1(APS1) without Any Other Manifestation of the Disease: Case Report and a Brief Review of the Literature. *Case Rep. Clin. Med.* 9(7):208-16. <https://doi.org/10.4236/crcm.2020.97029>.

13. Cranston, T., H. Boon, M.K. Olesen, F.J. Ryan, D. Shears, R. London, H. Rostom, T. Elajnaf, R.V. Thakker, F.M. Hannan. 2022. Spectrum of germline AIRE mutations causing APS-1 and familial hypoparathyroidism. *Eur. J. Endocrinol.* 187(1):111-22. 10.1530/EJE-21-0730.

14. Celmeli, F., A. Kocabas, I.A. Isik, M. Parlak, K. Kisand, S. Ceylaner, D. Turkkahraman. 2017. Unexplained cyanosis caused by hepatopulmonary syndrome in a girl with APECED syndrome. *J. Pediatr. Endocrinol. Metab.* 30(3):365-9. 10.1515/jpem-2016-0276.

15. Fierabracci, A., M. Pellegrino, F. Frasca, S.S. Kilic, C. Betterle. 2018. APECED in Turkey: A case report and insights on genetic and phenotypic variability. *Clin. Immunol.* 194:60-6. 10.1016/j.clim.2018.06.012.

16. Kollios, K., A. Tsolaki, C. Antachopoulos, I. Moix, M.A. Morris, M. Papadopoulou, E. Roilides. 2011. Autoimmune polyendocrinopathy-candidiasis-ectodermal dystrophy syndrome (APECED) due to AIRET16M mutation in a consanguineous Greek girl. *J. Pediatr. Endocrinol. Metab.* 24(7-8):599-601. 10.1515/jpem.2011.012.

17. Capalbo, D., C. Mazza, R. Giordano, N. Improda, E. Arvat, S. Cervato, L. Morlin, C. Pignata, C. Betterle, M. Salerno. 2012. Molecular background and genotype-phenotype correlation in autoimmune-polyendocrinopathy-candidiasis-ectodermal-distrophy patients from Campania and in their relatives. *J. Endocrinol. Invest.* 35(2):169-73. 10.3275/7677.

18. Garelli, S., M. Dalla Costa, C. Sabbadin, S. Barollo, B. Rubin, R. Scarpa, S. Masiero, A. Fierabracci, C. Bizzarri, A. Crino, M. Cappa, M. Valenzise, A. Meloni, A.M. De Bellis, C. Giordano, F. Presotto, R. Perniola, D. Capalbo, M.C. Salerno, A. Stigliano, G. Radetti, V. Camozzi, N.A. Greggio, F. Bogazzi, I. Chiodini, U. Pagotto, S.K. Black, S. Chen, B. Rees Smith, J. Furmaniak, G. Weber, F. Pigliaru, L. De Sanctis, C. Scaroni, C. Betterle. 2021. Autoimmune polyendocrine syndrome type 1: an Italian survey on 158 patients. *J. Endocrinol. Invest.* 44(11):2493-510. 10.1007/s40618-021-01585-6.

19. Mazza, C., F. Buzi, F. Ortolani, A. Vitali, L.D. Notarangelo, G. Weber, R. Bacchetta, A. Soresina, V. Lougaris, N.A. Greggio, A. Taddio, S. Pasic, M. de Vroede, M. Pac, S.S. Kilic, S. Ozden, R. Rusconi, S. Martino, D. Capalbo, M. Salerno, C. Pignata, G. Radetti, G. Maggiore, A. Plebani, L.D. Notarangelo, R. Badolato. 2011. Clinical heterogeneity and diagnostic delay of autoimmune polyendocrinopathy-candidiasis-ectodermal dystrophy syndrome. *Clin. Immunol.* 139(1):6-11. 10.1016/j.clim.2010.12.021.

20. Valenzise, M., M. Wasniewska, S. Mirabelli, F. De Luca, S. Cervato, C. Betterle. 2012. Identification of two novel mutations in the first Sicilian APECED patient with no R203X mutation in AIRE gene and review of Italian APECED genotypes. *Gene*. 499(2):343-6. 10.1016/j.gene.2012.03.032.

21. Kamat, D., R. Mahajan, D. Chatterjee, J. Yadav, R. Kumar, D. Dayal, D. De, S. Handa. 2022. Clinical and Genetic Characteristics of Ectodermal Dysplasia in Four Indian Children. *Indian J. Dermatol.* 67(1):54-7. 10.4103/ijd.ijd_406_21.

22. Improda, N., D. Capalbo, E. Cirillo, M. Cerbone, A. Esposito, C. Pignata, M. Salerno. 2014. Cutaneous vasculitis in patients with autoimmune polyendocrine syndrome type 1: report of a case and brief review of the literature. *BMC Pediatr.* 14:272. 10.1186/1471-2431-14-272.

23. Halonen, M., P. Eskelin, A.G. Myhre, J. Perheentupa, E.S. Husebye, O. Kampe, F. Rorsman, L. Peltonen, I. Ulmanen, J. Partanen. 2002. AIRE mutations and human leukocyte antigen genotypes as determinants of the autoimmune polyendocrinopathy-candidiasis-ectodermal dystrophy phenotype. *J. Clin. Endocrinol. Metab.* 87(6):2568-74. 10.1210/jcem.87.6.8564.

24. Couturier, A., P. Saugier-Veber, J.C. Carel, J. Bertherat, A.P. Brezin. 2015. Keratopathy in Autoimmune Polyendocrinopathy Syndrome Type 1. *Cornea*. 34(9):1086-91. 10.1097/ICO.0000000000000513.

25. Oftedal, B.E., A.S. Wolff, E. Bratland, O. Kampe, J. Perheentupa, A.G. Myhre, A. Meager, R. Purushothaman, S. Ten, E.S. Husebye. 2008. Radioimmunoassay for autoantibodies against interferon omega; its use in the diagnosis of autoimmune polyendocrine syndrome type I. *Clin. Immunol.* 129(1):163-9. 10.1016/j.clim.2008.07.002.

26. Heino, M., H.S. Scott, Q. Chen, P. Peterson, U. Maebpaa, M.P. Papasavvas, L. Mittaz, C. Barras, C. Rossier, G.P. Chrousos, C.A. Stratakis, K. Nagamine, J. Kudoh, N. Shimizu, N. Maclaren, S.E. Antonarakis, K. Krohn. 1999. Mutation analyses of North American APS-1 patients. *Hum. Mutat.* 13(1):69-74. 10.1002/(SICI)1098-1004(1999)13:1<69::AID-HUMU8>3.0.CO;2-6.

27. Pearce, S.H., T. Cheetham, H. Imrie, B. Vaidya, N.D. Barnes, R.W. Bilous, D. Carr, K. Meeran, N.J. Shaw, C.S. Smith, A.D. Toft, G. Williams, P. Kendall-Taylor. 1998. A common and recurrent 13-bp deletion in the autoimmune regulator gene in British kindreds with autoimmune polyendocrinopathy type 1. *Am. J. Hum. Genet.* 63(6):1675-84. 10.1086/302145.

28. Ishii, T., Y. Suzuki, N. Ando, N. Matsuo, T. Ogata. 2000. Novel mutations of the autoimmune regulator gene in two siblings with autoimmune polyendocrinopathy-candidiasis-ectodermal dystrophy. *J. Clin. Endocrinol. Metab.* 85(8):2922-6. 10.1210/jcem.85.8.6726.

29. Capalbo, D., A. Elefante, M.I. Spagnuolo, C. Mazza, C. Betterle, C. Pignata, M. Salerno. 2008. Posterior reversible encephalopathy syndrome in a child during an accelerated phase of a severe APECED phenotype due to an uncommon mutation of AIRE. *Clin. Endocrinol. (Oxf.)*. 69(3):511-3. 10.1111/j.1365-2265.2008.03206.x.

30. Gutierrez, M.J., J. Gilson, J. Zacharias, F. Ishmael, C.A. Bingham. 2017. Childhood Polyarthritis As Early Manifestation of Autoimmune Polyendocrinopathy with Candidiasis and Ectodermal Dystrophy Syndrome. *Front. Immunol.* 8:377. 10.3389/fimmu.2017.00377.

31. Barg, E., M. Skarzynska, A. Pollak, R. Slezak, E. Glab, E. Petriczko, A. Jozwa, M.M. Sasiadek. 2014. Uncommon constellation of multiglandular deficiency with 2 mutations in AIRE gene in an 18-year-old girl - 12 years of observation. *Endokrynol. Pol.* 65(6):514-8. 10.5603/EP.2014.0070.

32. Arunachalam, A.K., M. Maddali, F.N. Aboobacker, A. Korula, B. George, V. Mathews, E.S. Edison. 2021. Primary Immunodeficiencies in India: Molecular Diagnosis and the Role of Next-Generation Sequencing. *J. Clin. Immunol.* 41(2):393-413. 10.1007/s10875-020-00923-2.

33. Zheng, W.B., L.J. Li, D.C. Zhao, O. Wang, Y. Jiang, W.B. Xia, M. Li. 2020. A novel variant in AIRE causing a rare, non‑classical autoimmune polyendocrine syndrome type 1. *Mol Med Rep*. 22(2):1285-94. 10.3892/mmr.2020.11227.

34. Zhu, W., Z. Hu, X. Liao, X. Chen, W. Huang, Y. Zhong, Z. Zeng. 2017. A new mutation site in the AIRE gene causes autoimmune polyendocrine syndrome type 1. *Immunogenetics*. 69(10):643-51. 10.1007/s00251-017-0995-5.

35. Soyak Aytekin, E., O. Serin, D. Cagdas, C. Tan, T. Aksu, Y. Unsal, S. Yeni, D. Orhan, Z.A. Ozon, I. Tezcan. 2021. A Patient With AIRE Mutation Who Presented With Severe Diarrhea and Lung Abscess. *Pediatr. Infect. Dis. J.* 40(1):66-9. 10.1097/INF.0000000000002887.

36. Cervato, S., B. Mariniello, F. Lazzarotto, L. Morlin, R. Zanchetta, G. Radetti, F. De Luca, M. Valenzise, R. Giordano, D. Rizzo, C. Giordano, C. Betterle. 2009. Evaluation of the autoimmune regulator (AIRE) gene mutations in a cohort of Italian patients with autoimmune-polyendocrinopathy-candidiasis-ectodermal-dystrophy (APECED) and in their relatives. *Clin. Endocrinol. (Oxf.)*. 70(3):421-8. 10.1111/j.1365-2265.2008.03318.x.

37. Meloni, A., R. Perniola, V. Faa, E. Corvaglia, A. Cao, M.C. Rosatelli. 2002. Delineation of the molecular defects in the AIRE gene in autoimmune polyendocrinopathy-candidiasis-ectodermal dystrophy patients from Southern Italy. *J. Clin. Endocrinol. Metab.* 87(2):841-6. 10.1210/jcem.87.2.8209.

38. Betterle, C., L. Ghizzoni, A. Cassio, F. Baronio, S. Cervato, S. Garelli, E. Barbi, G. Tonini. 2012. Autoimmune-polyendocrinopathy-candidiasis-ectodermal-dystrophy in Calabria: clinical, immunological and genetic patterns. *J. Endocrinol. Invest.* 35(10):877-81. 10.3275/8109.

39. Cervato, S., L. Morlin, M.P. Albergoni, S. Masiero, N. Greggio, C. Meossi, S. Chen, M. del Pilar Larosa, J. Furmaniak, B. Rees Smith, M. Alimohammadi, O. Kampe, M. Valenzise, C. Betterle. 2010. AIRE gene mutations and autoantibodies to interferon omega in patients with chronic hypoparathyroidism without APECED. *Clin. Endocrinol. (Oxf.)*. 73(5):630-6. 10.1111/j.1365-2265.2010.03862.x.

40. Scarpa, R., R. Alaggio, L. Norberto, J. Furmaniak, S. Chen, B.R. Smith, S. Masiero, L. Morlin, M. Plebani, F. De Luca, M.C. Salerno, R. Giordano, G. Radetti, L. Ghizzoni, G. Tonini, F. Farinati, C. Betterle. 2013. Tryptophan hydroxylase autoantibodies as markers of a distinct autoimmune gastrointestinal component of autoimmune polyendocrine syndrome type 1. *J. Clin. Endocrinol. Metab.* 98(2):704-12. 10.1210/jc.2012-2734.

41. Setoodeh, A., S. Panjeh-Shahi, F. Bahmani, F. Vand-Rajabpour, N. Jalilian, F. Sayarifard, F. Abbasi, A. Sayarifard, P. Rostami, N. Parvaneh, H. Akhavan-Niaki, M. Ahmadifard, M. Tabrizi. 2022. Molecular and clinical characterization of autoimmune polyendocrinopathy-candidiasis-ectodermal dystrophy syndrome (APECED) in Iranian non-Jewish patients: report of two novel AIRE gene pathogenic variants. *Orphanet J. Rare Dis.* 17(1):10. 10.1186/s13023-021-02170-z.

42. Meloni, A., E. Fiorillo, D. Corda, R. Perniola, A. Cao, M.C. Rosatelli. 2005. Two novel mutations of the AIRE protein affecting its homodimerization properties. *Hum. Mutat.* 25(3):319. 10.1002/humu.9309.

43. Zaidi, G., R.P. Sahu, L. Zhang, G. George, N. Bhavani, N. Shah, V. Bhatia, A. Bhansali, G. Jevalikar, R.V. Jayakumar, G.S. Eisenbarth, E. Bhatia. 2009. Two novel AIRE mutations in autoimmune polyendocrinopathy-candidiasis-ectodermal dystrophy (APECED) among Indians. *Clin. Genet.* 76(5):441-8. 10.1111/j.1399-0004.2009.01280.x.

44. Chen, C.B., N. Mistry Ambani, A. Zeft, R. Garcia-Naviero, V. Hupertz, K. Hashimoto, K. Radhakrishnan. 2021. Rare genetic mutation triggering acute liver failure in a toddler requiring a liver transplant. *Pediatr. Transplant.* 25(6):e14048. 10.1111/petr.14048.

45. Bjorses, P., M. Halonen, J.J. Palvimo, M. Kolmer, J. Aaltonen, P. Ellonen, J. Perheentupa, I. Ulmanen, L. Peltonen. 2000. Mutations in the AIRE gene: effects on subcellular location and transactivation function of the autoimmune polyendocrinopathy-candidiasis-ectodermal dystrophy protein. *Am. J. Hum. Genet.* 66(2):378-92. 10.1086/302765.

46. Eyal, O., A. Oren, H. Juppner, R. Somech, A. De Bellis, M. Mannstadt, A. Szalat, M. Bleiberg, Y. Weisman, N. Weintrob. 2014. Hypoparathyroidism and central diabetes insipidus: in search of the link. *Eur. J. Pediatr.* 173(12):1731-4. 10.1007/s00431-014-2448-6.

47. Carpino, A., R. Buganza, P. Matarazzo, G. Tuli, M. Pinon, P.L. Calvo, D. Montin, F. Licciardi, L. De Sanctis. 2021. Autoimmune Polyendocrinopathy-Candidiasis-Ectodermal Dystrophy in Two Siblings: Same Mutations but Very Different Phenotypes. *Genes (Basel)*. 12(2). 10.3390/genes12020169.

48. Akil, H., A.D. Bulus, N. Andiran, P.S. Veber, S. Keskin. 2016. A case of autoimmune polyendocrine syndrome type 1 with ocular findings and unique AIRE gene defect. *Journal of Clinical Ophthalmology and Research*. 4(1):37-9. 10.4103/2320-3897.174414.

49. Alshahrany, A.S.A., M. S.; Al-Ghamdi, A. H. 2020. Autoimmune poly glandular sy ndrome type 1 with end stage renal disease;rare presentationwithnovel allelic variant: a case report. *International Journal of Current Research*. 12(08 ):13031-3. <https://doi.org/10.24941/ijcr.39485.08.2020>.

50. Weiler, F.G., P. Peterson, B.T. Costa-Carvalho, M. de Barros Dorna, J.E. Correia-Deur, S.L. Sader, D. Espindola-Antunes, G. Guerra-Junior, M.R. Dias-da-Silva, M. Lazaretti-Castro. 2018. The heterogeneity of autoimmune polyendocrine syndrome type 1: Clinical features, new mutations and cytokine autoantibodies in a Brazilian cohort from tertiary care centers. *Clin. Immunol.* 197:231-8. 10.1016/j.clim.2018.09.012.

51. Winer, K.K., K.A. Fulton, P.S. Albert, G.B. Cutler, Jr. 2014. Effects of pump versus twice-daily injection delivery of synthetic parathyroid hormone 1-34 in children with severe congenital hypoparathyroidism. *J. Pediatr.* 165(3):556-63 e1. 10.1016/j.jpeds.2014.04.060.

52. Pellegrino, M., E. Bellacchio, R. Dhamo, F. Frasca, C. Betterle, A. Fierabracci. 2018. A Novel Homozygous Mutation of the AIRE Gene in an APECED Patient From Pakistan: Case Report and Review of the Literature. *Front. Immunol.* 9:1835. 10.3389/fimmu.2018.01835.

53. Seifi-Alan, M., R. Shamsi, A. Setoodeh, F. Sayarifard, P. Aghasi, F. Kompani, S. Ghafouri-Fard, F. Abbasi. 2016. Autoimmune polyendocrinopathy-candidiasis-ectodermal dystrophy: report of three cases from Iran. *J. Pediatr. Endocrinol. Metab.* 29(8):979-83. 10.1515/jpem-2016-0017.

54. Rosatelli, M.C., A. Meloni, A. Meloni, M. Devoto, A. Cao, H.S. Scott, P. Peterson, M. Heino, K.J. Krohn, K. Nagamine, J. Kudoh, N. Shimizu, S.E. Antonarakis. 1998. A common mutation in Sardinian autoimmune polyendocrinopathy-candidiasis-ectodermal dystrophy patients. *Hum. Genet.* 103(4):428-34. 10.1007/s004390050846.

55. Wolff, A.S., A.K. Sarkadi, L. Marodi, J. Karner, E. Orlova, B.E. Oftedal, K. Kisand, E. Olah, A. Meloni, A.G. Myhre, E.S. Husebye, R. Motaghedi, J. Perheentupa, P. Peterson, N. Willcox, A. Meager. 2013. Anti-cytokine autoantibodies preceding onset of autoimmune polyendocrine syndrome type I features in early childhood. *J. Clin. Immunol.* 33(8):1341-8. 10.1007/s10875-013-9938-6.

56. Zhang, J., H. Liu, Z. Liu, Y. Liao, L. Guo, H. Wang, L. He, X. Zhang, Q. Xing. 2013. A functional alternative splicing mutation in AIRE gene causes autoimmune polyendocrine syndrome type 1. *PLoS One*. 8(1):e53981. 10.1371/journal.pone.0053981.

57. Posovszky, C., G. Lahr, J. von Schnurbein, S. Buderus, A. Findeisen, C. Schroder, C. Schutz, A. Schulz, K.M. Debatin, M. Wabitsch, T.F. Barth. 2012. Loss of enteroendocrine cells in autoimmune-polyendocrine-candidiasis-ectodermal-dystrophy (APECED) syndrome with gastrointestinal dysfunction. *J. Clin. Endocrinol. Metab.* 97(2):E292-300. 10.1210/jc.2011-2044.

58. Wang, C.Y., A. Davoodi-Semiromi, W. Huang, E. Connor, J.D. Shi, J.X. She. 1998. Characterization of mutations in patients with autoimmune polyglandular syndrome type 1 (APS1). *Hum. Genet.* 103(6):681-5. 10.1007/s004390050891.

59. Dominguez, M., E. Crushell, T. Ilmarinen, E. McGovern, S. Collins, B. Chang, P. Fleming, A.D. Irvine, D. Brosnahan, I. Ulmanen, N. Murphy, C. Costigan. 2006. Autoimmune polyendocrinopathy-candidiasis-ectodermal dystrophy (APECED) in the Irish population. *J. Pediatr. Endocrinol. Metab.* 19(11):1343-52. 10.1515/jpem.2006.19.11.1343.

60. Jin, P., Q. Zhang, C.S. Dong, S.L. Zhao, Z.H. Mo. 2014. A novel mutation in autoimmune regulator gene causes autoimmune polyendocrinopathy-candidiasis-ectodermal dystrophy. *J. Endocrinol. Invest.* 37(10):941-8. 10.1007/s40618-014-0120-7.

61. Ferre, E.M., S.R. Rose, S.D. Rosenzweig, P.D. Burbelo, K.R. Romito, J.E. Niemela, L.B. Rosen, T.J. Break, W. Gu, S. Hunsberger, S.K. Browne, A.P. Hsu, S. Rampertaap, M. Swamydas, A.L. Collar, H.H. Kong, C.R. Lee, D. Chascsa, T. Simcox, A. Pham, A. Bondici, M. Natarajan, J. Monsale, D.E. Kleiner, M. Quezado, I. Alevizos, N.M. Moutsopoulos, L. Yockey, C. Frein, A. Soldatos, K.R. Calvo, J. Adjemian, M.N. Similuk, D.M. Lang, K.D. Stone, G. Uzel, J.B. Kopp, R.J. Bishop, S.M. Holland, K.N. Olivier, T.A. Fleisher, T. Heller, K.K. Winer, M.S. Lionakis. 2016. Redefined clinical features and diagnostic criteria in autoimmune polyendocrinopathy-candidiasis-ectodermal dystrophy. *JCI. Insight*. 1(13). 10.1172/jci.insight.88782.

62. Suspitsin, E.N., M.N. Guseva, M.M. Kostik, A.P. Sokolenko, N.V. Skripchenko, A.S. Levina, O.V. Goleva, M.F. Dubko, A.V. Tumakova, M.A. Makhova, L.V. Lyazina, I.V. Bizin, N.E. Sokolova, T.V. Gabrusskaya, L.V. Ditkovskaya, O.P. Kozlova, S.S. Vahliarskaya, I.V. Kondratenko, E.N. Imyanitov. 2020. Next generation sequencing analysis of consecutive Russian patients with clinical suspicion of inborn errors of immunity. *Clin. Genet.* 98(3):231-9. 10.1111/cge.13789.

63. Giordano, C., R. Modica, M.L. Allotta, V. Guarnotta, S. Cervato, S. Masiero, R. Giordano, S. Garelli, C. Betterle. 2012. Autoimmune polyendocrinopathy-candidiasis-ectodermal-dystrophy (APECED) in Sicily: confirmation that R203X is the peculiar AIRE gene mutation. *J. Endocrinol. Invest.* 35(4):384-8. 10.3275/7965.

64. De Luca, F., M. Valenzise, R. Alaggio, T. Arrigo, G. Crisafulli, G. Salzano, S. Cervato, B. Mariniello, F. Lazzarotto, C. Betterle. 2008. Sicilian family with autoimmune polyendocrinopathy-candidiasis-ectodermal dystrophy (APECED) and lethal lung disease in one of the affected brothers. *Eur. J. Pediatr.* 167(11):1283-8. 10.1007/s00431-008-0668-3.

65. Scott, H.S., M. Heino, P. Peterson, L. Mittaz, M.D. Lalioti, C. Betterle, A. Cohen, M. Seri, M. Lerone, G. Romeo, P. Collin, M. Salo, R. Metcalfe, A. Weetman, M.P. Papasavvas, C. Rossier, K. Nagamine, J. Kudoh, N. Shimizu, K.J. Krohn, S.E. Antonarakis. 1998. Common mutations in autoimmune polyendocrinopathy-candidiasis-ectodermal dystrophy patients of different origins. *Mol. Endocrinol.* 12(8):1112-9. 10.1210/mend.12.8.0143.

66. Sun, Y.X., Y.F. He, X.L. Li. 2016. [Clinical analysis and autoimmune regulator gene mutation of autoimmune polyendocrinopathy syndrome type I in a family: a report of one case]. *Zhongguo Dang Dai Er Ke Za Zhi*. 18(2):147-51. 10.7499/j.issn.1008-8830.2016.02.010.

67. Cetani, F., G. Barbesino, S. Borsari, E. Pardi, L. Cianferotti, A. Pinchera, C. Marcocci. 2001. A novel mutation of the autoimmune regulator gene in an Italian kindred with autoimmune polyendocrinopathy-candidiasis-ectodermal dystrophy, acting in a dominant fashion and strongly cosegregating with hypothyroid autoimmune thyroiditis. *J. Clin. Endocrinol. Metab.* 86(10):4747-52. 10.1210/jcem.86.10.7884.

68. Kisand, K., M. Link, A.S. Wolff, A. Meager, L. Tserel, T. Org, A. Murumagi, R. Uibo, N. Willcox, K. Trebusak Podkrajsek, T. Battelino, A. Lobell, O. Kampe, K. Lima, A. Meloni, B. Ergun-Longmire, N.K. Maclaren, J. Perheentupa, K.J. Krohn, H.S. Scott, E.S. Husebye, P. Peterson. 2008. Interferon autoantibodies associated with AIRE deficiency decrease the expression of IFN-stimulated genes. *Blood*. 112(7):2657-66. 10.1182/blood-2008-03-144634.

69. Nagamine, K., P. Peterson, H.S. Scott, J. Kudoh, S. Minoshima, M. Heino, K.J. Krohn, M.D. Lalioti, P.E. Mullis, S.E. Antonarakis, K. Kawasaki, S. Asakawa, F. Ito, N. Shimizu. 1997. Positional cloning of the APECED gene. *Nat. Genet.* 17(4):393-8. 10.1038/ng1297-393.

70. Fuchtenbusch, M., A. Vogel, P. Achenbach, M. Gummer, A.G. Ziegler, E. Albert, E. Standl, M.P. Manns. 2003. Lupus-like panniculitis in a patient with autoimmune polyendocrinopathy-candidiasis-ectodermal dystrophy (APECED). *Exp. Clin. Endocrinol. Diabetes*. 111(5):288-93. 10.1055/s-2003-41287.

71. Korniszewski, L., M. Kurzyna, B. Stolarski, A. Torbicki, A. Smerdel, R. Ploski. 2003. Fatal primary pulmonary hypertension in a 30-yr-old female with APECED syndrome. *Eur. Respir. J.* 22(4):709-11. 10.1183/09031936.03.00018203.

72. Sorkina, E., E. Frolova, D. Rusinova, S. Polyakova, E. Roslavtseva, E. Vasilyev, V. Petrov, A. Tiulpakov. 2016. Progressive Generalized Lipodystrophy as a Manifestation of Autoimmune Polyglandular Syndrome Type 1. *J. Clin. Endocrinol. Metab.* 101(4):1344-7. 10.1210/jc.2015-3722.

73. Sarkadi, A.K., S. Tasko, G. Csorba, B. Toth, M. Erdos, L. Marodi. 2014. Autoantibodies to IL-17A may be correlated with the severity of mucocutaneous candidiasis in APECED patients. *J. Clin. Immunol.* 34(2):181-93. 10.1007/s10875-014-9987-5.

74. Sediva, A., D. Cihakova, J. Lebl. 2002. Immunological findings in patients with autoimmune polyendocrinopathy-candidiasis-ectodermal dystrophy (APECED) and their family members: are heterozygotes subclinically affected? *J. Pediatr. Endocrinol. Metab.* 15(9):1491-6. 10.1515/jpem.2002.15.9.1491.

75. Güçlü, M., Cangül, H. ve Ersoy, C. . 2015. Strong similarities in Turkish and European patients diagnosed with APECED syndrome. *Turkish Journal of Endocrinology and Metabolism*. 19(3):89-92. <https://dx.doi.org/10.4274/tjem.2987>.

76. Skrabic, V., I. Skrabic, R. Skrabic, B. Roje, M. Simunovic. 2022. Clinical Characteristics in the Longitudinal Follow-Up of APECED Syndrome in Southern Croatia-Case Series. *Genes (Basel)*. 13(4). 10.3390/genes13040558.

77. Chinello, M., M. Mauro, G. Cantalupo, R. Balter, M. De Bortoli, V. Vitale, A. Zaccaron, E. Bonetti, R. Gaudino, E. Fiorini, S. Cesaro. 2019. Pure Red Cell Aplasia (PRCA) and Cerebellar Hypoplasia as Atypical Features of Polyglandular Autoimmune Syndrome Type I (APS-1): Two Sisters With the Same AIRE Mutation but Different Phenotypes. *Front. Pediatr.* 7:51. 10.3389/fped.2019.00051.

78. Jamee, M., S.A. Mahdaviani, D. Mansouri, G. Azizi, N. Joneidi, H. Ghaffaripour, S. Eskandarzade, M. Ghaini, M. Marjani, A. Moniri, M. Migaud, J. Casanova, A. Puel, A. Velayati. 2020. Delay in the Diagnosis of APECED: A Case Report and Review of Literature from Iran. *Immunol. Invest.* 49(3):299-306. 10.1080/08820139.2019.1671451.

79. Ma, Y., X. Wang, R. Li. 2022. AIRE gene mutation predisposing chronic mucocutaneous candidiasis and pigmented retinitis in two kids from a Chinese family. *Emerg Microbes Infect*. 11(1):1705-6. 10.1080/22221751.2022.2090860.

80. Puzenat, E., L. Pepin, A.M. Bertrand, F. Pelletier, D. Monnier, J. Levang, I. Mermet, P. Humbert, F. Aubin. 2010. [One case of type 1 auto-immune polyendocrinopathy or APECED]. *Ann. Dermatol. Venereol.* 137(12):794-8. 10.1016/j.annder.2010.08.014.

81. Fierabracci, A., M. Lanzillotta, I. Vorgucin, A. Palma, D. Katanic, C. Betterle. 2021. Report of two siblings with APECED in Serbia: is there a founder effect of c.769C>T AIRE genotype? *Ital. J. Pediatr.* 47(1):126. 10.1186/s13052-021-01075-8.

82. Bakrac, M., V. Jurisic, T. Kostic, V. Popovic, S. Pekic, N. Kraguljac, M. Colovic. 2007. Pure red cell aplasia associated with type I autoimmune polyglandular syndrome-successful response to treatment with mycophenolate mofetil: case report and review of literature. *J. Clin. Pathol.* 60(6):717-20. 10.1136/jcp.2006.042671.

83. Jovic, N.S., M. Nesovic, D.N. Vranjesevic, J. Ciric, D.M. Marinkovic, B. Bonaci. 1996. The Vogt-Koyanagi-Harada syndrome: association with autoimmune polyglandular syndrome type 1. *Postgrad. Med. J.* 72(850):495-7. 10.1136/pgmj.72.850.495.

84. Meyer, G., H. Donner, J. Herwig, H. Bohles, K.H. Usadel, K. Badenhoop. 2001. Screening for an AIRE-1 mutation in patients with Addison's disease, type 1 diabetes, Graves' disease and Hashimoto's thyroiditis as well as in APECED syndrome. *Clin. Endocrinol. (Oxf.)*. 54(3):335-8. 10.1046/j.1365-2265.2001.01230.x.

85. Myhre, A.G., M. Halonen, P. Eskelin, O. Ekwall, H. Hedstrand, F. Rorsman, O. Kampe, E.S. Husebye. 2001. Autoimmune polyendocrine syndrome type 1 (APS I) in Norway. *Clin. Endocrinol. (Oxf.)*. 54(2):211-7. 10.1046/j.1365-2265.2001.01201.x.

86. Buzi, F., R. Badolato, C. Mazza, S. Giliani, L.D. Notarangelo, G. Radetti, A. Plebani, L.D. Notarangelo. 2003. Autoimmune polyendocrinopathy-candidiasis-ectodermal dystrophy syndrome: time to review diagnostic criteria? *J. Clin. Endocrinol. Metab.* 88(7):3146-8. 10.1210/jc.2002-021495.

87. Finnish-German, A.C. 1997. An autoimmune disease, APECED, caused by mutations in a novel gene featuring two PHD-type zinc-finger domains. *Nat. Genet.* 17(4):399-403. 10.1038/ng1297-399.

88. Yukina, M., T. Erofeeva, N. Nuralieva, T. Andreeva, E. Savvateeva, N. Dudko, E. Troshina, E. Rogaev, G. Melnichenko. 2021. Novel Gene Mutations Regulating Immune Responses in Autoimmune Polyglandular Syndrome With an Atypical Course. *J Endocr Soc*. 5(8):bvab077. 10.1210/jendso/bvab077.

89. Chen, J., T. Lu, C. Liu, Y. Zhao, A. Huang, X. Hu, M. Li, R. Xiang, M. Feng, H. Lu. 2021. Autoimmune polyglandular syndrome type 1 with diabetes insipidus: a case report. *BMC Endocr. Disord.* 21(1):154. 10.1186/s12902-021-00822-6.

90. Gavrilova, T. 2019. Primary Immunodeficiency with Severe Multi-Organ Immune Dysregulation. *Case Reports Immunol*. 2019:8746249. 10.1155/2019/8746249.

91. Proust-Lemoine, E., P. Saugier-Veber, D. Lefranc, S. Dubucquoi, A. Ryndak, D. Buob, J.D. Lalau, R. Desailloud, J. Weill, L. Prin, H. Lefebvre, J.L. Wemeau. 2010. Autoimmune polyendocrine syndrome type 1 in north-western France: AIRE gene mutation specificities and severe forms needing immunosuppressive therapies. *Horm. Res. Paediatr.* 74(4):275-84. 10.1159/000297714.

92. Fierabracci, A., C. Bizzarri, A. Palma, A. Milillo, E. Bellacchio, M. Cappa. 2012. A novel heterozygous mutation of the AIRE gene in a patient with autoimmune polyendocrinopathy-candidiasis-ectodermal dystrophy syndrome (APECED). *Gene*. 511(1):113-7. 10.1016/j.gene.2012.09.029.

93. Lankisch, T.O., O. Mourier, E.M. Sokal, D. Habes, F. Lacaille, L. Bridoux-Henno, B. Hermeziu, C. Lenaerts, C.P. Strassburg, E. Jacquemin. 2009. AIRE gene analysis in children with autoimmune hepatitis type I or II. *J. Pediatr. Gastroenterol. Nutr.* 48(4):498-500. 10.1097/MPG.0b013e31818550de.

94. Oftedal, B.E., A. Hellesen, M.M. Erichsen, E. Bratland, A. Vardi, J. Perheentupa, E.H. Kemp, T. Fiskerstrand, M.K. Viken, A.P. Weetman, S.J. Fleishman, S. Banka, W.G. Newman, W.A. Sewell, L.S. Sozaeva, T. Zayats, K. Haugarvoll, E.M. Orlova, J. Haavik, S. Johansson, P.M. Knappskog, K. Lovas, A.S. Wolff, J. Abramson, E.S. Husebye. 2015. Dominant Mutations in the Autoimmune Regulator AIRE Are Associated with Common Organ-Specific Autoimmune Diseases. *Immunity*. 42(6):1185-96. 10.1016/j.immuni.2015.04.021.

95. Soderbergh, A., F. Rorsman, M. Halonen, O. Ekwall, P. Bjorses, O. Kampe, E.S. Husebye. 2000. Autoantibodies against aromatic L-amino acid decarboxylase identifies a subgroup of patients with Addison's disease. *J. Clin. Endocrinol. Metab.* 85(1):460-3. 10.1210/jcem.85.1.6266.

96. Linglart, A., A. Rothenbuhler, I. Gueorgieva, P. Lucchini, C. Silve, P. Bougneres. 2011. Long-term results of continuous subcutaneous recombinant PTH (1-34) infusion in children with refractory hypoparathyroidism. *J. Clin. Endocrinol. Metab.* 96(11):3308-12. 10.1210/jc.2011-1359.

97. Lundberg, C., B. Martinez, T.A. Banks. 2015. Expanding the spectrum: chronic urticaria and autoimmune polyendocrinopathy-candidiasis-ectodermal dystrophy. *Ann. Allergy. Asthma. Immunol.* 114(4):353-4. 10.1016/j.anai.2015.01.014.

98. Sahoo, S.K., G. Zaidi, R. Srivastava, A.N. Sarangi, N. Bharti, D. Eriksson, S. Bensing, O. Kampe, A. Aggarwal, R. Aggarwal, E. Bhatia. 2016. Identification of autoimmune polyendocrine syndrome type 1 in patients with isolated hypoparathyroidism. *Clin. Endocrinol. (Oxf.)*. 85(4):544-50. 10.1111/cen.13111.

99. Boe, A.S., P.M. Knappskog, A.G. Myhre, J.I. Sorheim, E.S. Husebye. 2002. Mutational analysis of the autoimmune regulator (AIRE) gene in sporadic autoimmune Addison's disease can reveal patients with unidentified autoimmune polyendocrine syndrome type I. *Eur. J. Endocrinol.* 146(4):519-22. 10.1530/eje.0.1460519.

100. Millar, S., D. Carson. 2012. Clinical phenotypes of autoimmune polyendocrinopathycandidiasis-ectodermal dystrophy seen in the Northern Ireland paediatric population over the last 30 years. *Ulster Med. J.* 81(3):118-22.

101. Harris, M., O. Kecha, C. Deal, C.R. Howlett, D. Deiss, V. Tobias, J. Simoneau-Roy, J. Walker. 2003. Reversible metaphyseal dysplasia, a novel bone phenotype, in two unrelated children with autoimmunepolyendocrinopathy-candidiasis-ectodermal dystrophy: clinical and molecular studies. *J. Clin. Endocrinol. Metab.* 88(10):4576-85. 10.1210/jc.2003-030089.

102. Crossland, K.L., M. Abinun, P.D. Arkwright, T.D. Cheetham, S.H. Pearce, C.M. Hilkens, D. Lilic. 2016. AIRE is not essential for the induction of human tolerogenic dendritic cells. *Autoimmunity*. 49(4):211-8. 10.3109/08916934.2016.1148692.

103. Cayir, A., R.I. Engin, M.I. Turan, E. Pala. 2014. Psoriasis vulgaris and autoimmune polyendocrine syndrome type I: a case report. *J. Pediatr. Endocrinol. Metab.* 27(7-8):791-3. 10.1515/jpem-2013-0472.

104. Li, D., E.A. Streeten, A. Chan, W. Lwin, L. Tian, R. Pellegrino da Silva, C.E. Kim, M.S. Anderson, H. Hakonarson, M.A. Levine. 2017. Exome Sequencing Reveals Mutations in AIRE as a Cause of Isolated Hypoparathyroidism. *J. Clin. Endocrinol. Metab.* 102(5):1726-33. 10.1210/jc.2016-3836.

105. Kendall-Taylor, P., A. Lambert, R. Mitchell, W.R. Robertson. 1988. Antibody that blocks stimulation of cortisol secretion by adrenocorticotrophic hormone in Addison's disease. *Br. Med. J. (Clin. Res. Ed)*. 296(6635):1489-91. 10.1136/bmj.296.6635.1489.

106. Perry, R., O. Kecha, J. Paquette, C. Huot, G. Van Vliet, C. Deal. 2005. Primary adrenal insufficiency in children: twenty years experience at the Sainte-Justine Hospital, Montreal. *J. Clin. Endocrinol. Metab.* 90(6):3243-50. 10.1210/jc.2004-0016.

107. Ward, L., J. Paquette, E. Seidman, C. Huot, F. Alvarez, P. Crock, E. Delvin, O. Kampe, C. Deal. 1999. Severe autoimmune polyendocrinopathy-candidiasis-ectodermal dystrophy in an adolescent girl with a novel AIRE mutation: response to immunosuppressive therapy. *J. Clin. Endocrinol. Metab.* 84(3):844-52. 10.1210/jcem.84.3.5580.

108. Saugier-Veber, P., N. Drouot, L.M. Wolf, J.M. Kuhn, T. Frebourg, H. Lefebvre. 2001. Identification of a novel mutation in the autoimmune regulator (AIRE-1) gene in a French family with autoimmune polyendocrinopathy-candidiasis-ectodermal dystrophy. *Eur. J. Endocrinol.* 144(4):347-51. 10.1530/eje.0.1440347.

109. Tsai, S.L., J. Green, L.A. Metherell, F. Curtis, B. Fernandez, A. Healey, J. Curtis. 2016. Primary Adrenocortical Insufficiency Case Series: Genetic Etiologies More Common than Expected. *Horm. Res. Paediatr.* 85(1):35-42. 10.1159/000441843.

110. Sajjadi-Jazi, S.M., A. Soltani, S. Enayati, A. Kakavand Hamidi, M.M. Amoli. 2019. Autoimmune Polyglandular Syndrome Type 1: a case report. *BMC Med. Genet.* 20(1):143. 10.1186/s12881-019-0870-3.

111. Lima Ferreira, J., F. Simoes de Carvalho, A.P. Marques, R.M. Principe. 2020. Hypoparathyroidism as the single major component for decades of autoimmune polyglandular syndrome type 1. *Endocrinol Diabetes Metab Case Rep*. 2020. 10.1530/EDM-20-0083.

112. Bruserud, O., E. Bratland, A. Hellesen, N. Delaleu, H. Reikvam, B.E. Oftedal, A.S.B. Wolff. 2017. Altered Immune Activation and IL-23 Signaling in Response to Candida albicans in Autoimmune Polyendocrine Syndrome Type 1. *Front. Immunol.* 8:1074. 10.3389/fimmu.2017.01074.

113. Sanford, E., K. Watkins, S. Nahas, M. Gottschalk, N.G. Coufal, L. Farnaes, D. Dimmock, S.F. Kingsmore, R. Investigators. 2018. Rapid whole-genome sequencing identifies a novel AIRE variant associated with autoimmune polyendocrine syndrome type 1. *Cold Spring Harb Mol Case Stud*. 4(3). 10.1101/mcs.a002485.

114. Meyer, T., V. Ruppert, K. Karatolios, B. Maisch. 2007. Hereditary long QT syndrome due to autoimmune hypoparathyroidism in autoimmune polyendocrinopathy-candidiasis-ectodermal dystrophy syndrome. *J. Electrocardiol.* 40(6):504-9. 10.1016/j.jelectrocard.2006.12.013.

115. Perniola, R., O. Filograna, G. Greco, V. Pellegrino. 2008. High prevalence of thyroid autoimmunity in Apulian patients with autoimmune polyglandular syndrome type 1. *Thyroid*. 18(9):1027-9. 10.1089/thy.2008.0027.

116. Perniola, R., G. Lobreglio, M.C. Rosatelli, E. Pitotti, E. Accogli, C. De Rinaldis. 2005. Immunophenotypic characterisation of peripheral blood lymphocytes in autoimmune polyglandular syndrome type 1: clinical study and review of the literature. *J. Pediatr. Endocrinol. Metab.* 18(2):155-64. 10.1515/jpem.2005.18.2.155.
