# Supplementary material for: Clinical, genetic, and immunologic features of APS-1 patients from the Middle East, and a review of the literature
Source: J Hum Immun. 2026 Aug 3;2(5):e20250254. doi: 10.70962/jhi.20250254 (PMC13431174; doi:10.70962/jhi.20250254)
Supplement: Table S2 — shows list of all clinical manifestations and their prevalence in APS-1 patients reported in the literature. [file jhi_20250254_tables2.docx]

**Supplemental Table 2.** List of all clinical manifestations and their prevalence in APS-1 patients reported in the literature.

| Clinical manifestation | Prevalence |
| --- | --- |
| Hypoparathyroidism | 86.4% |
| Candidiasis | 82.1% |
| Adrenal insufficiency | 73.0% |
| Alopecia | 25.2% |
| Other unspecified endocrinological dysfunctions^*^ | 17.6% |
| Ophthalmic symptoms | 13.7% |
| Hepatitis | 13.3% |
| Thyroid disease | 13.0% |
| Dermatologic manifestations^†^ | 11.6% |
| Ectodermal dystrophy | 11.6% |
| Anemia | 11.3% |
| Malabsorption | 10.3% |
| Dental abnormalities | 9.5% |
| Diabetes mellitus | 8.0% |
| Other unspecified gastrointestinal dysfunctions^#^ | 6.2% |
| Gastritis | 4.8% |
| Renal disease | 3.6% |
| Joints and skeletal disorder | 3.2% |
| Atopy | 2.9% |
| Neurologic symptoms | 2.9% |
| Respiratory/pulmonary disease | 2.1% |
| Spleen disorder | 1.7% |
| Head and neck manifestations | 1.5% |
| Ear symptoms | 1.4% |
| Malignancy | 1.4% |
| Pancreatic insufficiency | 1.2% |
| Sicca/Sjogren syndrome | 1.2% |
| Cardiac abnormalities | 0.8% |
| Cholelithiasis | 0.8% |
| Vasculitis | 0.6% |
| Connective tissue disorder | 0.5% |
| Diabetes insipidus | 0.5% |
| Facial dysmorphism | 0.5% |
| Hypoglycemia | 0.3% |
| Recurrent fever | 0.3% |
| Weakness | 0.3% |
| Weight/appetite changes | 0.3% |
| Enlarged thymus | 0.2% |
| Laboratory abnormalities | 0.2% |
| Raynaud’s disease | 0.2% |
| Recurrent infections | 0.2% |

^*^Hypothalamic-pituitary axis disorders; Growth retardation; Pubertal and gonadal disorders. **^† #^**Functional GI disorders (diarrhea, constipation); Autoimmune enteritis and colitis (Crohn’s disease, celiac disease, eosinophilic ileitis).
