# Supplementary material for: Clinical, genetic, and immunologic features of APS-1 patients from the Middle East, and a review of the literature
Source: J Hum Immun. 2026 Aug 3;2(5):e20250254. doi: 10.70962/jhi.20250254 (PMC13431174; doi:10.70962/jhi.20250254)
Supplement: Table S3 — shows list of all homozygous AIRE mutations in APS-1 patients reported in the literature. [file jhi_20250254_tables3.docx]

**Supplemental Table 3.** List of all homozygous *AIRE* mutations in APS-1 patients reported in the literature.

| Mutation | Protein (Affected domain) | Prevalence |
| --- | --- | --- |
| c.769C>T | p.R257X (SAND) | 44.8% |
| c.967_979del13bp | p.L323fs (PHD1) | 22.9% |
| c.232T>C | p.W78R (HSR/CARD) | 5.2% |
| c.254A>G | p.Y85C (HSR/CARD) | 4.0% |
| c.415C>T | p.R139X (Downstream of NLS) | 4.0% |
| c.205_208dupCAGG | p.D70AfsX148 (HSR/CARD) | 2.0% |
| c.1616C>T | p.P539L (TAD) | 1.4% |
| c.199_202delCTGinsTGG | p.L67WfsX80 (L) | 1.2% |
| c.1193delC | p.P398fs (PRR) | 1.2% |
| c.44G>A | p.R15H (HSR/CARD) | 1.0% |
| c.47C>T | p.T16M (HSR/CARD) | 1.0% |
| c.607C>T | p.R203X (SAND) | 1.0% |
| c.1095+2T>A | p.E9del (PHD1, PRR) | 0.8% |
| c.21_43dup23 | p.R15fs (HSR/CARD) | 0.6% |
| c.463G>A | p.G155S (downstream of NLS) | 0.6% |
| c.995+(3_5)delGAGinsTAT | p.E8del (PHD1) | 0.6% |
| c.1244_1245insC | p.L417fs (L) | 0.6% |
| c.1A>G | p.M1V (HSR/CARD) | 0.4% |
| c.62C>T | p.A21V (HSR/CARD) | 0.4% |
| c.132+1_132+3delGTGinsCT | Abolishes E1-E2 splicing (NLS) | 0.4% |
| c.239T>G | p.V80G (HSR/CARD) | 0.4% |
| c.274C>T | p.R92W (HSR/CARD) | 0.4% |
| c.463+2T>C | p.E3del (NLS) | 0.4% |
| c.789delC | p.G263fs (SAND) | 0.4% |
| c.879+1G>A | p.E7del (SAND) | 0.4% |
| c.958delC | p.L320fs (PHD1) | 0.4% |
| c.1096-1G>A | p.E10del (PRR, L) | 0.4% |
| c.93_94insT | p.L32SfsX3 (HSR/CARD) | 0.2% |
| c.173C>A | p.A58D (HSR/CARD) | 0.2% |
| c.195G>A | p.W65X (L) | 0.2% |
| c.206A>C | p.Q69P (HSR/CARD) | 0.2% |
| c.267_275del9 | p.Y90-R92del (HSR/CARD) | 0.2% |
| c.308-1G>C | p.E3del (NLS) | 0.2% |
| c.328delC | pR110fs (NLS) | 0.2% |
| c.396G>C | p.R132S (NLS) | 0.2% |
| c.483_484insC | p.163fs (downstream of NLS) | 0.2% |
| c.560C>G | p.S187X (upstream of SAND) | 0.2% |
| c.906T>A | p.C302X (PHD1) | 0.2% |
| c.1064_1068dupCCCGG | p.Q358fs (PRR) | 0.2% |
| c.1103dupC | p.L370fs (PRR) | 0.2% |
| c.1111ins4bp | p.R371fs (PRR) | 0.2% |
| c.1249dupC | p.L417fs (L) | 0.2% |
| c.1311C>A | p.C437X (PHD2) | 0.2% |
| c.1497delT | p.A500fs (upstream of TAD) | 0.2% |
| IVS1_IVS4 | p.E2-E4del (L,HSR/CARD,NLS) | 0.2% |

*Abbreviations*: dup, Duplication; del, Deletion; Ins, Insertion; IVS, intervening sequence; bp, base pair; HSR, Homogeneously staining region; CARD, Caspase activation and recruitment domain; L, Linker region; SAND, Sp100, AIRE-1, NucP41/75, DEAF-1 domain; PHD, Plant homeodomain; PRR, Proline-rich region; TAD, Transactivation domain.
