# Supplementary material for: Clinical, genetic, and immunologic features of APS-1 patients from the Middle East, and a review of the literature
Source: J Hum Immun. 2026 Aug 3;2(5):e20250254. doi: 10.70962/jhi.20250254 (PMC13431174; doi:10.70962/jhi.20250254)
Supplement: Table S4 — shows list of all heterozygous AIRE mutations in APS-1 patients reported in the literature. [file jhi_20250254_tables4.docx]

**Supplemental Table 4.** List of all heterozygous *AIRE* mutations in APS-1 patients reported in the literature.

| Mutation | Protein (Affected Domain) | Prevalence |
| --- | --- | --- |
| c.769C>T | p.R257X (SAND) | 52.5% |
| c.967_979del13bp | p.L323fs (PHD1) | 28.6% |
| c.232T>C | p.W78R (HSR/CARD) | 9.1% |
| c.47C>T | p.T16M (HSR/CARD) | 6.2% |
| c.62C>T | p.A21V (HSR/CARD) | 4.7% |
| c.932G>A | p.C311Y (PHD1) | 3.5% |
| c.977C>T | p.P326L (PHD1) | 3.1% |
| c.607C>T | p.R203X (SAND) | 2.9% |
| c.1072C>T | p.Q358X (PRR) | 2.9% |
| c.682G>T | p.G228W (SAND) | 2.0% |
| c.1616C>T | p.P539L (TAD) | 1.8% |
| c.1249dupC | p.L417fs (L) | 1.8% |
| c.1163_1164insA | p.M388fs (PRR) | 1.8% |
| c.1242_1243insA | p.H415fs (L) | 1.8% |
| c.415C>T | p.R139X (Downstream of NLS) | 1.5% |
| c.274C>T | p.R92W (HSR/CARD) | 1.5% |
| c.260T>C | p.L87P (HSR/CARD) | 1.5% |
| c.755C>T | p.P252L (SAND) | 1.5% |
| c.21_43dup23 | p.R15fs (HSR/CARD) | 1.2% |
| c.1249delC | p.L417fs (L) | 1.2% |
| c.1344delC | p.C449fs (PHD2) | 1.2% |
| c.1638A>T | p.X546C+59aa | 1.2% |
| c.995+(3_5)delGAGinsTAT | p.E8del (PHD1) | 0.9% |
| c.1A>G | p.M1V (HSR/CARD) | 0.9% |
| c.132+1_132+3delGTGinsCT | Abolishes E1-E2 splicing (NLS) | 0.9% |
| c.83T>C | p.L28P (HSR/CARD) | 0.9% |
| c.190_226del37 | p.S64_L75delfs (L , HSR/CARD) | 0.9% |
| c.290T>C | p.L97P (HSR/CARD) | 0.9% |
| c.905G>A | p.C302Y (PHD1) | 0.9% |
| c.1264_1265insA | p.P422fs (PRR) | 0.9% |
| c.1244_1245insC | p.H415fs (L) | 0.6% |
| c.463+2T>C | p.E3del (NLS) | 0.6% |
| g.(?_44285987)_(44297747_?)del | Complete deletion of AIRE | 0.6% |
| c.1A>T | p.M1L (HSR/CARD) | 0.6% |
| c.2T>C | p.M1T (HSR/CARD) | 0.6% |
| c.22C>T | p.R8C (L) | 0.6% |
| c.64_69delGTGGAC | p.V22_D23del (HSR/CARD) | 0.6% |
| c.271A>G | p.Y90C (HSR/CARD) | 0.6% |
| c.278T>G | p.L93R (HSR/CARD) | 0.6% |
| c.319_321delAGCinsTG | p.S107fs (upstream of NLS) | 0.6% |
| c.821delG | p.G274fs (SAND) | 0.6% |
| c.892G>A | p.E298K (upstream of PHD1) | 0.6% |
| c.901G>A | p.V301M (PHD1) | 0.6% |
| c.931delT | p.C311fs (PHD1) | 0.6% |
| c.1066C>T | p.R356W(PRR) | 0.6% |
| c.1103_1104insC | p.L370fs (PRR) | 0.6% |
| c.1264delC | p.P422fs (PRR) | 0.6% |
| c.1336T>G | p.C446G (PHD2) | 0.6% |
| c.1496delC | p.P499LfsX22 (downstream of PHD2) | 0.6% |
| c.1503delG | p.A505fs (TAD) | 0.6% |
| c.254A>G | p.Y85C (HSR/CARD) | 0.3% |
| c.1193delC | p.P398fs (PRR) | 0.3% |
| c.239T>G | p.V80G (HSR/CARD) | 0.3% |
| c.789delC | p.G263fs (SAND) | 0.3% |
| c.195G>A | p.W65X (L) | 0.3% |
| c.560C>G | p.S187X (upstream of SAND | 0.3% |
| c.1096-1G>A | p.E10del (PRR, L) | 0.3% |
| c.1-7_538+20del | p.E1-E4del (HSR/CARD, L, NLS) | 0.3% |
| c.32T>C | p.L11P (L) | 0.3% |
| c.38T>G | p.L13R (HSR/CARD) | 0.3% |
| c.44G>T | p.R15L (HSR/CARD) | 0.3% |
| c.55G>A | p.A19T (HSR/CARD) | 0.3% |
| c.169C>T | p.Q57X (HSR/CARD) | 0.3% |
| c.202A>C | p.T68P (HSR/CARD) | 0.3% |
| c.232T>A | p.W78R (HSR/CARD) | 0.3% |
| c.247A>G | p.K83E (HSR/CARD) | 0.3% |
| c.268T>C | p.Y90H (HSR/CARD) | 0.3% |
| c.361delG | p.A121fs (NLS) | 0.3% |
| c.371C>T | p.P124L (NLS) | 0.3% |
| c.402delC | p.S135QfsX12 (downstream of NLS) | 0.3% |
| c.462A>T | p.P154P (Silent mutation affecting E3 splicing resulting in p.E3del) (downstream of NLS) | 0.3% |
| c.517C>T | p.Q173X (downstream of NLS) | 0.3% |
| c.522_523ins13 | p.L175fs (downstream of NLS) | 0.3% |
| c.540delG | p.G180fs (upstream of SAND) | 0.3% |
| c.622G>T | p.G208W (SAND) | 0.3% |
| c.623G>T | p.G208V (SAND) | 0.3% |
| c.653-7_-5delCTC | p.G218fs (SAND) | 0.3% |
| c.892G>T | p.E298K (upstream of PHD1) | 0.3% |
| c.908G>C | p.R303P (PHD1) | 0.3% |
| c.913G>A | p.G305S (PHD1) | 0.3% |
| c.946C>T | p.R316W (PHD1) | 0.3% |
| c.961C>G | p.S278R (SAND) | 0.3% |
| c.1033_1034delGT | p.V345fs (downstream of PHD1) | 0.3% |
| c.1053_1060del8 | p.R351fs (PRR) | 0.3% |
| c.1095+1G>A | p.E9del (PHD1) | 0.3% |
| c.1118C>T | p.A373V (PRR) | 0.3% |
| c.1189delC | p.L397fs (PRR) | 0.3% |
| c.1195G>C | p.A399P (PRR) | 0.3% |
| c.1214delC | p.P405fs (PRR) | 0.3% |
| c.1265delC | p.P422LfsX58 (PRR) | 0.3% |
| c.1283_1284insA | p.L428fs (PRR) | 0.3% |
| c.1347C>A | p.C449X (PHD2) | 0.3% |
| c.1370-1371insG | p.C457fs (PHD2) | 0.3% |
| c.1422insAC | p.C475fs (downstream of PHD2) | 0.3% |
| c.1450G>A | p.V484M (downstream of PHD2) | 0.3% |
| c.1567-2A>G | IVS-2A>G | 0.3% |
| IVS8 | p.E8del (PHD1) | 0.3% |
| IVS9-1G>A | p.E9del (PHD1/PRR) | 0.3% |
| IVS9+5G>T | p.E9del (PHD1, PRR) | 0.3% |
| CNV of 21q22.3 | Duplication including E1-E2 | 0.3% |

*Abbreviations*: dup, Duplication; del, Deletion; Ins, Insertion; IVS, intervening sequence; bp, base pair; HSR, Homogeneously staining region; CARD, Caspase activation and recruitment domain; L, Linker region; SAND, Sp100, AIRE-1, NucP41/75, DEAF-1 domain; PHD, Plant homeodomain; PRR, Proline-rich region ; TAD, Transactivation domain.
