# Supplementary material for: Clinical, genetic, and immunologic features of APS-1 patients from the Middle East, and a review of the literature
Source: J Hum Immun. 2026 Aug 3;2(5):e20250254. doi: 10.70962/jhi.20250254 (PMC13431174; doi:10.70962/jhi.20250254)
Supplement: Table S5 — shows list of compound heterozygous, autosomal dominant, and autosomal recessive AIRE mutations in APS-1 patients and patients with a milder phenotype reported in the literature. [file jhi_20250254_tables5.docx]

**Supplemental Table 5.** List of compound heterozygous, autosomal dominant, and autosomal recessive *AIRE* mutations in APS-1 patients and patients with a milder phenotype reported in the literature.

| Compound heterozygous |  |
| --- | --- |
| cDNA | Protein (Domain) |
| g.(?_44285987)_(44297747_?)del/c.967_979del13bp | Complete deletion of AIRE/p.L323fs (PHD1) |
| c.1A>G/c.274C>T | p.M1V (HSR/CARD)/p.R92W (HSR/CARD) |
| c.1A>T/c.769C>T | p.M1L (HSR/CARD)/p.R257X (SAND) |
| c.2T>C/c.769C>T | p.M1T (HSR/CARD)/p.R257X (SAND) |
| c.21_43dup23bp/c.769C>T | p.R15fs (HSR/CARD)/p.R257X (SAND) |
| c.22C>T/c.290T>C | p.R8C (L)/p.L97P (HSR/CARD) |
| c.22C>T/c.402delC | p.R8C (L)/p.S135QfsX12 (downstream of NLS) |
| c.47C>T/c.47C>T+c.755C>T | p.T16M (HSR/CARD)/p.T16M (HSR/CARD)+P252L (SAND) |
| c.47C>T/c.62C>T | p.T16M (HSR/CARD)/p.A21V (HSR/CARD) |
| c.47C>T/c.169C>T | p.T16M (HSR/CARD)/p.Q57X (HSR/CARD) |
| c.47C>T/c.232T>C | p.T16M (HSR/CARD)/p.W78R (HSR/CARD) |
| c.47C>T/c.319_321delAGCinsTG | p.T16M (HSR/CARD)/p.S107fs (upstream of NLS) |
| c.47C>T/c.769C>T | p.T16M (HSR/CARD)/p.R257X (SAND) |
| c.47C>T/c.821delG | p.T16M (HSR/CARD)/p.G274fs (SAND) |
| c.47C>T/c.892G>A | p.T16M (HSR/CARD)/p.E298K (upstream of PHD1) |
| c.47C>T/c.967_979del13bp | p.T16M (HSR/CARD)/p.L323fs (PHD1) |
| c.62C>T/c.769C>T | p.A21V (HSR/CARD)/p.R257X (SAND) |
| c.62C>T/c.967_979del13bp | p.A21V (HSR/CARD)/p.L323fs (PHD1) |
| c.62C>T/c.1096-1G>A | p.A21V (HSR/CARD)/E10del (PRR, L) |
| c.62C>T/c.1163_1164insA | p.A21V (HSR/CARD)/p.M388fs (PRR) |
| c.83T>C/c.1249delC | p.L28P (HSR/CARD)/p.L417fs (L) |
| c.132+1_132+3delGTGinsCT/c.769C>T | Abolishes E1-E2 splicing (NLS)/p.R257X (SAND) |
| c.195G>A/c.769C>T | p.W65X (L)/p.R25X (SAND) |
| c.232T>C/c.62C>T | p.W78R (HSR/CARD)/p.A21V (HSR/CARD) |
| c.232T>A/c.64_69delGTGGAC | p.W78R (HSR/CARD)/p.V22_D23del (HSR/CARD) |
| c.232T>C/c.361delG | p.W78R (HSR/CARD)/p.A121fs (NLS) |
| c.232T>C/c.755C>T | p.W78R (HSR/CARD)/p.P252L (SAND) |
| c.232T>C/c.769C>T | p.W78R (HSR/CARD)/p.R257X (SAND) |
| c.232T>C/IVS8 | p.W78R (HSR/CARD)/E8del (PHD1) |
| c.232T>C/c.1072C>T | p.W78R (HSR/CARD)/p.Q358X (PRR) |
| c.232T>C/c.1496delC | p.W78R (HSR/CARD)/p.P499LfsX22 (downstream of PHD2) |
| c.239T>G/CNV of 21q22.3 (chr21:45,670,150–45,706,528)*1 | p.V80G (HSR/CARD)/duplication including E1-E2 |
| c.260T>C/c.967_979del13bp | p.L87P (HSR/CARD)/p.L323fs (PHD1) |
| c.271A>G/c.769C>T | p.Y90C (HSR/CARD)/p.R257X (SAND) |
| c.274C>T/c.967_979del13bp | p.R92W (HSR/CARD)/p.L323fs (PHD1) |
| c.371C>T/c.623G>T | p.P124L (NLS)/p.G208V (SAND) |
| c.415C>T/c.892G>T | p.R139X (downstream of NLS)/p.E298K (upstream of PHD1) |
| c.415C>T/c.967_979del13bp | p.R139X (downstream of NLS)/L323fs (PHD1) |
| c.463+2T>C/c.967_979del13bp | E3del (NLS)/p.L323fs (PHD1) |
| c.517C>T/IVS9-1G>A | p.Q173X (downstream of NLS)/E9del (PHD1, PRR) |
| c.522_523ins13/c.967_979del13bp | p.L175fs (downstream of NLS)/p.L323fs (PHD1) |
| c.560C>G/c.1072C>T | p.S187X (upstream of SAND)/p.Q358X (PRR) |
| c.607C>T/c.415C>T | p.R203X (SAND)/p.R139X (downstrem of NLS) |
| c.607C>T/c.769C>T | p.R203X (SAND)/p.R257X (SAND) |
| c.607C>T/IVS9+5G>T | p.R203X (SAND)/E9del (PHD1, PRR) |
| c.769C>T/c.32T>C | p.R257X (SAND)/p.L11P (L) |
| c.769C>T/c.55G>A | p.R257X (SAND)/p.A19T (HSR/CARD) |
| c.769C>T/c.232T>C | p.R257X (SAND)/p.W78R (HSR/CARD) |
| c.769C>T/c.247A>G | p.R257X (SAND)/p.K83E (HSR/CARD) |
| c.769C>T/c.462A>T | p.R257X (SAND)/p.P154P (Silent mutation affecting E3 splicing resulting in E3del) (downstream of NLS) |
| c.769C>T/c.540delG | p.R257X (SAND)/p.G180fs (upstream of SAND) |
| c.769C>T/c.653-7_-5delCTC | p.R257X (SAND)/p.G218fs (SAND) |
| c.769C>T/c.821delG | p.R257X (SAND)/p.G274fs (SAND) |
| c.769C>T/c.892G>A | p.R257X (SAND)/p.E298K (upstream of PHD1) |
| c.769C>T/c.932G>A | p.R257X (SAND)/p.C311Y (PHD1) |
| c.769C>T/c.967_979del13bp | p.R257X (SAND)/p.L323fs (PHD1) |
| c.769C>T/c.977C>T | p.R257X (SAND)/p.P326L (PHD1) |
| c.769C>T/c.1053_1060del8 | p.R257X (SAND)/p.R351fs (PRR) |
| c.769C>T/c.1095+1G>A | p.R257X (SAND)/E9del (PHD1) |
| c.769 C>T/c.1214delC | p.R257X (SAND)/p.P405fs (PRR) |
| c.769C>T/c.1242_1243insA | p.R257X (SAND)/p.H415fs (L) |
| c.769C>T/c.1264_1265insA | p.R257X (SAND)/p.P422fs (PRR) |
| c.769C>T/c.1336T>G | p.R257X (SAND)/p.C446G (PHD2) |
| c.769C>T/c.1344delC | p.R257X (SAND)/p.C449fs (PHD2) |
| c.769C>T/c.1370-1371insG | p.R257X (SAND)/p.C457fs (PHD2) |
| c.769C>T/c.1567-2A>G | p.R257X (SAND)/IVS-2A>G |
| c.769C>T/c.1616C>T | p.R257X (SAND)/p.P539L (TAD) |
| c.879+1G>A/c.879+1G>A | E7del/E7del (SAND) |
| c.905G>A/WT | p.C302Y (PHD1)/WT |
| c.932G>A/c.769C>T | p.C311Y (PHD1)/p.R257X (SAND) |
| c.967_979del13bp/c.1-7_538+20del | p.L323fs (PHD1)/E1-E4del (HSR/CARD, L, NLS) |
| c.967_979del13bp/c.38T>G | p.L323fs (PHD1)/p.L13R (HSR/CARD) |
| c.967_979del13bp/c.44G>T | p.L323fs (PHD1)/p.R15L (HSR/CARD) |
| c.967_979del13bp/c.62C>T | p.L323fs (PHD1)/p.A21V (HSR/CARD) |
| c.967_979del13bp/c.190_226del37 | p.L323fs (PHD1)/p.S64_L75delfs (L + HSR/CARD) |
| c.967_979del13bp/c.202A>C | p.L323fs (PHD1)/p.T68P (HSR/CARD) |
| c.967_979del13bp/c.260T>C | p.L323fs (PHD1)/p.L87P (HSR/CARD) |
| c.967_979del13bp/c.274C>T | p.L323fs (PHD1)/p.R92W (HSR/CARD**)** |
| c.967_979del13bp/c.278T>G | p.L323fs (PHD1)/p.L93R (HSR/CARD) |
| c.967_979del13bp/c.290T>C | p.L323fs (PHD1)/p.L97P (HSR/CARD) |
| c.967_979del13bp/c.769C>T | p.L323fs (PHD1)/p.R257X (SAND) |
| c.967_979del13bp/c.789delC | p.L323fs (PHD1)/p.G263fs (SAND) |
| c.967_979del13bp/c.946C>T | p.L323fs (PHD1)/p.R316W (PHD1) |
| c.967_979del13bp/c.967_979del13bp + c.290T>C | p.L323fs (PHD1)/p.L323fs (PHD1) + p.L97P (HSR/CARD) |
| c.967_979del13bp/c.977C>T | p.L323fs (PHD1)/p.P326L (PHD1) |
| c.967_979del13bp/c.995+(3_5)delGAGinsTAT | p.L323fs (PHD1)/E8del (PHD1) |
| c.967_979del13bp/c.1033_1034delGT | p.L323fs (PHD1)/p.V345fs (downstream of PHD1) |
| c.967_979del13bp/c.1195G>C | p.L323fs (PHD1)/p.A399P (PRR) |
| c.967_979del13bp/c.1244_1245insC | p.L323fs (PHD1)/p.H415fs (L) |
| c.967_979del13bp/c.1249delC | p.L323fs (PHD1)/p.L417fs (L) |
| c.967_979del13bp/c.1249dupC | p.L323fs (PHD1)/p.L417fs (L) |
| c.967–979del13bp/c.1163_1164insA | p.L323fs (PHD1)/p.M388fs (PRR) |
| c.967_979del13bp/c.1264delC | p.L323fs (PHD1)/p.P422fs (PRR) |
| c.967_979del13bp/c.1283_1284insA | p.L323fs (PHD1)/p.L428fs (PRR) |
| c.967_979del13bp/c.1347C>A | p.L323fs (PHD1)/p.C449X (PHD2) |
| c.967_979del13bp/c.1616C>T | p.L323fs (HSR/CARD)/p.P539L (TAD) |
| c.977C>T/c.769C>T | p.P326L (PHD1)/p.R257X (SAND) |
| c.977C>T/c.967_979del13bp | p.P326L (PHD1)/p.L323fs (PHD1) |
| c.1103_1104insC/c.1503delG | p.L370fs (PRR)/p.A505fs (TAD) |
| c.1249dupC/c.769C>T | p.L417fs (L)/p.R257X (SAND) |
| c.1249dupC/c.967–979del13bp | p.L417fs (L)/p.L323fs (PHD1) |
| c.1265delC/c.268T>C | p.P422LfsX58 (PRR)/p.Y90H (HSR/CARD) |
| c.1616C>T/c.260T>C | p.P539L (TAD)/p.L87P (HSR/CARD) |
| c.1616C>T/c.931delT | p.P539L (TAD)/p.C311fs (PHD1) |
| c.1616C>T/c.967_979del13 | p.P539L (TAD)/p.L323fs (PHD1) |
| Autosomal dominant |  |
| cDNA | Protein (Domain) |
| c.682G>T/WT | p.G228W (SAND)/WT |
| c.748A>T/WT | p/S250C (SAND)/WT |
| c.769C>T/WT | p.R257X (SAND)/WT |
| c.834C>G/WT | p.S278R (SAND)/WT |
| c.892G>A/WT | p.E298K (upstream of PHD1)/WT |
| c.901G>A/WT | p.V301M (PHD1)/WT |
| c.905G>A/WT | p.C302Y (PHD1)/WT |
| c.908G>C/WT | p.R303P (PHD1)/WT |
| c.913G>A/WT | p.G305S (PHD1)/WT |
| c.931delT/WT | p.C311fs (PHD1)/WT |
| c.934G>A/WT | p.D312N (PHD1)/WT |
| c.967_979del13bp/WT | p.L323fs (PHD1)/WT |
| c.977C>T/WT | p.P326L (PHD1)/WT |
| c.977C>A/WT | p.P326Q (PHD1)/WT |
| c.983G>A/WT | p.R328Q (PHD1)/WT |
| c.1189delC/WT | p.L397fs (PRR)/WT |
| c.1298delGT/WT | p.R433fs (PRR)/WT |
| c.1336T>G/WT | p.C446G (PHD2)/WT |
| Autosomal recessive |  |
| cDNA | Protein (Domain) |
| c.83T>C/WT | p.L28P (HSR/CARD)/WT |
| c.86T>C/WT | p.L29P (HSR/CARD)/WT |
| c.232T>C/WT | p.W78R (HSR/CARD)/WT |
| c.278T>G /WT | p.L93R (HSR/CARD)/WT |
| c.755C>T/WT | p.P252L (SAND)/WT |
| c.463+G>A/WT | E3del (NLS)/WT |
| c.932G>A/WT | p.C311Y (PHD1)/WT |
| c.1066C>T/WT | p.R356W (PRR)/WT |
| c.1322C>T/WT | p.T441M (PHD2)/WT |
| c.1411T>C/WT | p.R471C (PHD2)/WT |
| c.1450G>A/WT | p.V484M (downstream of PHD2)/WT |
| Recessive variants of uncertain significance |  |
| c.47C>T/WT | p.T16M (HSR/CARD)/WT |
| c.62C>T/ WT | p.A21V (HSR/CARD)/WT |
| c.132+1_132+3delGTGinsCT/WT | Abolishes E1-E2 splicing (NLS)/WT |
| c.254A>G/WT | p.Y85C (HSR/CARD)/WT |
| c.463+2T>C/WT | E3del (NLS)/WT |
| c.415C>T/WT | p.R139X (downstream of NLS)/WT |
| c.607C>T/WT | p.R203X (SAND)/WT |
| c.622G>T/WT | p.G208W (SAND)/WT |
| c.1118C>T/WT | p.A373V (PRR)/WT |
| c.1163_1164insA/WT | p.M388fs (PRR)/WT |
| c.1193delC/WT | p.P398fs (PRR)/WT |
| c.1242_1243insA/WT | p.H415fs (L)/WT |
| c.1244_1245insC/WT | p.L417fs (L)/WT |
| c.1422insAC/WT | p.C475fs (downstream of PHD2)/WT |
| c.1450G>A/WT | p.V484M (downstream of PHD2)/WT |
| c.1638A>T/WT | p.X546C+59aa/WT |

*Abbreviations*: dup, Duplication; del, Deletion; Ins, Insertion; IVS, intervening sequence; bp, base pair; HSR, Homogeneously staining region; CARD, Caspase activation and recruitment domain; L, Linker region; SAND, Sp100, AIRE-1, NucP41/75, DEAF-1 domain; PHD, Plant homeodomain; PRR, Proline-rich region ; TAD, Transactivation domain.
